# Introducing Rigidity into the GFP Chromophore via a Boron Bridge: Insights and Application in Two-Photon Imaging

**DOI:** 10.1021/acs.orglett.5c00284

**Published:** 2025-03-18

**Authors:** Attila Csomos, Brigitta Petrilla, Levente Cseri, Gábor Turczel, Arnold Steckel, Anett Matuscsák, Gitta Schlosser, Balázs J. Rózsa, Ervin Kovács, Zoltán Mucsi

**Affiliations:** † Femtonics Ltd., H-1087 Budapest, Hungary; □ ELTE Hevesy György PhD School of Chemistry, H-1117 Budapest, Hungary; ‡ BrainVisionCenter, H-1094 Budapest, Hungary; § NMR Research Laboratory, Centre for Structural Science, HUN-REN Research Centre for Natural Sciences, H-1117 Budapest, Hungary; ∥ MTA-ELTE Lendület (Momentum) Ion Mobility Mass Spectrometry Research Group, Faculty of Science, Institute of Chemistry, ELTE Eötvös Loránd University, Pázmány Péter sétány 1/A, H-1117 Budapest, Hungary; ⊥ Institute of Materials and Environmental Chemistry, HUN-REN Research Centre for Natural Sciences, H-1117 Budapest, Hungary; × The Faculty of Information Technology, Pázmány Péter Catholic University, H-1083 Budapest, Hungary; ∇ Laboratory of 3D Functional Network and Dendritic Imaging, HUN-REN Institute of Experimental Medicine, H-1083 Budapest, Hungary; ⊗ University of Miskolc, H-3515 Miskolc, Hungary

## Abstract

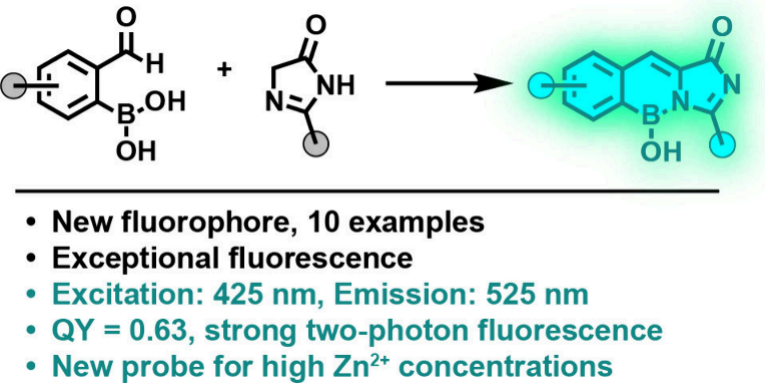

A new family of azaborine fluorophores, consisting of ten compounds, has been developed that mimics the chromophore of green fluorescent protein, with conformation locked by a hydroxyboron bridge. These fluorophores exhibit fluorescence Stokes shifts up to 100 nm (0.56 eV) and high brightness (>10^4^ M^–1^ cm^–1^) in the 408–525 nm range. Their potential utility for sensing applications was demonstrated by constructing zinc sensors capable of detecting 100 micromolar Zn^2+^ concentrations in biological systems with two-photon microscopy.

Fluorescence detection has a plethora of applications in analytical chemistry and bioimaging. Despite the diversity of requirements in these uses, the variety of available fluorophores is rather limited.^1−3^ Coumarins, rhodamines, fluoresceins, BODIPYs, and cyanines are used almost exclusively, and their properties are engineered to the physical limits.^4^ Still, much of the research efforts focus on fine-tuning these scaffolds and the development of novel fluorophore families gets little attention. Here, we aimed at the latter by synthesizing a new potentially fluorescent heteroaromatic polycycle inspired by the Green Fluorescent Protein (GFP) chromophore (GFPc).^4−6^ The GFPc is constrained within the β-barrel of the protein through hydrogen bonds, promoting intense fluorescence. However, its fluorescence is quenched by intramolecular rotations and isomerization when isolated. (Figure 1A).^7^ Many attempts were made for the restoration of the GFPc’s fluorescence,^8−13^ most notably the work of Wu and Burgess,^14^ (Figure 1B), however this structure resembles to BODIPYs, which also appears in the spectral characteristics, resulting in a small Stokes shift and strong green fluorescence. Others also used boron difluoride bridges to lock the conformation of GFP, revealing challenges such as hydrolytic stability issues, blueshifted absorption and harsh synthetic conditions.^15,16^ Recently, a new fluorescent polycyclic azaborine family was published containing a boron-hydroxide group instead of BF_2_ (Figure 1B). In addition to the facile synthesis from commercially available 2-formylphenylboronic acids, the reported fluorochromes had notable Stokes shifts and reasonable brightness. Unfortunately, they also had only UV excitation.^17−20^

**Figure 1:**
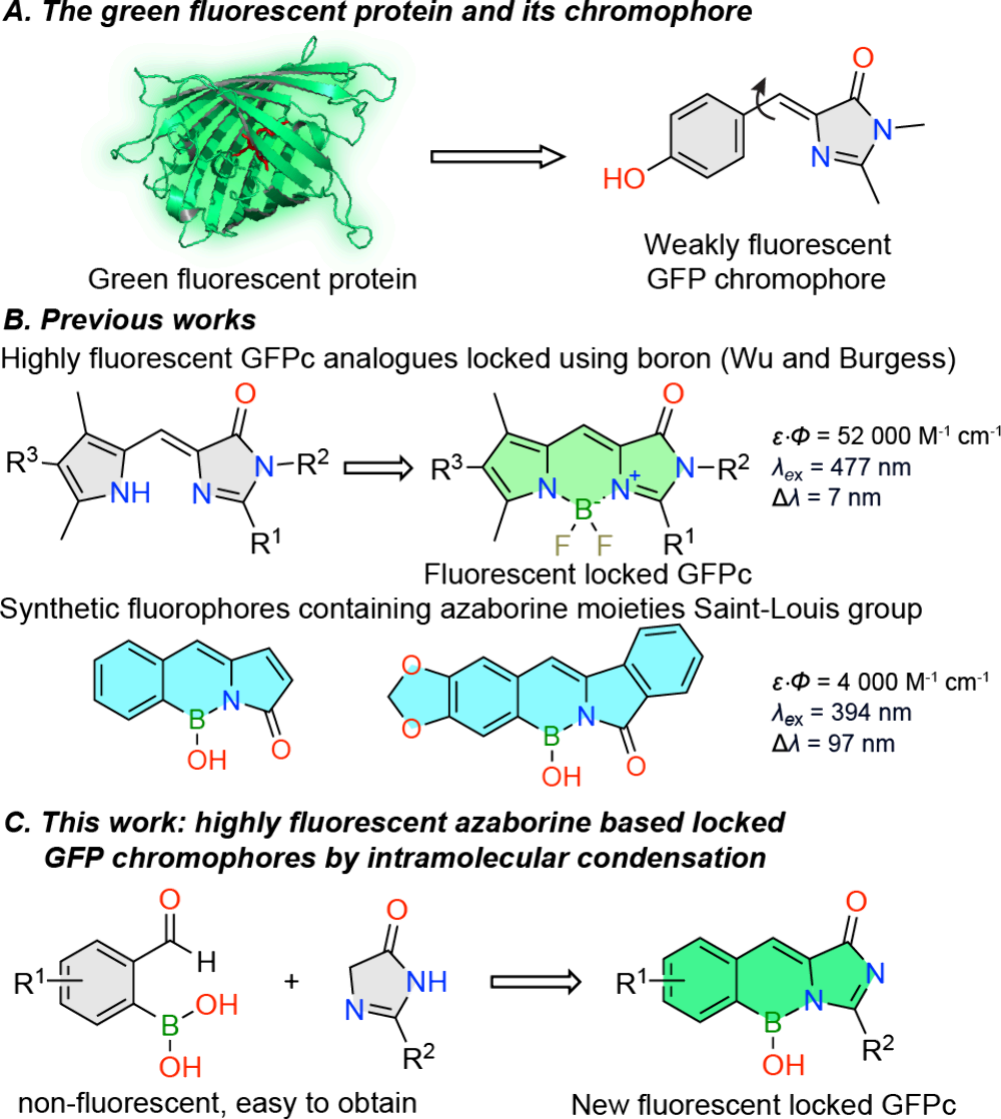
A. The GFP and its chromophore. B. Previous locked GFP chromophores and azaborines. C. The concept of this work.

We rationalized, that using these available 2-formylphenylboronic acids in a Knoevenagel condensation with 2*H*-imidazolinones we can obtain a new heterocyclic scaffold, which is a locked GFPc analogue (Figure 1C, Scheme 1). A single example of a similar reaction has been reported with hydantoin and 2-formylphenylboronic acid, but the fluorescence properties of their product were not studied.^21^ Our experiments showed mentionable fluorescence only in MeOH beside UV excitation (Figure S1).

**Scheme 1:**
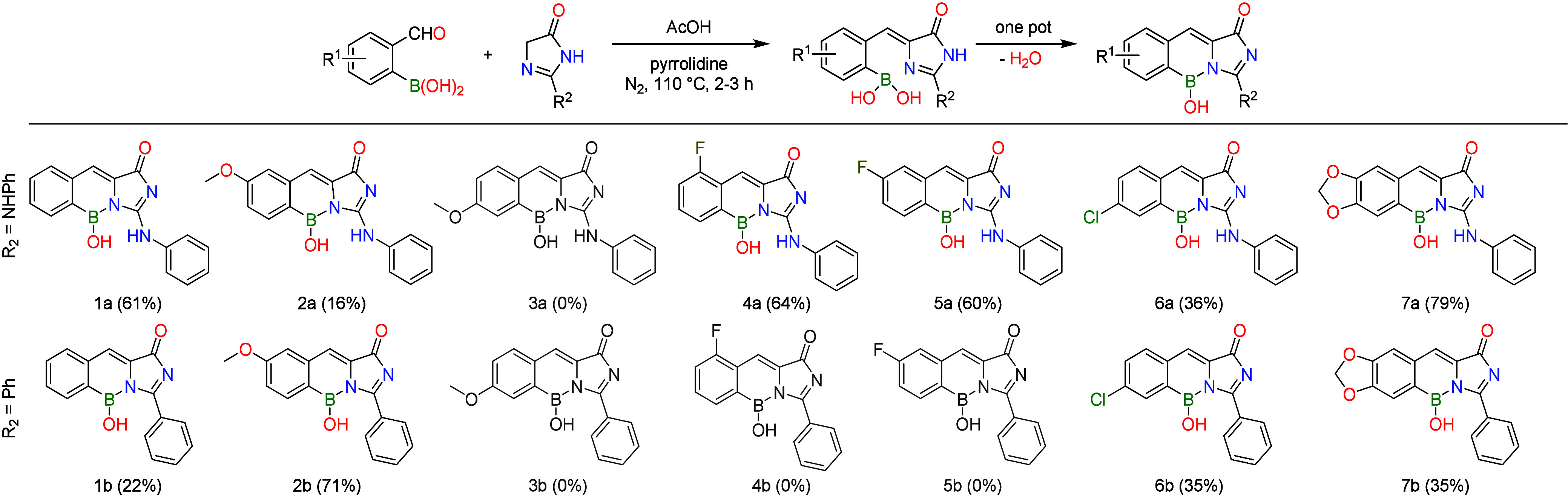
One-Pot Synthesis of Novel Fluorophores; Unsuccessful Reactions Are Grayed Out

In our work, the one-pot Knoevenagel condensation and ring closure were screened by using seven commercially available 2-formylphenylboronic acids. We employed two imidazolinones often used in other synthetic GFP chromophores with success as the reaction partner.^10,14,22^ In our procedure, an AcOH solution of the reactants and catalytic pyrrolidine was stirred at 110 °C for 2–3 h. The products were purified using preparative RP-HPLC, followed by filtration or lyophilization. In a few cases, the pure product could be filtered out as crystals from the reaction mixture. Ten out of the 14 products were successfully isolated (Scheme 1). No obvious correlation was observed between the yields and the substitution patterns of the substrates. In the case of methoxy derivative **3**, the formed intermediates suffered protodeboronation, perhaps before the ring closure could happen. In contrast, protodeboronation was only partially observed in the case of methoxy derivative **2a**, and not observed during the synthesis of **2b**. Attempts to obtain **3b**, or the fluoro derivatives **4b** and **5b**, yielded complex mixtures without traces of the expected products or intermediates. In the successful reactions, we always only detect traces of the intermediates, pointing to the rapidness of the ring closure. The isolated products did not show signs of decomposition during a year of storage at room temperature. Further functionalization of the B–OH group with alcohols was not successful. The photophysical characteristics of the prepared fluorophores were studied in different organic solvents (MeOH, DMSO, DCM) and aqueous buffers. The properties measured in DMSO are summarized in Table 1, while the rest is shown in Table S1 and Figure S1. The fluorescence of the products was investigated in both protonated and deprotonated forms. The protonation quenched the fluorescence in a few cases depending on the solvent and in other cases, it caused a slight increase and redshift in the emission (SI S1.3). Most derivatives did not have mentionable emission and the connection between the structure and photophysical characteristics were not always clear. However, the compounds substituted with a methylenedioxy group (**7a, 7b**) had excellent, red-shifted fluorescence compared to the other derivatives. In general, the phenyl derivatives (**1b**–**7b**) were also red-shifted and brighter compared to the phenylamino analogues (**1a**–**7a**). Unlike **1a**, compound **1b** showed remarkable fluorescence properties; while the best characteristics among all fluorophores were exhibited by **7b** (Figure 2A). Excitation by blue (425 nm) light yielded an intense yellow (525 nm) emission with brightness over 10 000 M^–1^ cm^–1^ and a remarkable 100 nm Stokes shift (0.56 eV; Figure 2B,C). Compared to previously published azaborines in the similar wavelength range, our fluorophore provided higher brightness.^17,18^ Photobleaching studies revealed that all the fluorophores had mediocre photostability, slightly superior compared to fluorescein. No notable solvatochromism was observed. The pH sensitivity of the fluorescence (**1b**, **6b**, **7b**) was also assessed in different pH buffers, revealing that the fluorophores are mainly in their deprotonated, fluorescent form at the physiological pH levels (pH > 7, Figure 3A). These properties, along with the straightforward synthesis of the compound make them an advantageous fluorophore family.

**Table 1:** Photophysical Properties of the Reported Probes, Absorption and Emission Maximum Intensities and Wavelengths, and Quantum Yields (Φ) in Dilute DMSO Solutions

	λ_abs_/nm	λ_ex_/nm	λ_em_/nm	ε_max_/M^–1^ cm^–1^	Φ
**1a**	365	375	412	38 300	0.03
**2a**	365	370	411	20 700	0.08
**4a**	368	375	408	26 000	0.30
**5a**	362	375	421	28 600	0.02
**6a**	370	375	408	23 300	0.43
**7a**	371	376	438	19 700	0.28
**1b**	412	422	499	15 900	0.41
**2b**	397	390	481	9 500	0.09
**6b**	397	408	478	10 700	0.11
**7b**	436	425	525	16 200	0.63
**8**	358	379	410	24 800	0.004
**8-Zn**	358	379	438	27 300	0.12
**9**	390	384	496	24 800	0.07
**9-Zn**	394	420	533	25 200	0.31

**Figure 2:**
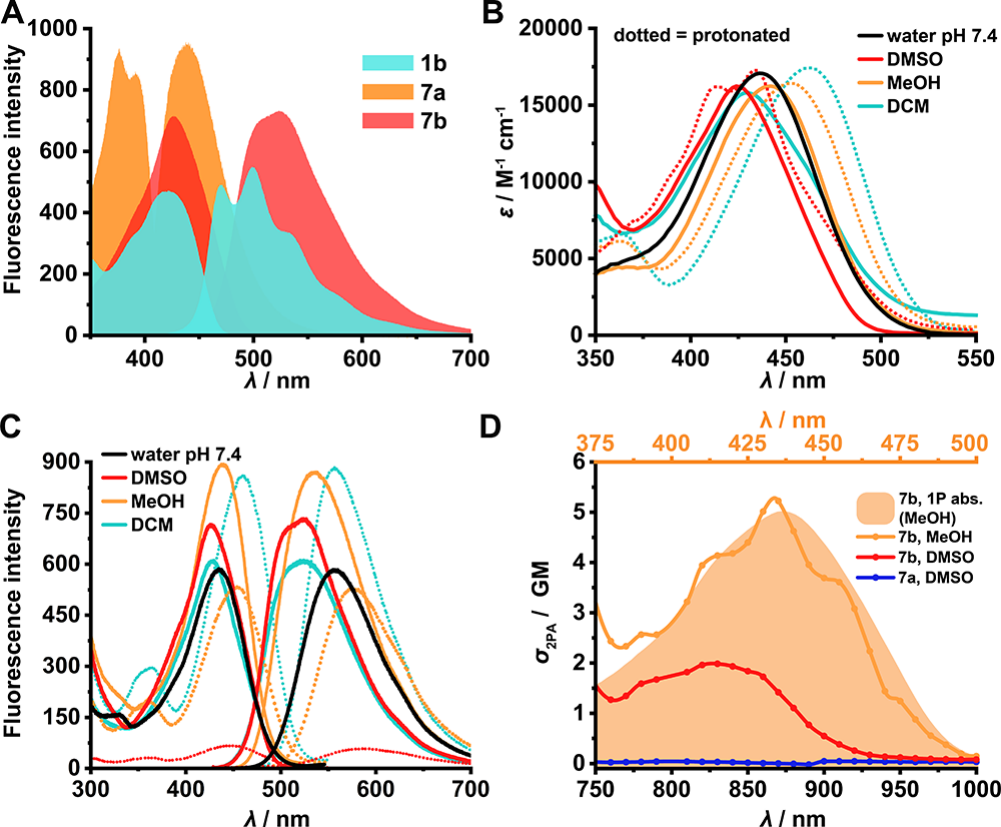
A. Excitation and emission spectra of selected compounds in DMSO. B. Absorption and C. fluorescence spectra of **7b** in various solvents. Excitation and emission spectra were normalized to 1 μM dye concentration, and dotted lines represent the spectra in the presence of TFA. D. 2P action cross section spectra of **7a** and **7b**. The overlay shows the 1P absorption of **7b**.

**Figure 3:**
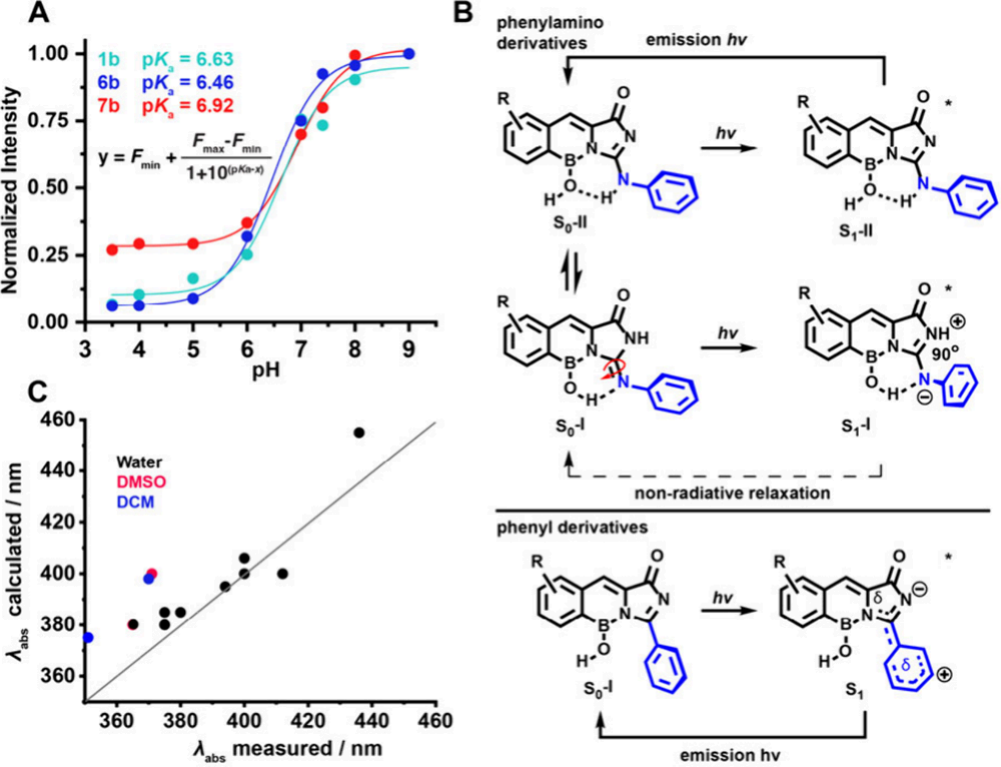
A. Fluorescence intensity of probes **1b**, **6b**, and **7b** in different pH aqueous HEPES buffers. p*K*_a_ values were determined using nonlinear regression. B. Computed mechanism of excitation and relaxation pathways of the fluorophores (B3LYP/6-31G(d,p)[PCM(water)]). C. Predicted absorption maxima against experimental values.

Two-photon (2P) microscopy is one of the most advanced branches of fluorescence microscopy, enabling fast, 3D imaging in deeper tissues with less photodamage.^23−25^ However, 2P fluorescence characteristics, which can be markedly different from one-photon (1P) excitation, have only been reported for a small fraction of all chemical dyes.^4,26,27^ Therefore, we characterized our best dyes (**1b**, **7b**) in 2P to promote their usage in 2P microscopy. The 2P excitation peak of **7b** was at 870 nm, corresponding to the double of the 1P absorption peak. The 2P action cross section of **7b** was 5.2 GM (Goeppert Mayer unit), validating that the dye is applicable for 2P imaging in the 800–950 nm excitation wavelength range. In subsequent experiments, we stained HEK-293 cells with **7b**, which proved to be membrane permeable and provided excellent 2P images of cell cultures (SI S1.4, Figure S6). We did not observe cytotoxicity, which makes this fluorophore even more promising.

To rationalize the spectroscopic differences, DFT computations were performed on phenylamino (**1a–7a**) and phenyl substituted scaffolds (**1b**–**7b**). First, the most stable tautomers and rotamers were scouted by comparing the computed enthalpies of the optimized structures. The calculated absorption and emission wavelengths were in good agreement with the experimental values (Figure 3C, SI S1.5). Theoretical calculations revealed that the phenylamino (**1a–7a**) derivatives exist in a tautomeric equilibrium, where one of the tautomers is nonfluorescent due to a quenching mechanism that involves a twisted intramolecular charge-transfer (TICT) state (Scheme S2). The presence of the equilibrium is supported by the broadening of the NMR peaks in certain cases(**1a**, **7a**). This tautomerism could explain the lower quantum yields of the phenylamino chromophores (**1a–7a**). In contrast, the phenyl derivatives (**1b–7b**) do not have stable tautomeric forms and are thus usually more emissive (Figure 3B, SI S1.5). The concept of systems chemistry was used to calculate the properties of rings and bonds of the new heterocycle.^28−31^ In the case of the phenylamino deriatives, the newly formed ring and the imidazolone ring shows slight aromaticity. The imide character moves from the imidazole N to the one adjacent to the phenyl ring. In the case of the phenyl derivatives, the newly formed ring is nonaromatic and the imidazolone ring gets slightly antiaromatic according to computations. The amidicity of the amide moiety in the compound also decreases significantly. (SI S1.5.3, Figure S11). Based on our recent experience with fluorescent Zn^2+^ sensors, we hypothesized that replacing the phenylamino group with a dipicolylamino binding motif would result in fluorogenic complexation of Zn^2+^ ions. In this sensor, the Zn^2+^ binding was expected to conformationally lock the molecule around the amino group, and also to disable the PET effect of the N atom by coordinatively binding to its lone electron pair (Figure 4A). Along this hypothesis, we have prepared two fluorogenic Zn^2+^ sensors based on the herein-reported fluorophore. The unsubstituted (**8**) and methylenedioxy (**9**) substituted analogues were obtained in good yields (69% and 50%, respectively) employing the same standard reaction procedure using the dipicolylamino substituted imidazolone. Both compounds retained similar spectral characteristics to the parent fluorophores (**1a**,**7a**) and showed a remarkable turn-on effect when Zn^2+^ was added to the solution containing the probes. The absorption spectra of the compounds remained unchanged upon Zn^2+^ addition (Figures 4B and S3A). The unsubstituted (**8**) probe had a smaller background fluorescence and therefore larger turn-on effect, but lower brightness in Zn-bound form (Δ*F*/*F* = 31 with a peak brightness of 3800 M^–1^ cm^–1^, Figure S3B). Whereas the methylenedioxy derivative (**9**) showed a higher brightness and a redshift caused by the Zn^2+^ binding (Δ*F*/*F* = 3.6 with a peak brightness of 7800 M^–1^ cm^–1^, Figure 4C). The selectivity of the probes was also tested. No turn-on was observed in the presence of relevant interfering ions, and the fluorescence in the presence of Zn^2+^ was disturbed only by Ni^2+^ (Figure 4D and S3C). The affinity of the probe for Zn^2+^ was determined by a complexometric titration for both probes (Figure 4E and S3E). Surprisingly, the reported probes have high *K*’_d_ values for Zn^2+^ in the μM–mM range, compared to other dipicolylamine derivatives, which usually turn on in the nM concentration range. Probe **9** had a stronger affinity with a *K*’_d_ of 380 μM, whereas in the case of **8** the binding is so weak, that the titration did not reach the end point before the solubility limit. We estimate its *K*’_d_ to be 7.4 mM. We speculate that hydrogen bonding exists between the acidic B–OH moiety and the N atom of dipicolylamine, which stabilizes the free compound resulting in a smaller energy gain of the Zn^2+^ binding (calculated 9.1 kJ mol^–1^). Notably, the methylenedioxy derivative (**9**) has about an order of magnitude stronger affinity toward Zn^2+^. This may be explained by the weaker acidity of the B–OH caused by the electron-donating effect of the methylenedioxy group resulting in weaker hydrogen bonding in the free state. These unusually low affinities illustrate how our new fluorophore scaffold can alter the properties of biological probes by previously unexploited intramolecular interactions. In this case, the dynamic range of the probe covers a concentration region previously scarcely populated by other sensors. The importance of this concentration region is crucial, as most other sensors detect lower Zn^2+^ levels, while the role of Zn^2+^ in neurotransmission can be compared to that of Ca^2+^. Zn^2+^ levels in the gray matter and neurons can be as high as 0.3 mM,^32,33^ exactly the working range of **9**. The photostability of **9** was excellent in a zinc-free solution, however, its zinc complex bleached in a similar extent to fluorescein. The 2P action cross section of probes **8** and **9** were determined (Figures 5A and S3D) both in their free form and in the presence of Zn^2+^.

**Figure 4:**
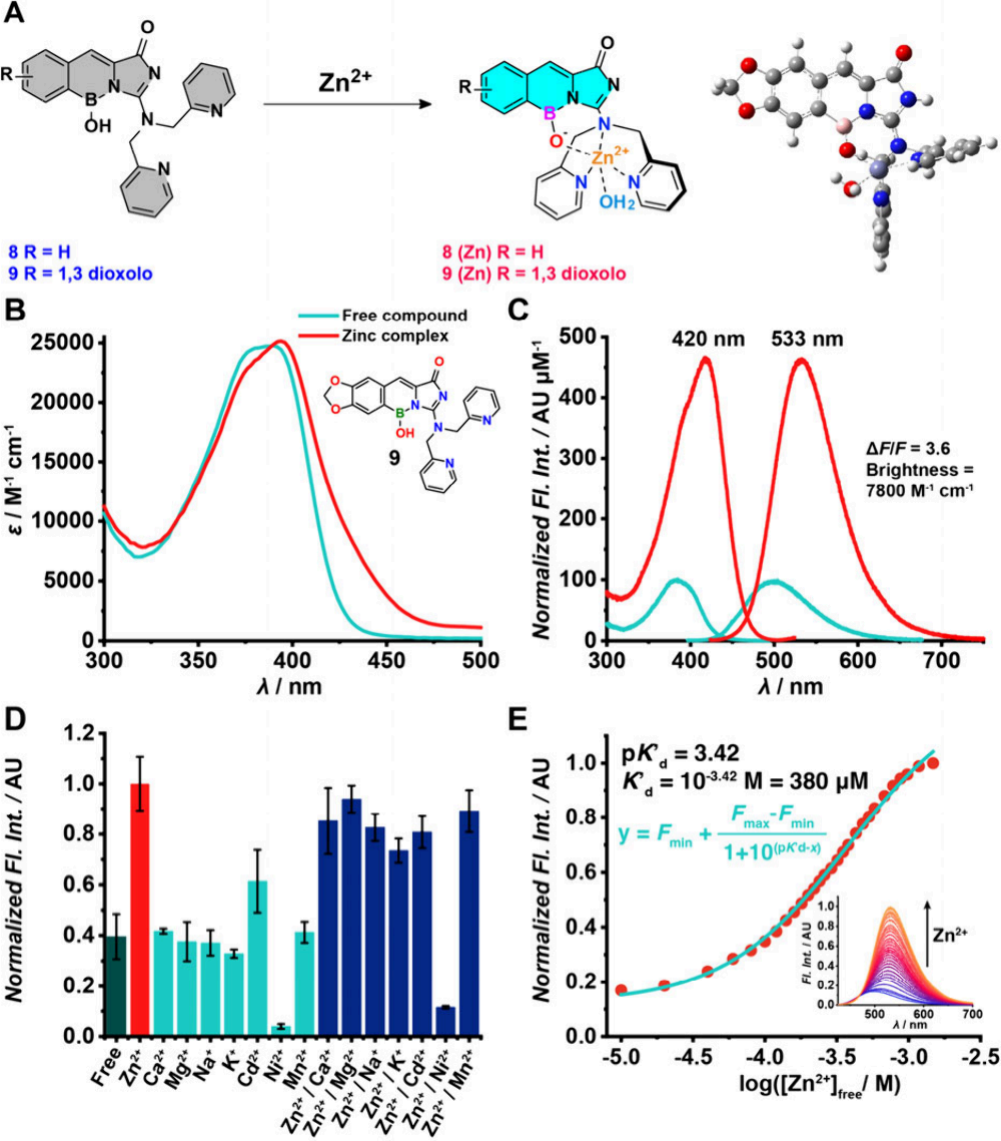
A. Structure of the prepared Zn^2+^ sensors (**8**, **9**) and their Zn^2+^ complexes confirmed by DFT studies (B3LYP/6-31G(d,p)[PCM(water)]). B. UV–vis and C. normalized fluorescence excitation and emission spectra of **9** in free (turquoise) and Zn^2+^-containing (red) solutions (pH = 7.4, HEPES). D. Selectivity of the reported Zn^2+^ sensor **9**. Normalized fluorescence intensity in the presence of Zn^2+^ (red), interfering ions (turquoise), and both simultaneously (blue). Error bars represent the standard deviation of triplicate measurements. E. Fluorometric titration of the reported sensors with Zn^2+^. *K*’_d_ was determined using nonlinear regression.

**Figure 5:**
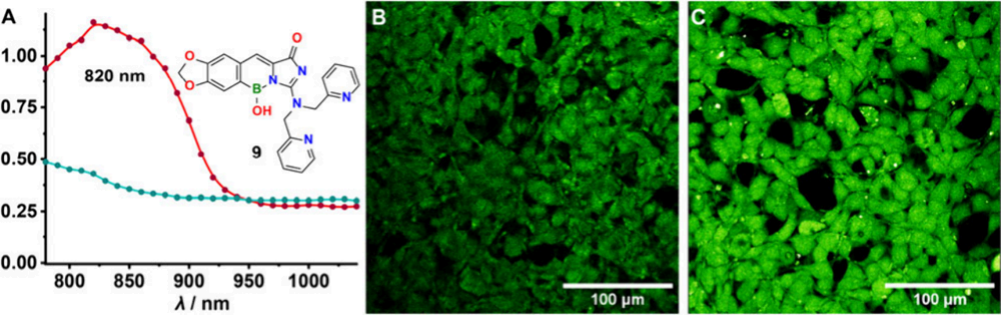
2P results. A. 2P action cross section spectra of **9** (100 μM) in zinc-free aqueous solution (turquoise) and zinc-containing solution (10 mM, red). B. 2P image of HEK-293 cell cultures stained with **9** (50 μM) without Zn^2+^ addition and C. in the presence of Zn^2+^ (1 mM).

While Zn^2+^ induced a notable increase in both cases, **9** had a much brighter fluorescence. This enabled us to showcase its biological potential by staining HEK-293 cells with it (Figure 5B). When cell-permeable Zn^2+^ (zinc pyrithione) was added during the staining a significant increase in the intracellular fluorescence intensity was observed (Figure 5C), suggesting, that compound **9** can be a good choice for detecting higher Zn^2+^ concentrations in biology using 2P microscopy. The addition of concentrated Zn^2+^ to the external imaging solution also had a similar effect, however, crystal formation was also observed due to precipitation of Zn(OH)_2_ at the pH of the imaging solution. TPEN, a stronger ionophore quenched the fluorescence confirming, that it was triggered by the free Zn^2+^ (Figure S7).

In summary, we synthesized and studied a novel boron-containing heterocycle analogous to the GFP chromophore with conformational restraints displaying remarkable fluorescent and 2P properties. The use of the fluorophore was demonstrated as a high millimolar Zn^2+^ sensor covering a useful concentration range.

## Data Availability

The data underlying this study are available in the published article and its Supporting Information. Raw 2PM images can be found at Mendeley Data in Data set S1:10.17632/pj2ph9jbj5.1.^34^ Additional raw data are available from the corresponding authors upon request.

## Supporting Information Available

Experimental procedures and characterization of all new compounds (PDF)
