# Supplementary material for: Introducing Rigidity into the GFP Chromophore via a Boron Bridge: Insights and Application in Two-Photon Imaging
Source: Org Lett. 2025 Mar 18;27(13):3128–33. doi: 10.1021/acs.orglett.5c00284 (PMC11976841; doi:10.1021/acs.orglett.5c00284)
Supplement: Supplementary file 1 — ol5c00284_si_001.pdf [file ol5c00284_si_001.pdf]

Supplementary information for

Introducing rigidity into the GFP chromophore via a boron bridge: insights and application in two-photon imaging.

Attila Csomos[a,b,#], Brigitta Petrilla[c,#], Levente Cseri[c], Gábor Turczel[d], Arnold Steckel[e], Anett Matuscsák[c], Gitta Schlosser[e], Balázs Rózsa[c,g,h,*], Ervin Kovács[g,i,*], Zoltán Mucsi[a,c,j,*]

[a]Femtonics Ltd., Asztalos Sándor út 9, H-1087 Budapest, Hungary

[b]ELTE Hevesy György PhD School of Chemistry, Pázmány Péter sétány 1/A, H-1117 Budapest, Hungary

[c]BrainVisionCenter, Liliom utca 43–45, H-1094 Budapest, Hungary

[d]NMR Research Laboratory, Centre for Structural Science, HUN-REN Research Centre for Natural Sciences, Magyar tudósok körútja 2, H-1117 Budapest, Hungary

[e]MTA-ELTE Lendület (Momentum) Ion Mobility Mass Spectrometry Research Group, ELTE Eötvös Loránd University, Faculty of Science, Institute of Chemistry, Pázmány Péter sétány 1/A, H-1117 Budapest, Hungary

[g]The Faculty of Information Technology, Pázmány Péter Catholic University, Práter utca 50, H-1083 Budapest, Hungary

[h]Laboratory of 3D Functional Network and Dendritic Imaging, HUN-REN Institute of Experimental Medicine, Szigony utca 43, H-1083 Budapest, Hungary

[i]Institute of Materials and Environmental Chemistry, HUN-REN Research Centre for Natural Sciences, Magyar tudósok körútja 2, H-1117 Budapest, Hungary

[j]Institute of Chemistry, Faculty of Materials Science and Engineering, University of Miskolc, H-3515 Miskolc, Hungary

#These authors contributed equally.

Table of Contents

S1. Detailed experimental procedures, synthetic notes and characterization of products 4

S1.1. Materials and methods 4

S1.2. General synthetic procedure and preparation of the reported derivatives 5

S1.2.1. Preparation of S1 5

S1.2.2. Preparation of S2 5

S1.2.3. Preparation of 1a 6

S1.2.4. Preparation of 2a 7

S1.2.5. Attempt on the preparation of 3a 8

S1.2.6. Preparation of 4a 8

S1.2.7. Preparation of 5a 9

S1.2.8. Preparation of 6a 9

S1.2.9. Preparation of 7a 10

S1.2.10. Preparation of 1b 11

S1.2.11. Preparation of 2b 11

S1.2.12. Attempt on the preparation of 3b 12

S1.2.13. Attempt on the preparation of 4b 12

S1.2.14. Attempt on the preparation of 5b 13

S1.2.15. Preparation of 6b 13

S1.2.16. Preparation of 7b 14

S1.2.17. Preparation of S3 14

S1.2.18. Preparation of 8 15

S1.2.19. Preparation of 9 16

S1.2.20. Preparation of S4 16

S1.2.21. Attempt on post-functionalization of the B-OH group with alcohols 17

S1.3. Spectroscopical characterization of the prepared fluorophores 17

S1.3.1. Determination of spectrophysical properties 17

S1.3.2. Spectrophysical data of all the reported compounds 20

S1.3.3. Raw spectra of the reported fluorophores 26

S1.4. Biological studies 30

S1.5. Computational studies 32

S1.5.1. Computational methods 32

S1.5.2. Theoretical results – photochemical mechanisms 32

S1.5.3. Theoretical results – systems chemistry analysis 42

S2. NMR and HRMS spectra of the prepared products ... 43
S2.1 NMR and HRMS spectra of 1a ... 43
S2.2 NMR and HRMS spectra of 2a ... 45
S2.3 NMR and HRMS spectra of 2c ... 46
S2.4 NMR and HRMS spectra of 3c ... 48
S2.5 NMR and HRMS spectra of 4a ... 49
S2.6 NMR and HRMS spectra of 5a ... 52
S2.7 NMR and HRMS spectra of 6a ... 56
S2.8 NMR and HRMS spectra of 7a ... 59
S2.9 NMR and HRMS spectra of 1b ... 61
S2.10 NMR and HRMS spectra of 2b ... 63
S2.11 NMR and HRMS spectra of 6b ... 65
S2.12 NMR and HRMS spectra of 7b ... 67
S2.13 NMR and HRMS spectra of 8 ... 70
S2.14 NMR and HRMS spectra of 9 ... 74
S2.15 NMR and HRMS spectra of S1 ... 78
S2.16 NMR and HRMS spectra of S2 ... 78
S2.17 NMR and HRMS spectra of S3 ... 79
S2.18 NMR and HRMS spectra of S4 ... 80
S3 Coordinates of the computationally optimized structures ... 81
S4 References ... 122

S1. Detailed experimental procedures, synthetic notes and characterization of products

S1.1. Materials and methods

All reagents, solvents and buffers were purchased from Merck, Fluorochem and TCI. NMR solvents were purchased from Eurisotop. Deionized water (DI) was prepared by a Milli-Q RiOs-DI-3UV system (>10 MΩ×cm). Solvents used for spectroscopy were UVASol brand, purchased from Supelco. The pH of the solutions was measured using a VWR pHenomenal pH 1100LB pH/mV/°C Meter with a 221 general purpose electrode.

For reaction monitoring and solubility determination a Shimadzu LC-40D XR UPLC-MS system was used equipped with a SIL-40C XR autosampler, SPD-M40 photodiode array detector, an RF-20A XS fluorescent detector and an LCMS-2020 DUIS Mass Spectrometer operated in alternating negative and positive modes. An Ascentis Express C18, 2 µm UHPLC column (L × I.D. 5 cm × 2.1 mm) was used at 50 °C provided by a CTO-40s column oven. Gradient elution was used with either using 0.1% v/v TFA in water (A) and MeCN (B) or 0.4 g L^{-1} NH_4HCO_3 in water (A) and MeCN (B). Preparative HPLC purifications were carried out on a Teledyne ACCQ Prep HP150 instrument using a Phenomenex Gemini C18, 250×50.00 mm; 10 µm, 110 Å column. The flow speed was set to 120 mL min^{-1} and the elution programs were usually around 30 minutes. NMR spectra were recorded on Varian Unity INOVA spectrometers operating at an equivalent ^1H frequency of 400, 500 and 600 MHz. Spectra were acquired at room temperature unless noted otherwise. ^{13}C spectra were acquired with ^1H decoupling. Notations for the ^1H NMR spectral splitting patterns include singlet (s), doublet (d), triplet (t), quartet (q), broad (br) and multiplet/overlapping peaks (m). Chemical shifts of the resonances are given as δ values in ppm and coupling constants (J) are expressed in Hz. Individual resonances were assigned based on 2D NMR experiments shown in this document. Exact mass measurements were performed on a high-resolution Thermo Fisher Scientific Q-Exactive Focus hybrid quadrupole-orbitrap mass spectrometer equipped with heated electrospray ionization source. Samples were dissolved in acetonitrile-water 1:1 (*V*/*V*) solvent mixture containing 0.1% v/v formic acid. Flow injection analysis was performed using a 50 µL/min eluent flow. Under the applied conditions, the compounds form protonated molecules, $[M + H]^+$.

Determination of the photophysical characteristics of the fluorophores was performed on a Shimadzu UV-1900i spectrophotometer and a Shimadzu RF-6000 spectrofluorometer (quartz cell, Hellma, pathlength: 1.0 cm).

Two-photon experiments were carried out using a single dual wavelength galvo-scanning microscope (SMART-2D, Femtonics). Femtosecond laser pulses were provided by a Chameleon Ultra II (Coherent) tunable laser. The intensity of the laser beam was controlled with an electro-optical modulator (Model 350-80 LA, Conoptics). The excitation was delivered to the sample, and the fluorescence signal was collected by an XLUMPlanFLN lens (Olympus, 20×, NA = 1.0) and then separated from the excitation light by a dichroic mirror (700dcxru, Chroma Technology). The fluorescence emission was split with a t570lpxr dichroic mirror (Chroma Technology) and filtered using FF01-527/70-25 (Semrock) and ET595/50m (Chroma Technology) bandpass filters for the green (492–562 nm) and red (570–620 nm) channels, respectively. The split fluorescence was delivered to GaAsP photomultiplier tubes fixed on the objective arm (H7422P-40-MOD, Hamamatsu). Images were processed using MES 6.5.8966 (Femtonics) and ImageJ 1.53t (NIH).

S1.2. General synthetic procedure and preparation of the reported derivatives

General procedure: to a stirred solution of 2-(phenylamino)-3,5-dihydro-4*H*-imidazol-4-one or 2-phenyl-3,5-dihydro-4*H*-imidazol-4-one (1 equiv., 0.3–1.0 mmol) in AcOH (5 mL $mmol^{-1}$), the (2-formylphenyl)boronic acid derivative (1 equiv., 0.3–1.0 mmol) and a catalytic amount of pyrrolidine (3% v/v) was added. The reaction mixture was stirred on a hotplate magnetic stirrer at 110 °C, under N_2 for 1-3 hours (until complete conversion, monitored by LC-MS). The solvent was evaporated in vacuo, then the mixture was dissolved in 10 mL of DMSO (THF, MeOH or MeCN were added if needed for dissolution). The crude product was purified by gradient elution preparative HPLC. Gradient elution was applied using 0.2% V/V TFA in water (A) and MeCN (B), or 0.1% m/m NH_4HCO_3 in water (A) and MeCN (B) as the mobile phase components. The pure fractions were collected, and MeCN was removed under vacuum. The residual aqueous solution was lyophilized to obtain the desired product.

S1.2.1. Preparation of S1

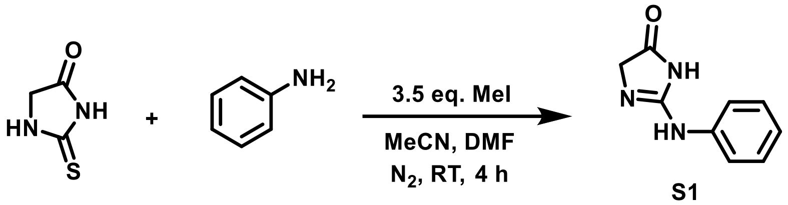

S1
3.05 g, 81%

2.5 g (21.5 mmol) 2-thiohydantoin was suspended in 30 ml MeCN in a dry round-bottom flask under N_2 atmosphere, and 1 mL of DMF was added to promote dissolution. The flask was sealed with rubber septum and 4.7 mL (75.3 mmol, 3.5 equiv.) MeI was added in one portion. The suspension was stirred overnight at 25 °C. The volatile components were drawn off in vacuo, and the solid residue was dissolved in 15 mL DMF under N_2 atmosphere, while the solution turned dark orange. 1.96 mL (21.5 mmol, 1 equiv.) aniline was added turning the color of the solution deeper. Reaction time was 4 hours. The solution was poured over a solution containing 4.3 g AcOK in 150 mL water. The product precipitated and was filtered to obtain a beige powder. The procedure was based on our previous work.[1]

S1: beige powder, 3.05 g, 81%

^{1}H NMR (400 MHz, DMSO-d_6) δ 10.11 – 9.71 (m, 1H), 7.57 (s, 1H), 7.46 (d, J = 8.0 Hz, 2H), 7.30 (t, J = 7.8 Hz, 2H), 7.05 (t, J = 7.4 Hz, 1H), 3.70 (s, 2H).

S1.2.2. Preparation of S2

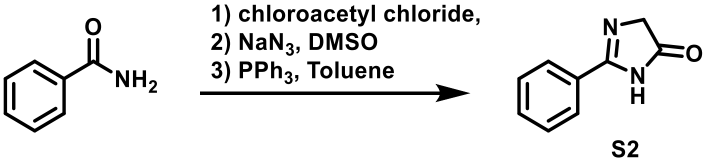

S2
529 mg, 32% total

Step 1: 2.5 g benzamide (20.6 mmol) and 1.7 mL (21.3 mmol, 1.03 equiv.) chloroacetic acid were mixed in a round bottom flask and heated to 110 °C for 30 minutes. The solids turned into a yellow solution after a few minutes, and after 30 minutes precipitation was observed again.

The volatiles were removed in vacuo and the precipitate was suspended in 20 mL Et_2O filtered and washed with 2×25 mL Et_2O. The filtrate was a white powder weighing 2.47 g (60.6% yield)

Step 2: 1.5 g of this product (7.6 mmol) was dissolved in 15 mL DMSO. Then, 0.99 g sodium azide (15.2 mmol, 2 equiv.) was added. The solution was stirred overnight at room temperature, then poured over 100 mL of ice-cold water. The product precipitated and was filtered and washed with 25 mL Et_2O. The product was a white solid, weighing 0.98 g (63.2% yield).

Step 3: 800 mg of this product (3.9 mmol) was dissolved in dry toluene in a previously dried round bottom flask. Triphenylphosphine (1.13 g, 4.3 mmol, 1.1 equiv.) was added and the system was flushed with nitrogen. The reaction was stirred overnight at room temperature, then the solvent was evaporated in vacuo. The solids were suspended in 25 mL Et_2O, filtered and washed with 2×25 mL Et_2O. The crude product was purified by flash chromatography on silica gel, using gradient elution: DCM to 10% MeOH in DCM over 10 CV. The preparation was based on the previously reported method.[2]

Product: pale red powder, 529 mg. 84% yield, 32% for the 3 steps.

^{1}H NMR (400 MHz, $CDCl_3$) δ 10.81 (s, 1H), 7.95 (d, J = 7.5 Hz, 2H), 7.72 – 7.42 (m, 5H), 4.36 (s, 2H).

S1.2.3. Preparation of 1a

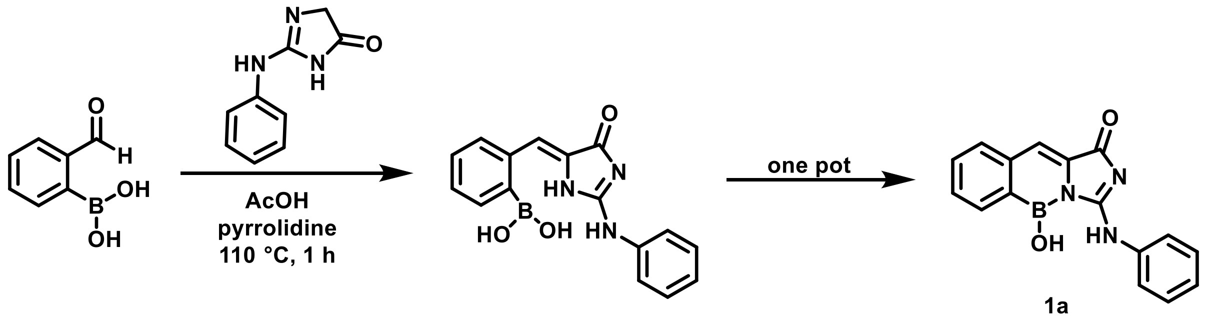

1a

175 mg, 61%

The general synthetic procedure has been used (see S1.2) starting from 150 mg (1 mmol) 2-formylphenylboronic acid and 2-(phenylamino)-3,5-dihydro-4*H*-imidazol-4-one. Reaction time was 1 hour. The product was purified using preparative HPLC, with the TFA-buffered eluent system. The product started to crystallize in the collected fractions. MeCN was removed under vacuum and the resulting aqueous suspension was cooled to 5 °C, then the product was filtered as white needle-like crystals.

1a: white needle crystals, 175 mg, 61% yield

^{1}H NMR (400 MHz, DMSO-d_6) δ 12.00 – 11.00 (m, 1H), 7.79 – 7.65 (m, 1H), 7.35 – 7.16 (m, 3H), 7.14 – 6.88 (m, 3H), 6.70 (d, J = 7.5 Hz, 2H), 6.59 (s, 1H).

^{13}C NMR (101 MHz, DMSO-d_6) δ 165.5, 151.9, 141.3, 137.8, 132.1, 129.2, 128.9, 128.5, 128.0, 127.6, 125.8, 110.5.

HRMS-ESI m/z $[M+H]^+$ calcd. for $C_{16}H_{13}BN_3O_2$ 290.1096 found 290.1088.

S1.2.4. Preparation of 2a

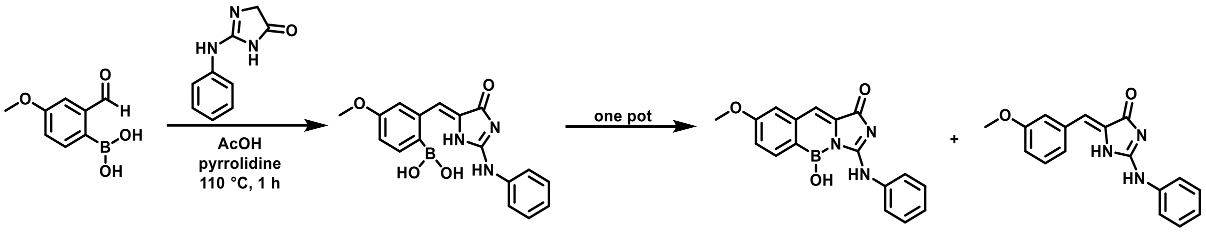

2a
53 mg, 16%

2c
63 mg, 22%

The general synthetic procedure has been used (see S1.2) starting from 186 mg (1 mmol) 4-methoxy-2-formylphenylboronic acid and 2-(phenylamino)-3,5-dihydro-4*H*-imidazol-4-one. The reaction time was 1 hour. Half of the conversion was towards protodeboronated product (2c), and half towards the expected product. After the evaporation of the solvent, the mixture was dissolved in DMSO, and the crude product was purified by prep. HPLC using TFA buffered eluent system. MeCN was removed in vacuum from the collected fractions, and the resulting aqueous suspensions were cooled to 5 °C, then the products were filtered.

2a: yellowish white powder, 53 mg, 16%

^{1}H NMR (500 MHz, CD_3OD) δ 8.45 – 8.39 (m, 2H), 7.66 (td, J = 7.5, 1.9 Hz, 1H), 7.57 – 7.52 (m, 2H), 7.49 (d, J = 8.2 Hz, 1H), 7.42 (s, 1H), 7.08 (d, J = 2.5 Hz, 1H), 7.01 (dd, J = 8.2, 2.5 Hz, 1H), 3.84 (s, 3H).

The ^{13}C NMR peaks of the product were broadened by chemical exchange and could not be observed directly. Therefore, HSQC and HMBC measurements were used to determine the ^{13}C chemical shifts.

^{13}C NMR (126 MHz, CD_3OD) δ 170.1, 168.3, 159.1, 141.8, 136.5, 133.6, 133.0, 132.7, 130.9, 128.4, 127.7, 126.7, 117.6, 114.4.

HRMS-ESI *m/z* $[M+H]^+$ calcd. for $C_{17}H_{15}O_3N_3B$ 320.1201 found 320.1196.

^{13}C NMR could not be recorded due to the signal broadening in the spectrum. ^{13}C chemical shifts were determined based on 2D spectra.

2c: white powder, 63 mg, 22% yield

The NMR spectrum of the product is in good agreement with the literature.[3]

^{1}H NMR (500 MHz, DMSO-*d6*) δ 10.67 (s, 1H), 9.82 (s, 1H), 8.10 (s, 1H), 7.85 (d, J = 7.8 Hz, 2H), 7.42 (d, J = 7.6 Hz, 1H), 7.34 (dd, J = 8.6, 7.4 Hz, 2H), 7.29 (t, J = 7.9 Hz, 1H), 7.08 (tt, J = 7.3, 1.1 Hz, 1H), 6.86 (ddd, J = 8.3, 2.7, 1.0 Hz, 1H), 6.50 (s, 1H), 3.85 (s, 3H).

^{13}C NMR (126 MHz, DMSO-*d6*) δ 170.2, 159.2, 155.1, 140.8, 138.7, 136.8, 129.3, 128.7, 123.3, 122.9, 119.4, 115.1, 114.4, 113.5, 54.8.

HRMS-ESI *m/z* $[M+H]^+$ calcd. for $C_{17}H_{16}N_3O_2$ 294.1237 found 294.1231.

S1.2.5. Attempt on the preparation of 3a

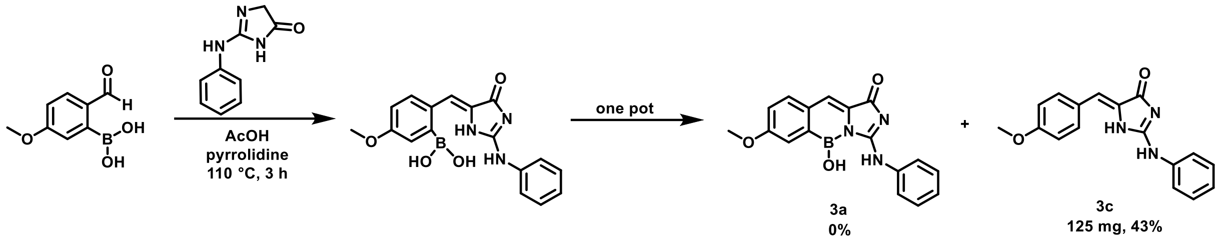

The general synthetic procedure has been used (see S1.2) starting from 180 mg (1 mmol) aldehyde 5-methoxy-2-formylphenylboronic acid and 2-(phenylamino)-3,5-dihydro-4*H*-imidazol-4-one. After the starting materials were dissolved, the reaction mixture turned slightly orange, then a white precipitate was observed. The reaction yielded only the protodeboronated product (3c) after 3 hours. The precipitate was filtered and washed with EtOH, yielding a beige powder.

3c: beige powder, 125 mg, 43% yield.

The MS, NMR and spectroscopic characteristics of 3c are in good agreement with the ones previously reported in the literature.[1]

^{1}H NMR (600 MHz, DMSO-d_6) δ 10.57 (s, 1H), 9.70 (s, 1H), 8.09 – 8.05 (m, 2H), 7.79 (d, J = 7.9 Hz, 2H), 7.42 – 7.36 (m, 2H), 7.07 (tt, J = 7.3, 1.1 Hz, 1H), 7.02 – 6.98 (m, 2H), 6.50 (s, 1H), 3.80 (s, 3H).

^{13}C NMR (151 MHz, DMSO-d_6) δ 170.3, 159.2, 154.3, 138.9, 138.7, 131.9, 128.9, 128.1, 122.7, 119.2, 115.1, 114.1, 55.2.

HRMS-ESI m/z $[M+H]^+$ calcd. for $C_{17}H_{16}N_3O_2$ 294.1237 found 294.1231.

S1.2.6. Preparation of 4a

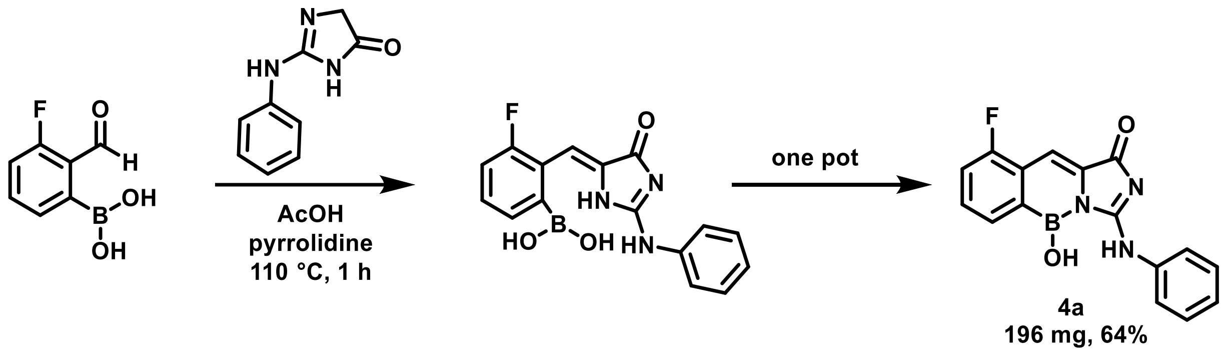

The general synthetic procedure has been used (see S1.2) starting from 168 mg (1 mmol) 3-fluoro-2-formylphenylboronic acid and 2-(phenylamino)-3,5-dihydro-4*H*-imidazol-4-one. Reaction time was 1 hour. The collected prep. HPLC fractions started to crystallize. MeCN was removed under vacuum and the resulting aqueous suspension was cooled to 5 °C, then the product was filtered off as white crystals.

4a: white crystals, 196 mg, 64% yield

^{1}H NMR (400 MHz, DMSO-$d6$) δ 7.47 (d, J = 7.3 Hz, 1H), 7.21 (td, J = 7.6, 5.1 Hz, 1H), 7.07 (d, J = 7.4 Hz, 3H), 6.95 (dd, J = 10.8, 8.1 Hz, 1H), 6.78 (d, J = 6.2 Hz, 2H), 6.55 (s, 1H).

^{13}C NMR (101 MHz, DMSO-*d6*) δ 164.8, 159.1 (d, J = 250.7 Hz), 152.8, 142.9, 139.9, 129.3 (d, J = 7.7 Hz), 129.0, 128.4, 127.8 (d, J = 3.3 Hz), 126.6, 126.5, 124.8 (d, J = 10.7 Hz), 113.5(d, J = 21.1 Hz), 101.8 (d, J = 7.0 Hz).

HRMS-ESI *m/z* $[M+H]^+$ calcd. for $C_{16}H_{12}BFN_3O_2$ 308.1001 found 308.0997.

S1.2.7. Preparation of 5a

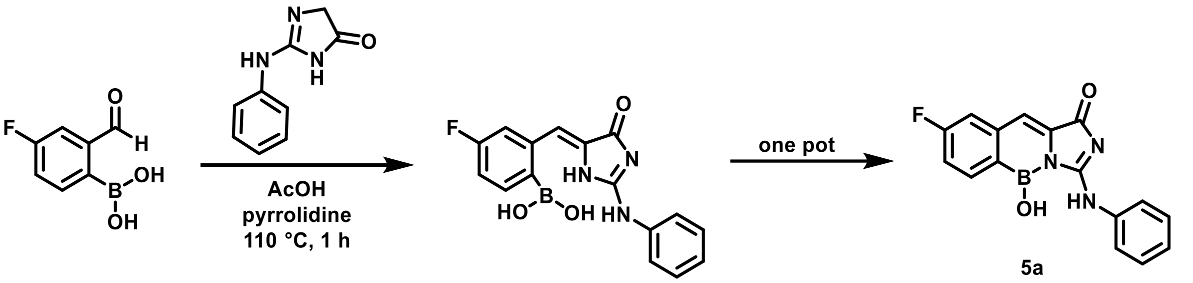

5a
185 mg, 60%

The general synthetic procedure has been used (see Chapter S1.2) starting from 168 mg (1 mmol) 4-fluoro-2-formylphenylboronic acid and 2-(phenylamino)-3,5-dihydro-4*H*-imidazol-4-one. Reaction time was 1 hour. The collected prep. HPLC fractions started to crystallize. MeCN was removed under vacuum and the resulting aqueous suspension was cooled to 5 °C, then the product was filtered off as white crystals.

5a: white crystals, 185 mg, 60% yield

^1H NMR (500 MHz, DMSO-d_6) δ 11.54 (s, 1H), 7.72 (t, J = 7.4 Hz, 1H), 7.15 – 7.07 (m, 3H), 7.08 – 6.98 (m, 2H), 6.74 (d, J = 7.6 Hz, 2H), 6.59 (s, 1H).

^{13}C NMR (126 MHz, DMSO-d_6) δ 164.9, 162.8, 160.9, 151.9, 140.8, 138.9, 134.0, 129.1, 128.5, 126.0, 125.9, 114.3, 114.2, 109.6

HRMS-ESI *m/z* $[M+H]^+$ calcd. for $C_{16}H_{12}BFN_3O_2$ 308.1001 found 308.0997.

S1.2.8. Preparation of 6a

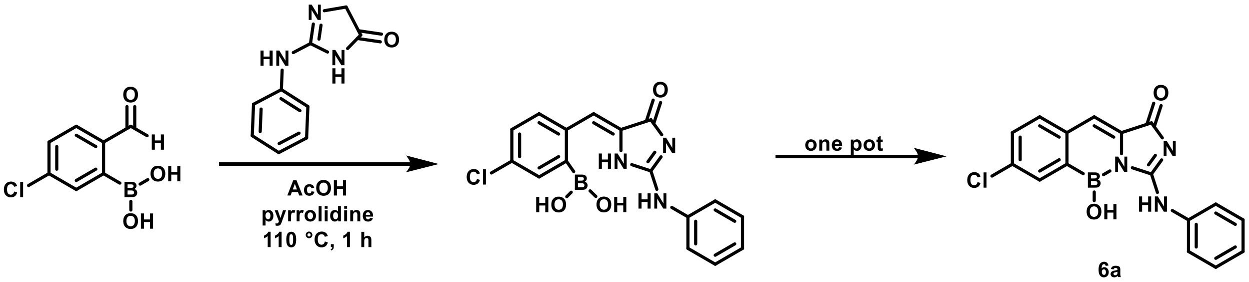

6a
115 mg, 36%

The general synthetic procedure has been used (see S1.2) starting from 184 mg (1 mmol) 5-chloro-2-formylphenylboronic acid and 2-(phenylamino)-3,5-dihydro-4*H*-imidazol-4-one. Reaction time was 1 hour. After the starting materials were dissolved, the reaction mixture turned slightly orange, then a precipitate formed in 1 hour, which was filtered off and washed with EtOH.

6a: slightly pink crystals, 115 mg, 36% yield

^{1}H NMR (400 MHz, DMSO-*d6*) *δ* 7.57 (d, *J* = 2.2 Hz, 1H), 7.21 (d, *J* = 8.4 Hz, 1H), 7.17 (dd, *J* = 8.3, 2.2 Hz, 1H), 7.09 (t, *J* = 7.3 Hz, 2H), 7.04 (d, *J* = 7.1 Hz, 1H), 6.79 (d, *J* = 7.0 Hz, 2H), 6.59 (s, 1H).

^{13}C NMR (101 MHz, DMSO-*d6*) *δ* 165.0, 152.7, 142.7, 140.1, 136.2, 132.5, 131.3, 130.6, 128.6, 128.5, 127.2, 126.5, 126.4, 110.5

HRMS-ESI *m/z* $[M+H]^+$ calcd. for $C_{16}H_{12}BClN_3O_2$ 324.0706 found 324.0699.

S1.2.9. Preparation of 7a

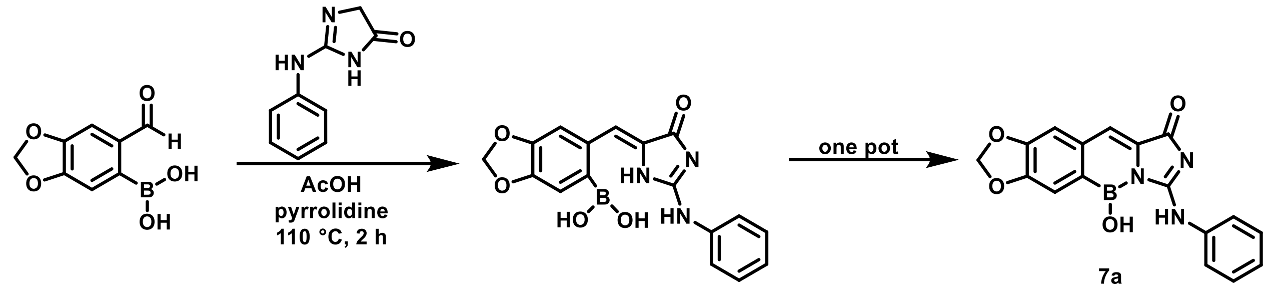

The general synthetic procedure has been used (see S1.2) starting from 150 mg (0.77 mmol) 2-formyl-4,5-methylenedioxyphenylboronic acid and 2-(phenylamino)-3,5-dihydro-4*H*-imidazol-4-one. The reaction mixture turned orange after a few minutes, and after 2 hours conversion was complete according to LC-MS. The solution was cooled in ice-water bath and a white powder precipitated, which was filtered yielding a white pure product. The mother liquor still contained a significant amount of product, therefore it was purified by prep. HPLC in TFA eluent. MeCN was removed from the fractions under vacuum. The product crystallized from this aqueous medium as yellow precipitate, which was isolated after lyophilisation.

7a: yellow powder, 203 mg, 79%

^{1}H and ^{13}C NMR spectra contain multiple set of peaks due to the possibility of tautomerism. HRMS and HPLC-UV studies are shown to confirm the purity of the compound.

^{1}H NMR (400 MHz, DMSO-*d6*) *δ* 11.70 (s, 1H), 11.40 (s, 1H), 10.97 (s, 1H), 9.90 (s, 1H), 8.80 (s, 1H), 7.99 – 7.68 (m, 1H), 7.56 – 7.31 (m, 4H), 7.29 – 6.99 (m, 9H), 6.96 (s, 1H), 6.69 (d, *J* = 7.7 Hz, 3H), 6.59 (s, 1H), 6.17 (s, 2H), 6.01 (d, *J* = 11.5 Hz, 3H).

^{13}C NMR (101 MHz, DMSO-*d6*) *δ* 151.4, 148.3, 147.9, 145.6, 142.7, 138.9, 138.8, 138.0, 137.2, 133.6, 129.6, 129.1, 128.0, 127.6, 125.7, 125.4, 123.1, 121.4, 111.7, 110.4, 110.2, 109.7, 108.9, 108.3, 102.2, 101.3

HRMS-ESI *m/z* $[M+H]^+$ calcd. for $C_{17}H_{13}BN_3O_4$ 334.0994 found 334.0987.

S1.2.10. Preparation of 1b

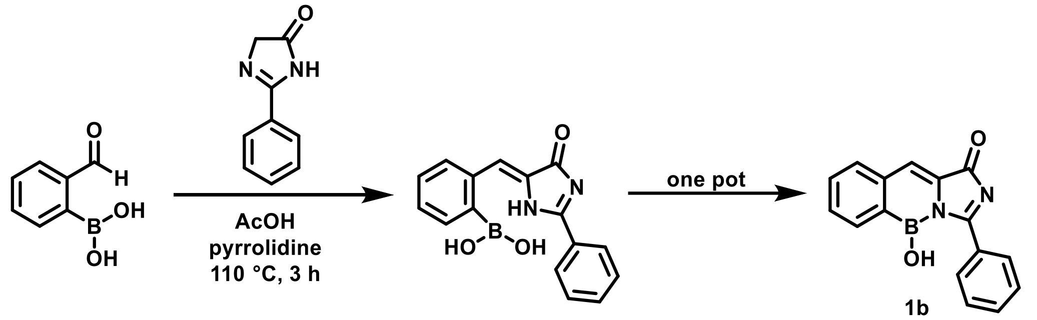

1b
38 mg, 22%

The general synthetic procedure has been used (see S1.2) starting from 94 mg (0.62 mmol) 2-formylphenylboronic acid and phenylimidazolone. Reaction time was 3 hours. The crude product was purified by prep. HPLC in TFA eluent. MeCN was removed from the fractions under vacuum, then the aqueous suspension was lyophilized, resulting in an orange powder. The product was not pure according to LC-MS, therefore it was purified again by prep. HPLC in NH_4HCO_3 eluent. MeCN was removed from the fractions under vacuum, then the aqueous suspension was lyophilized, resulting in a yellow powder.

1b: yellow powder, 38 mg, 22% yield

^{1}H NMR (600 MHz, CD_3OD) δ 8.43 – 8.38 (m, 2H), 7.74 – 7.68 (m, 1H), 7.60 – 7.56 (m, 3H), 7.55 (s, 1H), 7.54 (d, J = 7.4 Hz, 1H), 7.44 (td, J = 7.3, 1.1 Hz, 1H), 7.33 (td, J = 7.5, 1.4 Hz, 1H).

^{13}C NMR (151 MHz, CD_3OD) δ 167.6, 166.2, 151.4, 136.3, 134.8, 133.4, 133.2, 132.7, 132.5, 132.3, 131.5, 129.6, 128.5, 126.4.

HRMS-ESI m/z $[M+H]^+$ calcd. for $C_{16}H_{12}BN_2O_2$ 275.0987 found 275.0981.

S1.2.11. Preparation of 2b

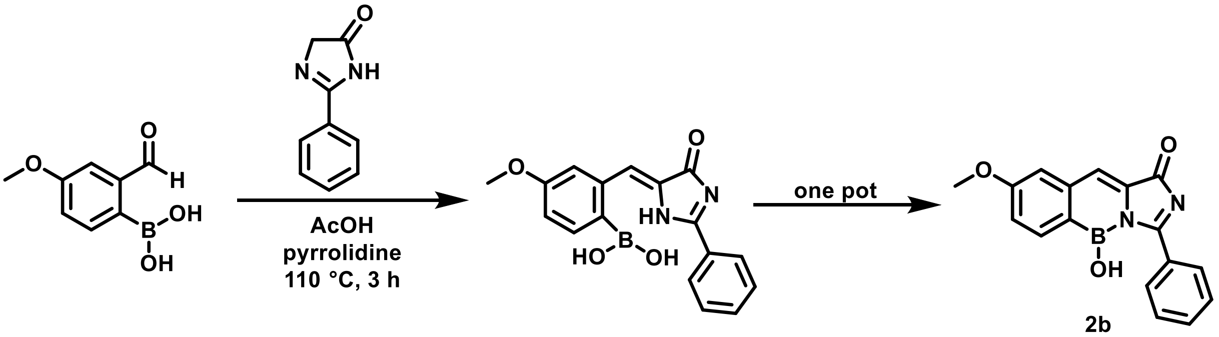

2b
67 mg, 71%

The general synthetic procedure has been used (see S1.2) starting from 58 mg (0.31 mmol) aldehyde 4-methoxy-2-formylphenylboronic acid and phenylimidazolone. The reaction time was 3 hours. The crude product was purified by prep. HPLC according to the general procedure first in TFA containing eluent then again in NH_4HCO_3 containing eluent to eliminate all impurities. MeCN was removed from the fractions under vacuum, then the aqueous suspension was lyophilized, resulting a cotton-like yellow powder.

2b: cotton-like yellow powder, 67 mg, 71%.

^{1}H NMR (500 MHz, CD_3OD) δ 8.45 – 8.39 (m, 2H), 7.66 (td, J = 7.5, 1.9 Hz, 1H), 7.57 – 7.52 (m, 2H), 7.49 (d, J = 8.2 Hz, 1H), 7.42 (s, 1H), 7.08 (d, J = 2.5 Hz, 1H), 7.01 (dd, J = 8.2, 2.5 Hz, 1H), 3.84 (s, 3H).

The ^{13}C NMR peaks of the product were broadened by chemical exchange and could not be observed directly. Therefore, HSQC and HMBC measurements were used to determine the ^{13}C chemical shifts.

^{13}C NMR (126 MHz, CD_3OD) δ 170.1, 168.3, 159.1, 141.8, 136.5, 133.6, 133.0, 132.7, 130.91, 128.4, 127.8, 126.8, 117.6, 114.5, 55.3.

HRMS-ESI m/z $[M+H]^+$ calcd. for $C_{17}H_{14}BN_2O_3$ 305.1092 found 305.1087.

S1.2.12. Attempt on the preparation of 3b

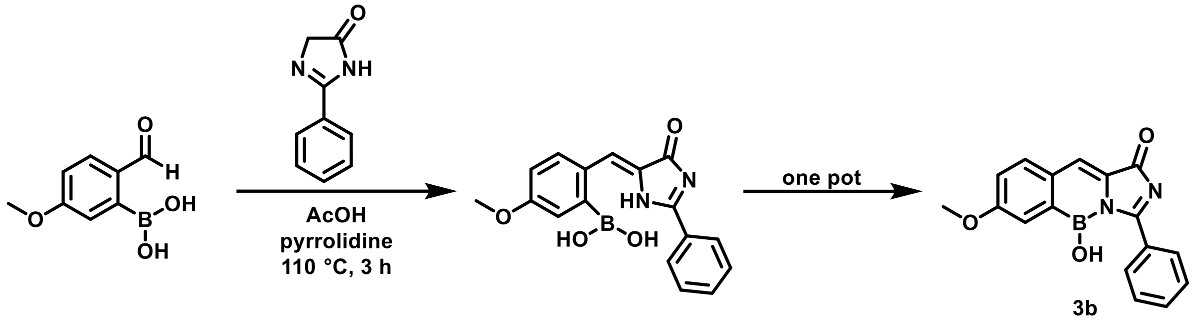

3b
0%

The general synthetic procedure has been used (see S1.2) starting from 56 mg (0.31 mmol) 5-methoxy-2-formylphenylboronic acid and phenylimidazolone. The reaction mixture decomposed in 3 hours without the detectable formation of the desired product.

S1.2.13. Attempt on the preparation of 4b

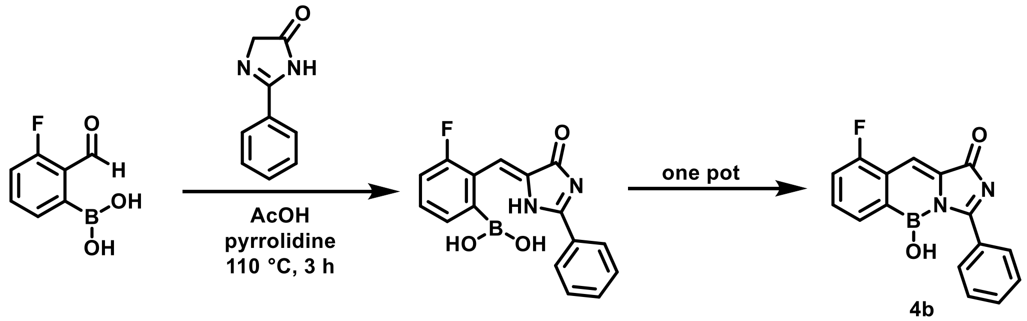

4b
0%

The general synthetic procedure has been used (see S1.2) starting from 52 mg (0.31 mmol) 3-fluoro-2-formylphenylboronic acid and phenylimidazolone. Only the formation of the Knoevenagel adduct was detected and decomposition was observed after 3 hours.

S1.2.14. Attempt on the preparation of 5b

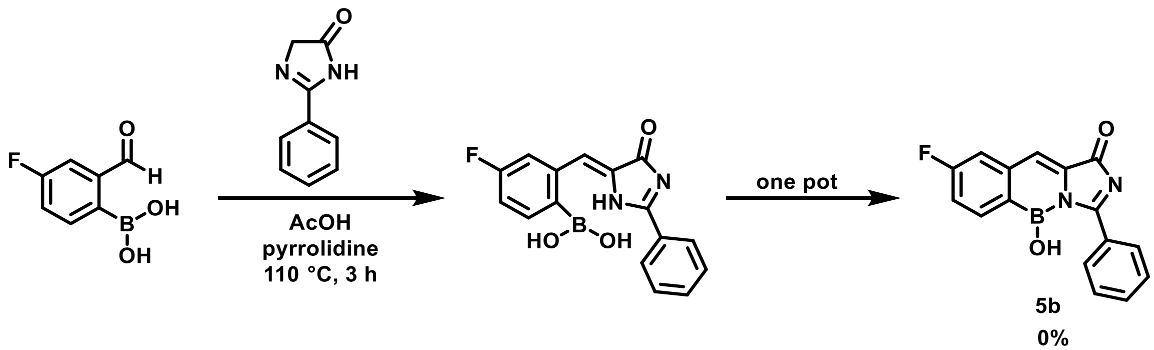

5b
0%

The general synthetic procedure has been used (see S1.2) starting from 52 mg (0.31 mmol) 4-fluoro-2-formylphenylboronic acid and phenylimidazolone. Only the formation of the Knoevenagel adduct and decomposition were observed after 3 hours (HPLC-MS). As the expected product did not form, and the reaction mixture was complex, the Knoevenagel intermediate was not isolated.

S1.2.15. Preparation of 6b

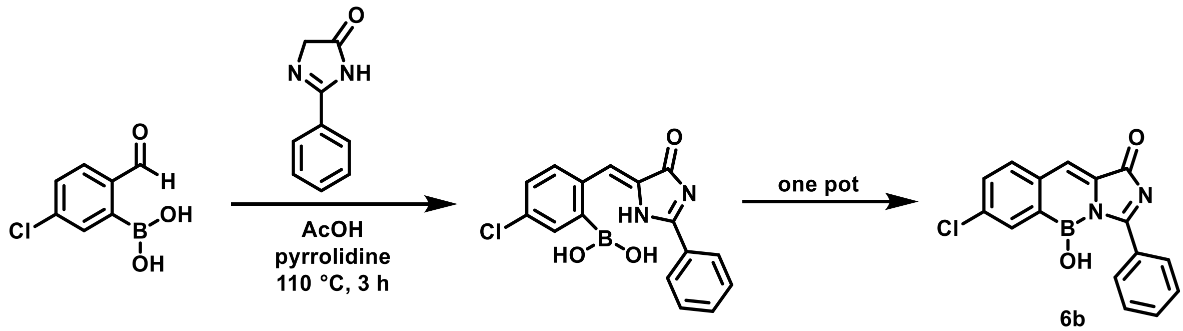

6b
20 mg, 35%

The general synthetic procedure has been used (see S1.2) starting from 58 mg (0.31 mmol) 5-chloro-2-formylphenylboronic acid and phenylimidazolone. The reaction time was 3 hours. The crude product was purified by prep. HPLC according to the general procedure first in TFA containing eluent then again in NH_4HCO_3 containing eluent to eliminate all impurities. MeCN was removed from the fractions under vacuum, then the aqueous suspension was lyophilized, resulting a yellow powder.

6b: yellow powder, 20 mg, 35%.

^{1}H NMR (500 MHz, CD_3OD) δ 8.45 – 8.39 (m, 2H), 7.65 (td, J = 7.5, 1.8 Hz, 1H), 7.57 – 7.52 (m, 3H), 7.48 (d, J = 8.1 Hz, 1H), 7.40 (s, 1H), 7.29 (dd, J = 8.1, 2.3 Hz, 1H)

The ^{13}C NMR spectrum of the product was broad and insensitive, therefore, HSQC and HMBC measurements were used to determine the ^{13}C chemical shifts.

^{13}C NMR (126 MHz, CD_3OD) δ 171.1, 169.8, 153.0, 137.1, 133.4, 131.0, 127.9, 127.1, 126.1.

HRMS-ESI m/z $[M+H]^+$ calcd. for $C_{16}H_{11}BClN_2O_2$ 309.0597 found 309.0590.

S1.2.16. Preparation of 7b

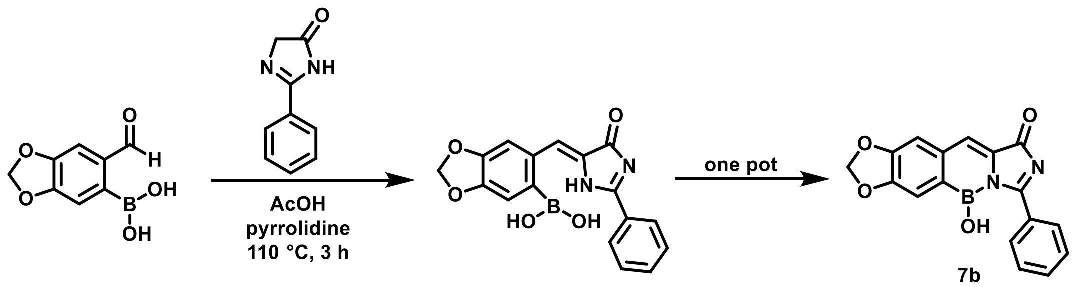

7b
70 mg, 35%

The general synthetic procedure has been used (see S1.2) starting from 121 mg (0.62 mmol) 2-formyl-4,5-methylenedioxyphenylboronic acid and phenylimidazolone. Reaction time was 3 hours. The crude product was purified by prep. HPLC in TFA eluent. MeCN was removed from the fractions under vacuum, then the aqueous suspension was lyophilized, resulting a cotton-like yellow powder.

7b: yellow powder, 70 mg, 35% yield

^{1}H NMR (600 MHz, CD_3OD) δ 8.54 (dd, 2H), 7.79 (t, J = 7.5 Hz, 1H), 7.68 (t, J = 7.7 Hz, 2H), 7.53 (s, 1H), 7.19 (s, 1H), 7.16 (s, 1H), 6.14 (s, 2H).

^{13}C NMR (151 MHz, CD_3OD) δ 153.0, 149.0, 134.4, 133.0, 132.5, 131.1, 130.6, 129.6, 128.2, 113.1, 111.4, 102.9.

HRMS-ESI m/z $[M+H]^+$ calcd. for $C_{17}H_{12}BN_2O_4$ 319.0885 found 319.0880.

S1.2.17. Preparation of S3

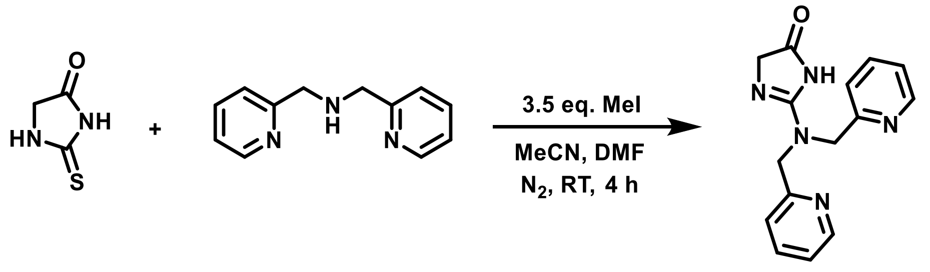

S3
352 mg, 22%

650 mg (5.6 mmol) 2-thiohydantoin was suspended in 8 mL MeCN in a dry round-bottom flask under N_2 atmosphere, and a few drops of DMF was added to promote dissolution. The flask was sealed with rubber septum and 1.2 mL (19.6 mmol, 3.5 equiv.). MeI was added in one portion. The suspension was stirred overnight at 25 °C. The volatile components were drawn off in vacuo, and the solid residue was dissolved in 5 mL DMF under N_2 atmosphere, while the solution turned dark orange. 1.1 mL (6.2 mmol, 1.1 equiv.) bis(2-pyridylmethyl)amine was added turning the color of the solution deeper. Reaction time was 4 hours. The crude product was purified by prep. HPLC in NH_4HCO_3 eluent system. MeCN was removed from the fractions under vacuum, then the aqueous suspension was lyophilized, resulting a viscose brown liquid.

S3: brown viscous liquid, 352 mg, 22% yield

^{1}H NMR (400 MHz, DMSO-*d6*) δ 8.53 (s, 2H), 8.36 (s, 1H), 7.78 (t, *J* = 7.7 Hz, 2H), 7.32 (s, 2H), 7.30 (s, 2H), 4.75 (d, *J* = 27.3 Hz, 4H), 3.85 (s, 2H).

^{13}C NMR (101 MHz, DMSO-*d6*) δ 185.4, 171.9, 156.4, 149.2, 136.9, 122.6, 121.8, 53.5, 51.7, 50.8

HRMS-ESI *m/z* $[M+H]^+$ calcd. for $C_{15}H_{16}ON_5$ 282.1350 found 282.1343.

S1.2.18. Preparation of 8

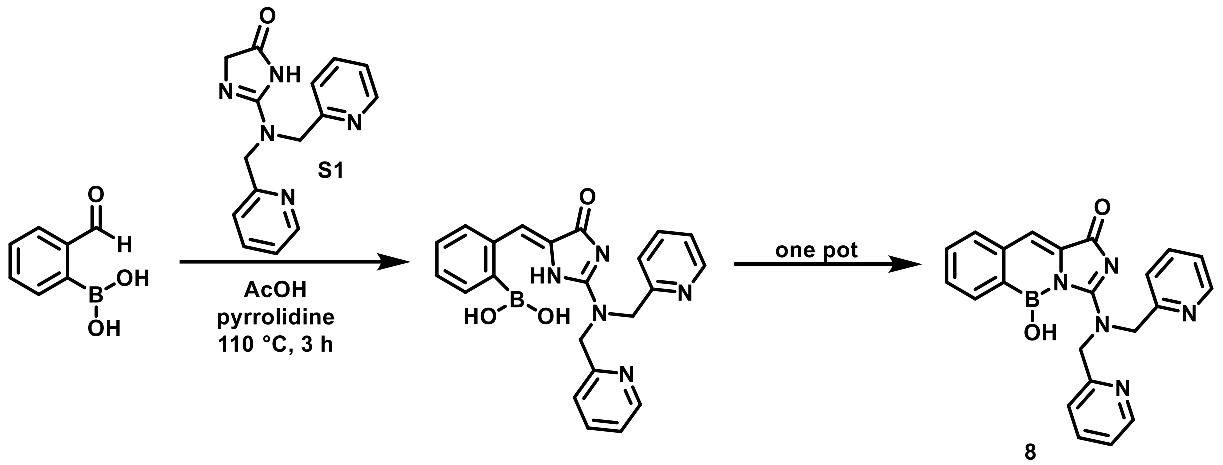

8
102 mg, 69%

56 mg 2-formylphenylboronic acid (0.37 mmol) was dissolved in 2 mL AcOH and 105 mg S1 was added (0.37 mmol). A catalytic amount of pyrrolidine (3% v/v) was added to the mixture and the mixture was heated to 110 °C on a hotplate magnetic stirrer, under nitrogen. After 3 hours of stirring, the orange solution turned deeper red, and the solvent was removed. The solid residue was dissolved in DMSO and purified using preparative HPLC in a TFA-containing eluent system described in S1.2. The pure fractions were collected, the MeCN was removed on a rotary evaporator and the aqueous suspension was lyophilized resulting the desired product 8. The ^{1}H NMR peaks indicate a strong H-bond between the pyridyl N and the B-OH (See Fig S8, Fig S68).

8: off-white powder, 102 mg, 69% yield

^{1}H NMR (500 MHz, CD_3OD) δ 8.51 (d, *J* = 6.0 Hz, 1H), 8.43 (d, *J* = 4.8 Hz, 1H), 8.29 (t, *J* = 7.7 Hz, 1H), 7.90 (t, *J* = 7.8 Hz, 1H), 7.76 (t, *J* = 6.9 Hz, 1H), 7.72 (d, *J* = 7.4 Hz, 1H), 7.67 (d, *J* = 7.8 Hz, 1H), 7.60 (s, 1H), 7.59 (s, 1H), 7.55 – 7.52 (m, 1H), 7.55 – 7.50 (m, 1H), 7.39 (t, *J* = 6.3 Hz, 1H), 7.15 (s, 1H), 6.89 (d, *J* = 15.1 Hz, 1H), 5.18 (d, *J* = 17.0 Hz, 1H), 5.12 (d, *J* = 17.0 Hz, 1H), 4.76 (d, *J* = 15.1 Hz, 1H).

^{13}C NMR (126 MHz, CD_3OD) δ 165.3, 159.4, 154.2, 152.6, 150.7, 147.5, 145.5, 139.0, 137.1, 135.4, 131.5, 131.2, 131.2, 130.4, 127.6, 125.0, 124.0, 123.0, 118.9, 59.2, 54.2.

HRMS-ESI *m/z* $[M+H]^+$ calcd. for $C_{22}H_{19}BN_5O_2$ 396.1627 found 396.1617.

S1.2.19. Preparation of 9

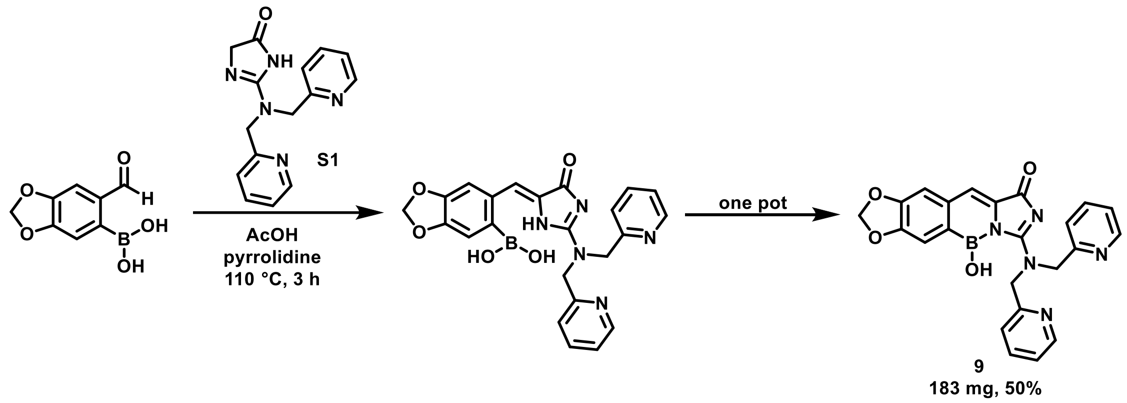

162 mg 2-formyl-4,5-methylenedioxyphenylboronic acid (0.84 mmol) was dissolved in 5 mL AcOH and 235 mg S1 was added (0.84 mmol). A catalytic amount of pyrrolidine (3% v/v) was added to the mixture and the mixture was heated to 110 °C on a hotplate magnetic stirrer, under nitrogen. After 3 hours of stirring, the orange solution turned deeper red, and the solvent was removed. The solid residue was dissolved in a mixture of MeOH and water and purified using preparative HPLC in an ammonium bicarbonate-containing eluent system described in S1.2. The pure fractions were collected, the MeCN was removed on a rotary evaporator and the aqueous suspension was lyophilized.

9: yellow powder, 183 mg, 50% yield

^{1}H NMR (600 MHz, $CDCl_3$) δ 8.49 (d, J = 5.4 Hz, 2H), 7.78 (t, J = 8.0 Hz, 2H), 7.49 (d, J = 7.8 Hz, 2H), 7.35 (t, J = 6.5 Hz, 2H), 7.14 (s, 1H), 6.83 (s, 1H), 6.61 (s, 1H), 6.00 (s, 2H), 5.43 (br s, 4H).

^{13}C NMR (151 MHz, $CDCl_3$) δ 178.2, 169.7, 154.9, 148.5, 148.2, 147.3, 139.7, 135.3, 133.0, 132.5, 126.5, 124.2, 112.4, 109.9, 108.6, 101.2, 54.3.

HRMS-ESI m/z $[M+H]^+$ calcd. for $C_{23}H_{19}BN_5O_4$ 440.1525 found 440.1515.

S1.2.20. Preparation of S4

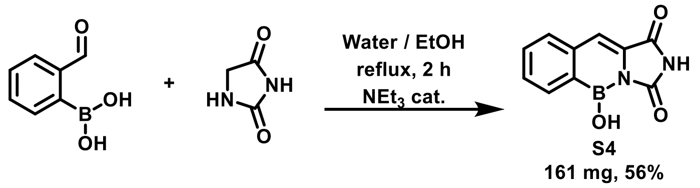

Compound S2 was prepared according to the literature method.[4] 133 mg (1.33 mmol) hydantoin was dissolved in 10 mL hot deionized water and the pH was set to 7 by adding $NaHCO_3$. 2-Formylphenylboronic acid (200 mg, 1.33 mmol) was dissolved in 10 mL EtOH and was added to the mixture. A drop of triethylamine was added as catalyst and the mixture was refluxed for 2 hours on a hotplate magnetic stirrer. A white precipitate formed which was filtered and dried. The NMR spectra of the compound is in good agreement with the one reported in the literature.

S4: white powder, 161 mg, 56 % yield

^{1}H NMR (400 MHz, DMSO-*d6*) δ 11.69 (s, 1H), 9.00 (s, 1H), 8.20 (d, J = 7.5 Hz, 1H), 7.78 (d, J = 7.8 Hz, 1H), 7.67 (dd, J = 7.8, 1.6 Hz, 1H), 7.52 (t, J = 7.4 Hz, 1H), 7.13 (s, 1H).

S1.2.21. Attempt on post-functionalization of the B-OH group with alcohols

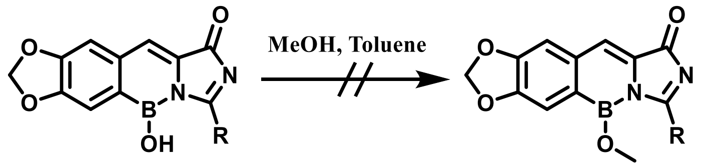

7a: R = NH-Ph
7b: R = Ph

7a: R = NH-Ph
7b: R = Ph

30 mg of 7a or 7b in a mixture of 20 mL methanol and 10 mL toluene was stirred on a hotplate magnetic stirrer at 70 °C in a dry round-bottom flask equipped with a reflux condenser under N_2 atmosphere for 18 hours. LC-MS analysis found no traces of methyl ester formation. While 7a proved to be stable, 7b underwent protodeboronation with considerable conversion (~70%) under the reaction conditions. Previous works reported, that sp^3 type boronic acid derivatives undergo esterification under the mentioned conditions. [5] The calulated B-N bond length for our compound is 1.46 Å. Calculated sp^3 type B-N length for 1,2-BN cyclohexane is reported to be 1.66 Å, while sp^2 character B-N bond for 1,2-BN cyclohexene is 1.40 Å, and the aromatic 1,2-dihydro-1,2-azaborine contains a 1.45 Å B-N bond. [6] This suggests, that the B-N bond in our compounds have a rather sp^2 character, which may explain the observed lack of reactivity.

S1.3. Spectroscopical characterization of the prepared fluorophores

S1.3.1. Determination of spectrophysical properties

Absorption spectra were recorded with a 1 nm slit width in the high-speed mode setting of the instrument. Fluorescence spectra were recorded using 600 nm min^{-1} scan speed and 3 nm slit widths both for excitation and emission. UV-Vis spectra were corrected with the background of the solvents before adding the dye stock solutions. The spectra were recorded in different solvent systems: DMSO, MeOH and DCM. In each case, the deprotonated and the protonated products were studied by either addition of 0.05 v/v% base (DIPEA) to the solutions or addition of the same quantity of acid (TFA) to the solutions. Values measured in DCM ofter agreed with the DCM containing TFA, due to the HCl traces from the decomposition of DCM. In addition, the spectra were also studied in aqueous solutions at different pH levels (acetate and HEPES buffers). Acetate (50 mM) buffers between pH = 3.5 and pH = 5.0 and HEPES (50 mM) buffers pH = 6.0 and pH = 9.0 were prepared by dissolving the free acid (50 mM) in DI water and adding conc. NaOH (20% *m*/*m*) until the desired pH level was reached.

The UV-Vis and fluorescent spectra were acquired from solutions containing ca. 1 µM of the studied dye. The fluorescence spectra were normalized with the actual concentration, emission spectra were recorded by excitation in the absorption maximum. Excitation spectra were measured in the emission maximum wavelength. If the absorption and excitation maxima were not the same, the emission spectra were recorded again by excitation in the wavelength of the emission maximum. The individual stock solutions were prepared by dissolving 1-2 mg of the compounds in 10 mL DMSO and then adding 10 µL aliquots of the stock solutions to 1.99 mL of solvent.

The molar absorption coefficient (ε) was calculated using equation (1), where A stands for absorbance, c for the concentration in M and l for the path length of the cuvette in cm:

$$\varepsilon \,/\, (\mathrm{dm^3(mol\ cm)^{-1}}) = A\, c^{-1}\, l^{-1} \quad (1)$$

The fluorescence quantum yields were determined based on the recorded fluorescence spectra using the relative method as the literature.[7] Eq. (2) was used to determine the fluorescence quantum yield, where subscript 'ref' marks the reference and 's' is the sample, A stands for the absorbance, $\int F$ for the area under the emission curve and 'n' for the refractive index of the solvents (both were taken for 1.33 as dilute aqueous solutions). Fluorescein in 0.1 M aqueous NaOH solution was used as a standard ($\Phi_{\mathrm{ref}} = 0.95$) for 7b and 9, whereas Coumarin 1 in EtOH ($\Phi_{\mathrm{ref}} = 0.73$) was used for the other derivatives.

$$\Phi = \Phi_{\mathrm{ref}} \times \frac{\int F_{\mathrm{s}}}{\int F_{\mathrm{ref}}} \times \frac{A_{\mathrm{ref}}}{A_{\mathrm{s}}} \times \frac{n_{\mathrm{s}}^{\mathrm{D}}}{n_{\mathrm{ref}}^{\mathrm{D}}} \quad (2)$$

Two-photon cross sections (δ) are reported in Goeppert-Mayer units denoted by GM and were measured using a similar relative method. For reference 20 μM Rhodamine 6G or Coumarin102 in MeOH were used.[8–10] Approximately 100 μM samples of the dyes were studied in different solvents. To achieve this, 1 mM stock solutions in DMSO were prepared, which were diluted 10x with the desired solvent. The samples were loaded into capillary tubes and investigated with a 2P microscope described in S1.1. The incident light was focused into the capillary and the average intensity of the emitted light in the field of view was recorded ($F^{2\mathrm{P}}$). The power of the laser source was kept constant at 32 mW and the excitation wavelength was stepwise modified in the $\lambda_{\mathrm{ex}} = 700 - 1040$ nm range. The emitted light was detected over the 440–480, 492–562 or the 570–620 nm range depending on the 1P emission spectrum of the studied fluorophore. δ was calculated using eq. 3., in which the subscript 'ref' marks the reference and 's' stands for sample. The markings c, n and Φ stand for concentration, solvent refractive index, and one-photon (1P) quantum yield respectively, while the integral ratios are introduced as a correction factor derived from the 1P emission spectra to compensate for the different amount of light cut off by the dichroic mirror of the microscope in case of the reference and sample. λ_{upper} and λ_{lower} refers to the upper and lower wavelengths of the used bandpass filters This correction assumes that the shape of the 1P and 2P emission spectra are identical.

$$\delta = \delta_{\mathrm{ref}} \times \frac{F_{\mathrm{s}}^{2\mathrm{P}} \times c_{\mathrm{ref}} \times n_{\mathrm{ref}}^{\mathrm{D}}}{F_{\mathrm{ref}}^{2\mathrm{P}} \times c_{\mathrm{s}} \times n_{\mathrm{s}}^{\mathrm{D}}} \times \frac{(\int_{\lambda\mathrm{lower}}^{\lambda\mathrm{upper}} F / \int F)_{\mathrm{ref}}}{(\int_{\lambda\mathrm{lower}}^{\lambda\mathrm{upper}} F / \int F)_{\mathrm{s}}} \times \Phi_{\mathrm{s}}^{-1} \quad (3)$$

Where fluorescence was detectable in aqueous solutions, the pK_{a} of the fluorophores were determined. The fluorescence intensities were measured at a series of pH levels (3.5, 4, 5, 6, 7, 7.4, 8, 9) using the buffers prepared as mentioned above. The intensity values were plotted against the pH level and a nonlinear regression was used to determine the K_{a} value using equation (4) derived from the Henderson-Hasselbach equation:

$$y = F_{\mathrm{min}} + \frac{F_{\mathrm{min}} - F_{\mathrm{max}}}{1 + 10^{(\mathrm{p}K_{\mathrm{a}} + \mathrm{x})}} \quad (4)$$

The absorption and fluorescence of the Zn^{2+} sensors were also studied similarly. The probes were studied in Zn^{2+}-free solutions at pH = 7.4 (HEPES buffer, prepared the way described above), and in the same buffer but also containing different amounts of Zn^{2+}. The fluorescence

was plotted against the logarithm of free Zn^{2+} concentration. A nonlinear regression was used to determine the K'_d similarly to the K_a determination using equation (5).

$$y = F_{\min} + \frac{F_{\min} - F_{\max}}{1 + 10^{(\mathrm{p}K'_d + x)}} \tag{5}$$

The turn-on measurements show the data recorded in Zn^{2+}-free solutions and solutions containing 10 mM Zn^{2+}. The sensor concentrations were kept around 1 μM in the case of 1P measurements and 100 μM in the 2P case. The areas under the fluorescence curves were used to calculate the brightness and $\Delta F/F$ values as well. The excitation was performed at the excitation maximum of the studied species. The selectivity of the sensors was determined by dissolving the relevant salts in the same buffer. Salt concentrations were set to 10 mM. Solutions containing 10 mM Zn^{2+} and the same amount of the competing ion were also studied to investigate the interference caused in the presence of Zn^{2+}. The studied salts were the following: $CaCl_2$, $MgCl_2$, NaCl, KCl, $Cd(NO_3)_3$, $NiCl_2$, $MnCl_2$. The other instrument settings and methods were kept the same as described above.

The photostability of the dyes and sensors was assessed by irradiating 200 μM solutions of the prepared dyes in a ThalesNano PhotoCube photoreactor with 10% violet and 10% blue channel intensity (see Fig S5.), in low power mode, using 4 LED panels and a 480 rpm stirring. The fluorescence of the solutions was measured before the irradiation, then at 2, 5, 10, 20, 40 and 60 minutes after the start of the irradiation experiment. The solutions were prepared by diluting the previously prepared dye stock solutions using DMSO in the case of **1b, 7a,** and **7b**, or 0.1 M NaOH for fluorescein, measured for reference. The Zn^{2+} sensor **9** was measured both in zinc-free HEPES buffer and zinc-containing buffer using the same dilution method. The fluorescence intensities were normalized to the one measured before irradiation and plotted against the time of irradiation.

S1.3.2. Spectrophysical data of all the reported compounds

Table S1. Spectroscopical characteristics of the prepared fluorophores in different solvents.

Probe	Solvent	λ_{abs} / nm	λ_{ex} / nm	λ_{em} / nm	$\Delta_{Stokes}\lambda$ / nm	Φ	ε_{max} / $dm^3 \cdot mol^{-1} \cdot cm^{-1}$	Brightness
1a	MeOH	375	375	417	42	0.013	51 600	700
	DMSO	365	375	412	37	0.029	38 300	1100
	DCM	351	375	435	60	0.32	22 800	7300
	MeOH+DIPEA	376	373	417	44	0.032	24 000	800
	DMSO+DIPEA	370	370	415	45	0.040	18 100	700
	DCM+DIPEA	350	367	414	47	0.14	19 700	2700
	MeOH+TFA	370	370	415	45	0.009	21 400	200
	DMSO+TFA	352	352	393	41	0.004	19 500	100
2a	MeOH	379	378	427	49	0.035	23 900	800
	DMSO	365	370	411	41	0.078	20 700	1600
	DCM	349	345	445	100	0.005	22 000	100
	MeOH+DIPEA	379	376	429	53	0.042	26 600	1100
	DMSO+DIPEA	369	369	414	45	0.036	21 900	800
	DCM+DIPEA	349	367	402	35	0.086	22 400	1900
	MeOH+TFA	356	353	500	147	0.015	22 900	400
	DMSO+TFA	349	387	437	50	0.004	25 000	100
4a	MeOH	376	343	400	57	0.048	31 800	1500
	DMSO	368	375	408	33	0.3	26 000	7900
	DCM	356	344	370	26	0.022	35 200	800
	MeOH+DIPEA	376	347	409	62	0.036	34 600	1200
	DMSO+DIPEA	370	378	425	47	0.075	28 300	2100
	DCM+DIPEA	356	373	406	33	0.45	31 200	13 900
	MeOH+TFA	358	340	389	49	0.028	36 500	1000
	DMSO+TFA	356	346	386	40	0.01	30 500	300
5a	MeOH	375	375	432	57	0.012	33 820	400
	DMSO	362	375	421	46	0.017	28 600	500

	DCM	346	375	435	60	0.28	38 200	10 500
	MeOH+DIPEA	376	376	423	47	0.014	39 800	600
	DMSO+DIPEA	370	380	427	47	0.028	33 500	900
	DCM+DIPEA	346	371	408	37	0.12	36 000	4300
	MeOH+TFA	356	357	398	41	0.003	37 700	100
	DMSO+TFA	348	348	388	40	0.002	36 200	60
6a	MeOH	378	376	421	45	0.019	33 000	600
	DMSO	370	375	408	33	0.43	23 300	10 100
	DCM	356	345	372	27	0.003	31 900	100
	MeOH+DIPEA	378	377	423	46	0.021	32 900	700
	DMSO+DIPEA	378	384	423	39	0.15	25 400	3700
	DCM+DIPEA	356	374	408	34	0.68	27 300	18 400
	MeOH+TFA	376	376	423	47	0.004	32 600	100
	DMSO+TFA	356	346	421	75	0.0013	27 600	40
7a	MeOH	404	389	466	77	0.47	38 100	18 000
	DMSO	372	378	440	62	0.30	30 000	9000
	DCM	364	360	420	60	0.021	29 500	600
	MeOH+DIPEA	406	388	466	78	0.52	40 700	21 300
	DMSO+DIPEA	374	377	439	62	0.15	34 400	5300
	DCM+DIPEA	364	377	443	66	0.10	27 800	2900
	MeOH+TFA	402	389	504	115	0.36	39 900	14 500
	DMSO+TFA	364	364	450	86	0.02	30 600	600
1b	MeOH	400	400	478	78	0.047	16 200	800
	DMSO	412	422	499	77	0.41	15 900	6600
	DCM	396	382	460	78	0.019	25 500	500
	MeOH+DIPEA	400	388	453	65	0.10	79 00	800
	DMSO+DIPEA	404	500	417	83	0.23	49 00	1100
	DCM+DIPEA	408	400	464	64	0.20	19 700	3900
	MeOH+TFA	392	392	443	51	0.01	15 300	200
	DMSO+TFA	390	389	459	70	0.015	21 200	300

2b	MeOH	404	413	499	86	0.031	20 400	600
	DMSO	394	388	460	72	0.027	28 100	800
	DCM	378	386	460	74	0.022	24 800	600
	MeOH+DIPEA	412	416	497	81	0.52	17 400	9100
	DMSO+DIPEA	398	416	491	75	0.14	14 600	2000
	DCM+DIPEA	392	407	488	81	0.083	20 100	1700
	MeOH+TFA	378	376	573	197	0.022	27 300	600
	DMSO+TFA	394	389	460	71	0.034	18 600	600
6b	MeOH	404	413	482	69	0.28	25 800	7300
	DMSO	396	394	488	94	0.056	23 400	1300
	DCM	394	388	463	75	0.10	27 800	2800
	MeOH+DIPEA	406	406	483	77	0.36	25 000	9100
	DMSO+DIPEA	404	421	507	86	0.24	15 700	3800
	DCM+DIPEA	404	405	480	75	0.24	28 900	6900
	MeOH+TFA	396	387	457	70	0.010	34 000	400
	DMSO+TFA	394	387	464	77	0.022	25 400	600
7b	MeOH	442	438	538	100	0.57	16 200	9300
	DMSO	436	425	524	99	0.63	16 200	10 200
	DCM	460	462	557	95	0.73	17 400	12 800
	MeOH+DIPEA	436	437	526	89	0.67	18 300	12 200
	DMSO+DIPEA	424	426	522	96	0.70	16 200	11 400
	DCM+DIPEA	430	430	524	94	0.52	15 800	8200
	MeOH+TFA	452	454	575	121	0.35	16 400	5700
	DMSO+TFA	434	445	587	142	0.055	17 300	950
10	MeOH	344	400	342	58	0.94	24 000	22 600
	DMSO	342	391	340	51	0.16	22 000	1600
	DCM + DIPEA	340	364	338	26	0.36	22 400	8100
	HEPES pH 7.4	350	409	348	61	0.03	24 000	360

Table S2. Spectroscopical characteristics of the prepared fluorophores at different pH levels in aqueous solutions.

**Probe**	**pH**	λ_{abs} **/ nm**	λ_{ex} **/ nm**	λ_{em} **/ nm**	$\Delta_{Stokes}\lambda$ **/ nm**	**Φ**	ε_{max} **/ dm^3·mol^{-1}·cm^{-1}**	**Brightness**
1a	3.5	373	373	412	39	0.0004	15 600	20
	4	373	373	412	39	0.0005	15 800	20
	5	373	373	412	39	0.0005	16 900	30
	6	373	373	412	39	0.0006	16 200	30
	7	375	375	426	51	0.0011	15 600	50
	7.4	375	375	431	56	0.0006	15 600	30
	8	375	375	412	37	0.0019	16 000	100
	9	375	375	412	37	0.0013	16 400	100
2a	3.5	358	376	532	156	0.0051	23 300	100
	4	357	378	533	155	0.0053	21 700	100
	5	360	377	532	155	0.0061	21 600	100
	6	374	374	522	148	0.0038	20 400	100
	7	375	374	430	56	0.0026	22 900	100
	7.4	375	376	431	55	0.0020	23 700	50
	8	377	378	433	55	0.0020	23 400	50
	9	378	378	435	57	0.0023	23 300	100
4a	3.5	360	347	422	75	0.0012	34 000	40
	4	362	346	422	76	0.0013	28 000	40
	5	372	347	421	74	0.001	33 500	30
	6	374	350	422	72	0.0012	32 700	40
	7	376	352	422	70	0.0012	31 100	40
	7.4	374	366	421	55	0.010	28 800	300
	8	376	352	422	70	0.0011	32 300	40
	9	376	356	422	66	0.0013	31 700	40
5a	3.5	360	360	410	50	0.001	37 400	40
	4	358	359	408	49	0.001	45 300	50
	5	372	371	425	54	0.001	43 400	20
	6	374	374	429	55	0.001	42 700	40

	7	374	374	429	55	0.001	42 400	40
	7.4	374	374	428	54	0.001	41 000	50
	8	374	374	428	54	0.001	42 700	40
	9	374	374	428	54	0.001	42 000	40
6a	3.5	366	366	422	56	0.0004	31 900	10
	4	376	376	421	45	0.0003	30 900	10
	5	376	376	422	46	0.0006	31 400	20
	6	376	376	421	45	0.0005	31 600	20
	7	378	378	422	44	0.0005	31 400	20
	7.4	378	378	421	43	0.0007	31 300	20
	8	380	380	421	41	0.0006	29 500	20
	9	378	378	422	44	0.0006	31 500	20
7a	3.5	401	401	520	119	0.100	19 100	1900
	4	401	401	520	119	0.079	26 100	2100
	5	401	401	518	117	0.010	21 600	2200
	6	398	398	511	113	0.087	20 400	1800
	7	397	397	488	91	0.066	17 800	1200
	7.4	396	396	497	101	0.065	23 500	1500
	8	398	398	492	94	0.057	25 500	1500
	9	399	399	490	91	0.057	22 300	1300
1b	3.5	388	390	449	59	0.004	30 000	110
	4	388	389	450	61	0.005	28 000	130
	5	388	388	450	62	0.004	33 200	140
	6	390	390	450	60	0.009	30 500	300
	7	394	389	475	86	0.026	32 600	800
	7.4	396	387	475	88	0.029	34 400	1000
	8	396	390	475	85	0.035	25 800	900
	9	396	388	475	87	0.034	36 000	1200
2b	3.5	376	376	431	55	0.007	10 200	100
	4	376	376	432	56	0.005	11 100	100

	5	367	367	419	52	0.018	47 00	100
	6	372	392	455	63	0.017	11 100	200
	7	394	390	481	91	0.049	8300	400
	7.4	401	390	478	88	0.06	8200	500
	8	400	390	480	90	0.057	8700	500
	9	401	391	480	89	0.067	8300	600
6b	3.5	394	387	474	87	0.025	30 500	800
	4	394	388	472	84	0.023	30 400	700
	5	394	389	478	89	0.026	31 200	800
	6	396	392	480	88	0.085	28 500	2400
	7	400	392	479	87	0.24	25 100	6000
	7.4	400	392	479	87	0.29	23 100	6800
	8	400	391	478	87	0.30	22 800	6900
	9	400	393	479	86	0.31	27 000	8300
7b	3.5	450	448	585	137	0.14	15 800	2200
	4	450	449	585	136	0.14	16 900	2400
	5	450	445	585	140	0.15	16 200	2400
	6	448	444	575	131	0.18	16 700	3100
	7	438	436	560	124	0.33	17 300	5800
	7.4	436	435	555	120	0.35	17 100	6100
	8	434	436	552	116	0.44	18 500	8200
	9	434	434	551	117	0.45	18 300	8300

Table S3 Spectroscopical characteristics of the prepared fluorescent Zn^{2+} sensors in Zn^{2+}-free solutions and at the endpoint of Zn^{2+} titrations.

**Probe**	**State**	λ_{abs} **/ nm**	λ_{ex} **/ nm**	λ_{em} **/ nm**	$\Delta_{Stokes}\lambda$ **/ nm**	**Φ**	ε_{max} **/ $dm^3 \cdot mol^{-1} \cdot cm^{-1}$**	**Brightness**	$\lambda_{2P\ abs}$ **/ *nm***	δ **/ GM**	$\Delta F/F$
8	Free	358	379	410	31	0.004	24 800	100	-	-	31
	Zn^{2+} cpx.	358	379	438	59	0.12	27 300	3800	760	0.03	
9	Free	390	384	496	112	0.07	24 800	1700	-	-	3.6
	Zn^{2+} cpx.	394	420	533	115	0.31	25 200	7800	820	1.2	

S1.3.3. Raw spectra of the reported fluorophores

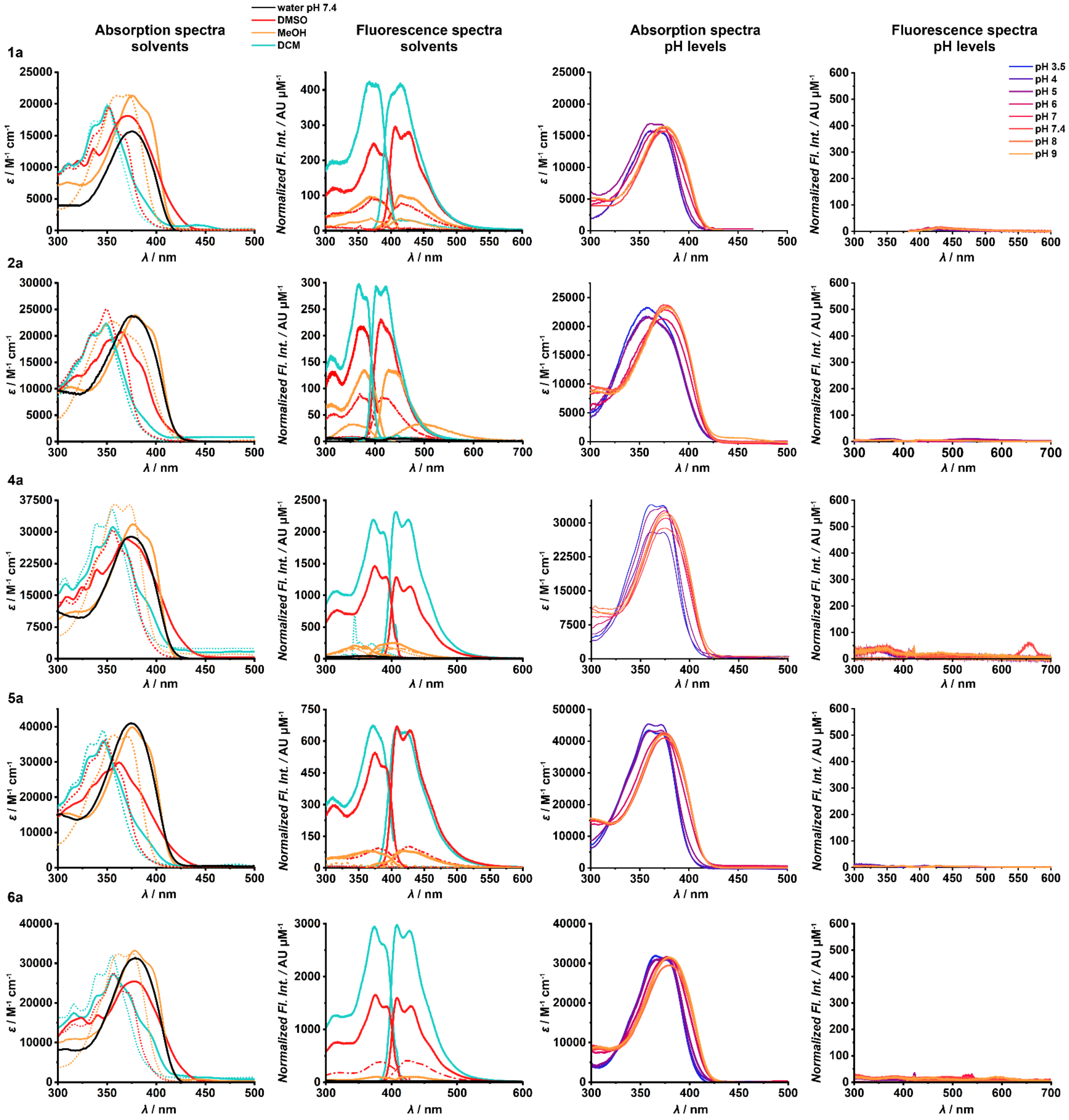

Fig S1. Absorption and fluorescence spectra of the prepared fluorophores recorded in different solvents or aqueous solutions at various pH levels. Different colors represent different solvents: black: water, red: DMSO, yellow: MeOH, turquoise: DCM. The dotted lines represent the protonated form of the compounds (achieved by adding 1 μL TFA to 3 mL solutions) and the dashed and dotted lines represents where the compound underwent further deprotonation by adding 1 μL DIPEA to the 3 mL solutions. (continued next page).

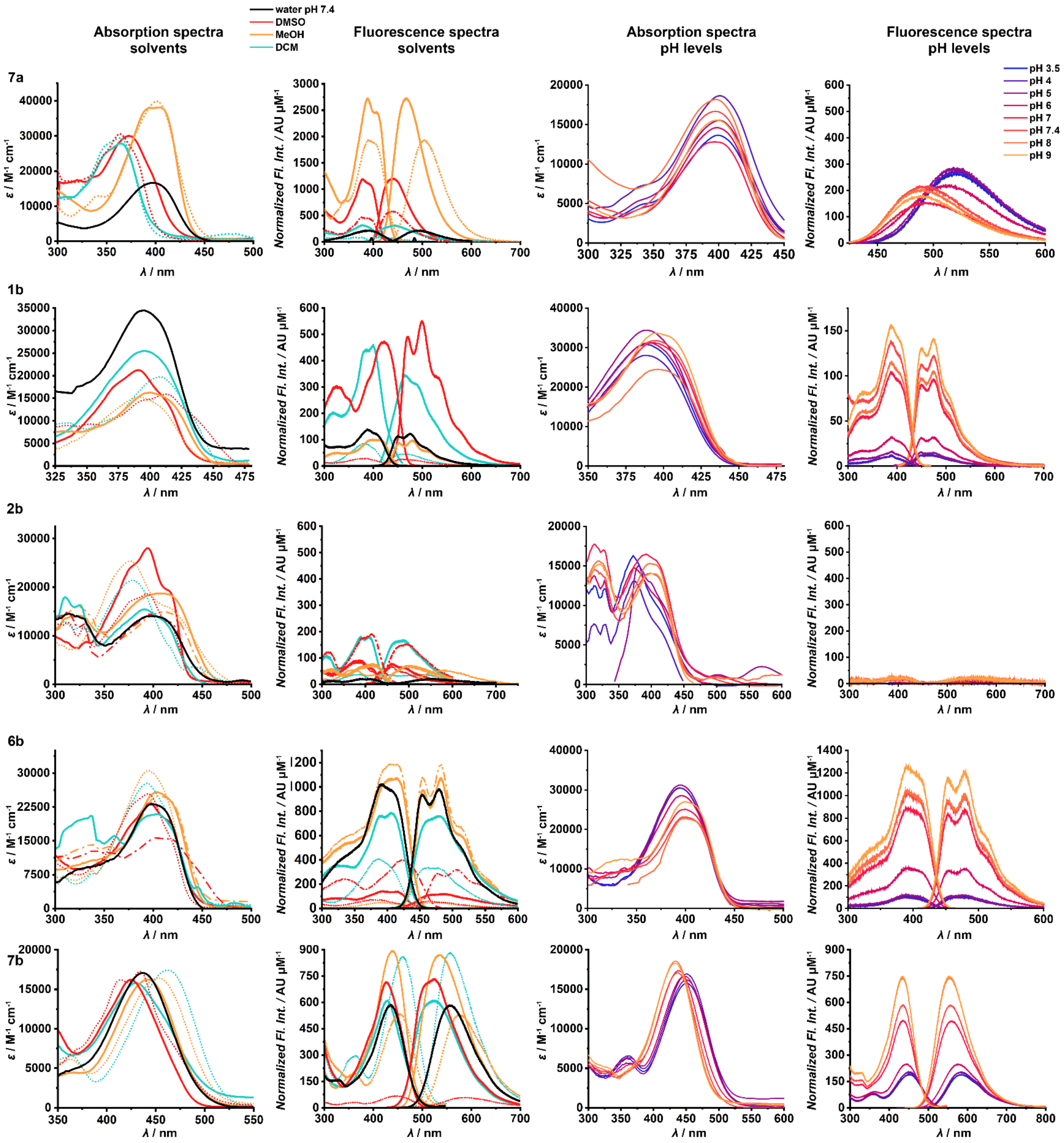

Fig S1. Absorption and fluorescence spectra of the prepared fluorophores recorded in different solvents or aqueous solutions at various pH levels. Different colors represent different solvents: black: water, red: DMSO, yellow: MeOH, turquoise: DCM. The dotted lines represent the protonated form of the compounds (achieved by adding 1 μL TFA to 3 mL solutions) and the dashed and dotted lines represents where the compound underwent further deprotonation by adding 1 μL DIPEA to the 3 mL solutions. (continued from previous page).

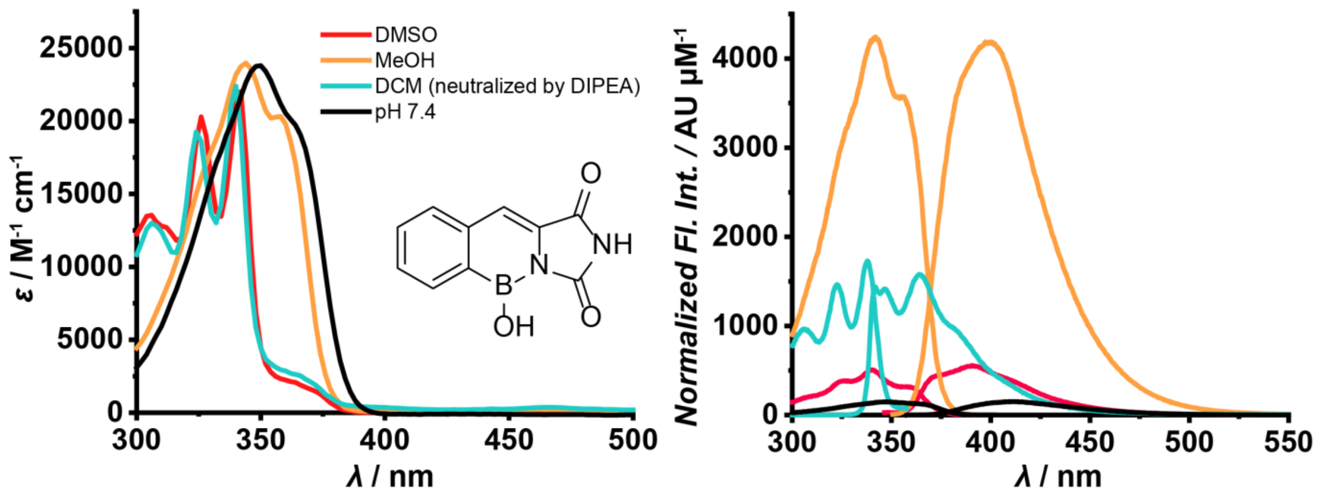

Fig S2. Previously reported imidazolidine-2,4-dione **S2** was studied to investigate its previously unknown fluorescent properties. Absorption and fluorescence spectra of **S2** recorded in different solvents.

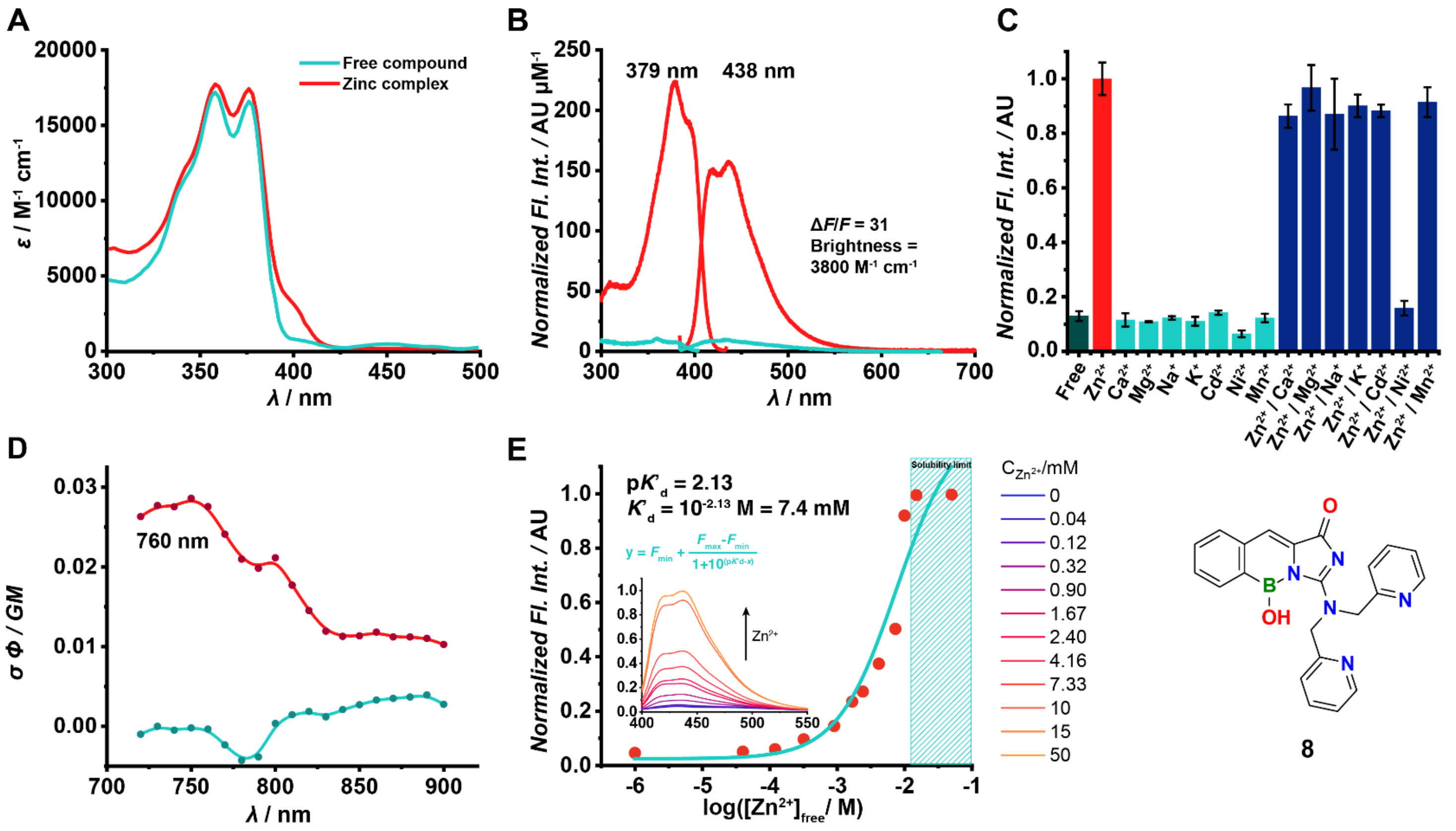

Fig S3. A. UV-Vis and B. normalized fluorescence spectra of **8** in free (turquoise) and Zn^{2+} containing (red) solutions (pH = 7.4, HEPES). C. Selectivity of the reported Zn^{2+} sensor **8**. Normalized fluorescence intensity in the presence of Zn^{2+} (red), interfering ions (turquoise) and both simultaneously (blue). Error bars represent the standard deviation of triplicate measurements. D. 2P action cross section spectra of **8** in zinc-free aqueous solution (turquoise) and zinc containing solution (red). E. Fluorometric titration of **8** with Zn^{2+}. The fluorescence intensity is plotted against the free Zn^{2+} concentration on a logarithmic scale. The line represents the fitted equation shown in turquoise. K'_d was determined using nonlinear regression. The area with striped pattern shows the concentration range, where colloidal Zn^{2+} precipitation was observed.

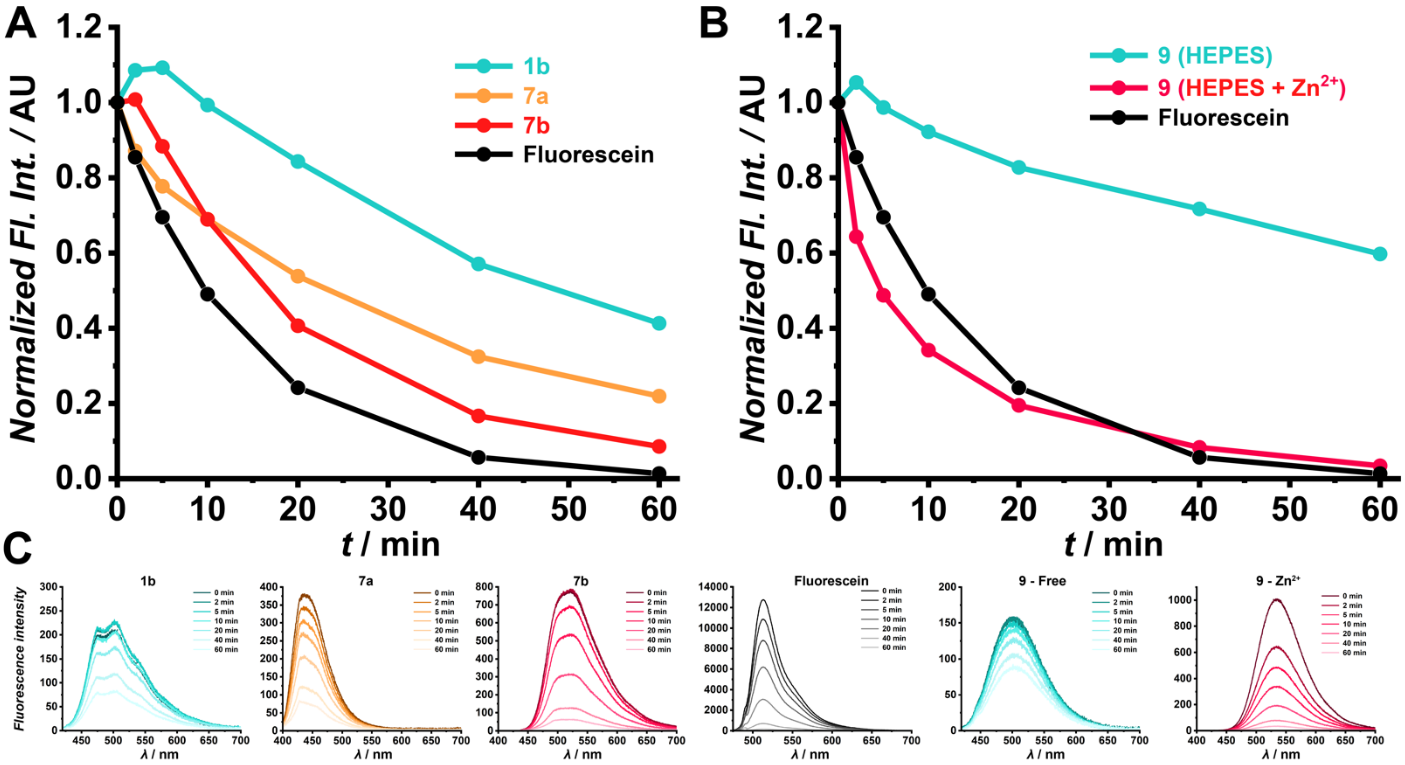

Fig S4. A. Normalized fluorescence intensity of compounds **1b, 7a** and **7b** in DMSO and B. **9** in zinc-free and zinc-contatining HEPES Buffer againts the time of irradiation. The black line represents the results of fluorescein in 0.1 M NaOH under the same experimental procedure for reference. C. The raw fluorescence spectra obtained after the start of irradiation. The shape of the spectra did not change in any case, only the intensity.

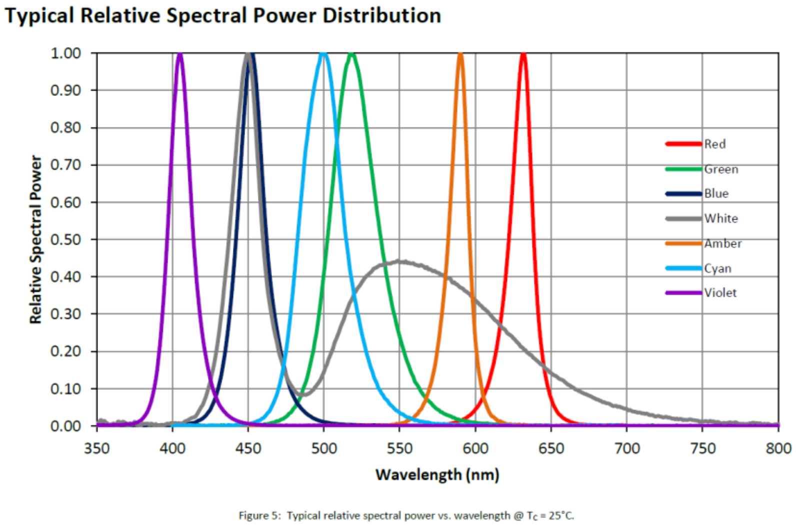

Fig S5. The spectra of the LEDs found in the used photoreactor. The blue and volet channels were used at a 10% power setting. Source: ThalesNano.

S1.4. Biological studies

HEK-293 cells (CRL-1573, ATCC) were cultured in Dulbecco's Modified Eagle's Medium (DMEM) supplemented with 10% fetal bovine serum (FBS), 100 U/mL penicillin and 100 μg/mL streptomycin. Cells were incubated (37 °C with 5% CO_2) in a humidified incubator and subcultured every 2-3 days. Then, the cultures were plated onto poly-lysinated glass coverslips at a density of approximately 25000 cells/cm^2 and further incubated for two more days. The culturing DMEM solution was replaced with 300 μL HEK-extracellular solution containing the following (in mM): 140 Na-gluconate, 5 K-gluconate, 3 $CaCl_2$, 1 $MgCl_2$, 5 D-glucose, 10 HEPES adjusted to pH 7.4 using NaOH. 5 mM DMSO solutions were prepared from fluorophore **7b** and probe **9** for the stainings. For the general staining experiment 3 μL of the stock solution of **7b** was added and the well was kept at 37 °C for 5 minutes (final dye concentration was 50 μM). The immersion was removed, and the well was washed with 500 μL of HEK-extracellular solution. The coverslip was placed under the microscope and imaging was started in 100 μL immersion of HEK-extracellular solution immediately. The fluorescence was detected in the green channel of the microscope (see S1.1), the excitation wavelength was 860 nm, and the laser intensity was set to 22 mW (Fig S6).

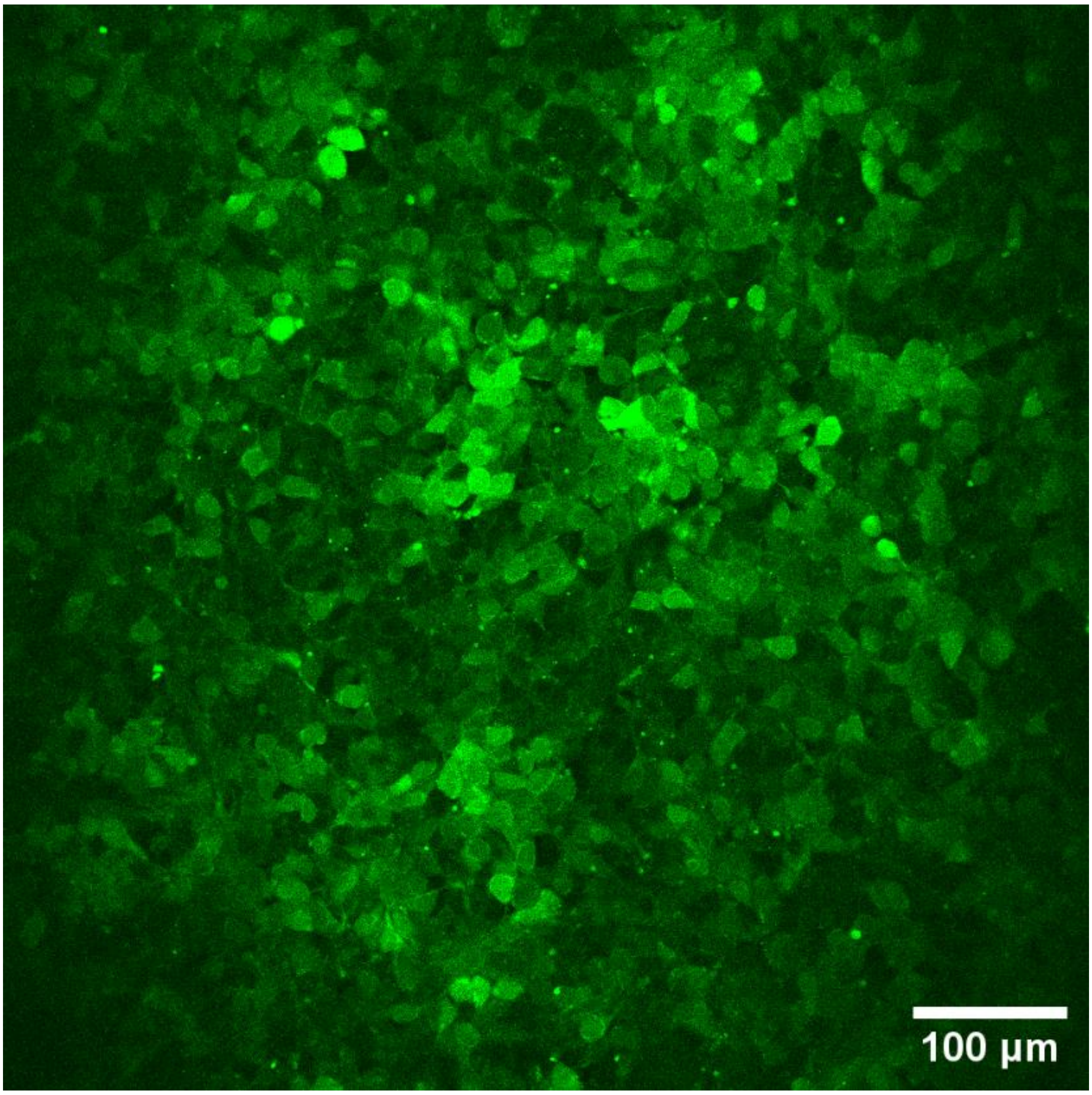

Fig S6. Two-photon image of HEK-293 cells stained with **7b** (860 nm, 22 mW, green channel, 50 μM).

For Zn^{2+} detection experiments 3 μL of the stock solution of **9** was added and the well was kept at 37°C for 5 minutes (final dye concentration was 50 μM). In another well the same protocol was followed, but 1 μL of 100 mM zinc pyrithione in DMSO was also added (330 μM final zinc concentration). In both cases after 5 minutes the solution was removed from the cells and the sample was washed with 500 μL of fresh solution, then placed under the imaging system. The imaging was carried out in 100 μL immersion similarly as described above. The detection was in the green channel, with an excitation wavelength of 820 nm and laser intensity of 11 mW. The fluorescence of the zinc-free sample was monitored for 2.5 minutes, then 1 μL of the zinc pyrithione solution was added to the immersion (1 mM final zinc concentration). The fluorescence increased and was monitored for further 5 minutes. Then 30 mL of 3 mM TPEN solution in DMSO was also added to the immersion (final concentration 1 mM), which quenched the fluorescence (Fig. S7).

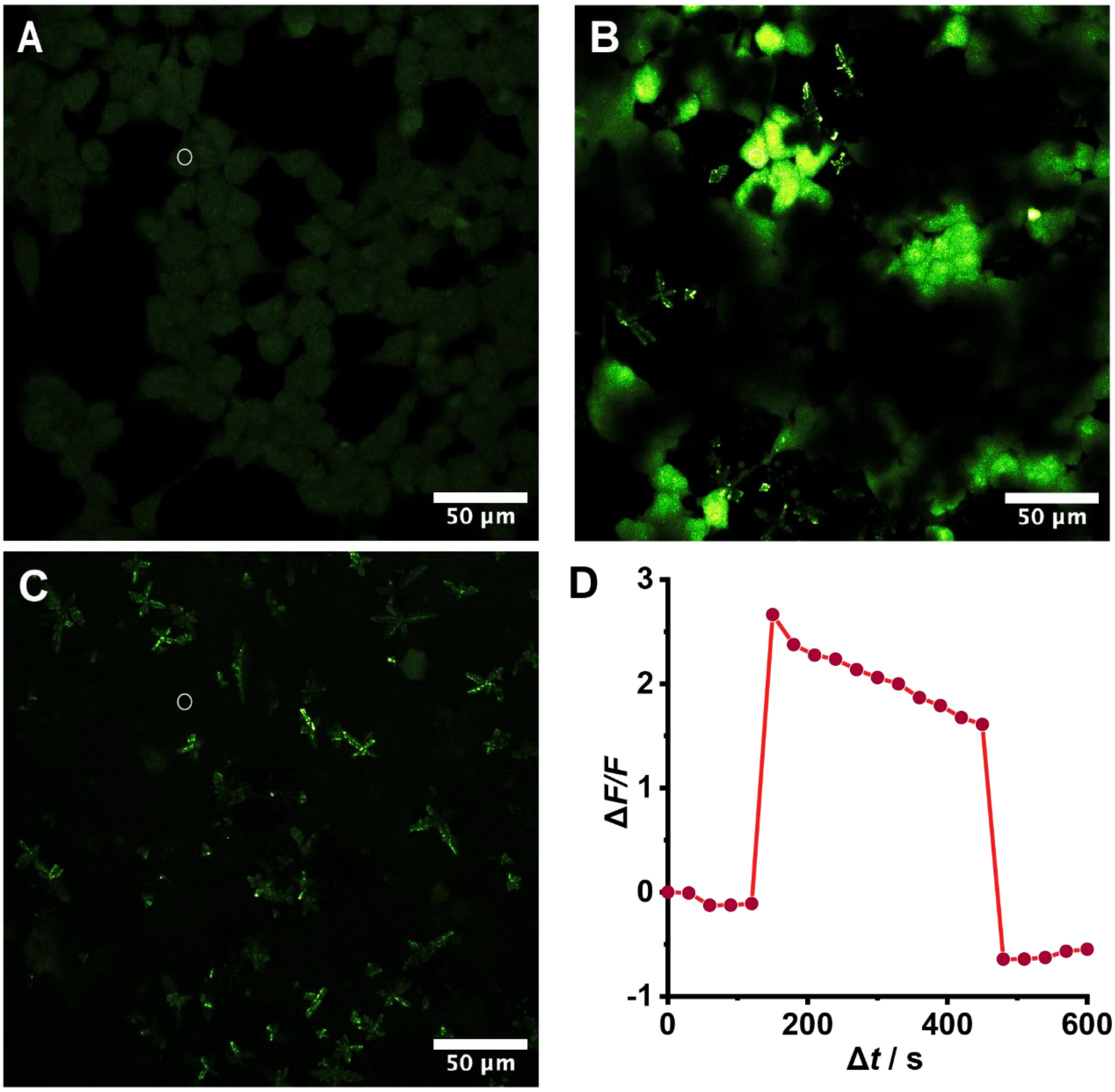

Fig S7. A. Two-photon image of HEK-293 cells stained with **9** (820 nm, 11 mW, green channel, 50 μM) B. after addition of zinc pyrithion solution (1 mM) C. after addition of TPEN solution (1 mM). D. the relative fluorescence intensity in the region of interest marked by the white circle against the elapsed time from the start of the experiment.

S1.5. Computational studies

S1.5.1. Computational methods

Theoretical calculations were carried out with Gaussian16 software[11] using the standard convergence criteria given as default. Optimization and vibrational frequencies were carried out with B3LYP method using the 6-31G(d,p) basis set. IEFF-PCM method (ε = 78.35 for water; given by G16) was used to account for the solvent effects.[12,13] Thermodynamic functions were computed at 298.15 K. For wavelength prediction, the vertical excitation was modelled by the TD-B3LYP/6-31G(d,p)[PCM(water)] level of theory using the optimized ground state geometries. The emission wavelengths were calculated after optimization using geometries provided by TD-B3LYP/6-31G(d,p)[PCM(water)]. LE abbreviates local excitation.

S1.5.2. Theoretical results – photochemical mechanisms

The photochemical mechanisms causing quenching were investigated for the different substituent patterns and solvents. The first group of compounds (**1a-7a**) exists in an equilibrium rich in possible rotamers and tautomers, while the second group (**1b-7b**) has only one highly favoured tautomer (Scheme S1).

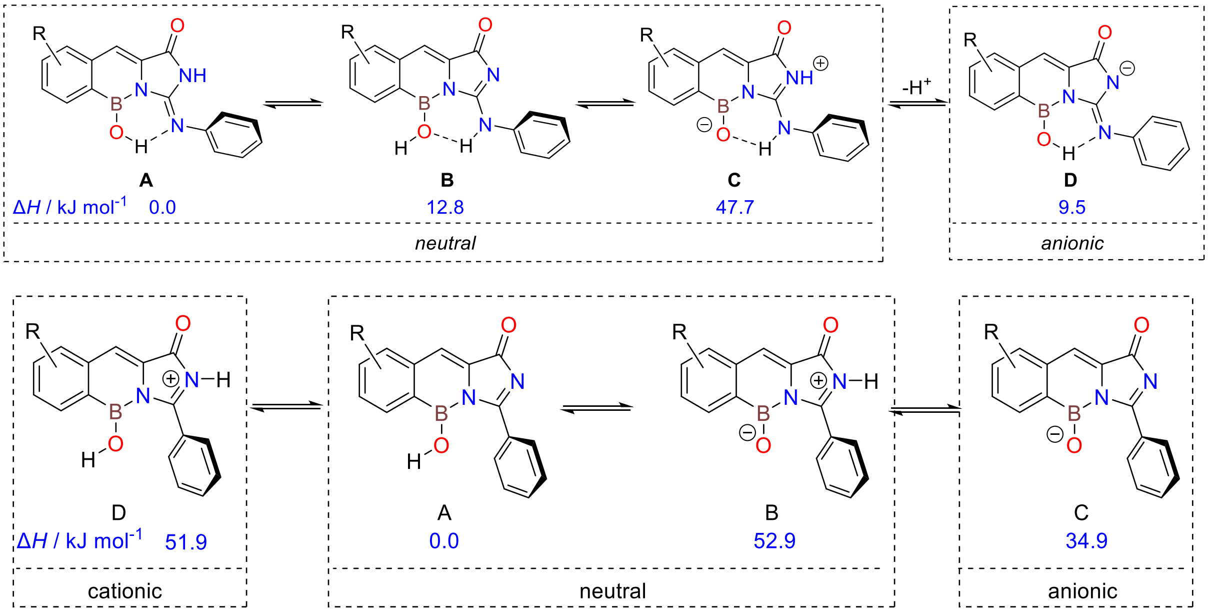

Scheme S1. Optimized structures at the ground state equilibrium representing compounds **1a-7a** and **1b-7b** (B3LYP/6-31G(d,p)[PCM(water)]). Calculated enthalpy values (kJ mol^{-1}) are given in blue for compound **1a**.

The NMR spectra of **2a** and **7a** also confirm this with a multitude of signals corresponding to species in exchange processes. The HPLC and HRMS data at the same time confirm the purity of the isolated compounds. According to computations, two forms (A and B) have lower energy therefore they probably represent the structures in solution. The enthalpy differences between these two structures A and B are between 12.1–13.9 kJ mol^{-1} (depending on substituent). The excitation wavelengths [λ_{ex}; $S_0 \rightarrow S_1$(LE)], calculated for the forms A and B are similar in all cases. Only the methylenedioxy (MDO) substituted derivative (**7a**) had a redshifted absorption according to the calculation (λ'_{ex} ca. 400 nm). This tendency is in good agreement with the experimental values, slightly overestimating the wavelengths systematically (Table S4; with ca.

20 nm). The relaxation processes of A and B at the excited states [λ_{ex}; S_1(LE) → S_1(opt)] LE = local excitation) differ significantly. At the excited state, the more stable Form A may undergo a nearly barrierless TICT process. Here the anilinyl moiety rotates around the C–N bond, which results in a high calculated wavelength, with zero oscillatory strength (*f*). This is a typical indicator of non-radiative processes. In contrast, the less stable Form B does not undergo significant geometrical change, which results in calculated fluorescence wavelengths closer to the experimental values, and strong oscillatory strength values. This explains that fluorescent intensities are weaker in this case due to the presence of the silent Form A in the equilibrium. To understand the process more deeply, we have thoroughly examined the complete photochemical process of **1a-A**, detailing both the photon emission and quenching mechanisms. In this process, photon absorption can occur in the most stable tautomer, **1a-A**, leading to the formation of the non-stationary **LE-A** state (Fig. S8). As demonstrated earlier, the molecule can then dissipate energy through a TICT process, relaxing to **S1(opt)-A**. This involves the NHPh group rotating out of the plane and becoming perpendicular to the tricyclic system. The subsequent non-radiative process returns to ground state, resulting in quenching, due to the quasi zero oscillatory strength (f = 0.001, see Fig S9A). However, since the energy level of **LE-B** is comparable to that of **LE-A**, a solvent-assisted ESIPT (Excited-State Intramolecular Proton Transfer) process is possible leading to **S1(opt)-B**. From here, the most probable process, which returns to the ground state, is a radiative process, resulting in fluorescence (see Fig S9B). The ratio of the non-radiative and radiative processes determines the overall fluorescence quantum yield. Figs. S9A–C illustrate the molecular orbital (MO) representation of the excitation and emission process of **1a-A, 1a-B ,** computed at B3LYP/6-31G(d,p). In all cases, the most probable S_0→S_1 transition upon the electronic excitation occur from HOMO (75) to LUMO (76) with good oscillatory strengths (*f*). From here, the emission relies on the LUMO → HOMO transition. The transition probability represented by the oscillatory strength in the case of **1a-A** nearly zero, due to the non-overlapping HOMO and LUMO in the **S1(opt)-A** structure. This large deviation in the MOs can be attributed to the above mentioned TICT process. In the case of **1a-B** the LUMO to HOMO transition and so the emission is predicted with high probability.

Table S4. Computed energies (ΔH), excitation and emission wavelengths (λ_{ex}, λ_{em}), molar extinction coefficients (ε) and oscillatory strengths (*f*) of species representing compounds **1a-7a** at (B3LYP/6-31G(d,p)[PCM(water)]).

Cpd.	R	Species	ΔH[a]/ (kJ mol^{-1})	λ_{ex} / nm	f	ε / M^{-1} cm^{-1}	λ_{em} / nm	f
1a	H	A	0.0	382	0.890	36850	1100	0
		B	12.8	377	0.936	40180	449	0.827
		C	47.7	404	0.801	32550	472	0.853
		D	9.5	397	1.072	42300	503	0.915
4a	5-F	A	0.0	388	0.883	36550	1165	0
		B	6.6	384	0.930	39950	458	0.812
5a	4-F	A	0.0	382	0.838	35130	1168	0
		B	6.9	375	0.932	40200	460	0.752
6a	3-Cl	A	0.0	388	0.921	38570	1170	0
		B	6.0	383	0.957	41500	460	0.828
7a	2,3-MDO	A	0.0	402	0.764	34070	1064	0
		B	14.0	400	0.640	30500	451	0.729

[a]Enthalpy values relative to the most stable species

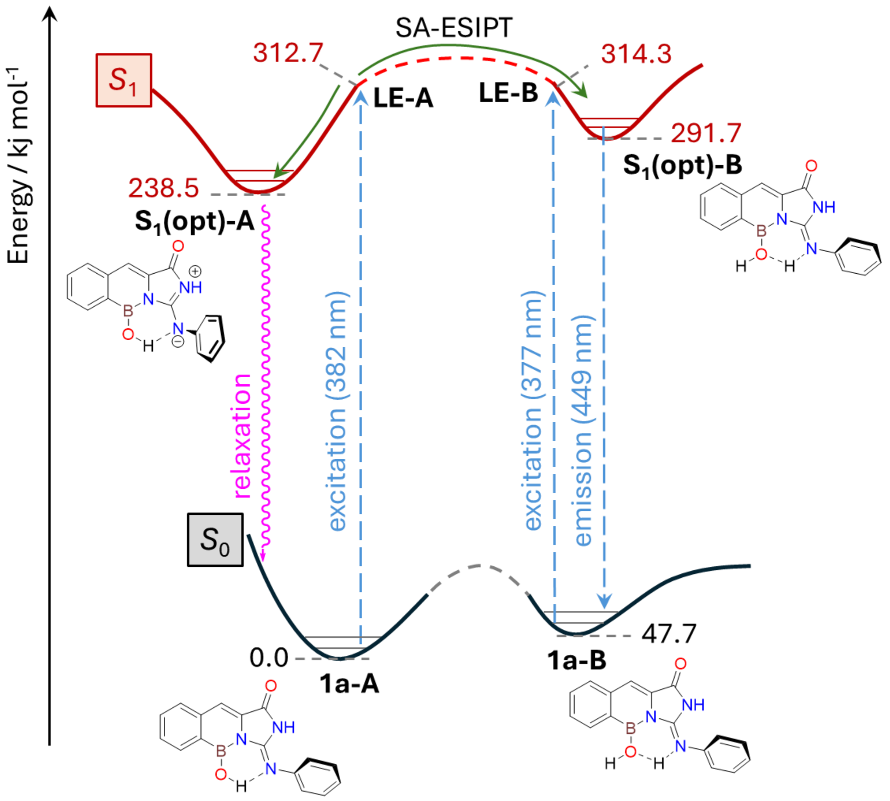

Fig S8. Energy representation of the photochemical mechanism of **1a**. SA-ESIPT = Solvent assisted excited state intramolecular proton transfer.

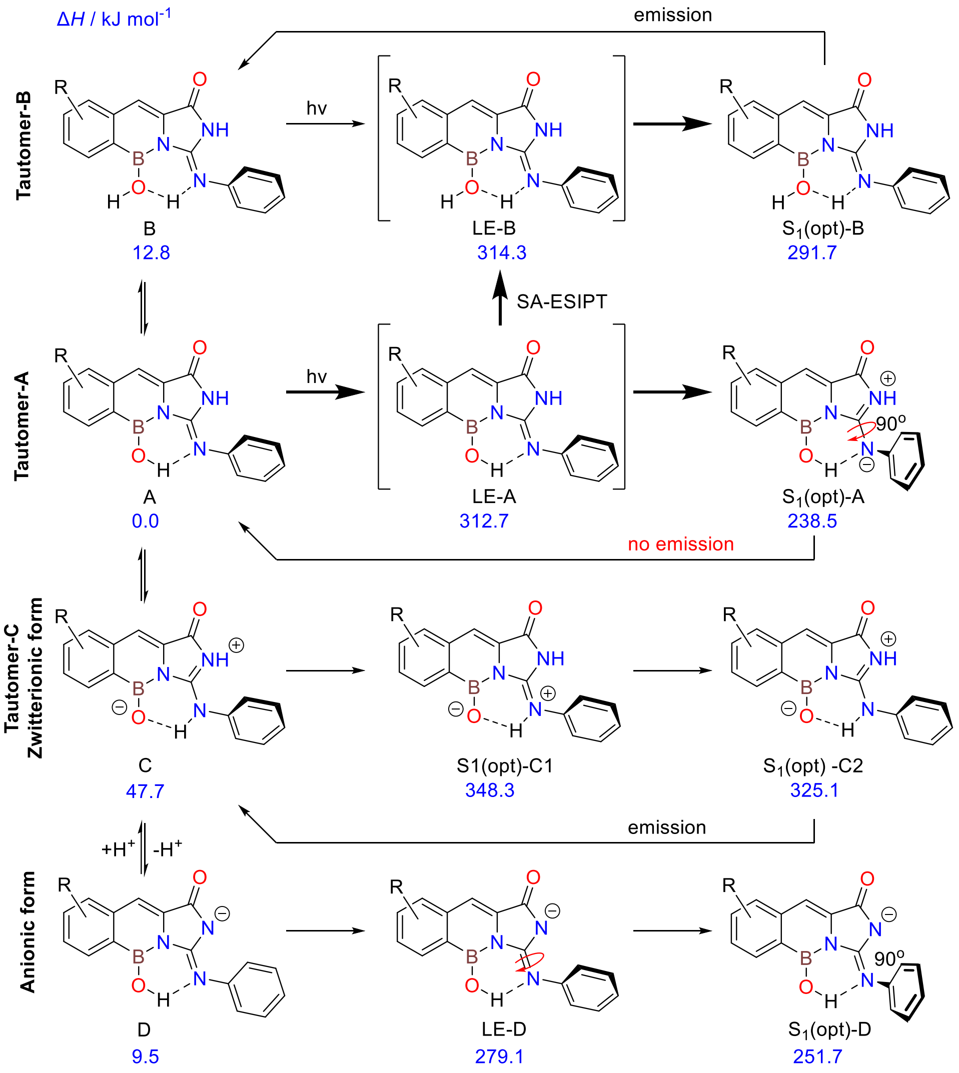

Scheme S2. The photochemical mechanism of the excitation and emission of compounds **1a-7a** (B3LYP/6-31G(d,p)[PCM(water)]). Calculated enthalpy values (kJ mol^{-1}) are given in blue for compound **1a**. Bold arrows represent the most probable processes. The LE-A corresponds to a branching point to S_1(opt)-A as well as to LE-B via a solvent assisted excited state intramolecular proton transfer. (SA-ESIPT). LE = locally excited, non-stationary state.

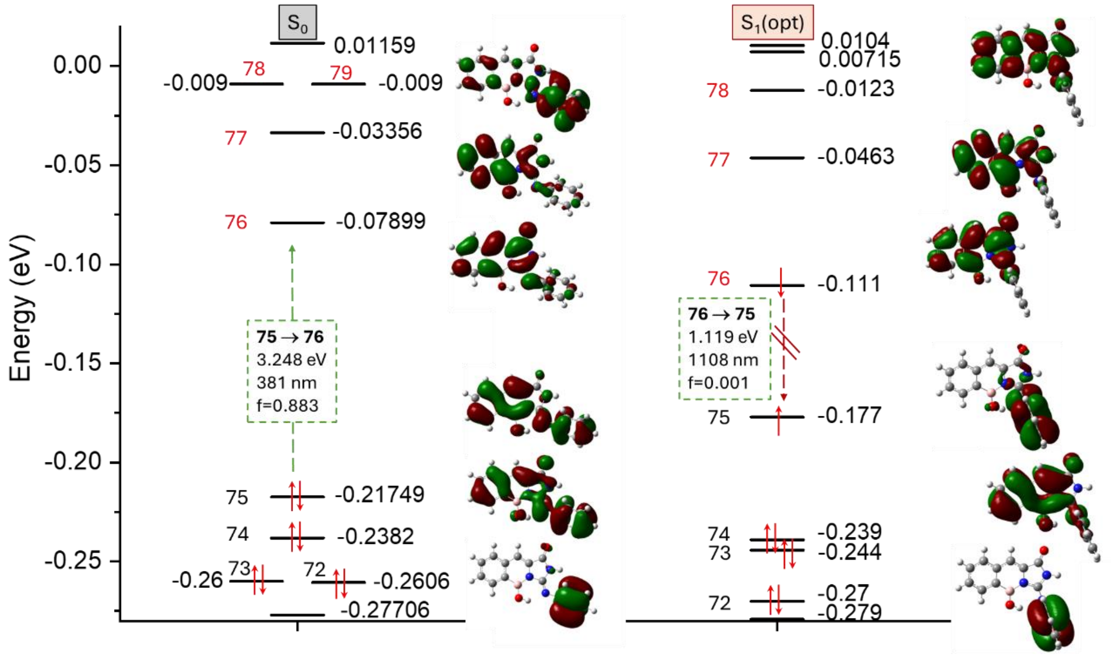

Fig S9A. Molecular orbital representation of the excitation and emission process of **1a-A**, computed at B3LYP/6-31G(d,p). The most probable S0→S1 transition occur from HOMO (75) to LUMO (76). The oscillatory strength of the emission is nearly zero due to the not overlapping molecular orbitals.

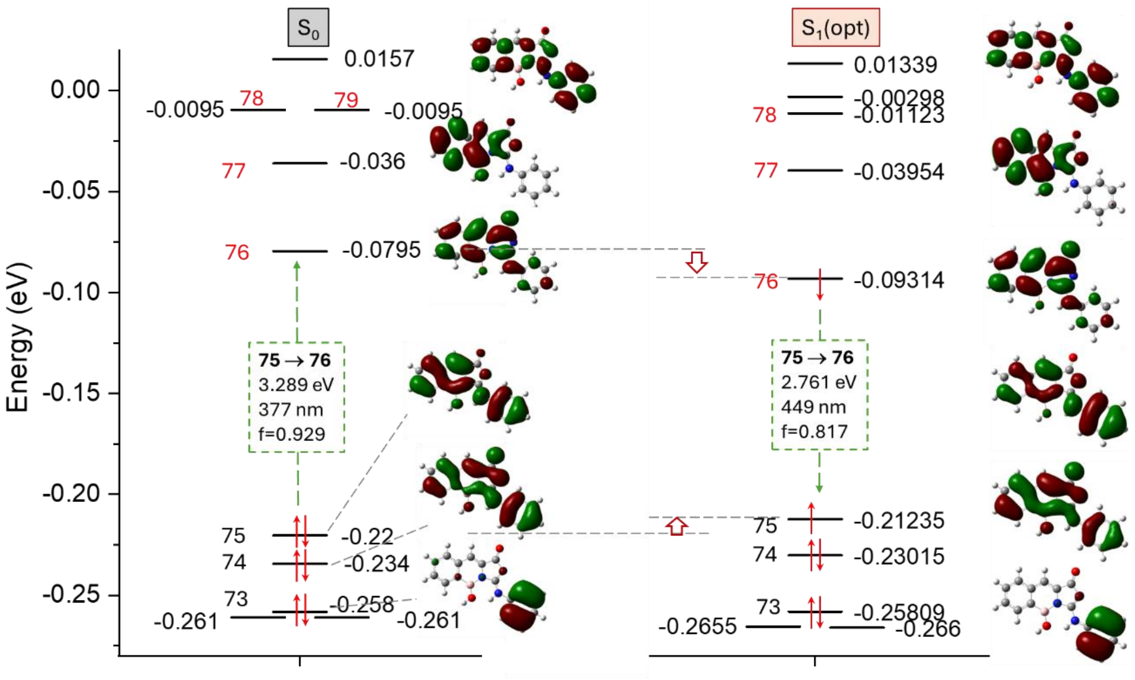

Fig S9B. Molecular orbital representation of the excitation and emission process of **1a-B,** computed at B3LYP/6-31G(d,p). The most probable $S_0 \rightarrow S_1$ transition occur from HOMO (75) to LUMO (76). The emission is predicted to occur from LUMO to HOMO with good probability.

The second group of compounds (**1b–7b**, see Scheme S1 bottom, S3) show a much simpler photochemical mechanism. The structure is represented by a single stable tautomer, whereas the others have significantly higher enthalpy levels. The calculated excitation wavelengths λ_{ex} at S_1(LE) are close to the measured values. The excited state has a more planarized structure in this case, which is almost unchanged compared to the initial one, not causing quenching effect. The calculated emitted wavelengths agree well with the observed values. Fig. S9C shows, that both the HOMO(71) – LUMO(72) and the LUMO (72) to HOMO (71) transition is allowed and favoured. This can be explained by the symmetry of the orbitals, which allow the strong fluorescence.

Table S5. Computed energies (ΔH), excitation and emission wavelengths (λ_{ex}, λ_{em}), molar extinction coefficients (ε) and oscillatory strengths (f) of species representing compounds **1a-7a** at (B3LYP/6-31G(d,p)[PCM(water)]).

**Cpd**	**R**	**Species**	**ΔH^a/(kJ mol^{-1})**	**λ_{ex} / nm**	***f***	**ε / M^{-1} cm^{-1}**	**λ_{em} / nm**	***f***
1b	H	A	0.0	400	0.605	26150	490	0.561
		B	52.9	448	0.620		538	0.267
		C	34.9	443	0.635		536	0.689
		D	51.9	440	0.557		531	0.252
2b	4-OMe	A	0.0	395	0.462	28400	485	0.537
		B	55.5	411	0.754		595	0.107
		C	36.8	446	0.436		550	0.193
		D	35.1	427	0.798		460	0.764
6b	3-Cl	A	0.0	406	0.658	28400	492	0.620
		B	47.5	375	0.932		460	0.752
		C	23.9	442	0.617		532	0.283
		D	43.7	457	0.665		549	0.625
7b	2,3-MDO	A	0.0	455	0.350	15030	544	0.359
		B	49.3	518	0.234		601	0.329
		C	33.5	479	0.543		555	0.317
		D	40.5	404	0.526		435	0.536

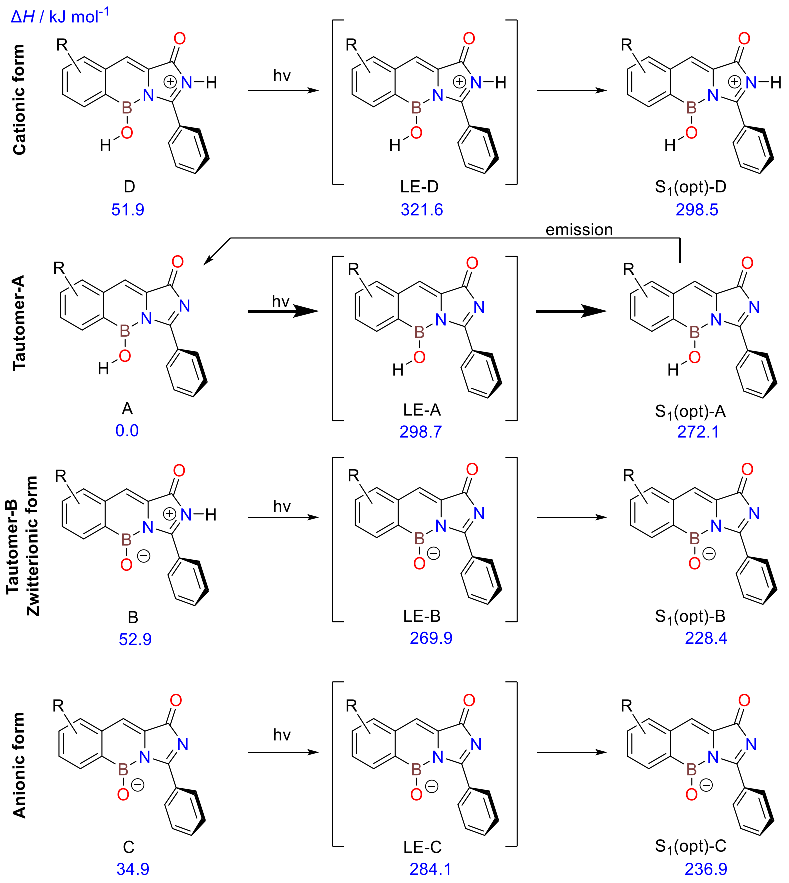

Scheme S3 Optimized structures in the equilibrium representing compounds **1b-7b** (B3LYP/6-31G(d,p) [PCM(water)]). Calculated enthalpy values (kJ mol[-1]) are given in blue for compound **1b**. Bold arrows represent the most probable processes.

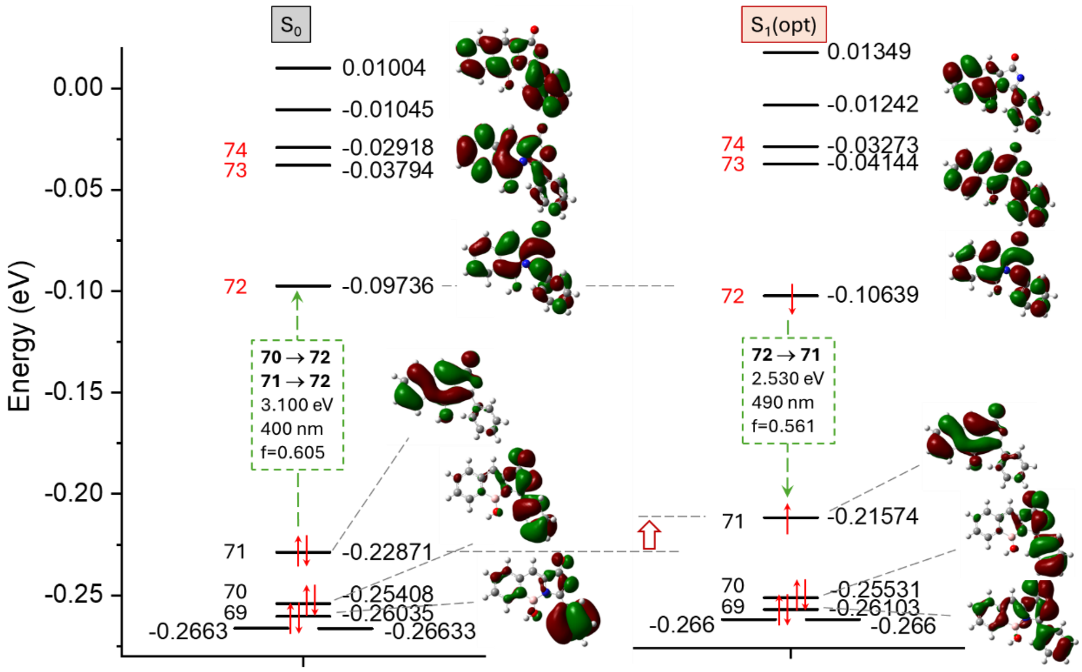

Fig S9C. Molecular orbital representation of the excitation and emission process of **1b,** computed at B3LYP/6-31G(d,p). The most probable $S_0 \rightarrow S_1$ transition occur from HOMO–1 (70) and HOMO (71) to LUMO (72). The emission is predicted to occur from LUMO to HOMO with medium probability.

The structure of the free state and the Zn^{2+} complexes were also modelled similarly (Fig S8). In the free state, a strong hydrogen bond is observed between one of the pyridyl *N*s and the B-OH group, stabilizing the structure. This explains the weak zinc binding of the compound, moreover, it also causes a splitting of the methylene hydrogens of the pycolyl groups in the NMR spectra (Fig S68-74) of **8**. The two methylene groups have a distinctly different chemical shift and the hydrogens on the same methylene also differ resulting in different chemical shifts and an observed J_2 coupling. Therefore, the computationally proposed structure is in very good agreement with the experimental results. The complex forms are probably pentadentate with a water molecule also coordinating to Zn^{2+}. Here we propose four similar structures that can represent reality.

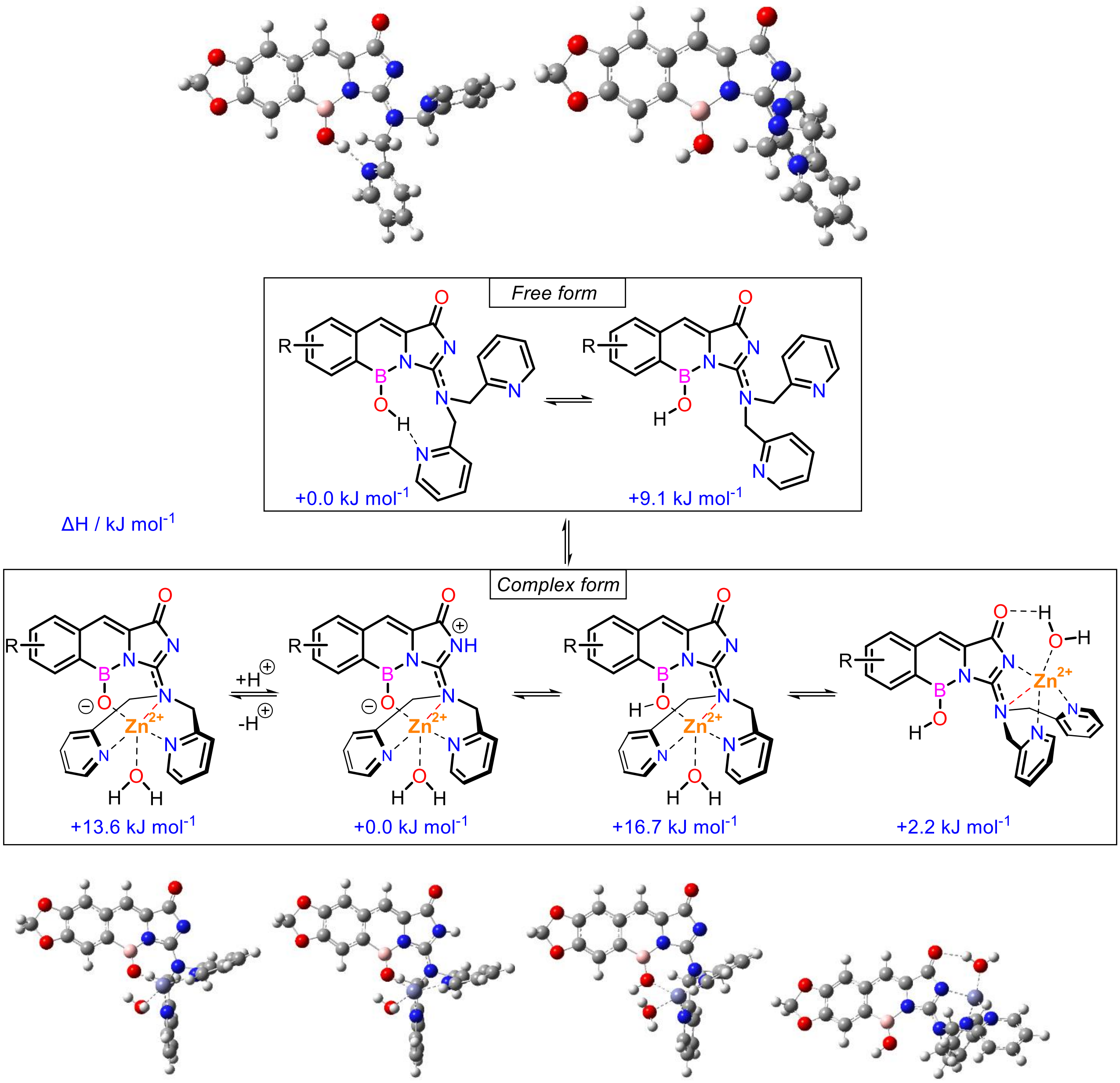

Fig S10. Optimized structures of the free probes and the Zn^{2+} complexes (B3LYP/6-31G(d,p)[PCM(water)]). Calculated enthalpy values (kJ mol^{-1}) are given in blue for compound **9**.

Table S6. Computed energies (*E*), zero point energies (*ZPE*), internal energies (*U*), enthalpies (*H*) and Gibbs free energies (*G*) given in Hartree as well as entropies (*S*) given in J mol^{-1} K^{-1} at B3LYPD3/6-31G(d,p)[PCM(water)] basis set with the consideration of PCM solvent method using the parameter set of water for small molecules and ions used for the calculations.

	Molecule	*E* / J mol^{-1} K^{-1}	*ZPE* / J mol^{-1} K^{-1}	*U* / J mol^{-1} K^{-1}	*H* / J mol^{-1} K^{-1}	*G* / J mol^{-1} K^{-1}	*S* / J mol^{-1} K^{-1}
	000aaa_5_H2O_+2H2O_B3lyp631dp_PCMw.log	-535.08700997	-534.912331	-534.895555	-534.894611	-534.957949	133.306
	000aaa_5_H2O_+3H2O_b3lyp631dp_PCMw.log	-611.52813700	-611.328949	-611.309255	-611.308311	-611.380614	152.175
	000aab_5_H2O_+3H2O+H+_b3lyp631dp_PCMw.log	-611.98027874	-611.769760	-611.750528	-611.749584	-611.819331	146.795
	000bab_5_H2O_b3lyp631dp_PCMw_f.log	-382.19412786	-382.069270	-382.057165	-382.056220	-382.106229	105.251
	000cab_5_H2O_-H+_b3lyp631dp_PCMw.log	-381.69437035	-381.584737	-381.574452	-381.573507	-381.619785	97.399

Table S7 Computed energies (*E*), zero point energies (*ZPE*), internal energies (*U*), enthalpies (*H*) and Gibbs free energies (*G*) given in Hartree as well as entropies (*S*) given in J mol^{-1} K^{-1} at B3LYPD3/6-31G(d,p)[PCM(water)] basis set with the consideration of PCM solvent method using the parameter set of water for compounds study of 1a-7a.

		Molecule	*E* / J mol^{-1} K^{-1}	*ZPE* / J mol^{-1} K^{-1}	*U* / J mol^{-1} K^{-1}	*H* / J mol^{-1} K^{-1}	*G* / J mol^{-1} K^{-1}	*S* / J mol^{-1} K^{-1}
**1a**	A	01aaa_FL_ClassIII_NHPh_alap_B3LYP631dp_PCMw.log	-956.66579006	-956.406803	-956.390305	-956.389361	-956.451802	131.418
	B	01bba_FL_ClassIII_NHPh_alap_B3LYP631dp_PCMw.log	-956.66119066	-956.402152	-956.385419	-956.384475	-956.447940	133.573
	C1	01caa_FL_ClassIII_NHPh_alap_ZW_B3LYP631dp_PCMw.log	-956.64581993	-956.388706	-956.372113	-956.371168	-956.433966	132.169
	C2	01daa_FL_ClassIII_NHPh_alap_ZW2_B3LYP631dp_PCMw.log	-956.64545777	-956.386659	-956.370172	-956.369227	-956.432274	132.692
	D	01dbb_FL_ClassIII_NHPh_alap-H+_B3LYP631dp_PCMw.log	-956.18659895	-955.940774	-955.924681	-955.923737	-955.985224	129.410
	A	01faa_FL_ClassIII_NHPh_alap_HOH_B3LYP631dp_PCMw.log	-1033.10710924	-1032.823182	-1032.803814	-1032.802869	-1032.871810	145.097
	B	01fba_FL_ClassIII_NHPh_HOH_alap_B3LYP631dp_PCMw.log	-1033.10479135	-1032.821399	-1032.801366	-1032.800422	-1032.871834	150.299
**4a**	A	02aaa_FL_ClassIII_5F_NHPh_alap_B3LYP631dp_PCMw.log	-1055.89735111	-1055.646664	-1055.629274	-1055.628330	-1055.692838	135.769
	B	02baa_FL_ClassIII_5F_NHPh_alap_B3LYP631dp_PCMw.log	-1055.89251239	-1055.641655	-1055.624092	-1055.623148	-1055.688438	137.415
**5a**	A	03aaa_FL_ClassIII_4F_NHPh_alap_B3LYP631dp631dp_PCMw.log	-1055.89771693	-1055.647127	-1055.629742	-1055.628798	-1055.693349	135.860
	B	03baa_FL_ClassIII_4F_NHPh_alap_B3LYP631dp_PCMw.log	-1055.89303895	-1055.642402	-1055.624770	-1055.623826	-1055.689439	138.094
	D	03caa_FL_ClassIII_4F_NHPh_alap_-H+_B3LYP631dp631dp_PCMw.log	-1055.41952014	-1055.182097	-1055.165109	-1055.164164	-1055.227783	133.896
**6a**	A	04aaa_FL_ClassIII_3Cl_NHPh_alap_B3LYP631dp_PCMw.log	-1416.26033032	-1416.011266	-1415.993436	-1415.992492	-1416.058510	138.946
	B	04baa_FL_ClassIII_3Cl_NHPh_alap_B3LYP631dp_PCMw.log	-1416.25504134	-1416.005638	-1415.987737	-1415.986793	-1416.052833	138.994
	D	04caa_FL_ClassIII_3Cl_NHPh_alap_-H+_B3LYP631dp_PCMw.log	-1415.78254177	-1415.546600	-1415.529137	-1415.528193	-1415.594204	138.932
**7a**	A	05aaa_FL_ClassIII_23MDOl_NHPh_alap_B3LYP631dp_PCMw.log	-1145.19792362	-1144.923804	-1144.904813	-1144.903869	-1144.972863	145.210
	B	05baa_FL_ClassIII_23MDOl_NHPh_alap_B3LYP631dp_PCMw.log	-1145.19293297	-1144.918624	-1144.899491	-1144.898547	-1144.967971	146.115
	D	05caa_FL_ClassIII_23MDOl_NHPh_alap_-H+_B3LYP631dp_PCMw.log	-1144.71788467	-1144.456849	-1144.438297	-1144.437353	-1144.505343	143.098
	A+H_2O^a	05aaa_FL_ClassIII_23MDOl_NHPh_alap_HOH_B3LYP631dp_PCMw.log	-1221.63926466	-1221.340246	-1221.318344	-1221.317400	-1221.393317	159.780
	B+H_2O^a	05baa_FL_ClassIII_23MDOl_NHPh_alap_HOH_B3LYP631dp_PCMw.log	-1221.63676417	-1221.338321	-1221.316628	-1221.315684	-1221.391678	159.943

[a]includes explicit water molecule

Table S8 Computed energies (*E*), zero point energies (*ZPE*), internal energies (*U*), enthalpies (*H*) and Gibbs free energies (*G*) given in Hartree as well as entropies (*S*) given in J mol^{-1} K^{-1} at B3LYPD3/6-31G(d,p)[PCM(water)] basis set with the consideration of PCM solvent method using the parameter set of water for compounds study of 1b-7b.

		**Molecule**	***E* / J mol^{-1} K^{-1}**	***ZPE* / J mol^{-1} K^{-1}**	***U* / J mol^{-1} K^{-1}**	***H* / J mol^{-1} K^{-1}**	***G* / J mol^{-1} K^{-1}**	***S* / J mol^{-1} K^{-1}**
**1b**	**A**	101aaa_FL_ClassIII_Ph_alap_B3LYP631dp_PCMw.log	-901.28238958	-901.040773	-901.025212	-901.024268	-901.084321	126.393
	**B**	101baa_FL_ClassIII_Ph_alap_ZW_B3LYP631dp_PCMw.log	-901.26258607	-901.020673	-901.005057	-901.004112	-901.064538	127.175
	**C**	101caa_FL_ClassIII_Ph_alap-H+_B3LYP631dp_PCMw.log	-900.79394675	-900.565155	-900.549908	-900.548964	-900.609574	127.565
	**D**	101daa_FL_ClassIII_Ph_alap+H+_B3LYP631dp_PCMw.log	-901.73309690	-901.478191	-901.462446	-901.461501	-901.521837	126.986
	**D2**	102aaa_FL_ClassIII_Ph_alap_+H+_B3LYP631dp_PCMw.log	-901.73309695	-901.478191	-901.462446	-901.461502	-901.521830	126.972
	**C2**	103aaa_FL_ClassIII_Ph_alap_-H+_B3LYP631dp_PCMw.log	-900.79394675	-900.565156	-900.549908	-900.548964	-900.609597	127.613
**2b**	**A**	104aaa_FL_ClassIII_3Cl_Ph_alap_B3LYP631dp_PCMw.log	-1360.87606043	-1360.644240	-1360.627393	-1360.626449	-1360.689874	133.489
	**B**	104baa_FL_ClassIII_3Cl_Ph_alap_B3LYP631dp_PCMw.log	-1360.85793247	-1360.625819	-1360.608929	-1360.607984	-1360.671794	134.299
	**C**	104caa_FL_ClassIII_3Cl_Ph_alap_-H+_B3LYP631dp_PCMw.log	-1360.39098360	-1360.171940	-1360.155410	-1360.154466	-1360.217917	133.544
	**D**	104daa_FL_ClassIII_3Cl_Ph_alap_+H+_B3LYP631dp_PCMw.log	-1361.32526765	-1361.080294	-1361.063258	-1361.062314	-1361.126016	134.073
**6b**	**A**	105aaa_FL_ClassIII_23MDO_Ph_alap_B3LYP631dp_PCMw.log	-1089.81424481	-1089.557438	-1089.539408	-1089.538464	-1089.604772	139.557
	**B**	105baa_FL_ClassIII_23MDO_Ph_alap_B3LYP631dp_PCMw.log	-1089.79543729	-1089.538391	-1089.520289	-1089.519345	-1089.586523	141.389
	**C**	105caa_FL_ClassIII_23MDO_Ph_alap_-H+_B3LYP631dp_PCMw.log	-1089.32551555	-1089.081426	-1089.063816	-1089.062872	-1089.128515	138.158
	**D**	105daa_FL_ClassIII_23MDO_Ph_alap_+H+_B3LYP631dp_PCMw.log	-1090.26625643	-1089.995996	-1089.977882	-1089.976938	-1090.042955	138.945
**7b**	**A**	106aaa_FL_ClassIII_4OMe_Ph_alap_B3LYP631dp_PCMw.log	-1015.81059789	-1015.536564	-1015.518306	-1015.517362	-1015.583561	139.326
	**B**	106baa_FL_ClassIII_4OMe_Ph_alap_B3LYP631dp_PCMw.log	-1015.78943785	-1015.515037	-1015.496777	-1015.495832	-1015.562024	139.312
	**C**	106caa_FL_ClassIII_4OMe_Ph_alap_-H+_B3LYP631dp_PCMw.log	-1015.32061137	-1015.059599	-1015.041587	-1015.040643	-1015.109518	144.959
	**D**	106daa_FL_ClassIII_4OMe_Ph_alap_+H+_B3LYP631dp_PCMw.log	-1016.26211385	-1015.974728	-1015.956288	-1015.955344	-1016.021811	139.892

S1.5.3. Theoretical results – systems chemistry analysis

1a and **1b** were analysed from the perspective of systems chemistry to understand the internal structural characteristics of the new scaffold. The parameters used are Nucleus Independent Chemical Shift (NICS, in ppm), aromaticity (AR%), amidicity (AM%), olefinicity (OL%), and imidicity (IM%). The values were calculated as reported previously. [14–17]

The open forms of **1a** and **1b** consist of two fully aromatic phenyl rings (*A* and *B*, confirmed by both NICS values and AR%) and a weakly or non-aromatic imidazolone ring (*C*, based on NICS). The closed forms of **1a** and **1b** feature a three-ring system (A'-D'-C'), where the newly formed ring D is a borazine ring situated between the aromatic ring A and ring C. For **1a** and **1b**, the AM% decrease slightly, while IM% values decrease significantly, and the OL% values increase. In **1a**, both rings C and D exhibit weak aromaticity according to NICS values, which stabilizes the structure formed in addition to a newly formed hydrogen bond. Due to this, the reaction shows exothermic character (ΔH_{RC} = -71.5 kJ mol^{-1}). In contrast, in **1b**, ring C becomes slightly anti-aromatic, and ring D is non-aromatic. This, and the lack of the possibility for intramolecular hydrogen bond formation results in a thermoneutral enthalpy for the ring closure (ΔH_{RC} = -1.1 kJ mol^{-1}). The results are summarized in Fig S11.

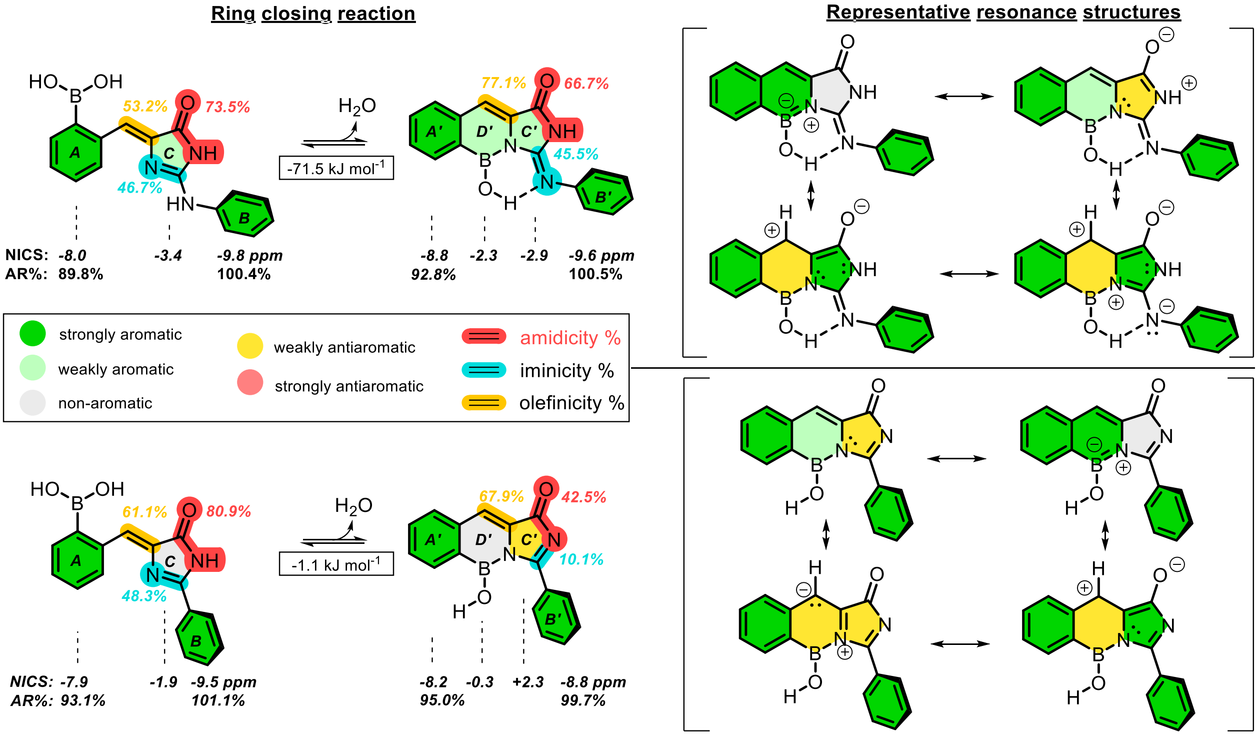

Fig S11. Summary of the systems chemistry analysis of the new scaffold (B3LYPD3/6-31G(d,p)[PCM(water)]).

S2. NMR and HRMS spectra of the prepared products

S2.1 NMR and HRMS spectra of 1a

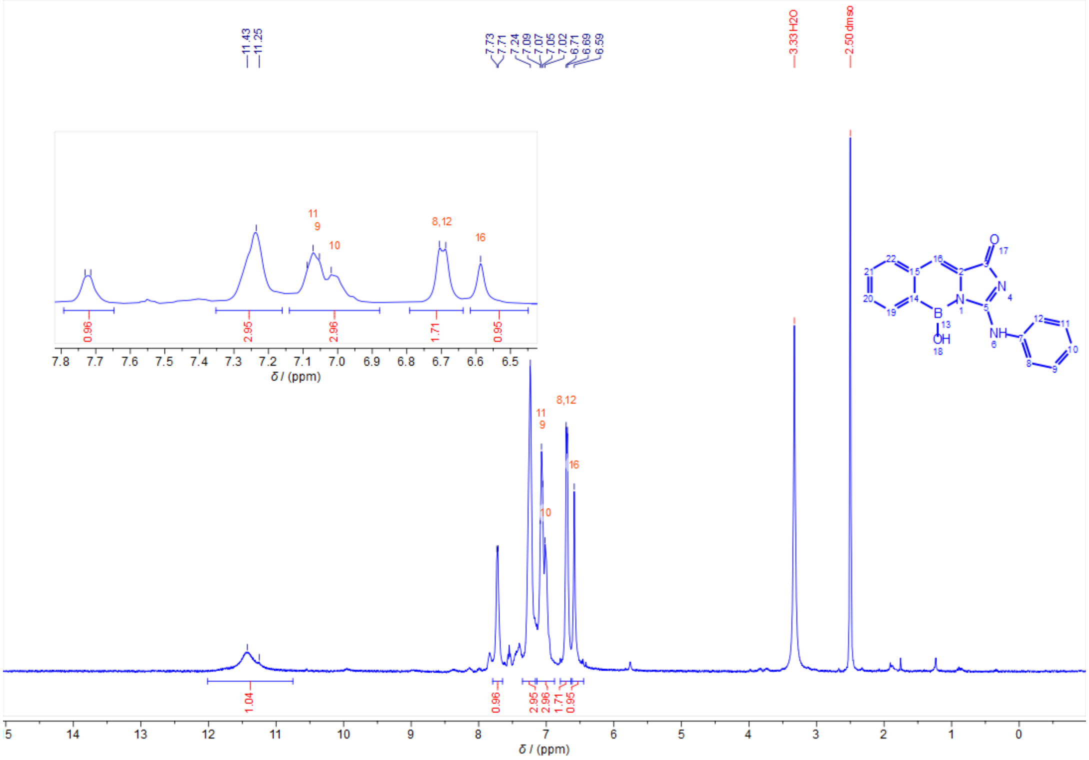

Fig S12. ^{1}H NMR spectrum of **1a** recorded at 400 MHz in DMSO-*d6*

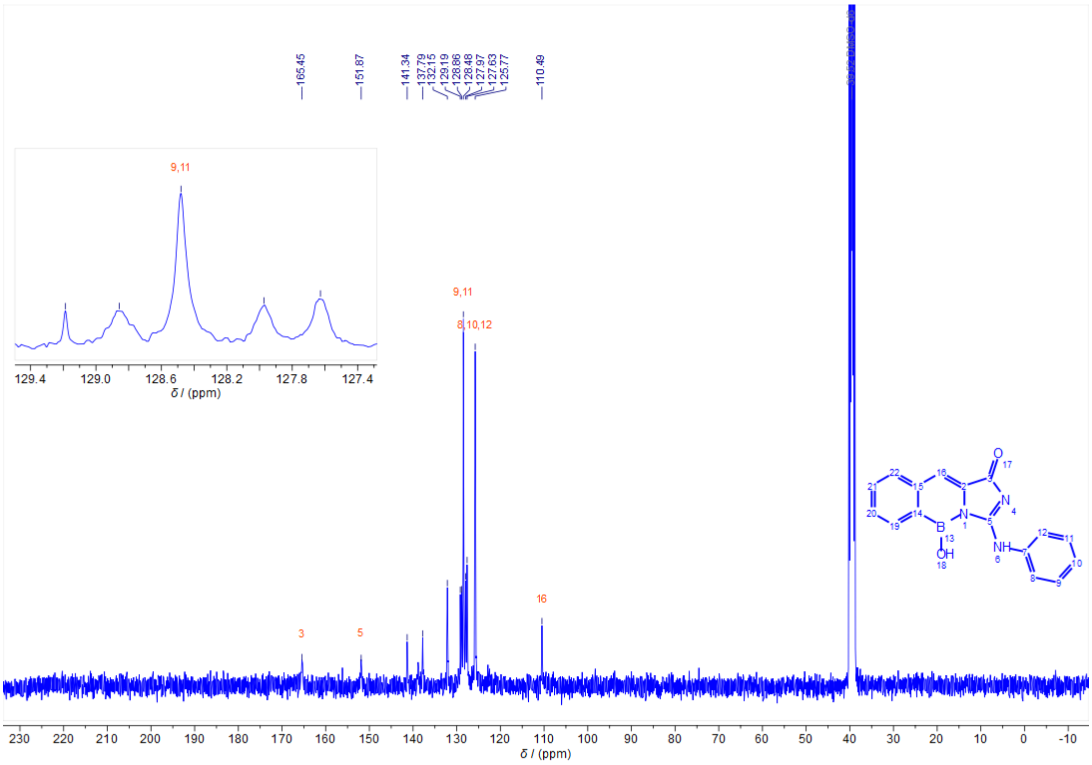

Fig S13. ^{13}C NMR spectrum of **1a** recorded at 101 MHz in DMSO-*d6*

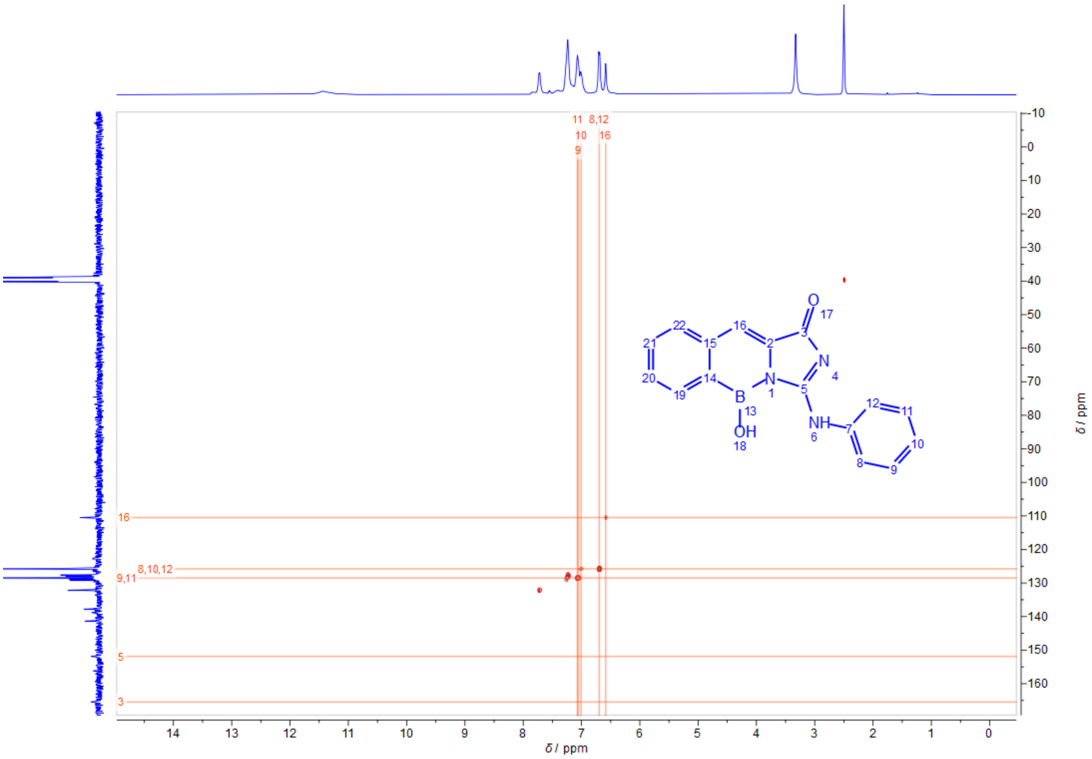

Fig S14. HSQC NMR spectrum of **1a** recorded at 400 MHz in DMSO-*d6*

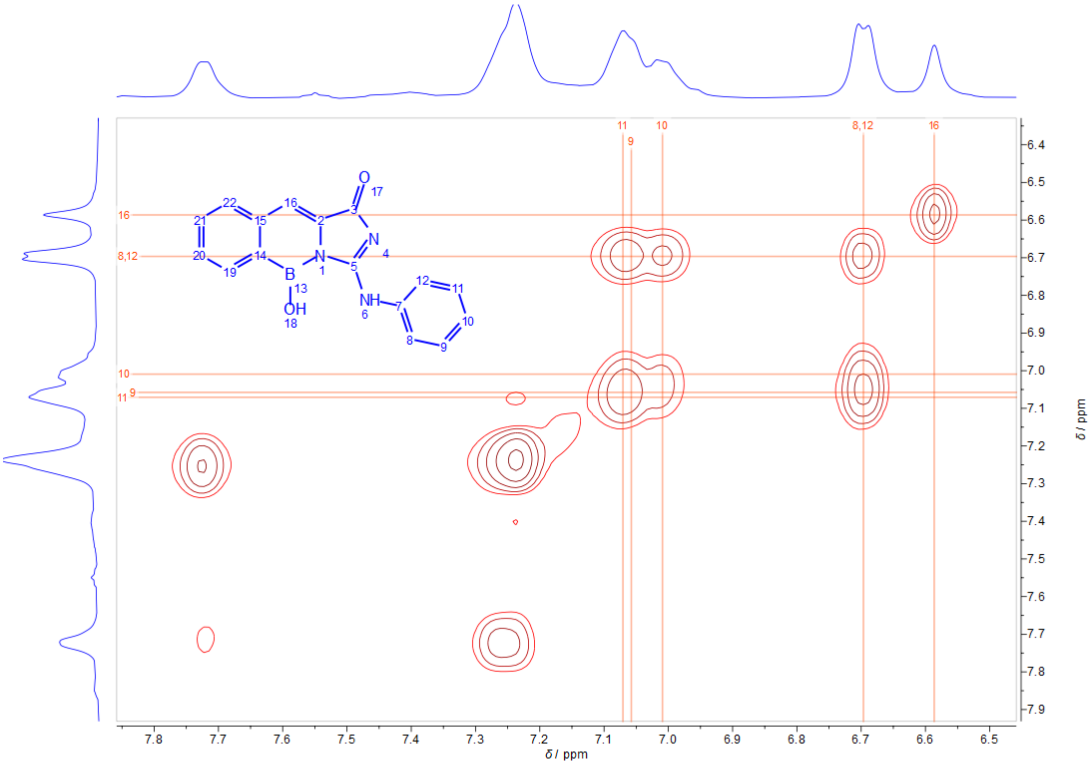

Fig S15. HMBC NMR spectrum of **1a** recorded at 400 MHz in DMSO-*d6*

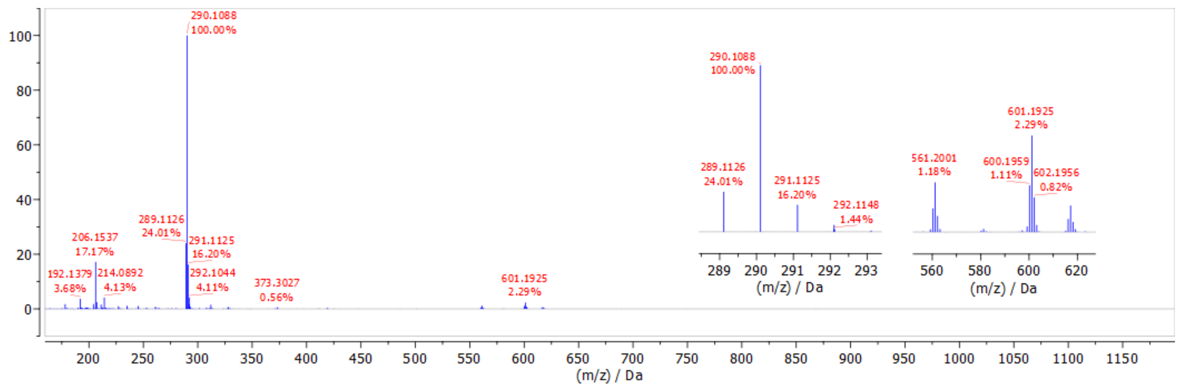

Fig S16. HRMS (ESI-Q-Orbitrap) spectrum of **1a**. *m/z*: $[M + H]^+$ Calcd for $C_{16}H_{13}O_2N_3B$ 290.1095; Found 290.1088.

S2.2 NMR and HRMS spectra of 2a

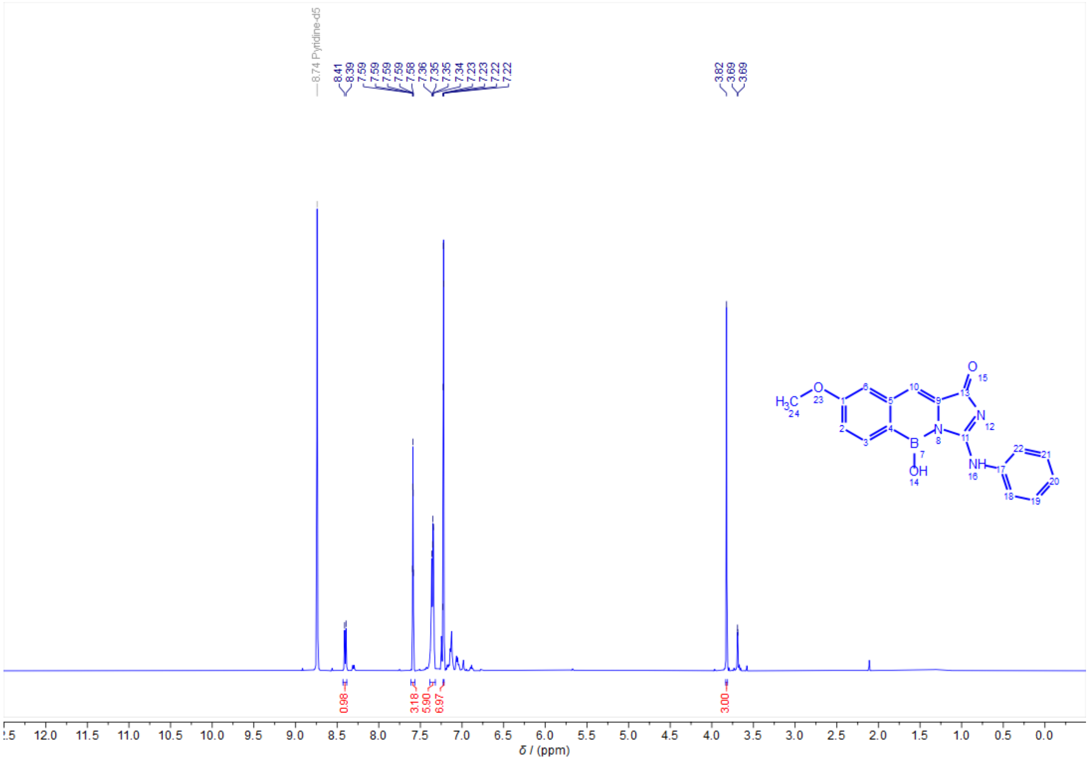

Fig S17 ^1H NMR spectrum of **2a** recorded at 400 MHz in CD_3OD

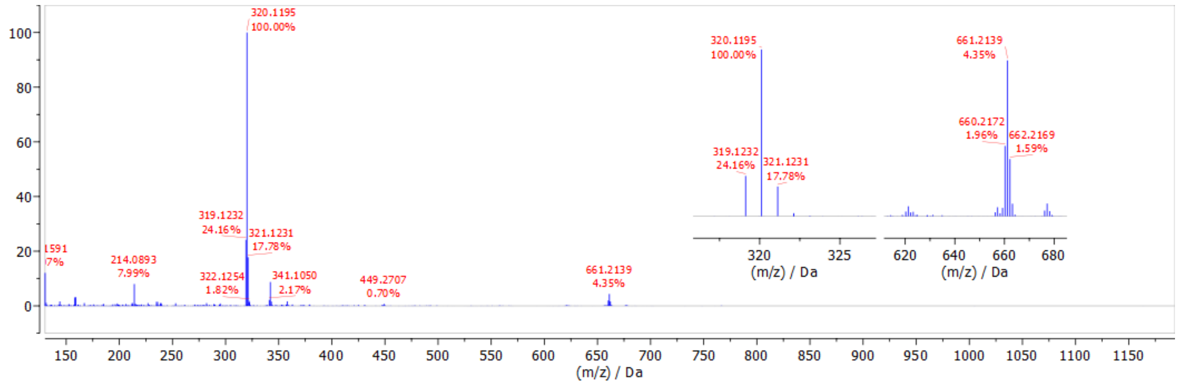

Fig S18. HRMS (ESI-Q-Orbitrap) spectrum of **2a**. *m*/*z*: $[M + H]^+$ Calcd for $C_{17}H_{15}O_3N_3B$ 320.1201; Found 320.1195.

S2.3 NMR and HRMS spectra of 2c

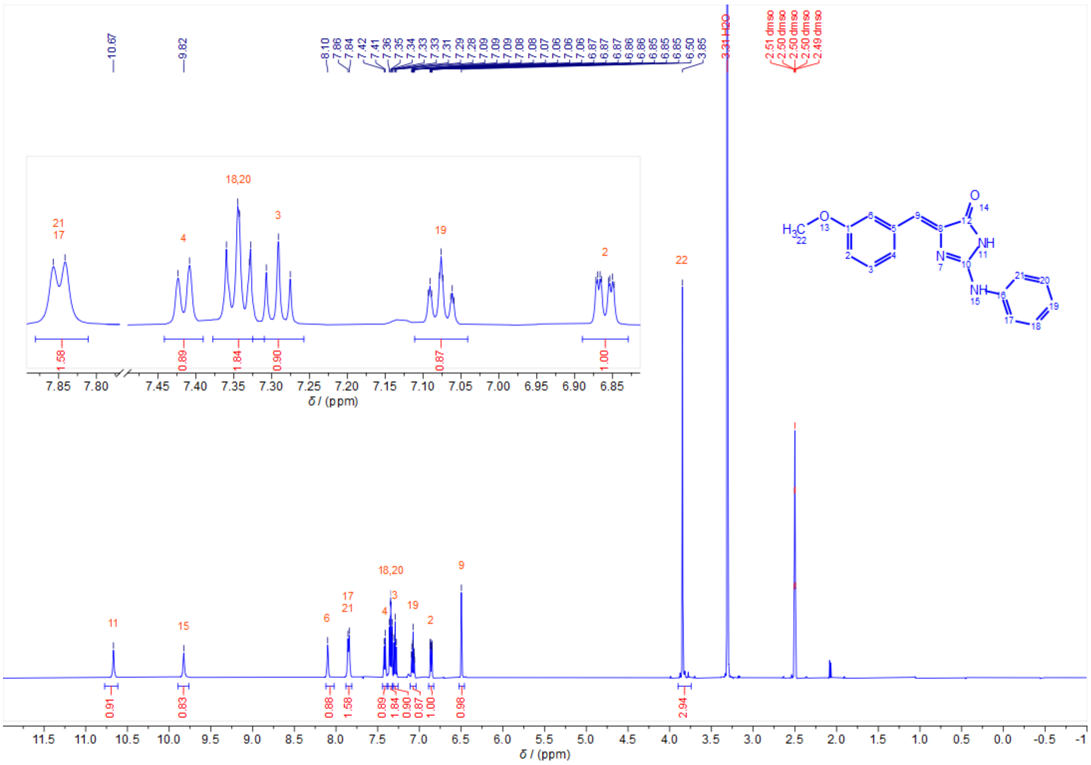

Fig S19. ^{1}H NMR spectrum of **2c** recorded at 500 MHz in DMSO-*d6*

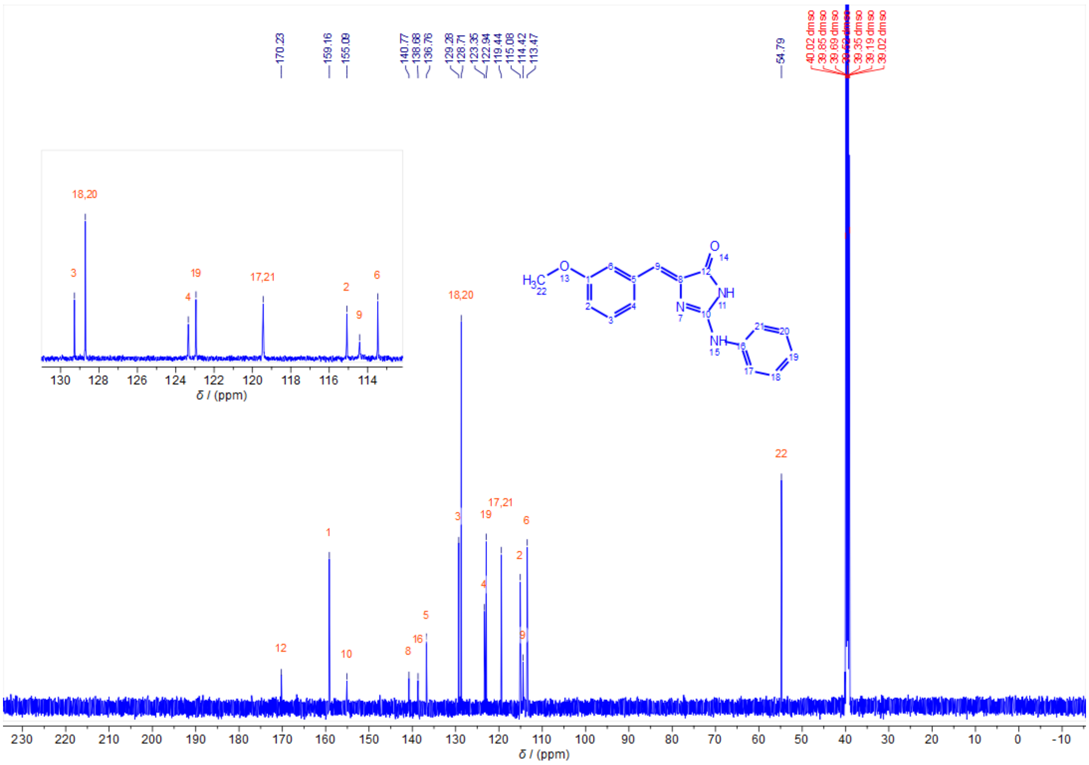

Fig S20. ^{13}C NMR spectrum of **2c** recorded at 126 MHz in DMSO-*d6*

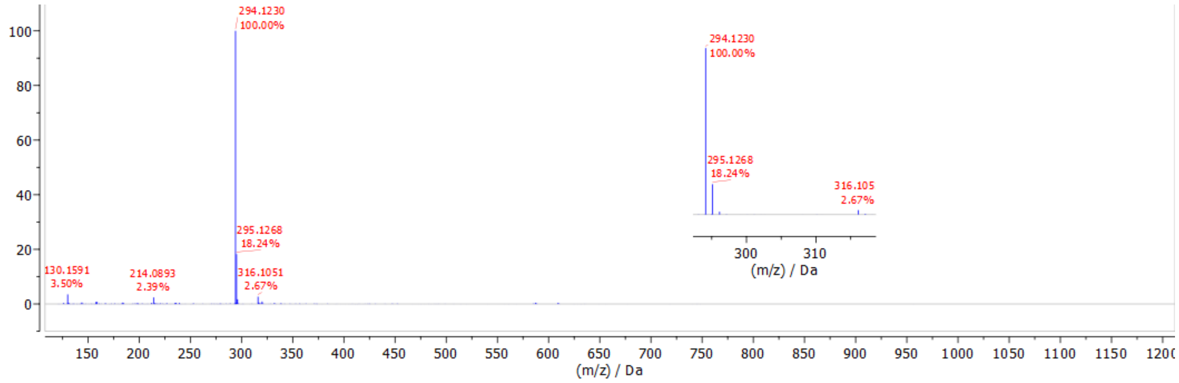

Fig S21. HRMS (ESI-Q-Orbitrap) spectrum of **2c**. *m*/*z*: $[M + H]^+$ Calcd for $C_{17}H_{16}O_2N_3$ 294.1237; Found 294.1230.

S2.4 NMR and HRMS spectra of 3c

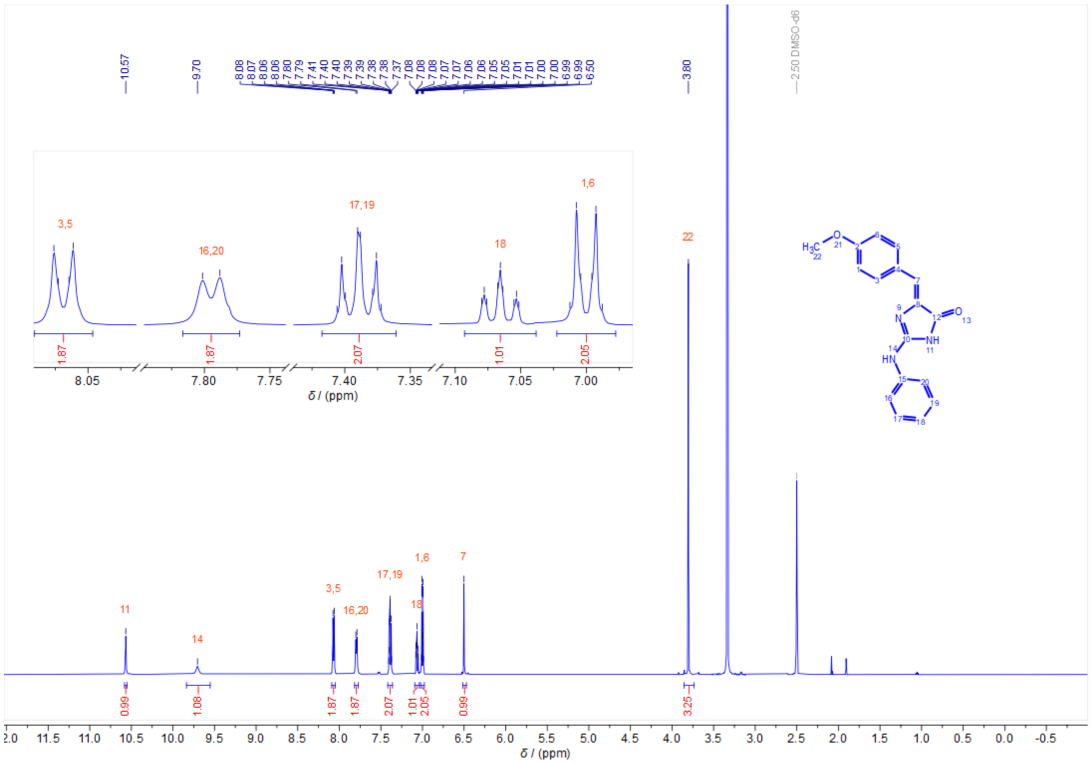

Fig S22. ^{1}H NMR spectrum of **3c** recorded at 600 MHz in DMSO-*d6*

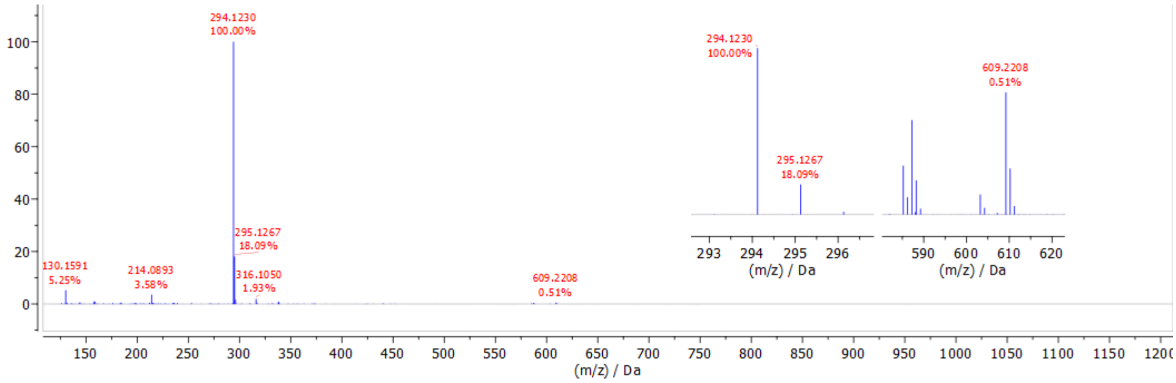

Fig S23. HRMS (ESI-Q-Orbitrap) spectrum of **3c**. *m*/*z*: $[M + H]^+$ Calcd for $C_{17}H_{16}O_2N_3$ 294.1237; Found 294.1230.

S2.5 NMR and HRMS spectra of 4a

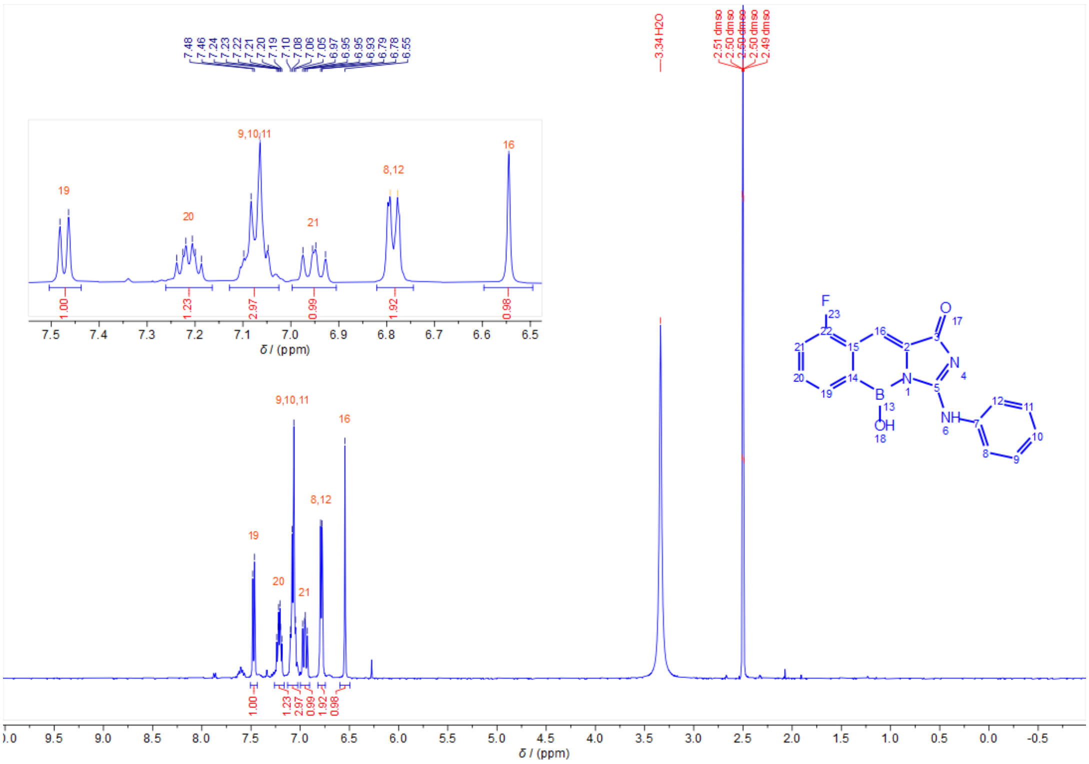

Fig S24. ^{1}H NMR spectrum of **4a** recorded at 400 MHz in DMSO-*d6*

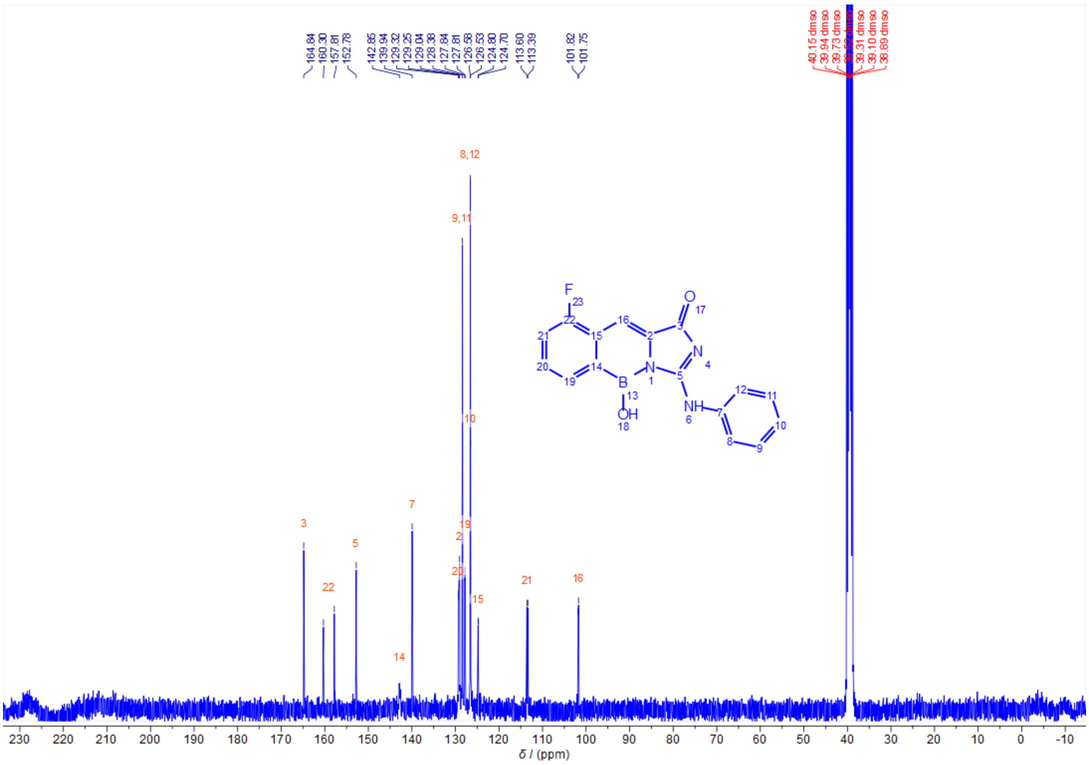

Fig S25. ^{13}C NMR spectrum of **4a** recorded at 101 MHz in DMSO-*d6*

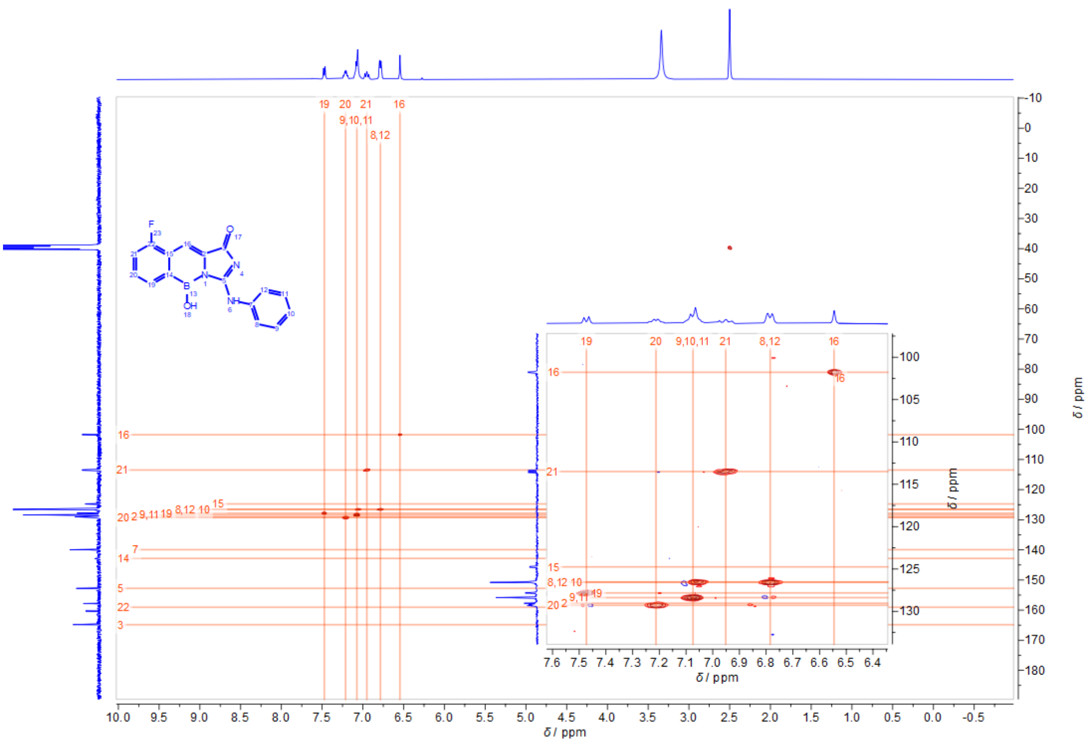

Fig S26. HSQC spectrum of **4a** recorded at 400 MHz in DMSO-*d6*.

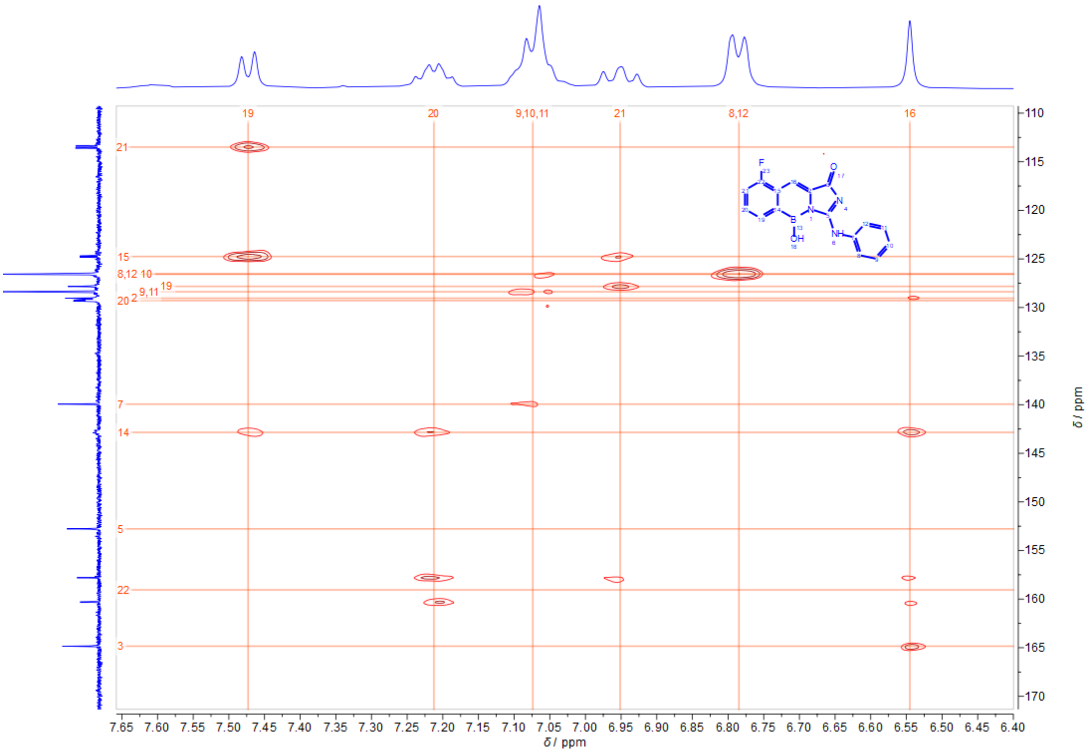

Fig S27. HMBC spectrum of **4a** recorded at 400 MHz in DMSO-*d6*

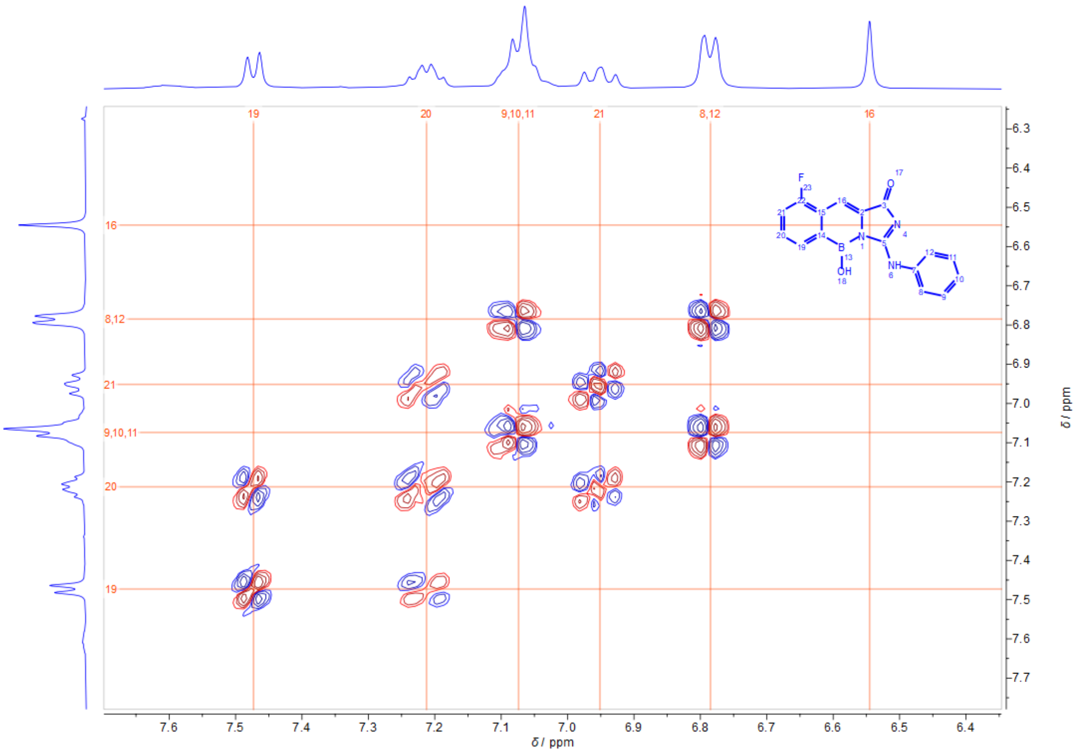

Fig S28. COSY spectrum of **4a** recorded at 400 MHz in DMSO-*d6*

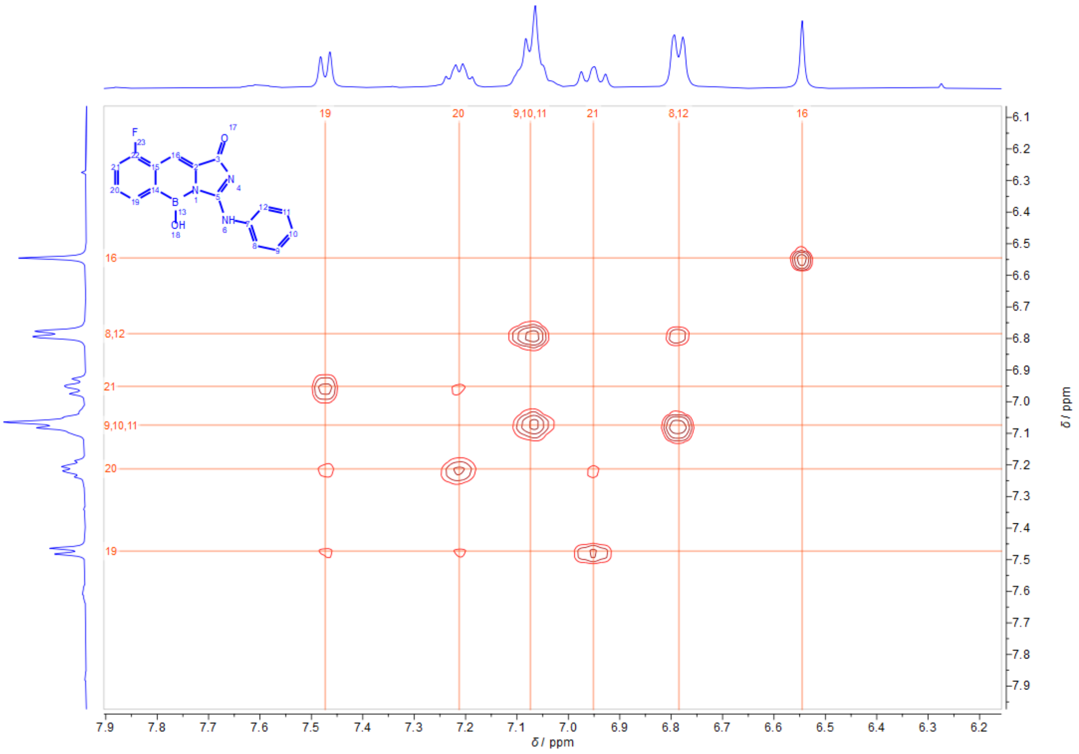

Fig S29. TOCSY spectrum of **4a** recorded at 400 MHz in DMSO-*d6*

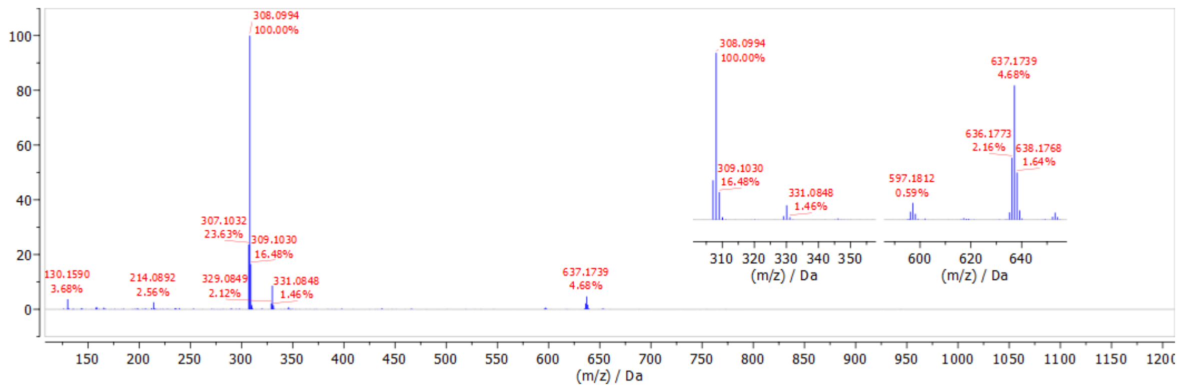

Fig S30. HRMS (ESI-Q-Orbitrap) spectrum of **4a**. *m*/*z*: $[M + H]^+$ Calcd for $C_{16}H_{12}O_2N_3BF$ 308.1001; Found 308.0994.

S2.6 NMR and HRMS spectra of 5a

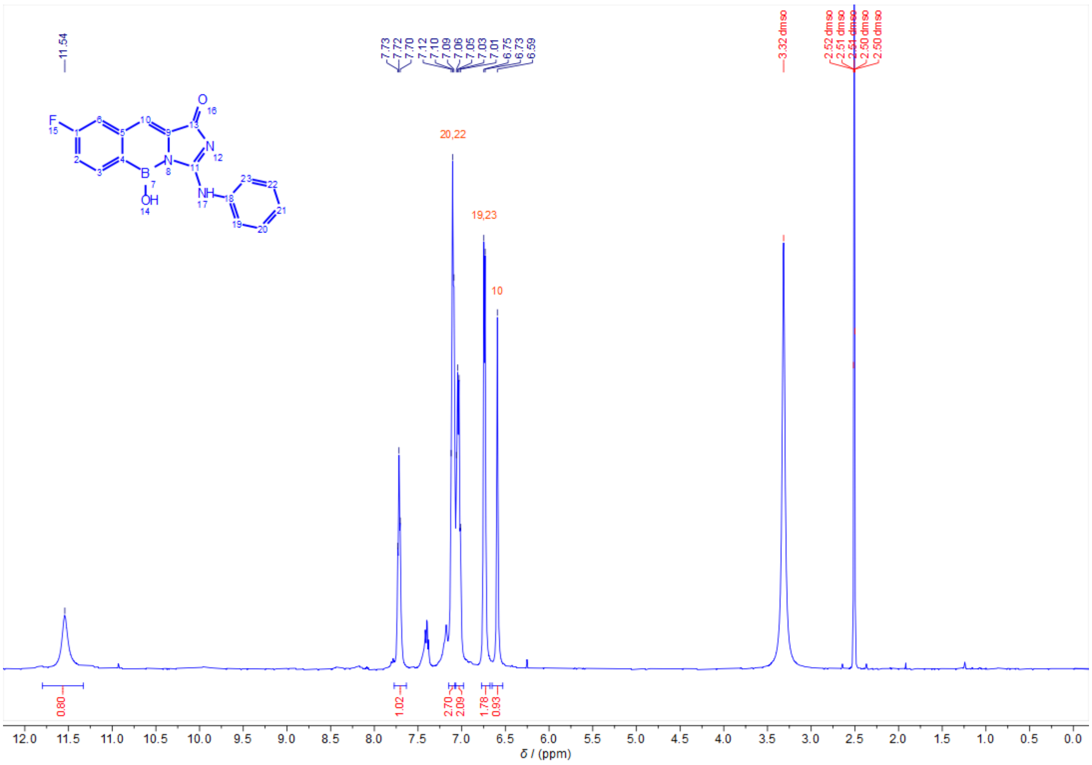

Fig S31. ^{1}H NMR spectrum of **5a** recorded at 500 MHz in DMSO-*d6*

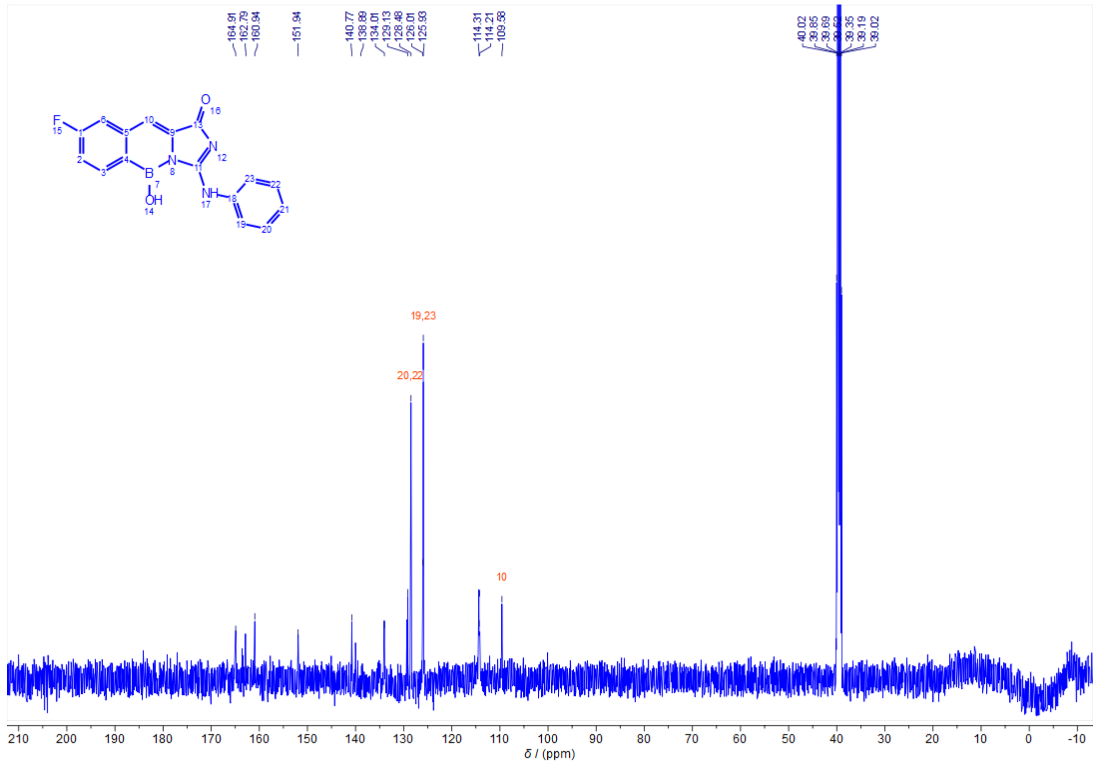

Fig S32. ^{13}C NMR spectrum of **5a** recorded at 126 MHz in DMSO-*d6*

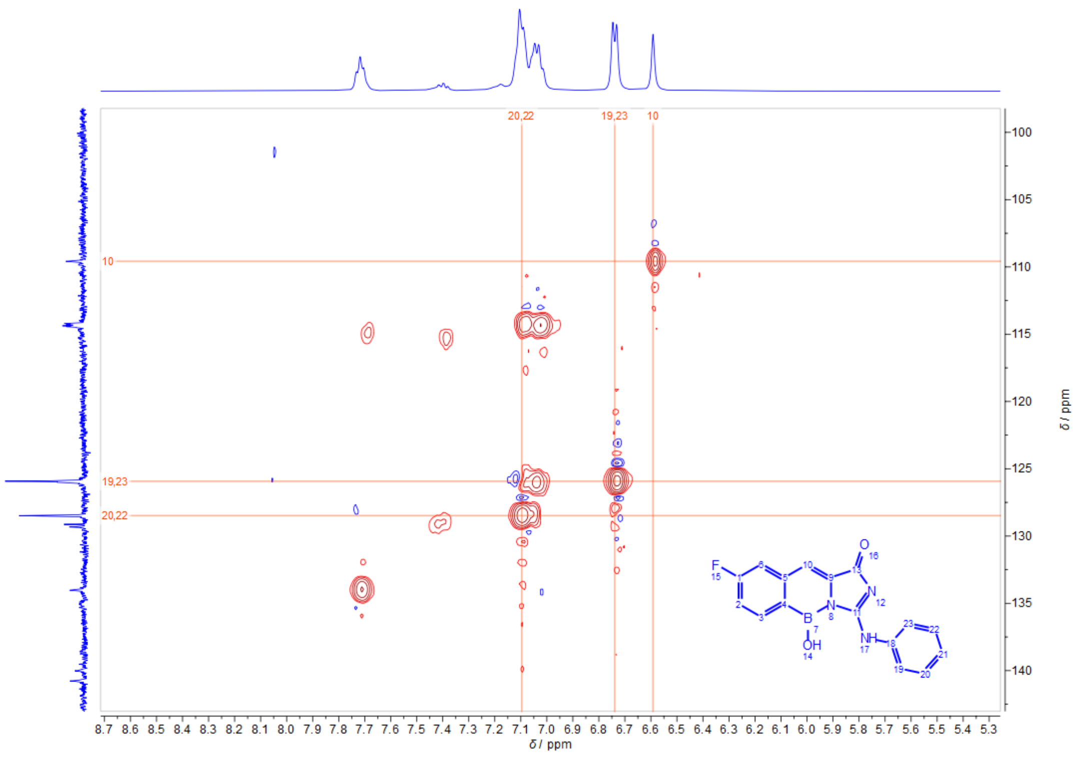

Fig S33. HSQC spectrum of **5a** recorded at 500 MHz in DMSO-*d6*

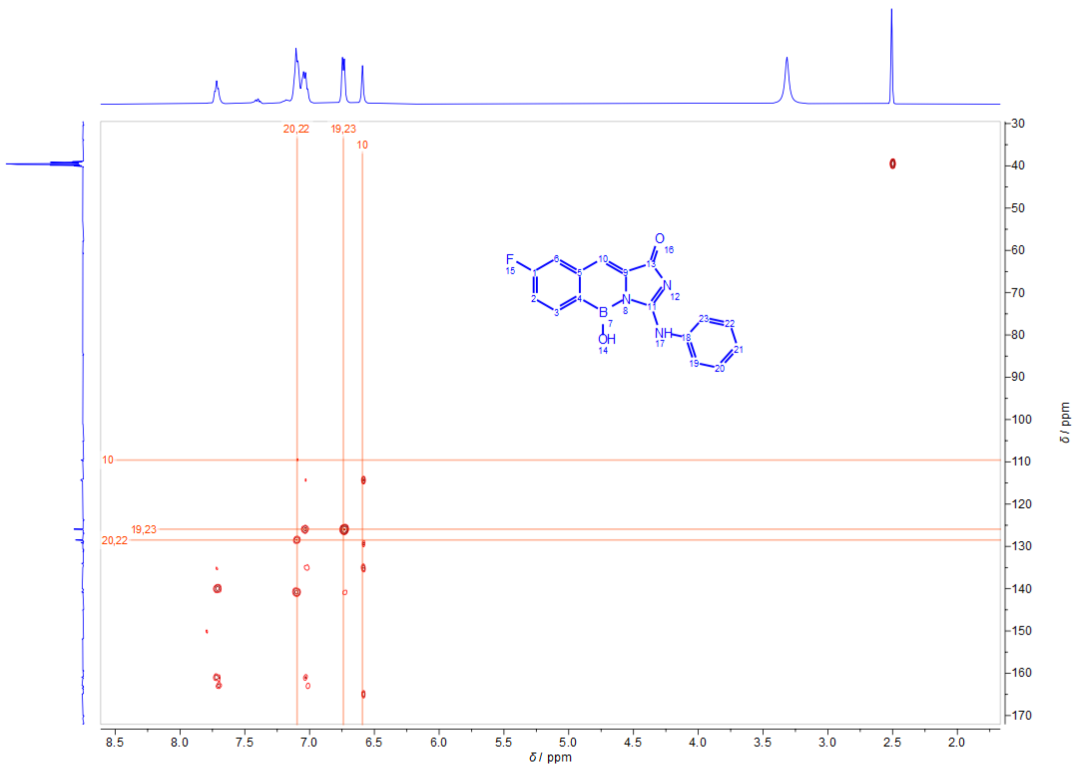

Fig S34 HMBC spectrum of **5a** recorded at 500 MHz in DMSO-*d6*

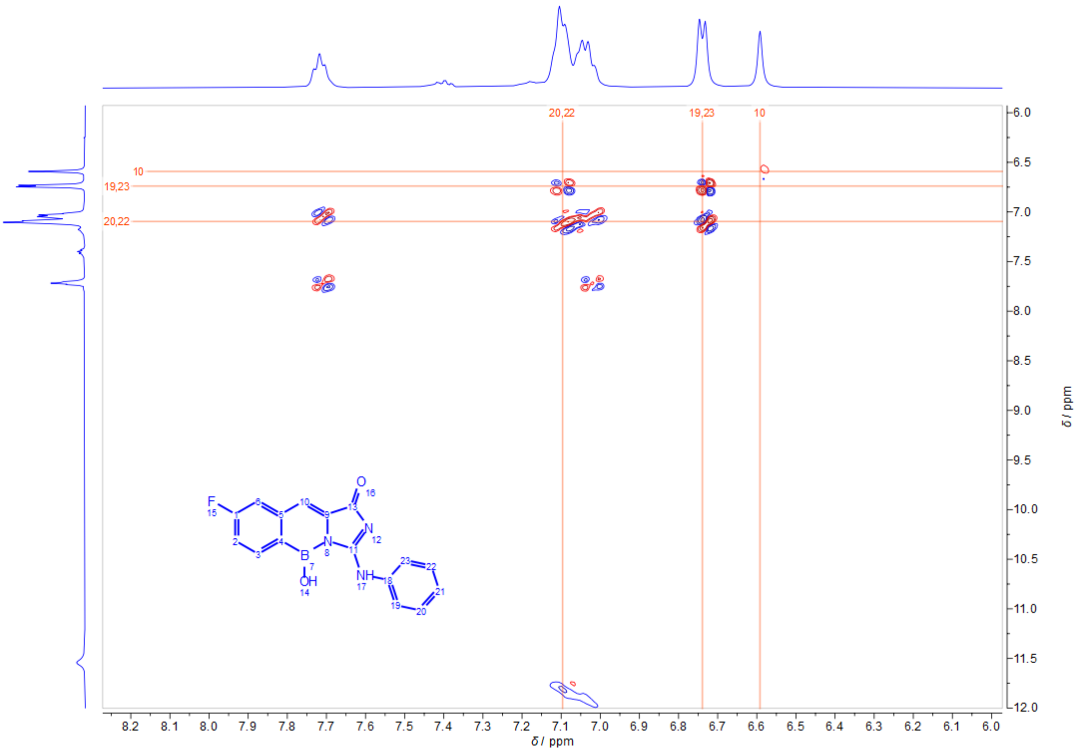

Fig S35. COSY spectrum of **5a** recorded at 500 MHz in DMSO-*d6*

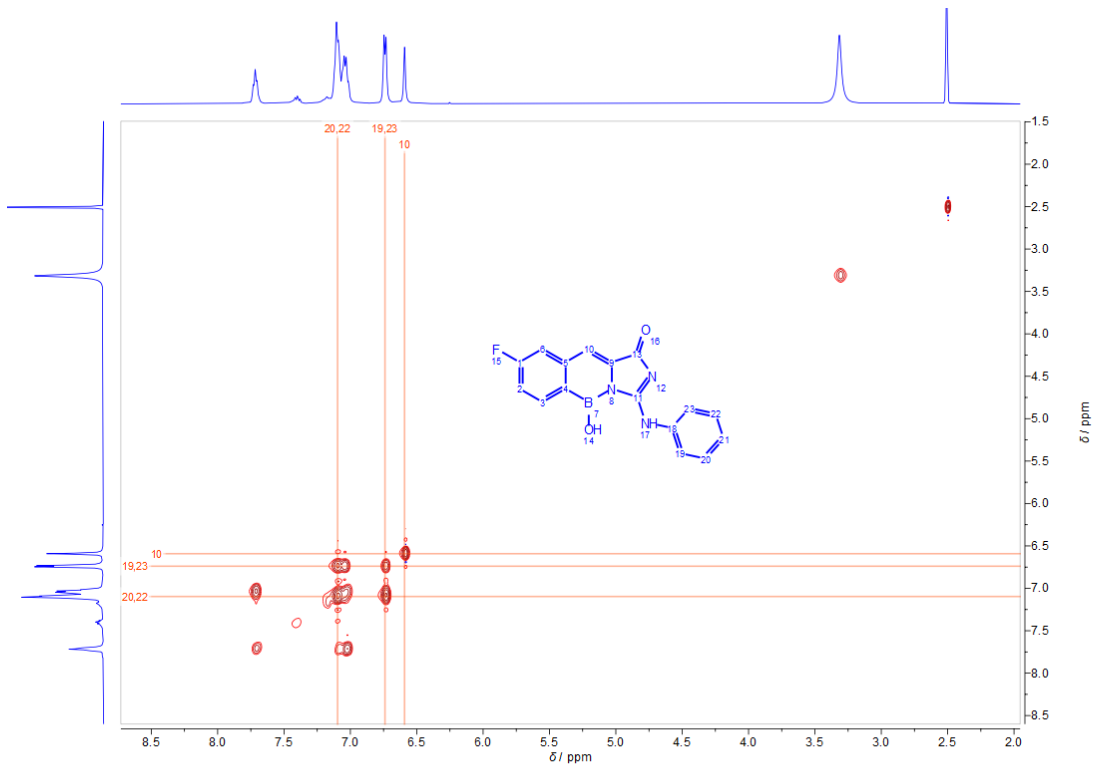

Fig S36. TOCSY spectrum of **5a** recorded at 500 MHz in DMSO-*d6*

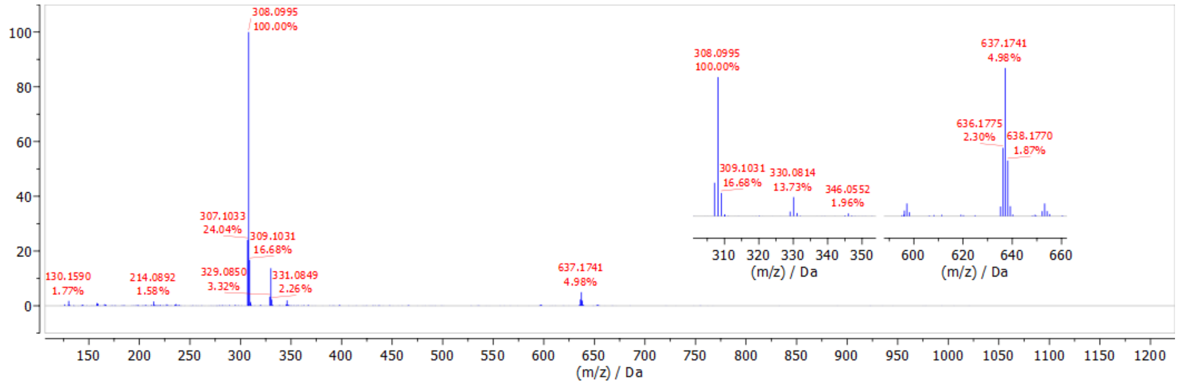

Fig S37. HRMS (ESI-Q-Orbitrap) spectrum of **5a**. *m*/*z*: $[M + H]^+$ Calcd for $C_{16}H_{12}O_2N_3BF$ 308.1001; Found 308.0995.

S2.7 NMR and HRMS spectra of 6a

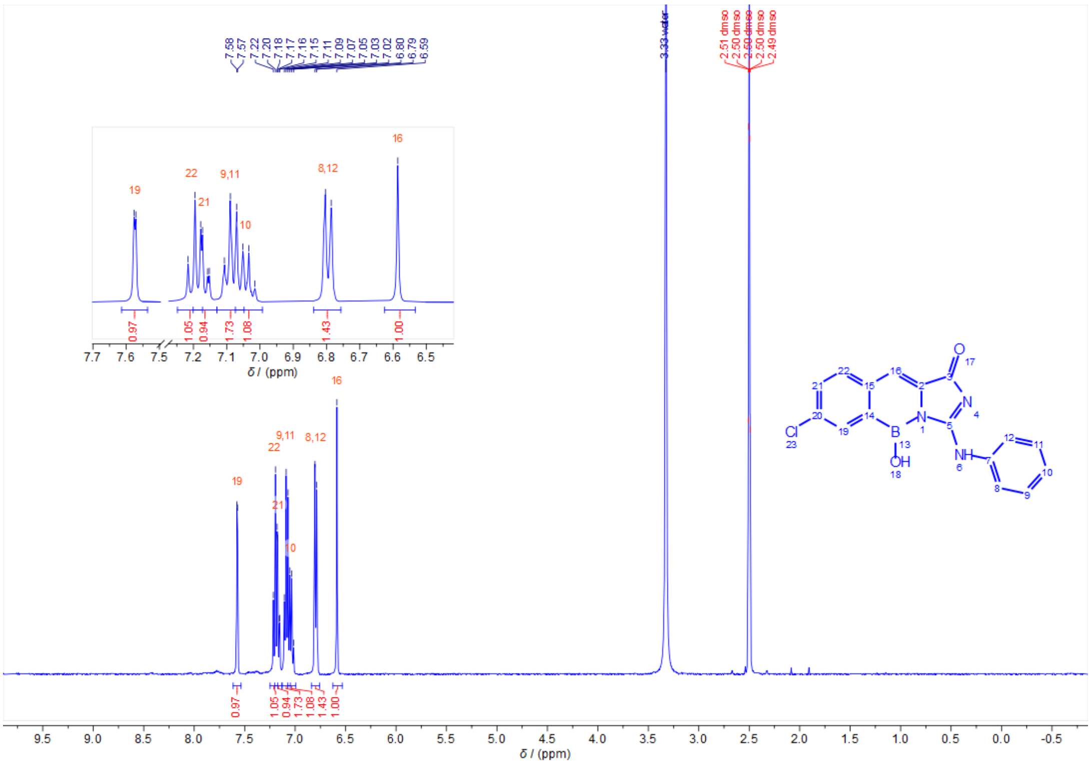

Fig S38. ^{1}H NMR spectrum of **6a** recorded at 400 MHz in DMSO-*d6*

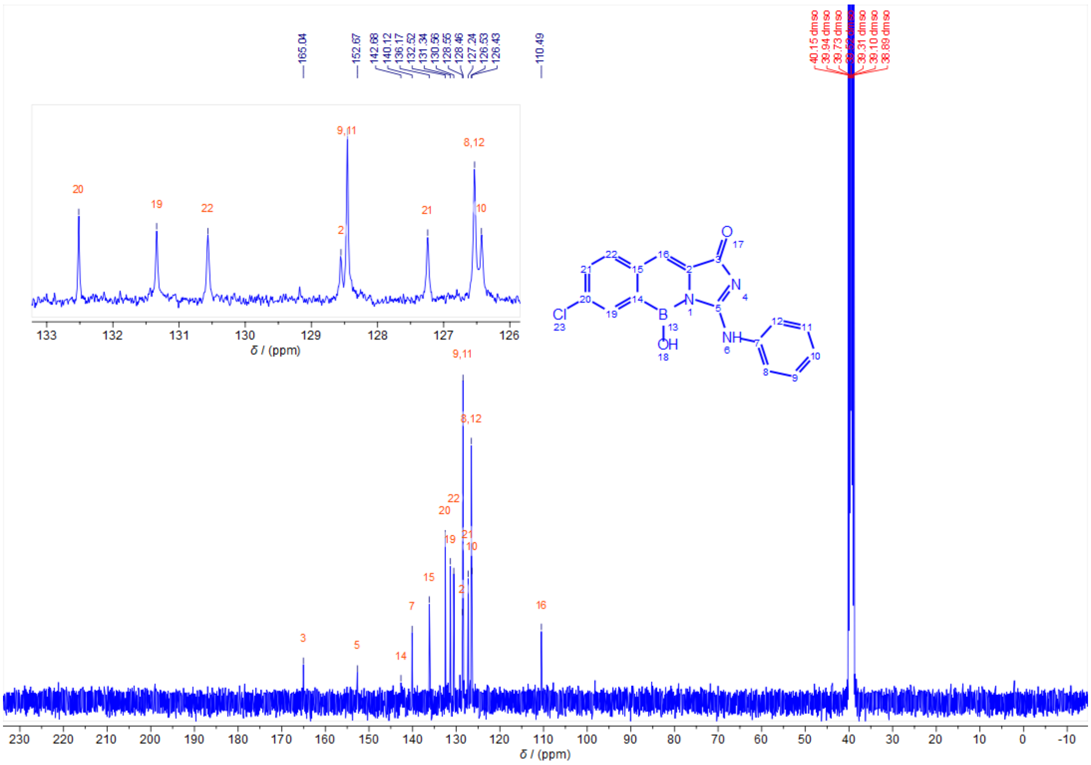

Fig S39. ^{13}C NMR spectrum of **6a** recorded at 101 MHz in DMSO-*d6*

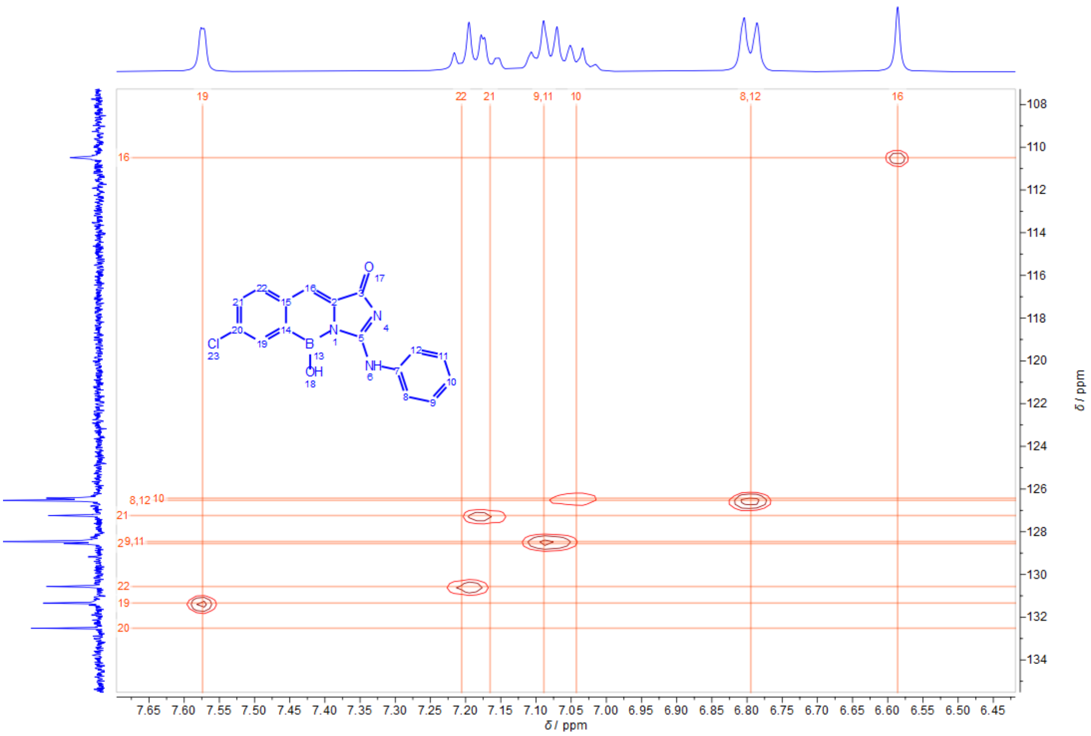

Fig S40. HSQC spectrum of **6a** recorded at 400 MHz in DMSO-*d6*

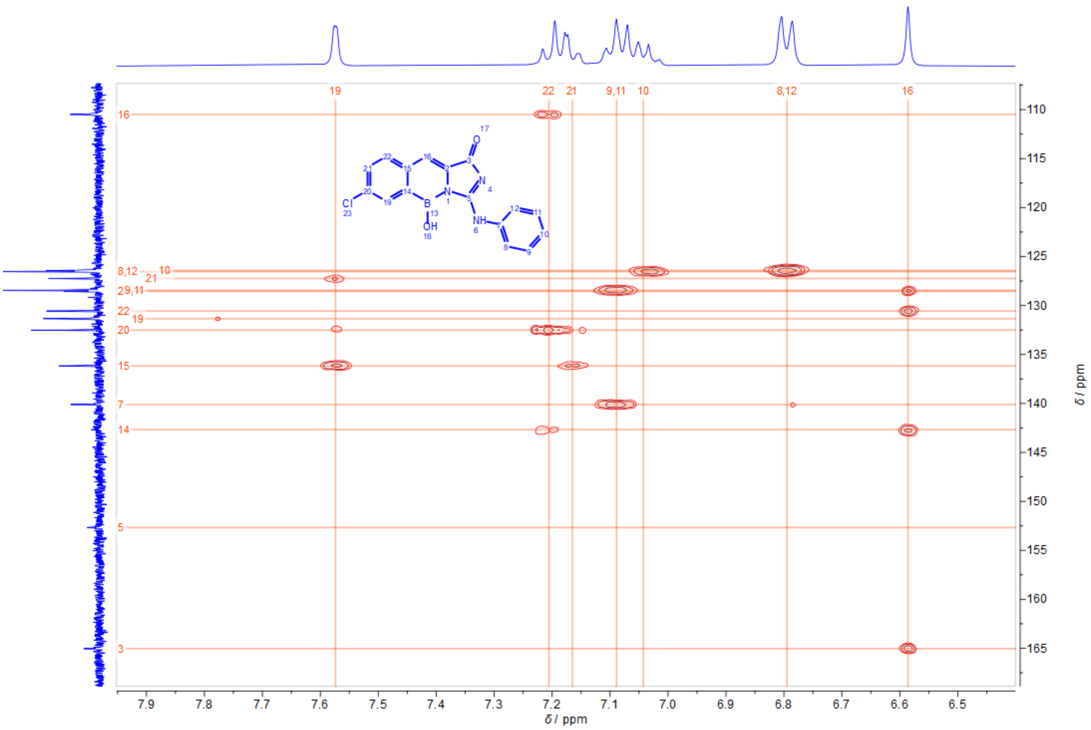

Fig S41. HMBC spectrum of **6a** recorded at 400 MHz in DMSO-*d6*

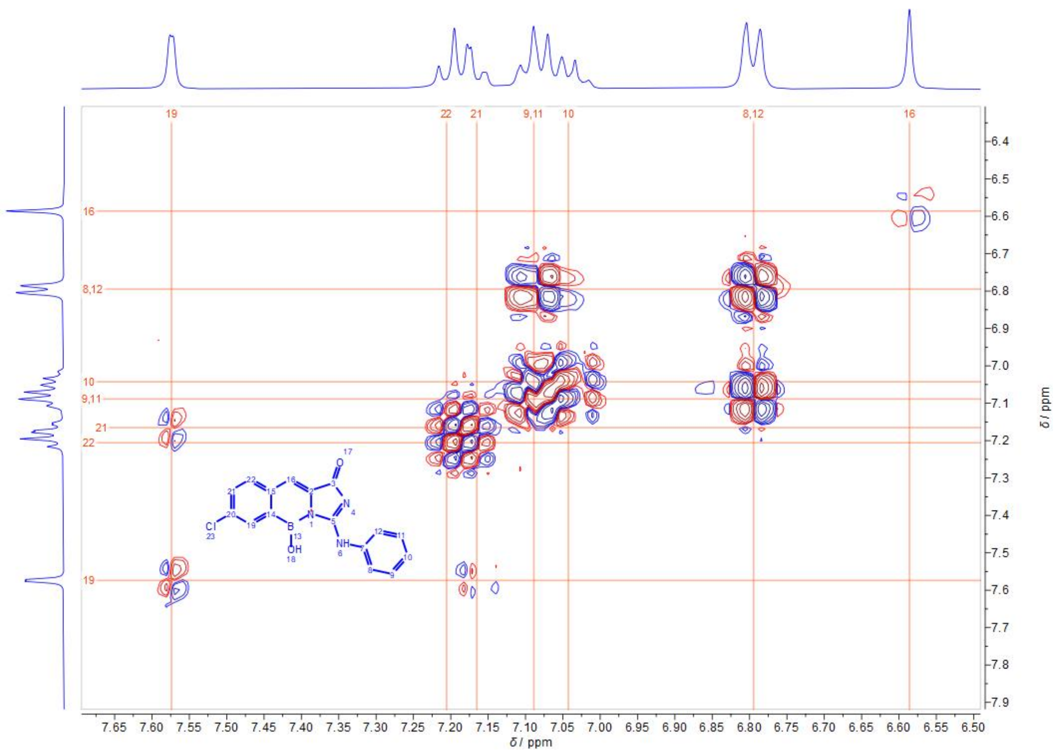

Fig S42. COSY spectrum of **6a** recorded at 400 MHz in DMSO-*d6*

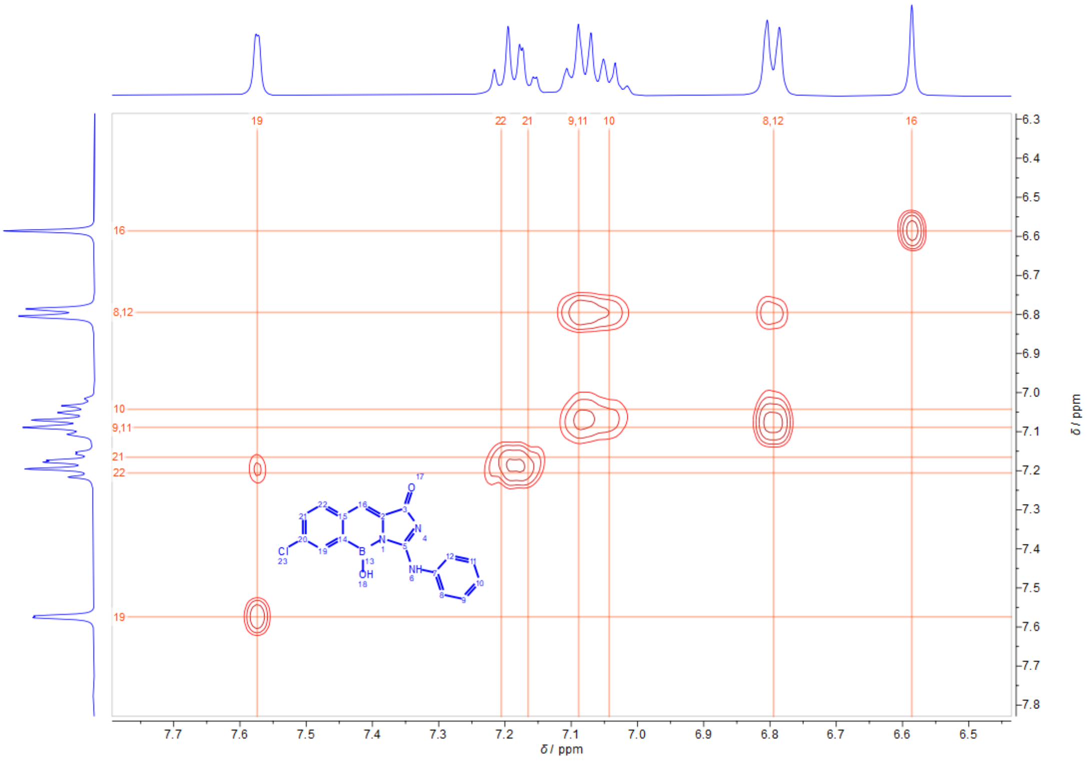

Fig S43. TOCSY spectrum of **6a** recorded at 400 MHz in DMSO-*d6*

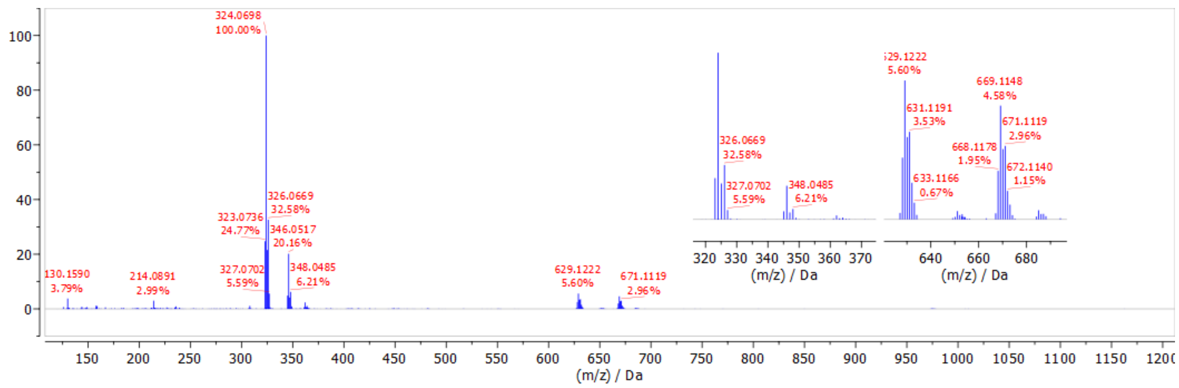

Fig S44. HRMS (ESI-Q-Orbitrap) spectrum of **6a**. *m/z*: $[M + H]^+$ Calcd for $C_{16}H_{12}O_2N_3BCl$ 324.0706; Found 324.0698.

S2.8 NMR and HRMS spectra of 7a

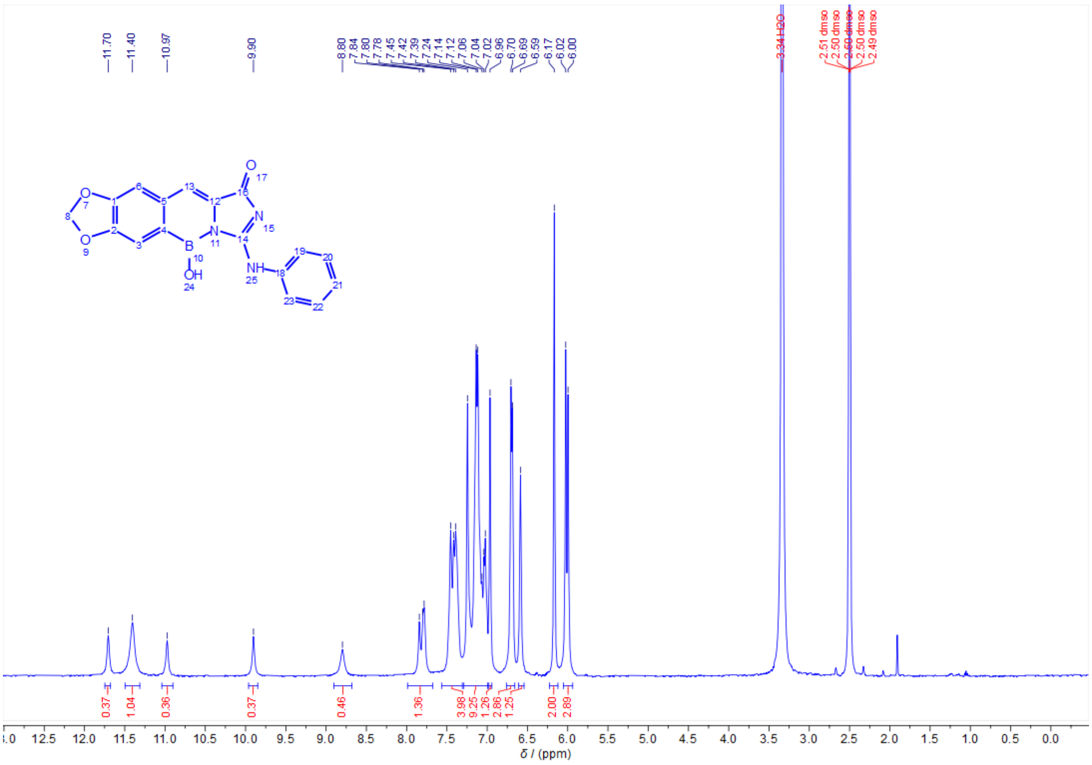

Fig S45. ^1H NMR spectrum of **7a** recorded at 400 MHz in DMSO-*d6*

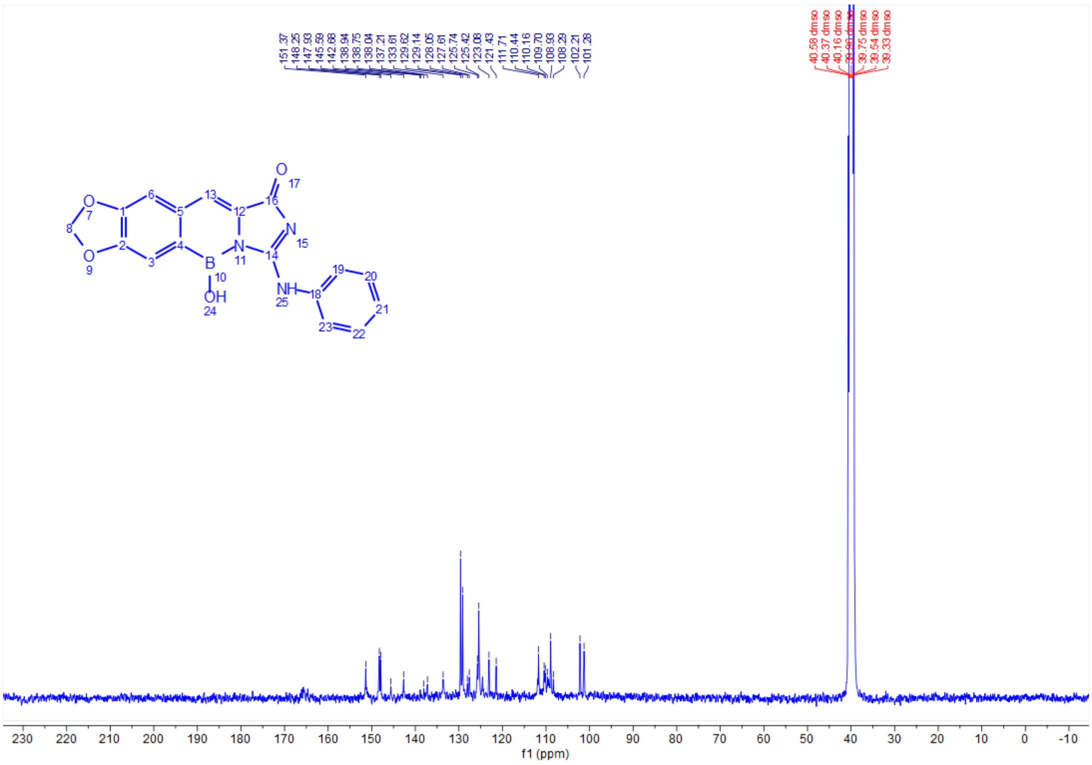

Fig S46. ^{13}C NMR spectrum of **7a** recorded at 101 MHz in DMSO-*d6*

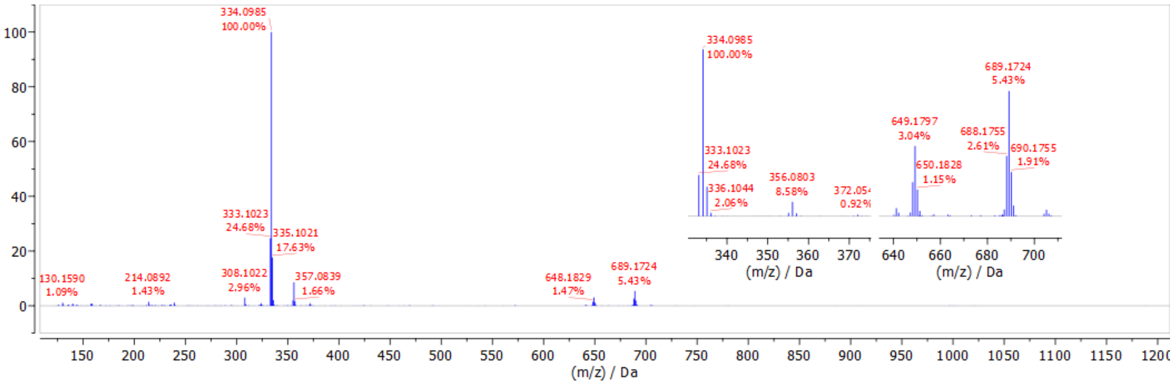

Fig S47. HRMS (ESI-Q-Orbitrap) spectrum of **7a**. *m*/*z*: $[M + H]^+$ Calcd for $C_{17}H_{13}O_4N_3B$ 334.0994; Found 334.0985.

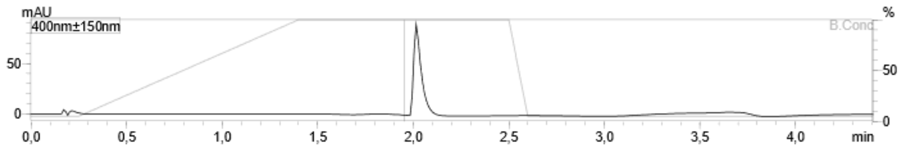

Fig S48. HPLC-DAD chromatogram of **7a** recorded in the 250-550 nm range.

S2.9 NMR and HRMS spectra of 1b

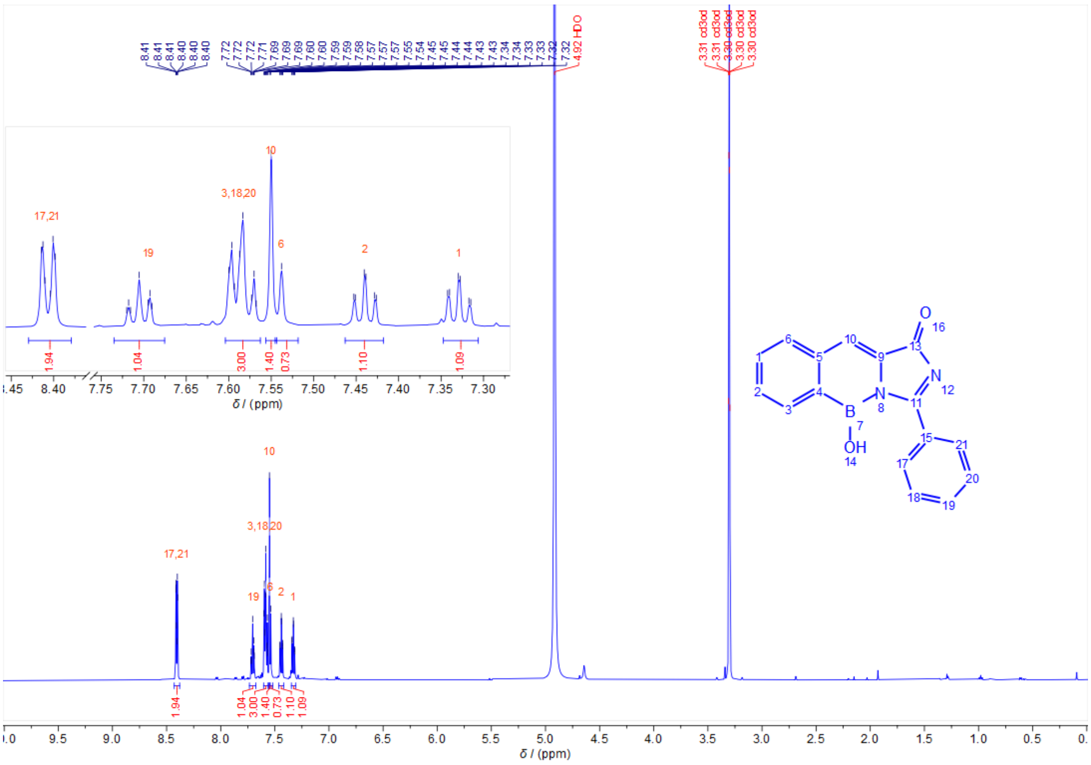

Fig S49. ^{1}H NMR spectrum of **1b** recorded at 60 MHz in CD_3OD

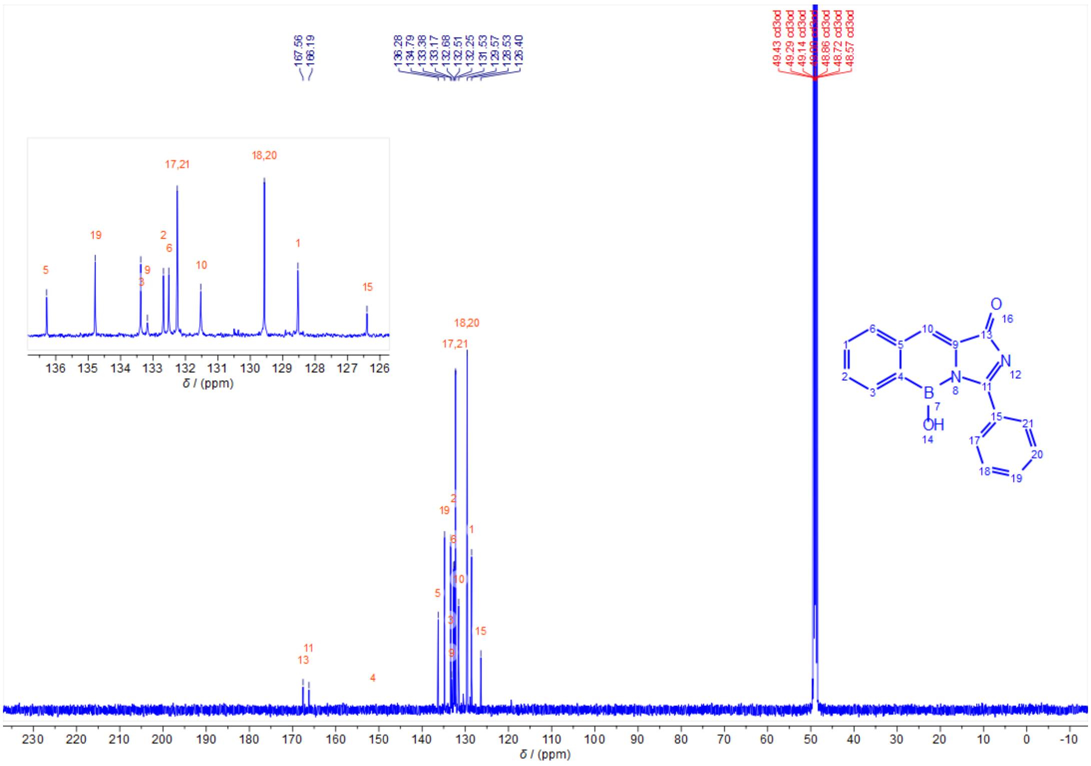

Fig S50. ^{13}C NMR spectrum of **1b** recorded at 151 MHz in CD_3OD

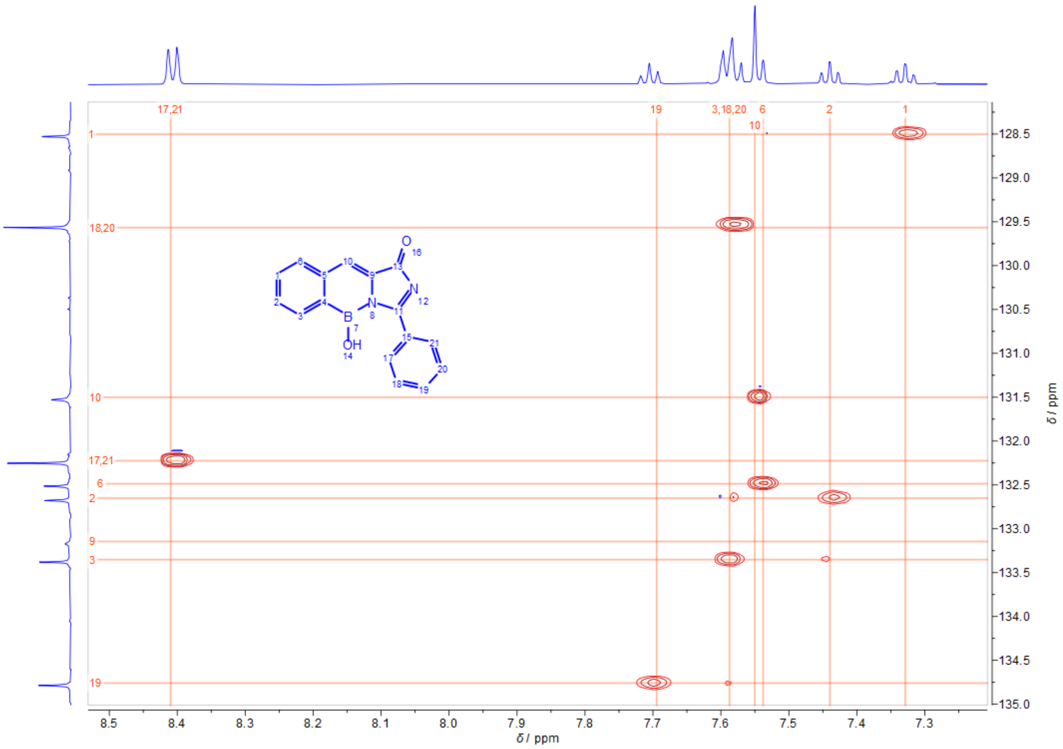

Fig S51. HSQC spectrum of **1b** recorded at 600 MHz in CD_3OD

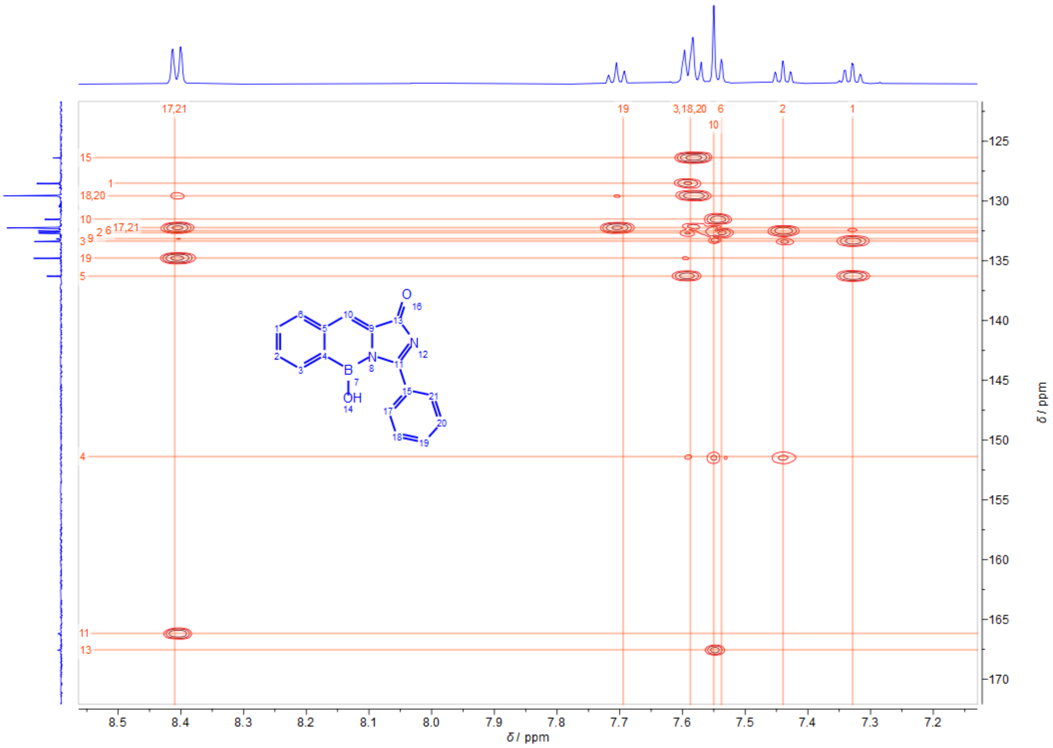

Fig S52. HMBC spectrum of **1b** recorded at 600 MHz in CD_3OD

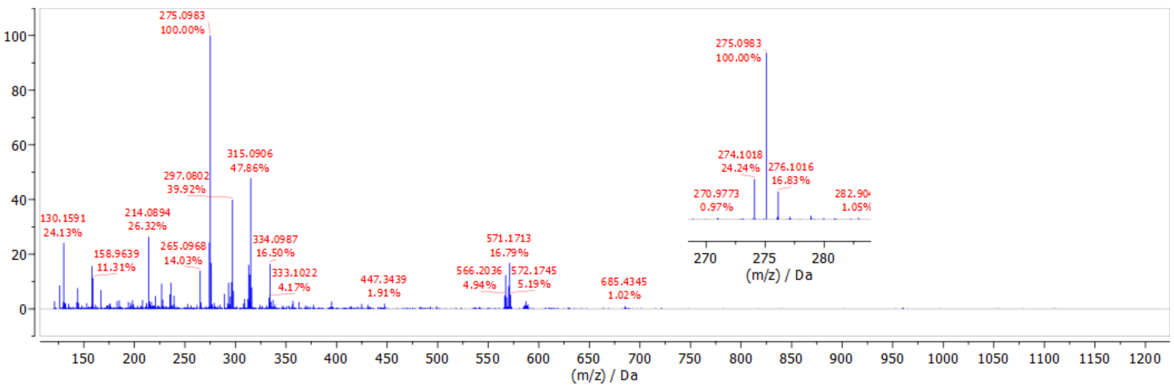

Fig S53. HRMS (ESI-Q-Orbitrap) spectrum of **1b**. m/z: $[M + H]^+$ Calcd for $C_{16}H_{12}O_2N_2B$ 275.0986; Found 275.0983.

S2.10 NMR and HRMS spectra of 2b

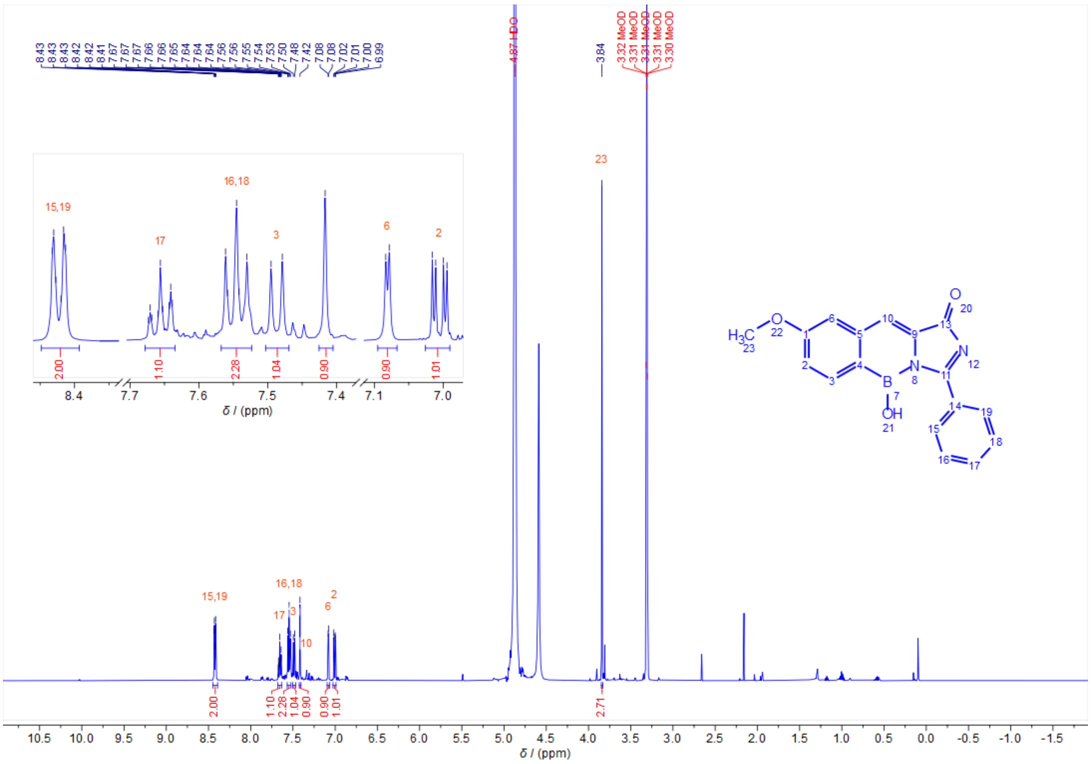

Fig S54. ^{1}H NMR spectrum of **2b** recorded at 500 MHz in CD_3OD

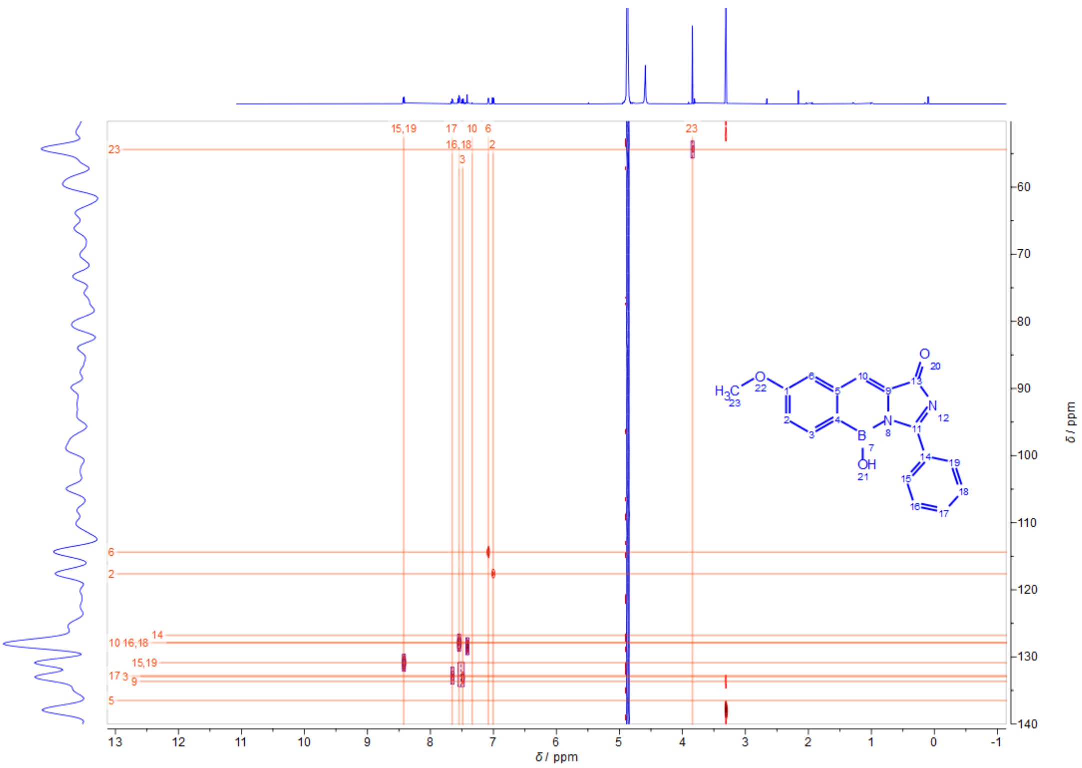

Fig S55. HSQC spectrum of **2b** recorded at 500 MHz in CD_3OD

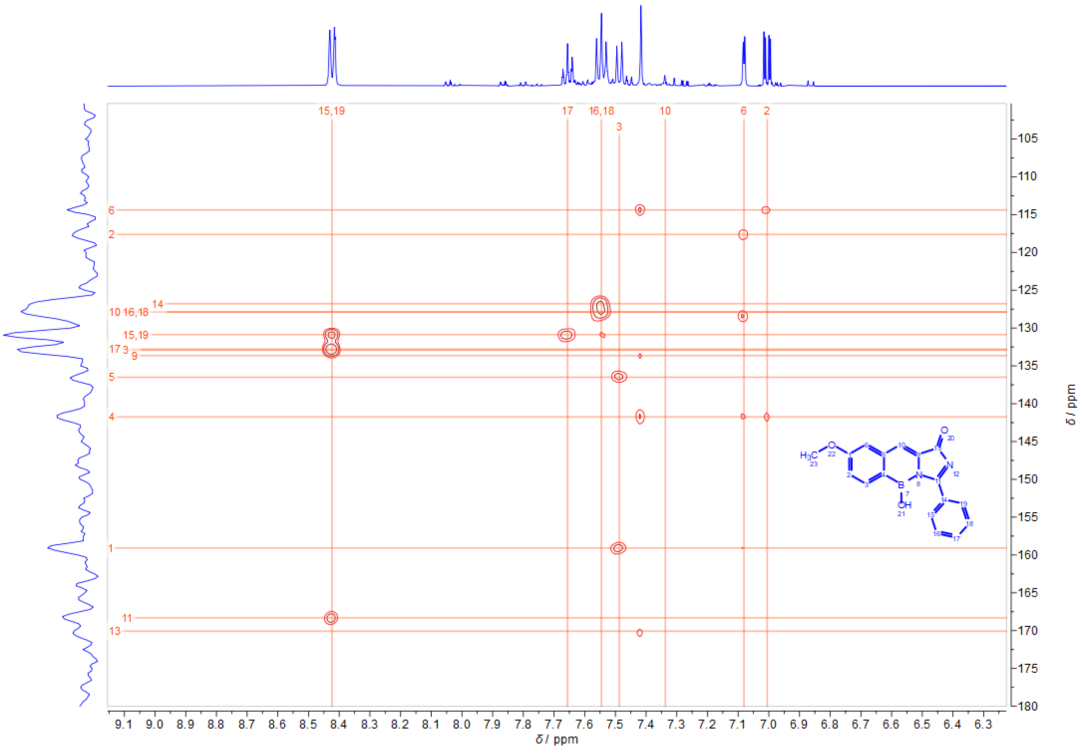

Fig S56. HMBC spectrum of **2b** recorded at 500 MHz in CD_3OD

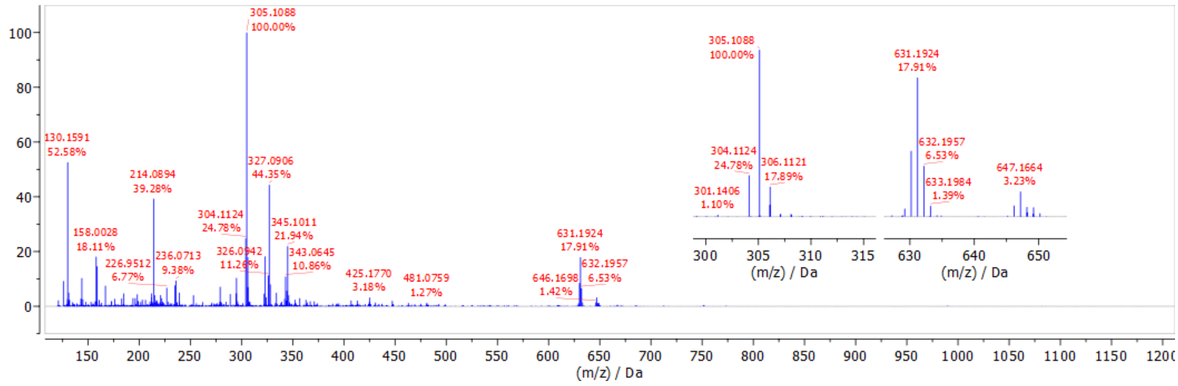

Fig S57. HRMS (ESI-Q-Orbitrap) spectrum of **2b**. *m/z*: $[M + H]^+$ Calcd for $C_{17}H_{14}O_3N_2B$ 305.1092; Found 305.1088.

S2.11 NMR and HRMS spectra of 6b

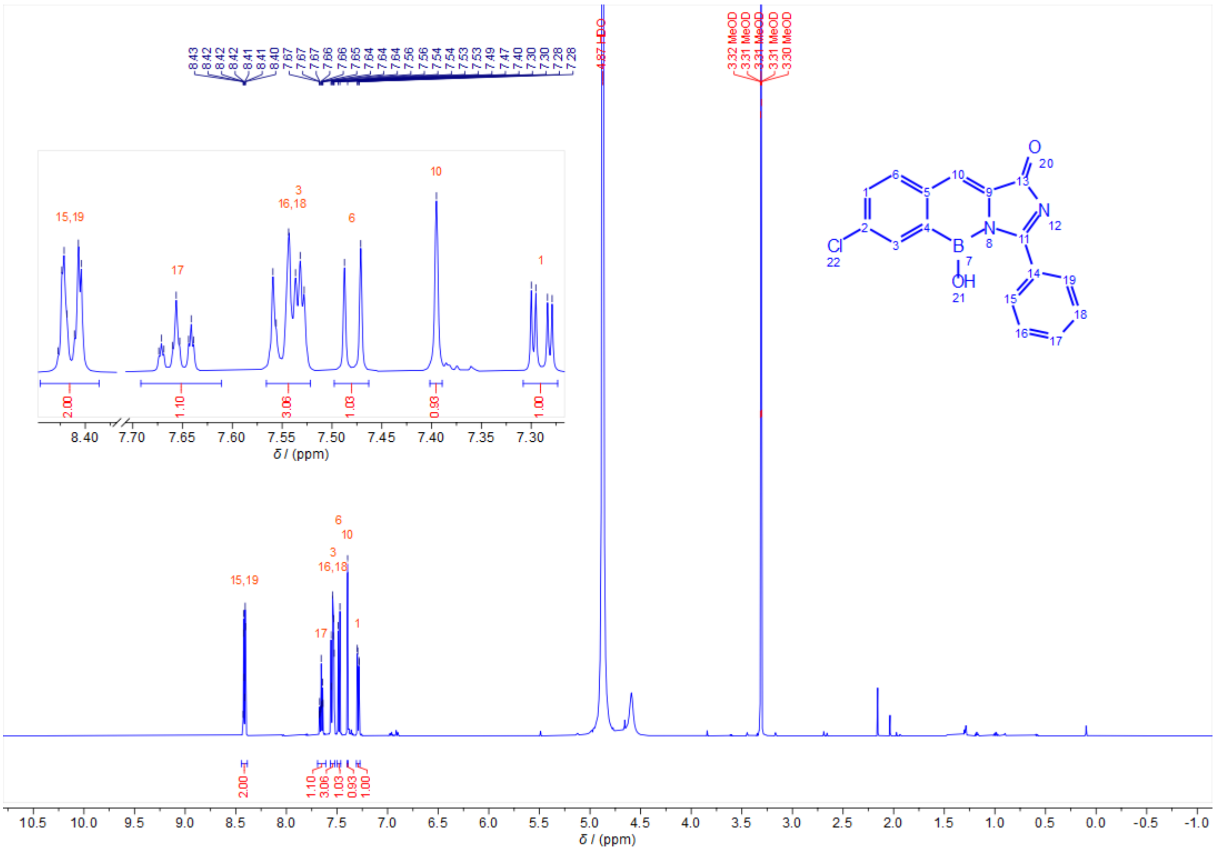

Fig S58. ^1H NMR spectrum of **6b** recorded at 500 MHz in CD_3OD

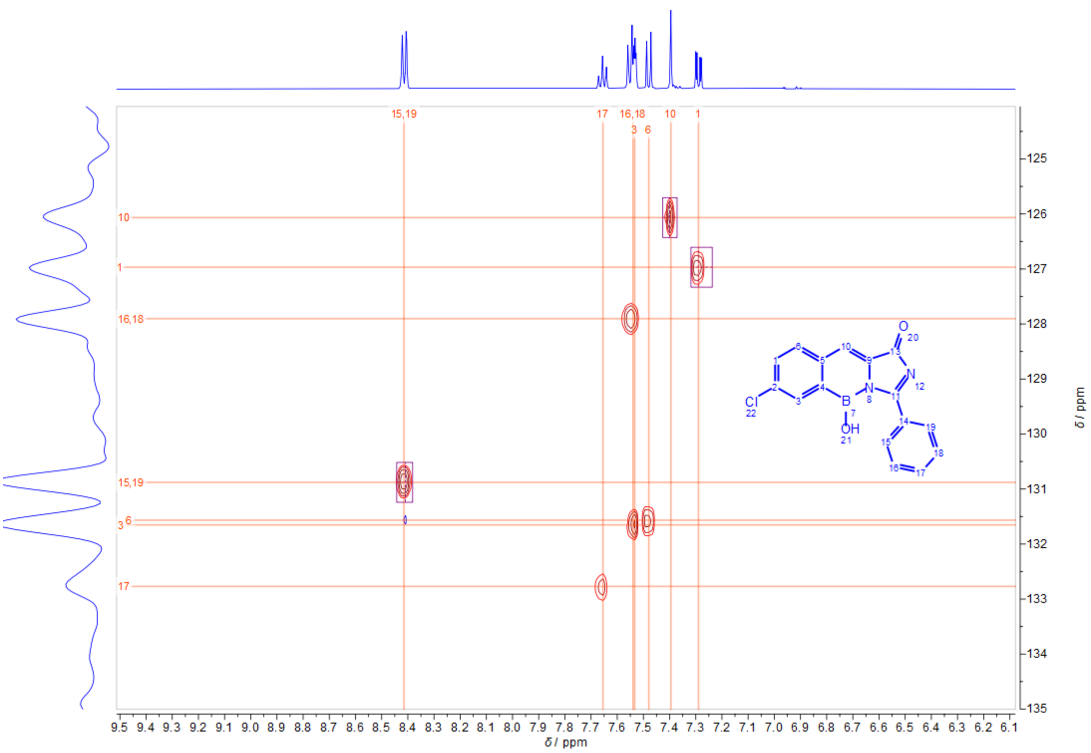

Fig S59. HSQC spectrum of **6b** recorded at 500 MHz in CD_3OD

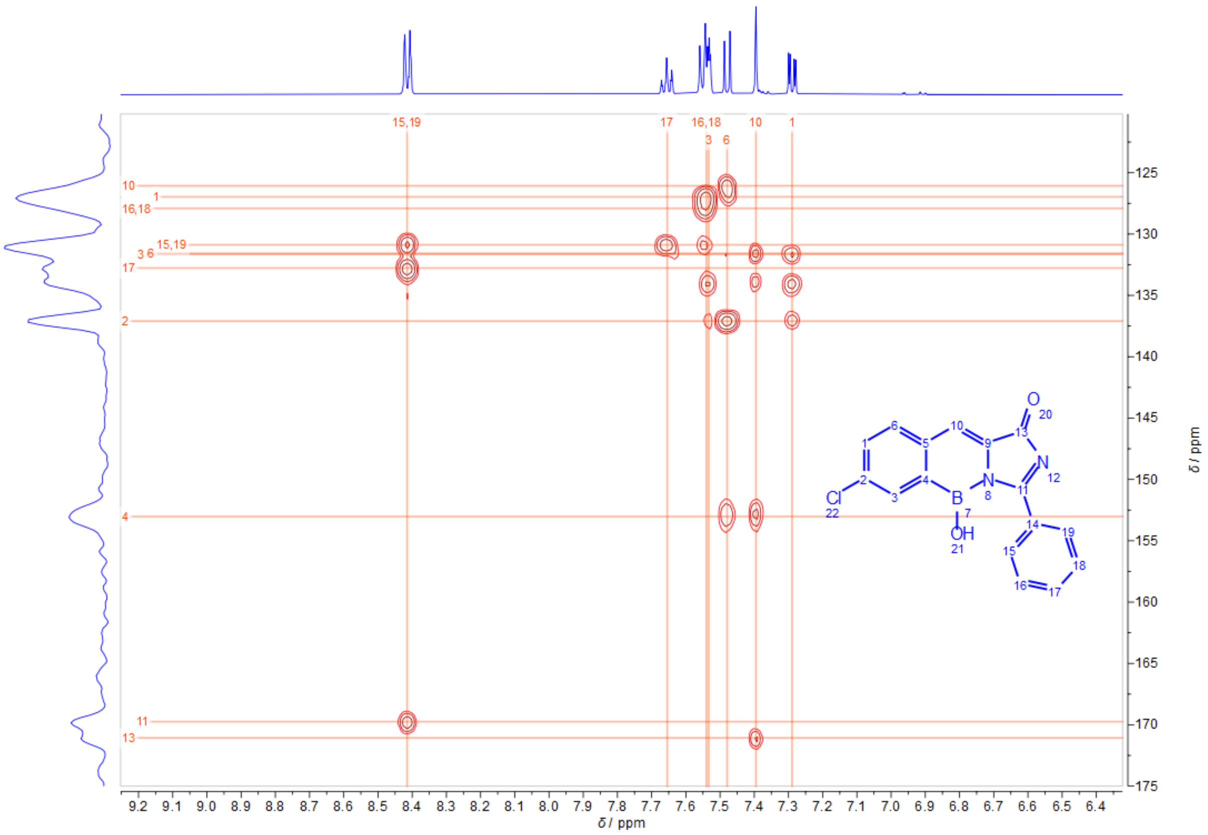

Fig S60. HMBC spectrum of **6b** recorded at 500 MHz in CD_3OD

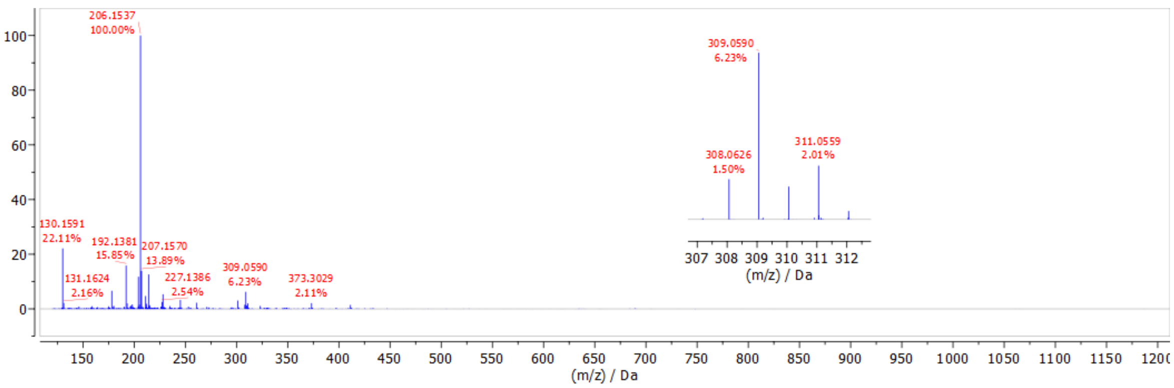

Fig S61. HRMS (ESI-Q-Orbitrap) spectrum of **6b**. *m/z*: $[M + H]^+$ Calcd for $C_{16}H_{11}O_2N_2BCl$ 309.0597; Found 309.0590.

S2.12 NMR and HRMS spectra of 7b

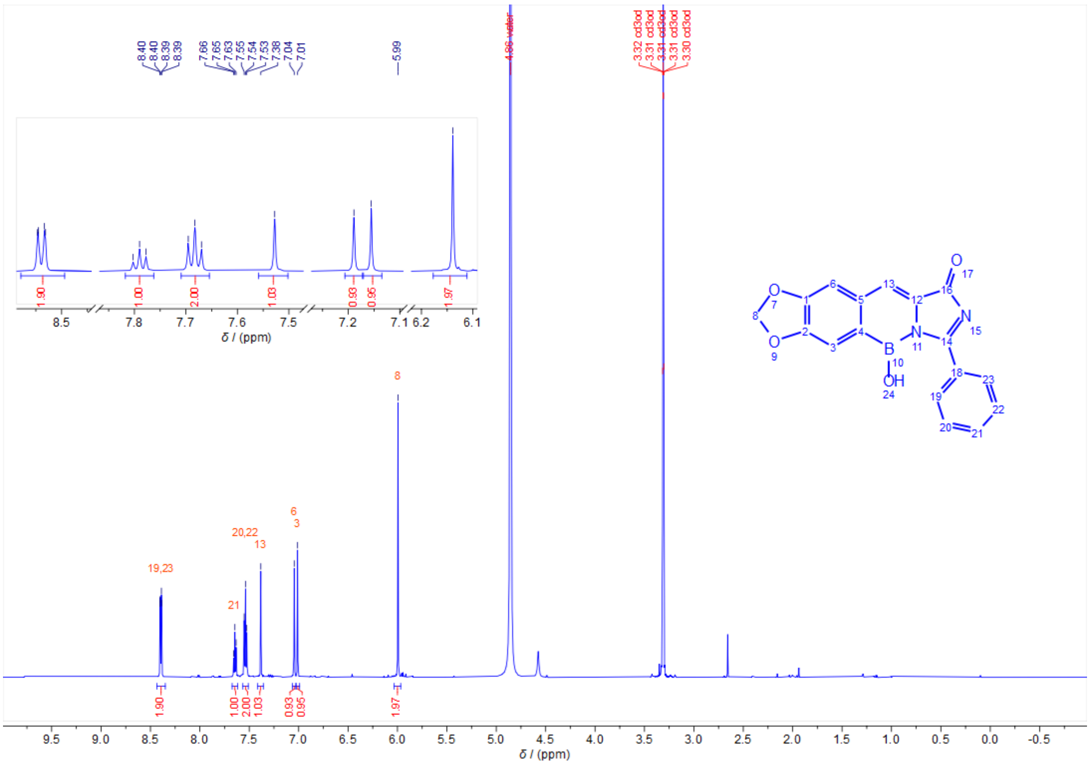

Fig S62. ^1H NMR spectrum of **7b** recorded at 600 MHz in CD_3OD

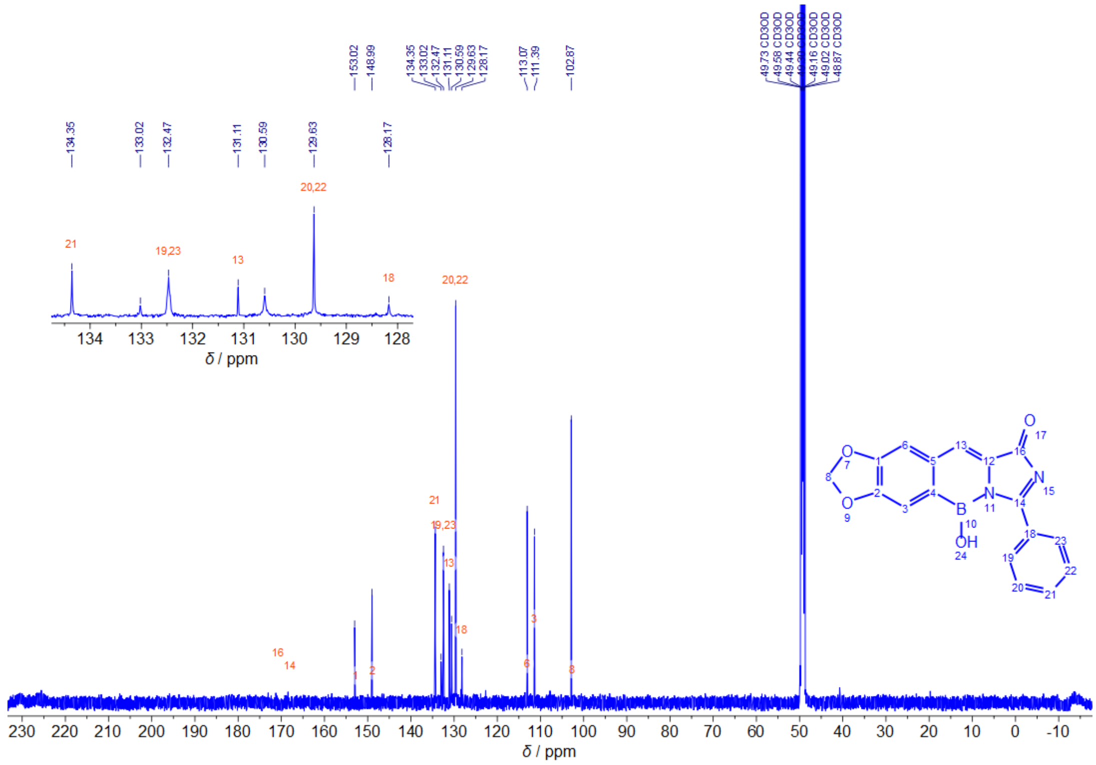

Fig S63. ^{13}C NMR spectrum of **7b** recorded at 151 MHz in CD_3OD

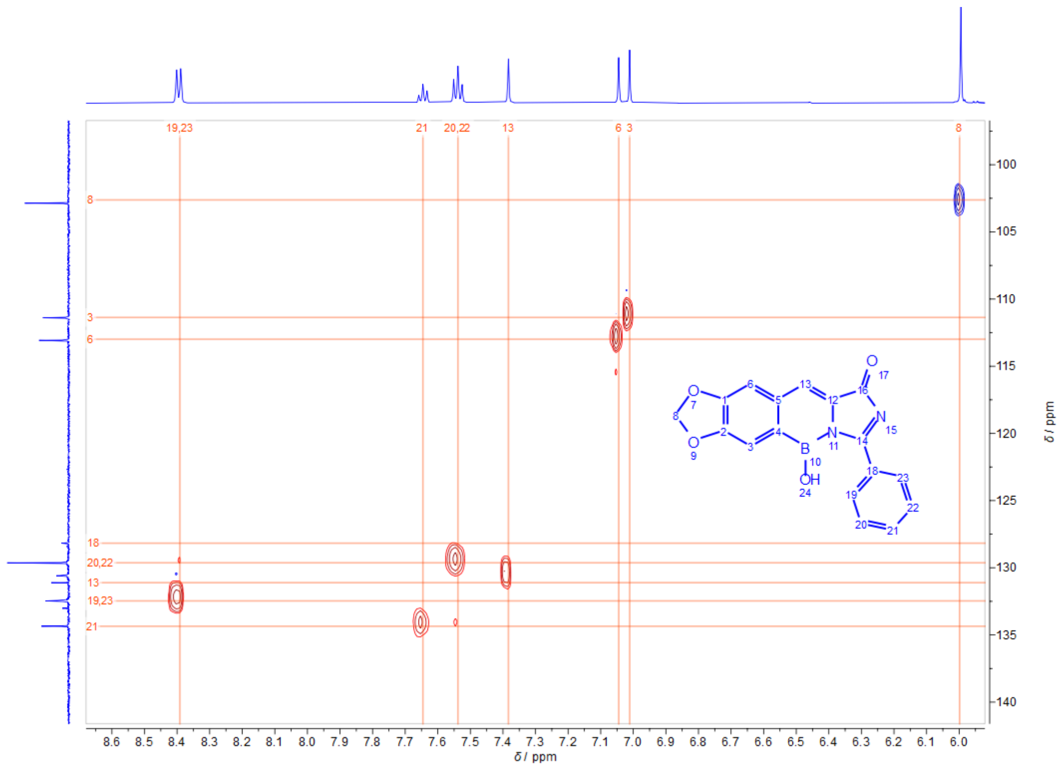

Fig S64. HSQC spectrum of **7b** recorded at 600 MHz in CD_3OD

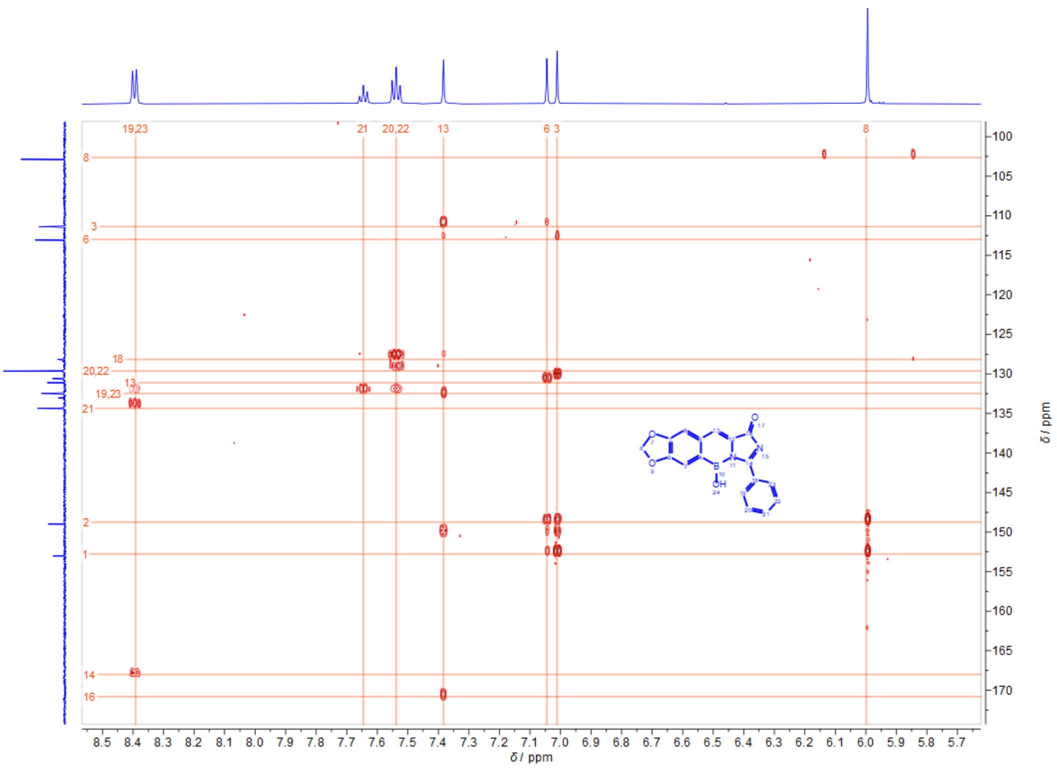

Fig S65. HMBC spectrum of **7b** recorded at 600 MHz in CD_3OD

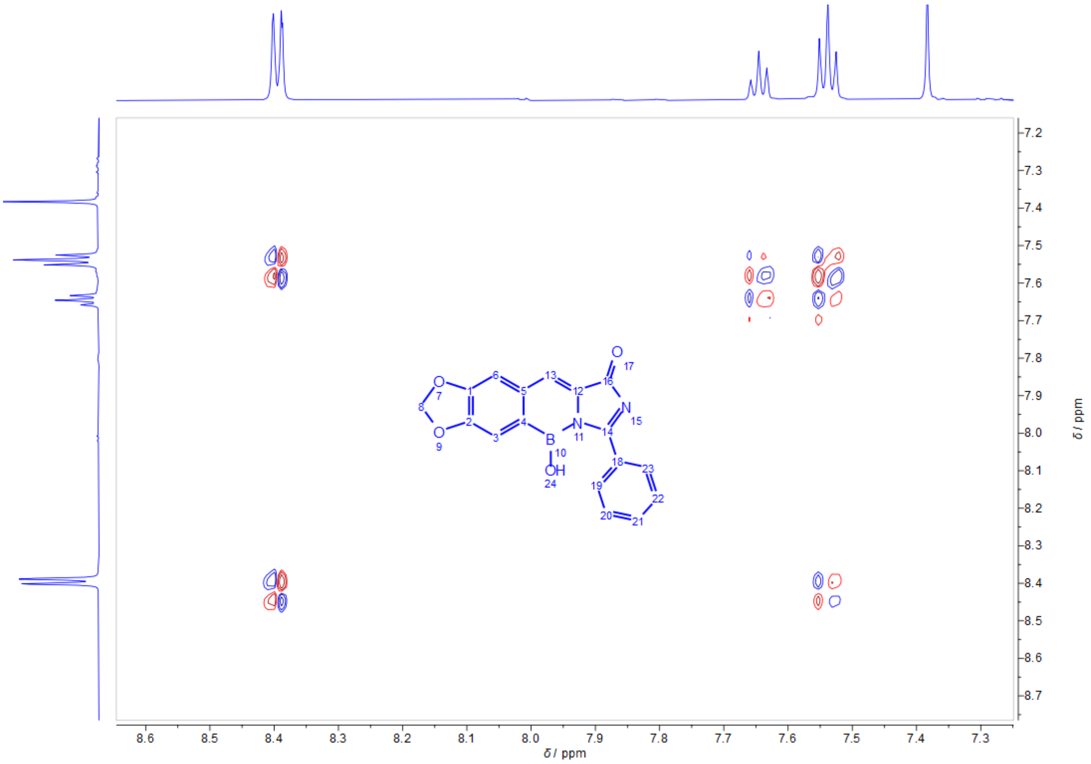

Fig S66. COSY spectrum of **7b** recorded at 600 MHz in CD_3OD.

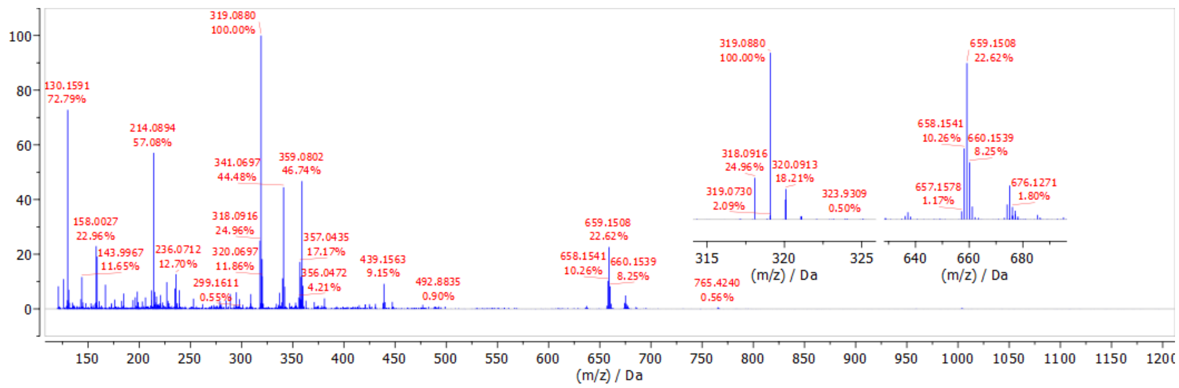

Fig S67. HRMS (ESI-Q-Orbitrap) spectrum of **7b**. *m*/*z*: $[M + H]^+$ Calcd for $C_{17}H_{12}O_4N_2B$ 319.0885; Found 319.0880.

S2.13 NMR and HRMS spectra of 8

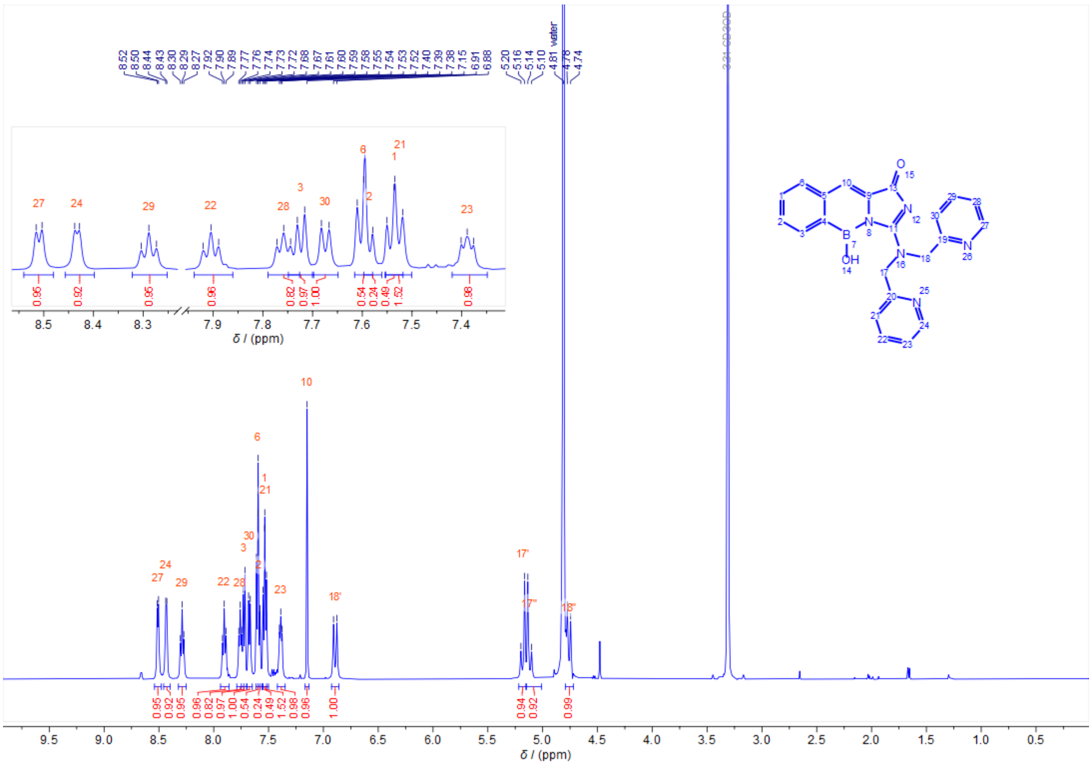

Fig S68. ^{1}H NMR spectrum of **8** recorded at 500 MHz in CD_3OD

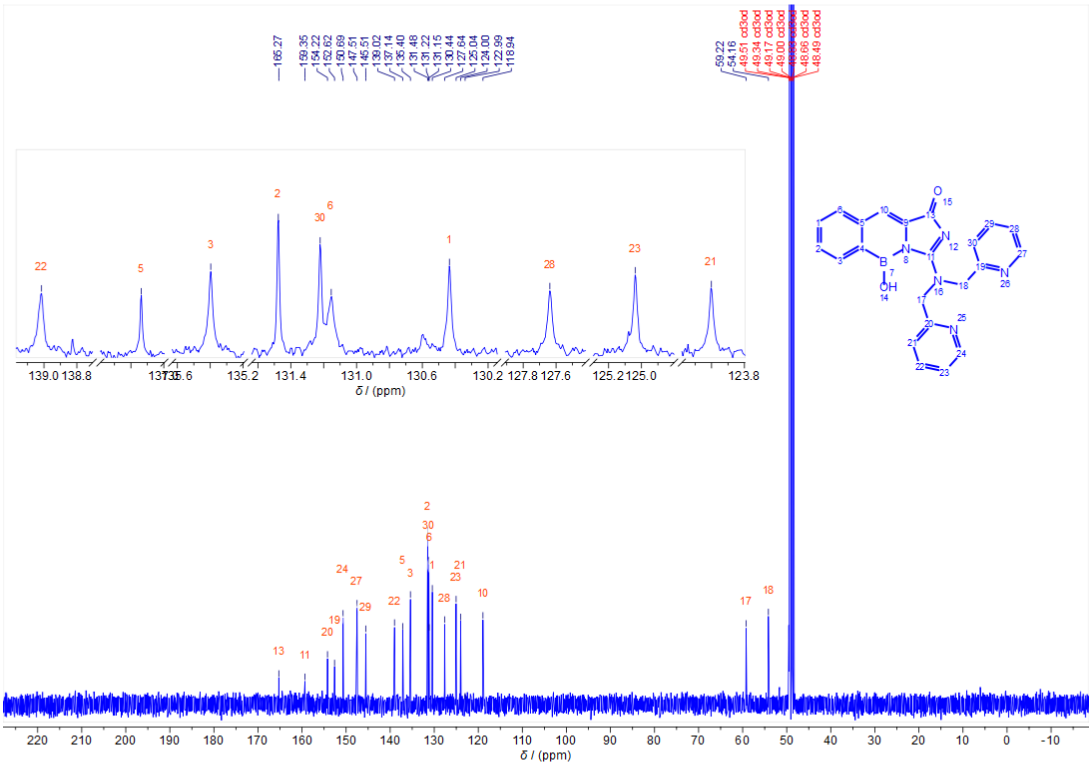

Fig S69. ^{13}C NMR spectrum of **8** recorded at 126 MHz in CD_3OD

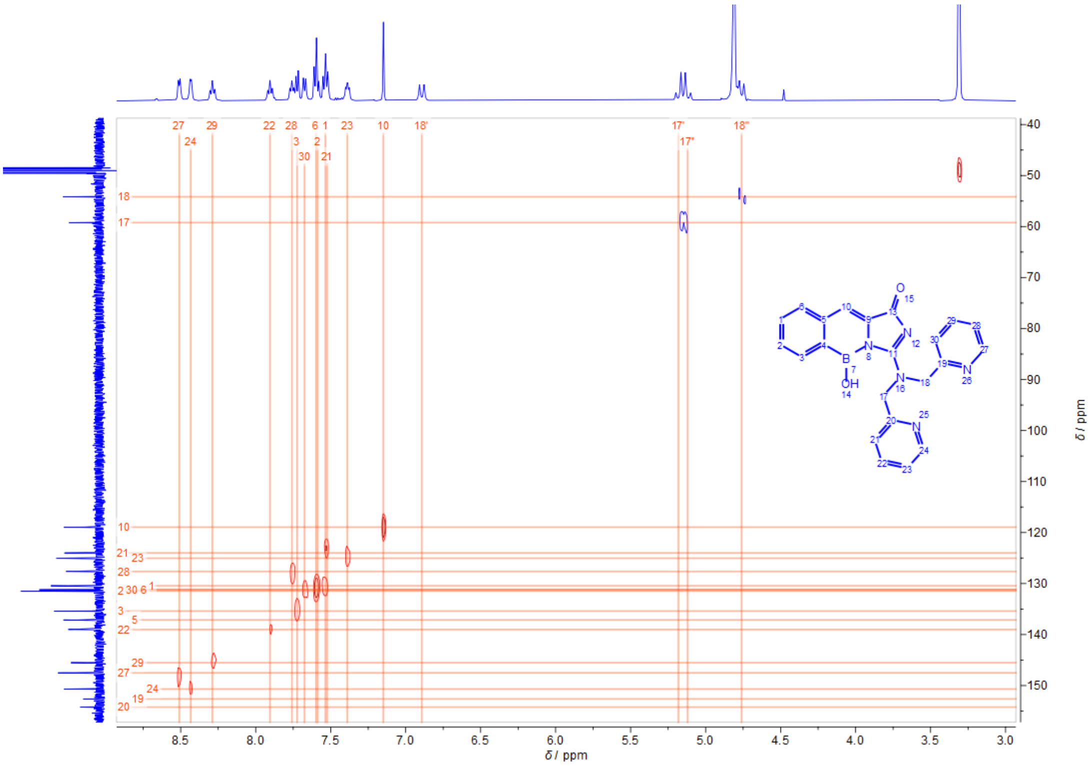

Fig S70. HSQC spectrum of **8** recorded at 500 MHz in CD_3OD

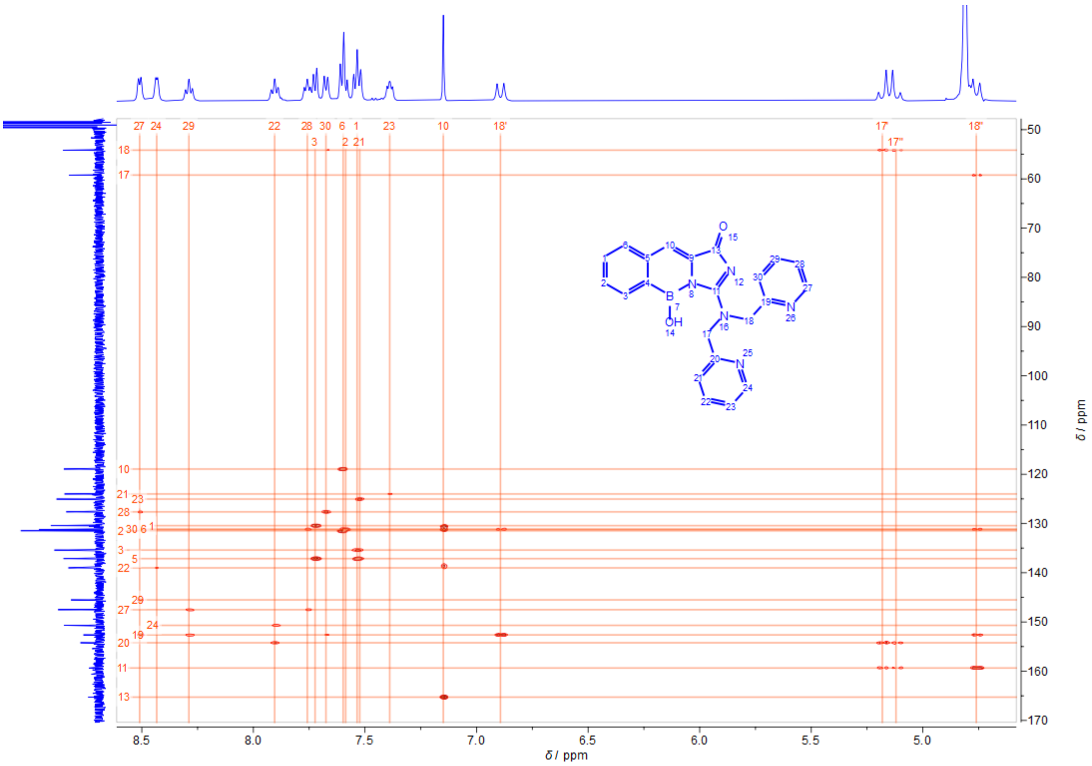

Fig S71. HMBC spectrum of **8** recorded at 500 MHz in CD_3OD

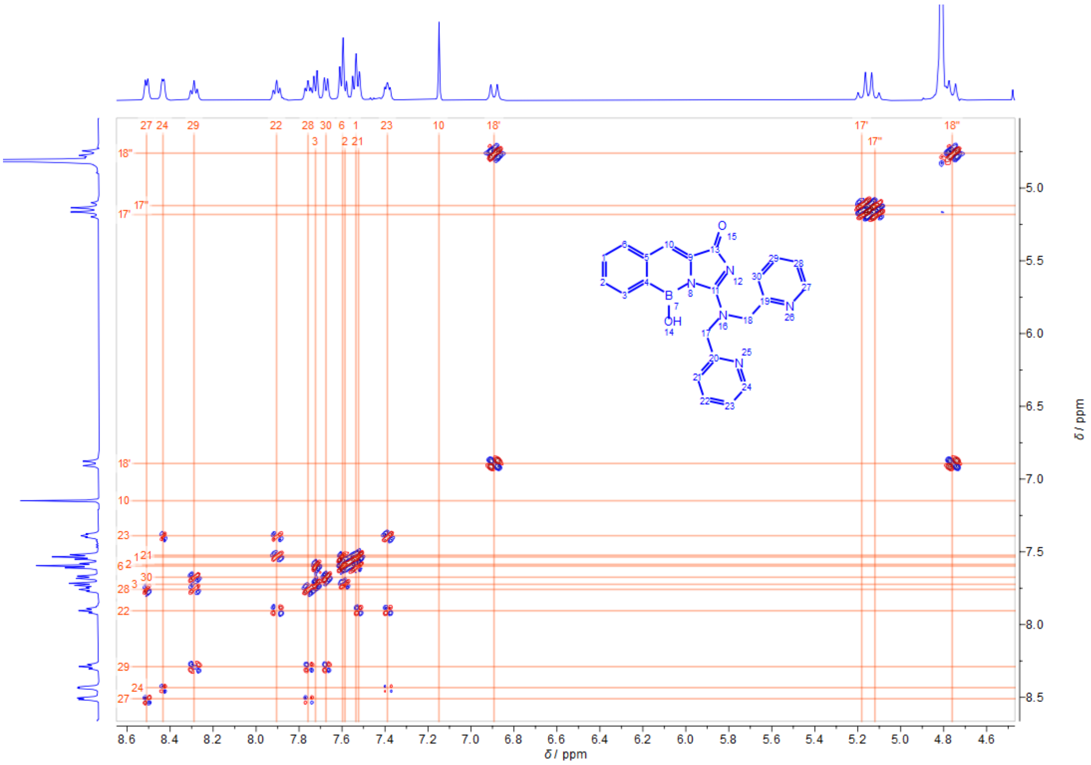

Fig S72. COSY spectrum of **8** recorded at 500 MHz in CD_3OD

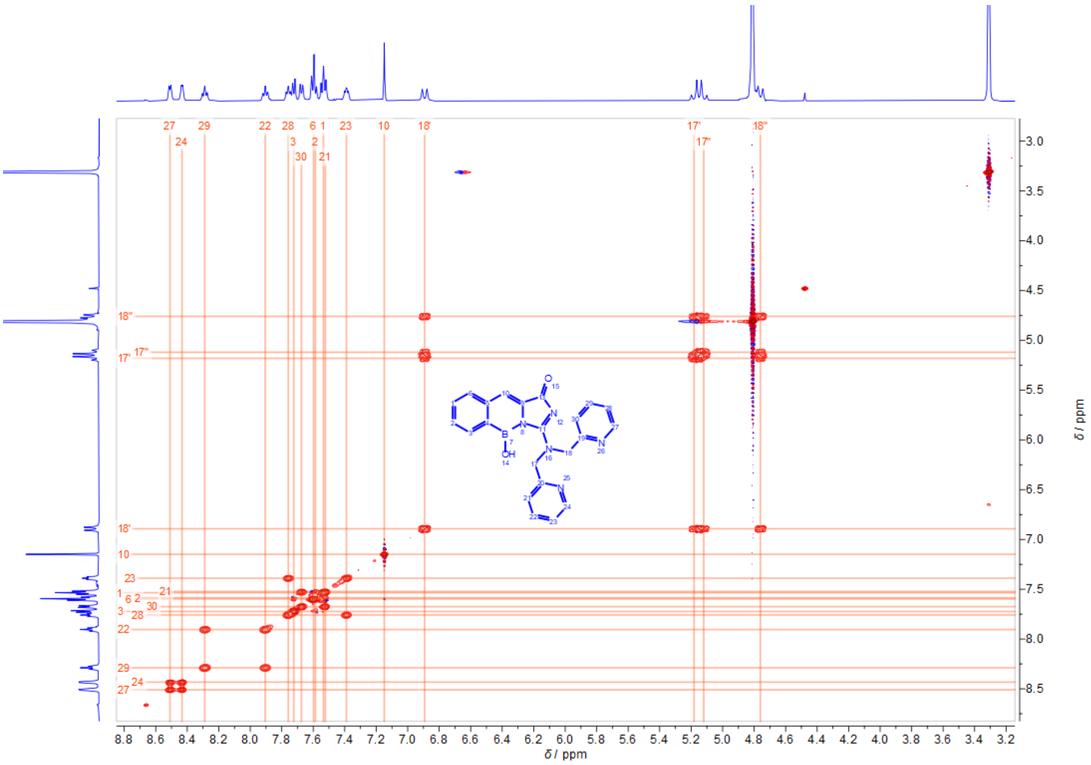

Fig S73. ROESY spectrum of **8** recorded at 500 MHz in CD_3OD

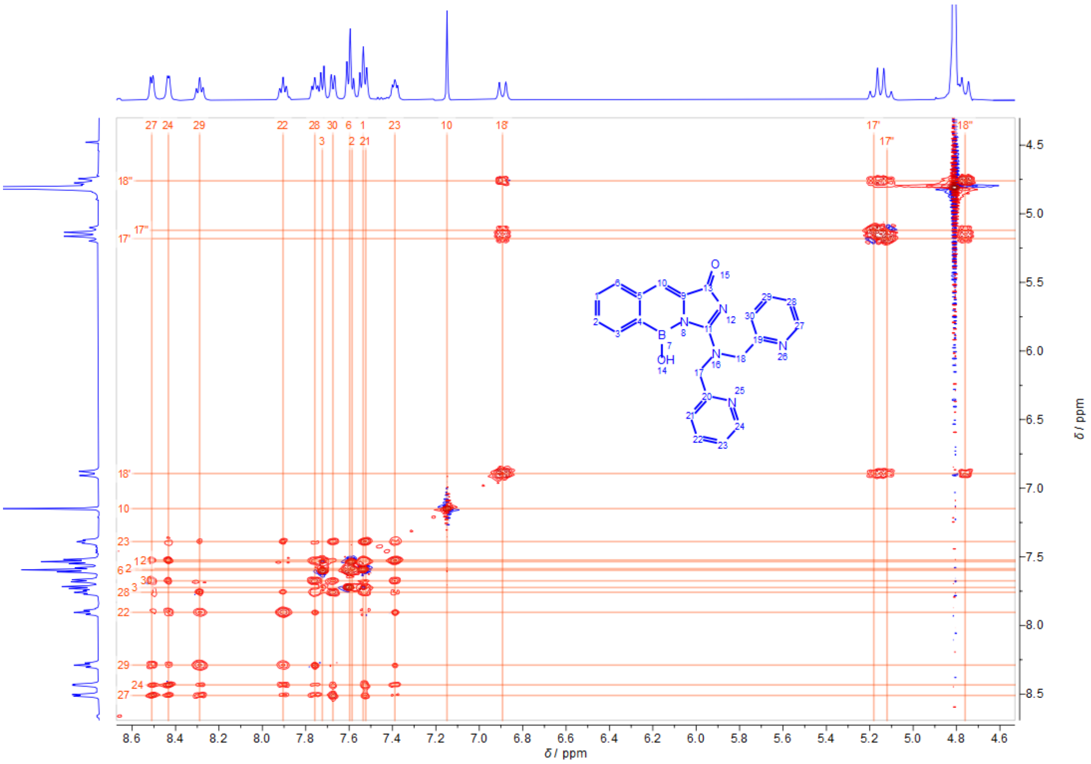

Fig S74. TOCSY spectrum of **8** recorded at 500 MHz in CD_3OD

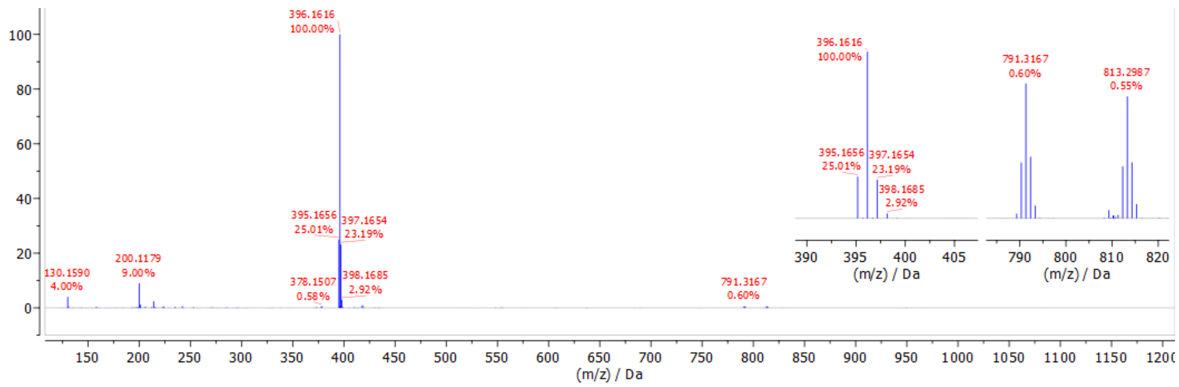

Fig S75. HRMS (ESI-Q-Orbitrap) spectrum of **8**. m/z: $[M + H]^+$ Calcd for $C_{22}H_{19}O_2N_5B$ 396.1627; Found 396.1616.

S2.14 NMR and HRMS spectra of 9

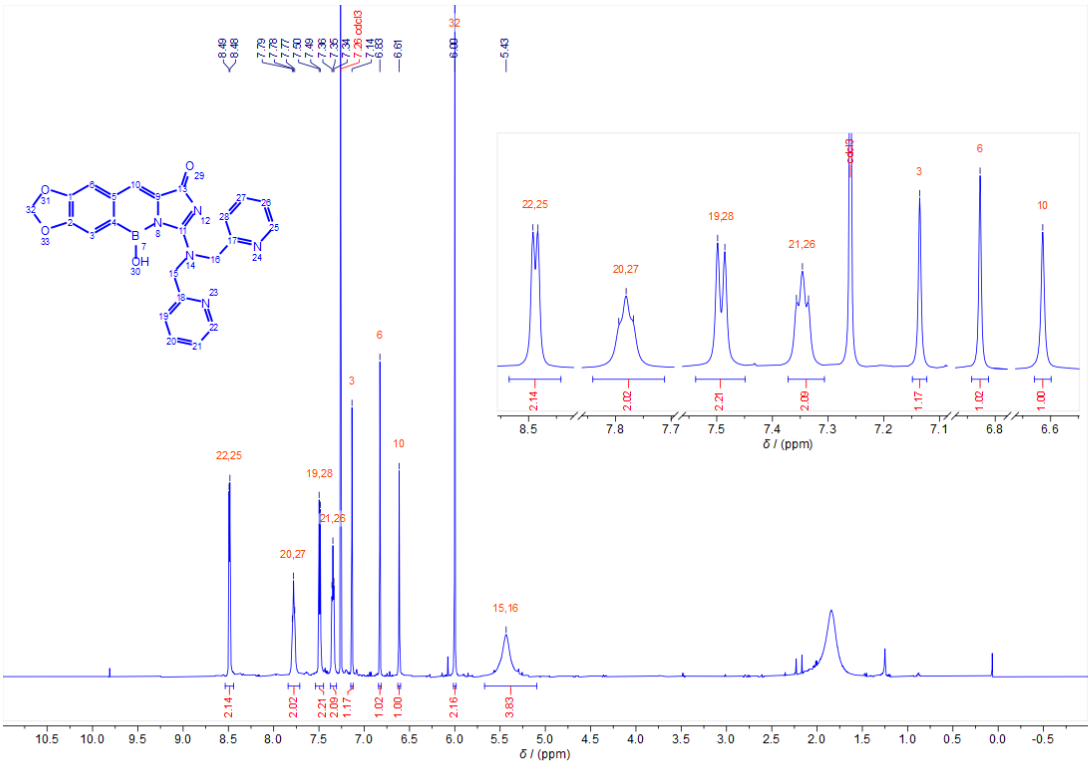

Fig S76. ^1H NMR spectrum of **9** recorded at 600 MHz in $CDCl_3$

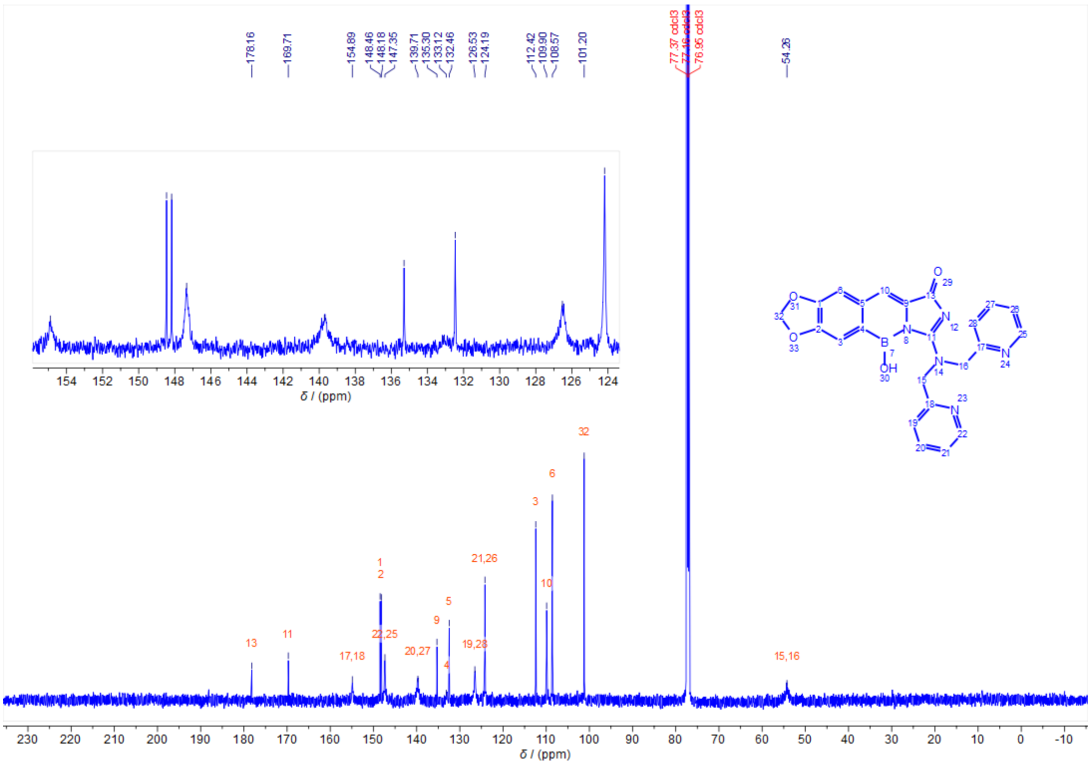

Fig S77. ^{13}C NMR spectrum of **9** recorded at 151 MHz in $CDCl_3$

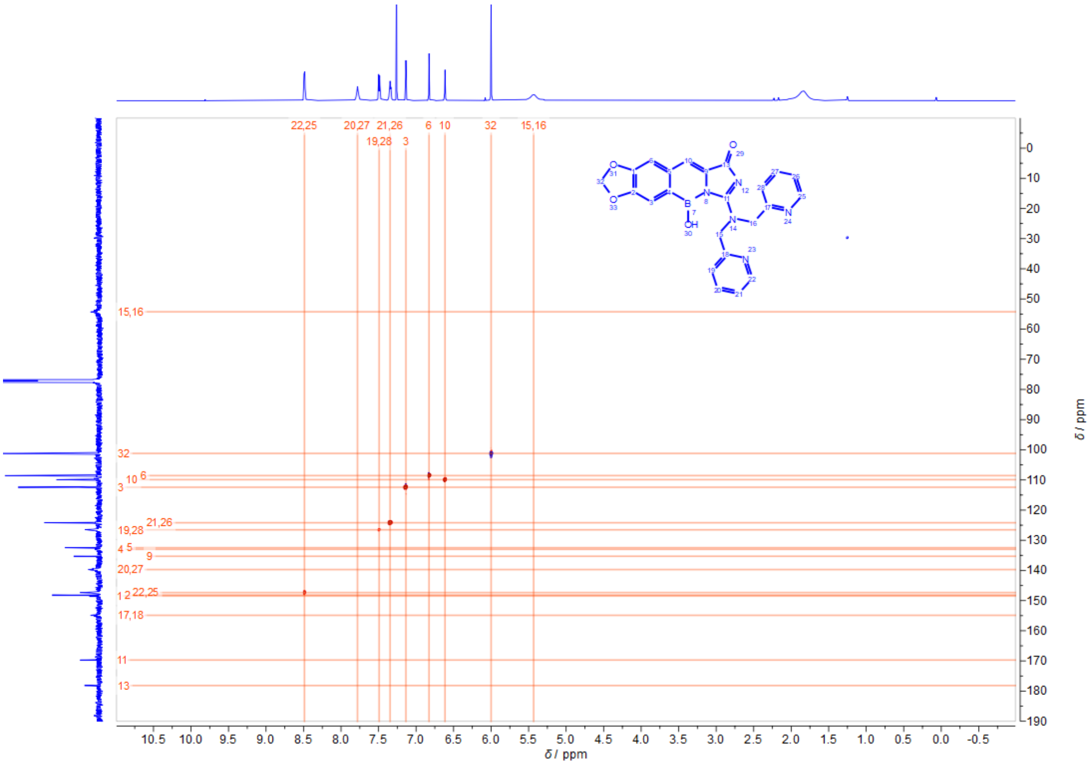

Fig S78. HSQC spectrum of **9** recorded at 600 MHz in $CDCl_3$

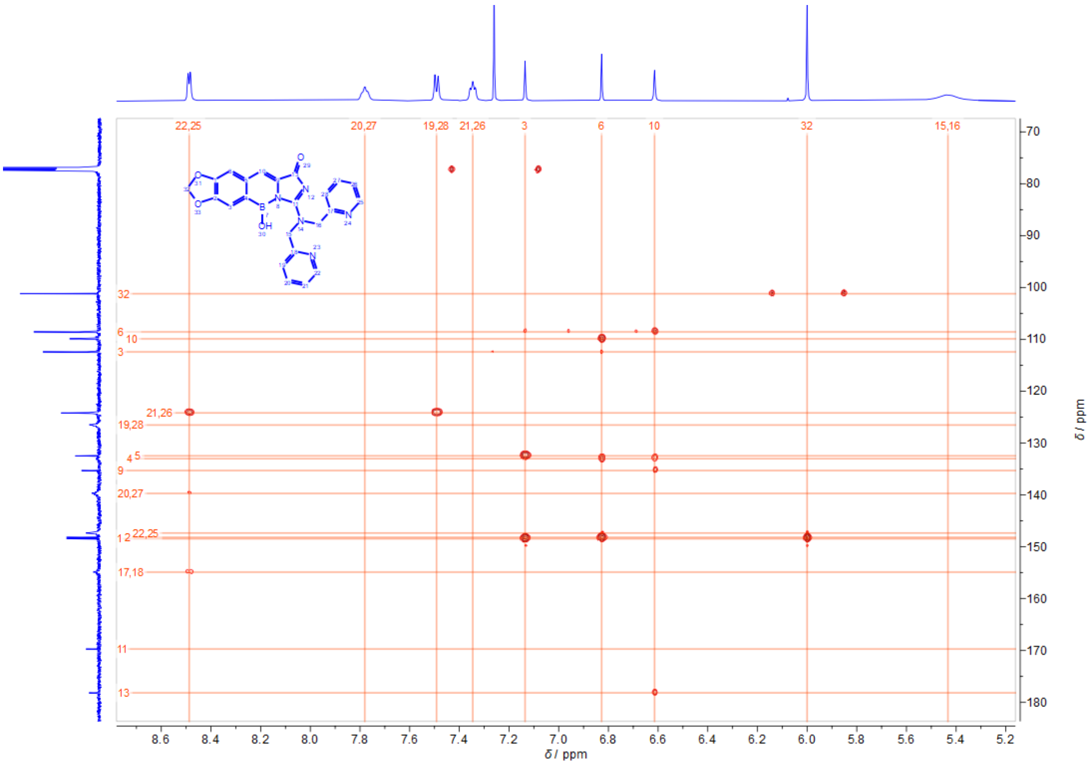

Fig S79. HMBC spectrum of **9** recorded at 600 MHz in $CDCl_3$

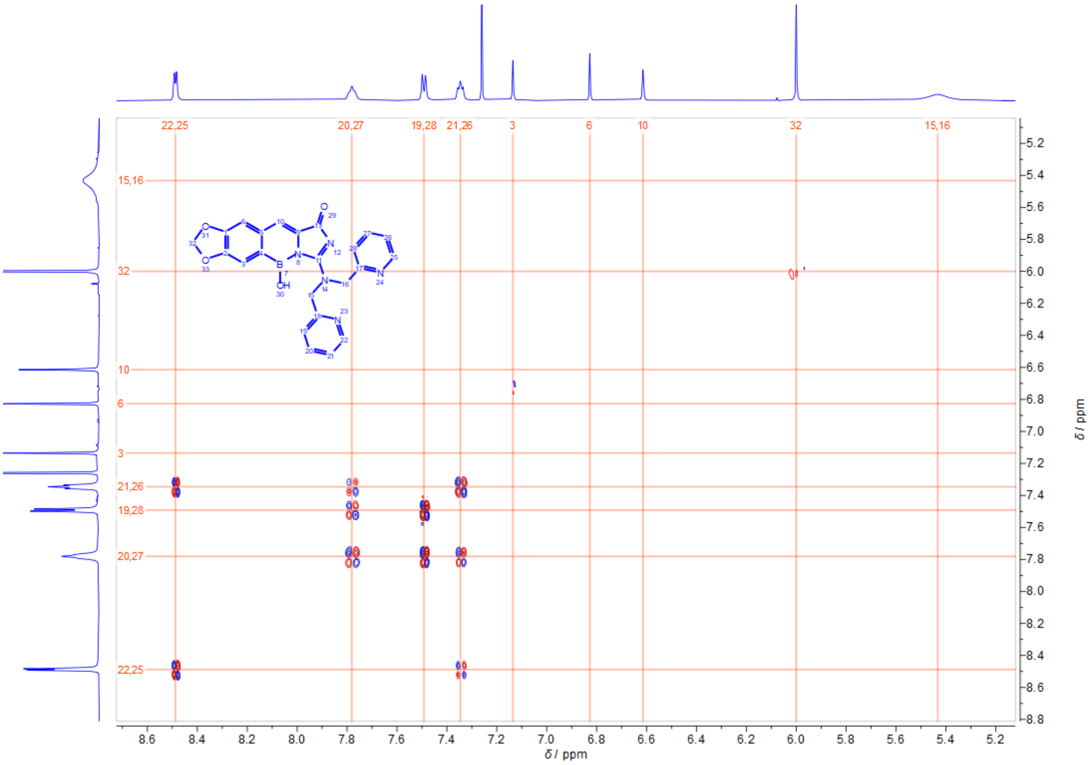

Fig S80. COSY spectrum of **9** recorded at 600 MHz in $CDCl_3$

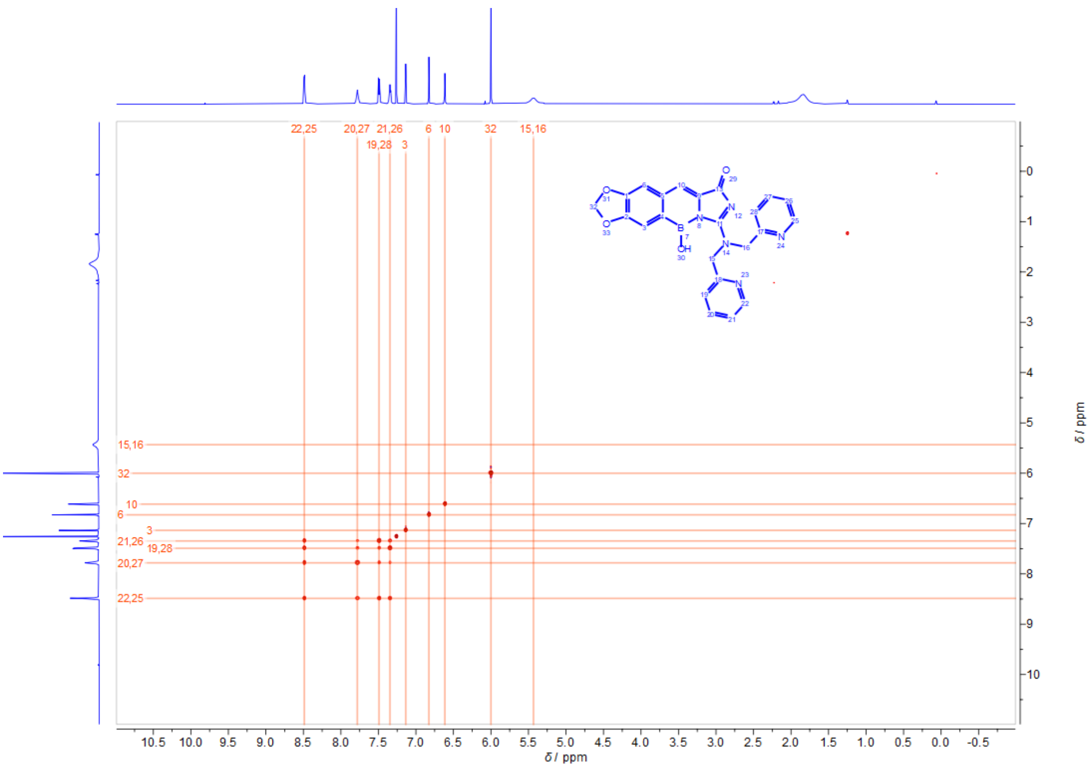

Fig S81. TOCSY spectrum of **9** recorded at 600 MHz in $CDCl_3$

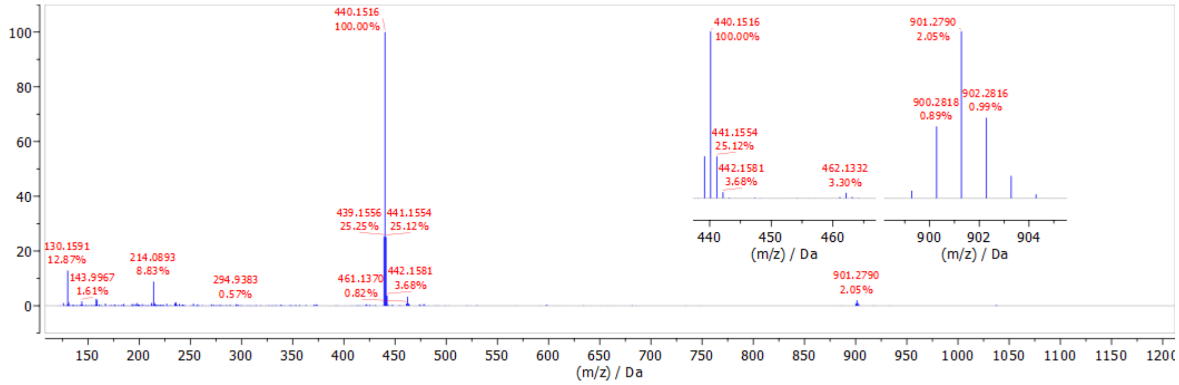

Fig S82. HRMS (ESI-Q-Orbitrap) spectrum of **9**. *m/z*: $[M + H]^+$ Calcd for $C_{23}H_{19}O_4N_5B$ 440.1525; Found 440.1516.

S2.15 NMR and HRMS spectra of S1

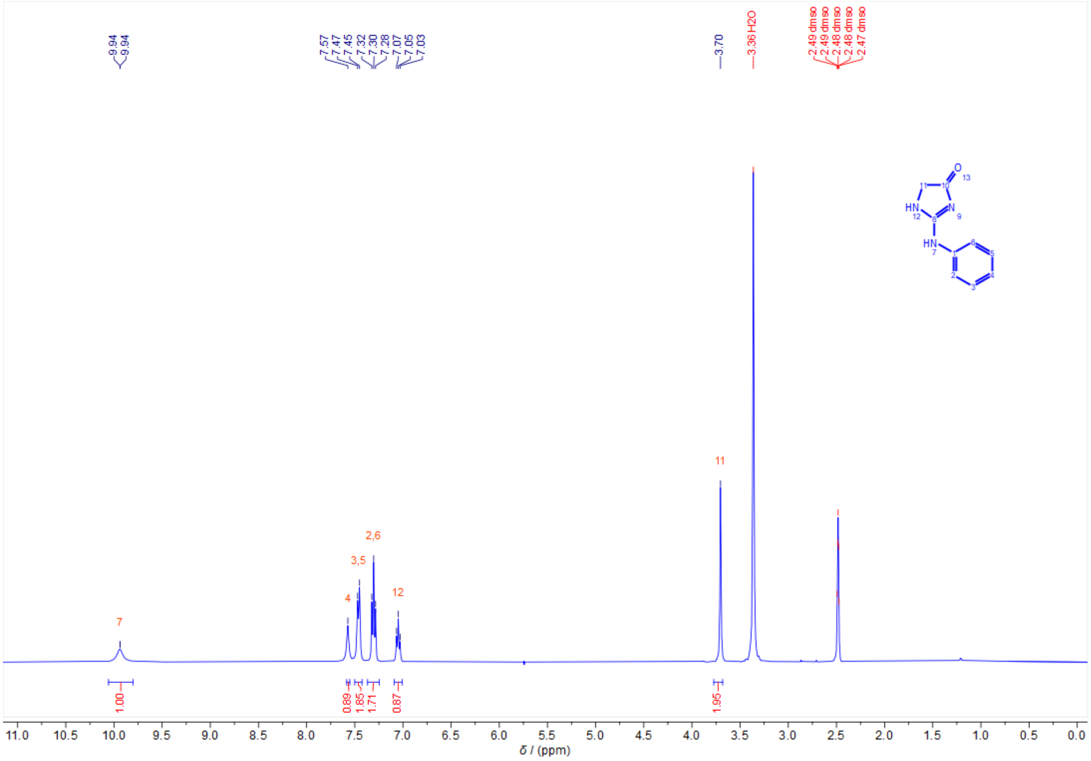

Fig S83. ^{1}H NMR spectrum of **S1** recorded at 400 MHz in DMSO-*d6*.

S2.16 NMR and HRMS spectra of S2

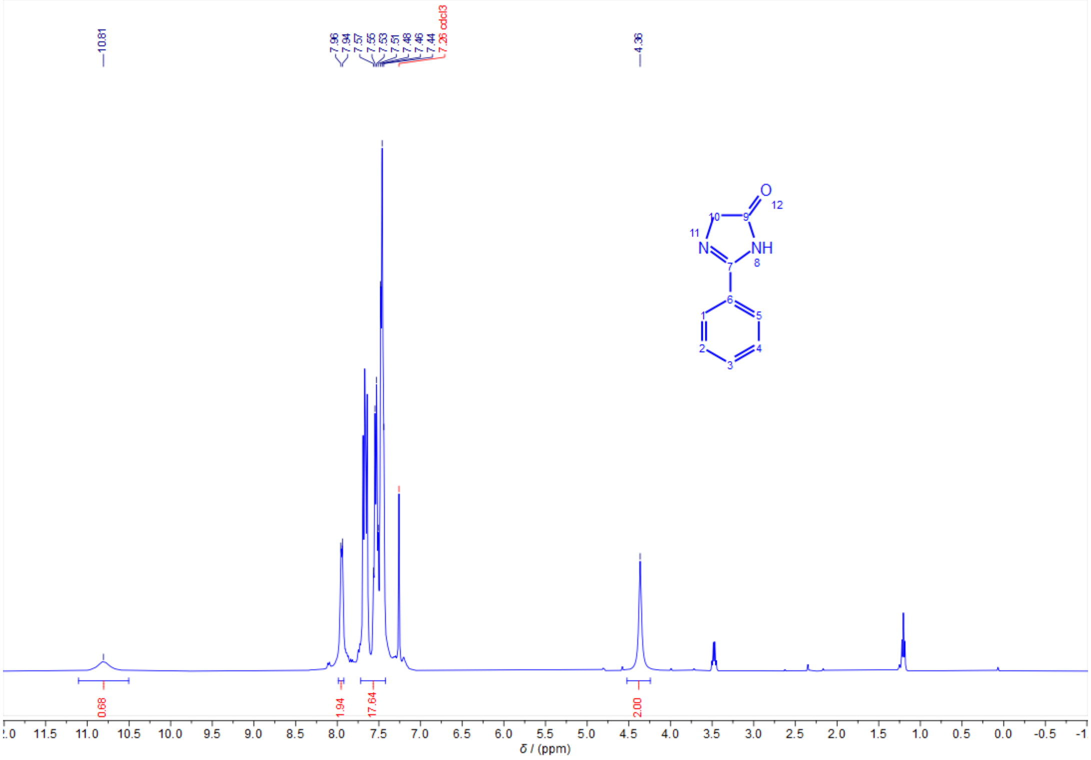

Fig S84. ^{1}H NMR spectrum of **S2** recorded at 400 MHz in $CDCl_3$.

S2.17 NMR and HRMS spectra of S3

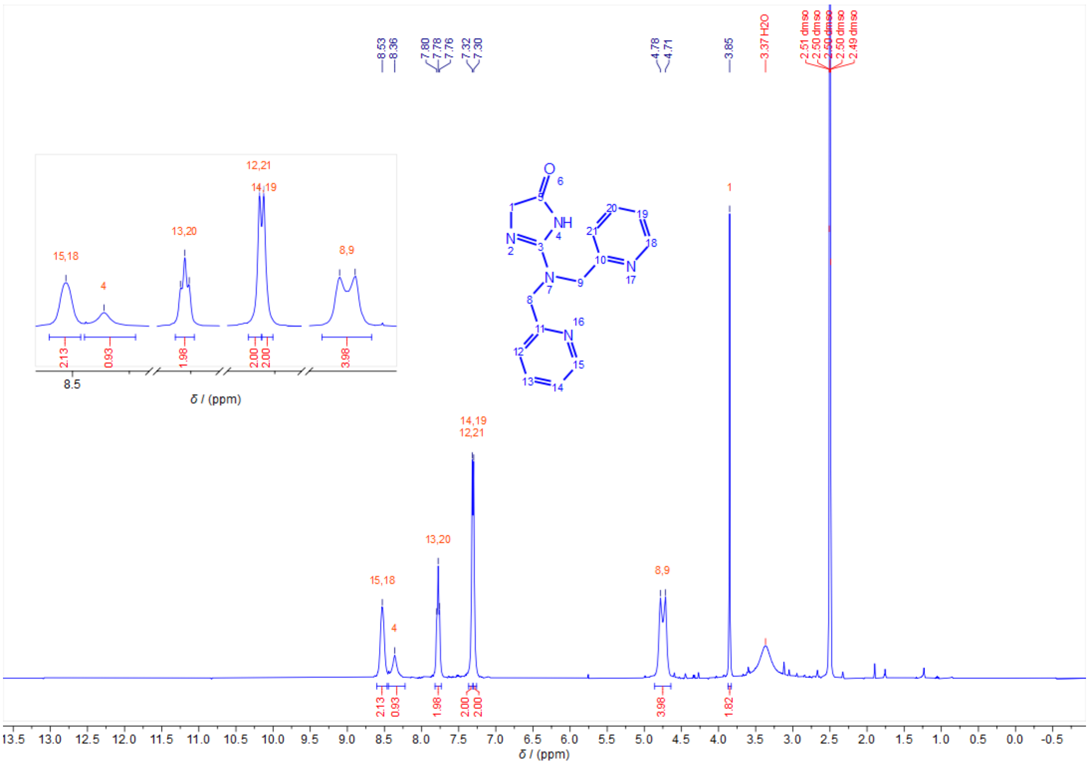

Fig S85. ^{1}H NMR spectrum of **S3** recorded at 400 MHz in DMSO-*d6*

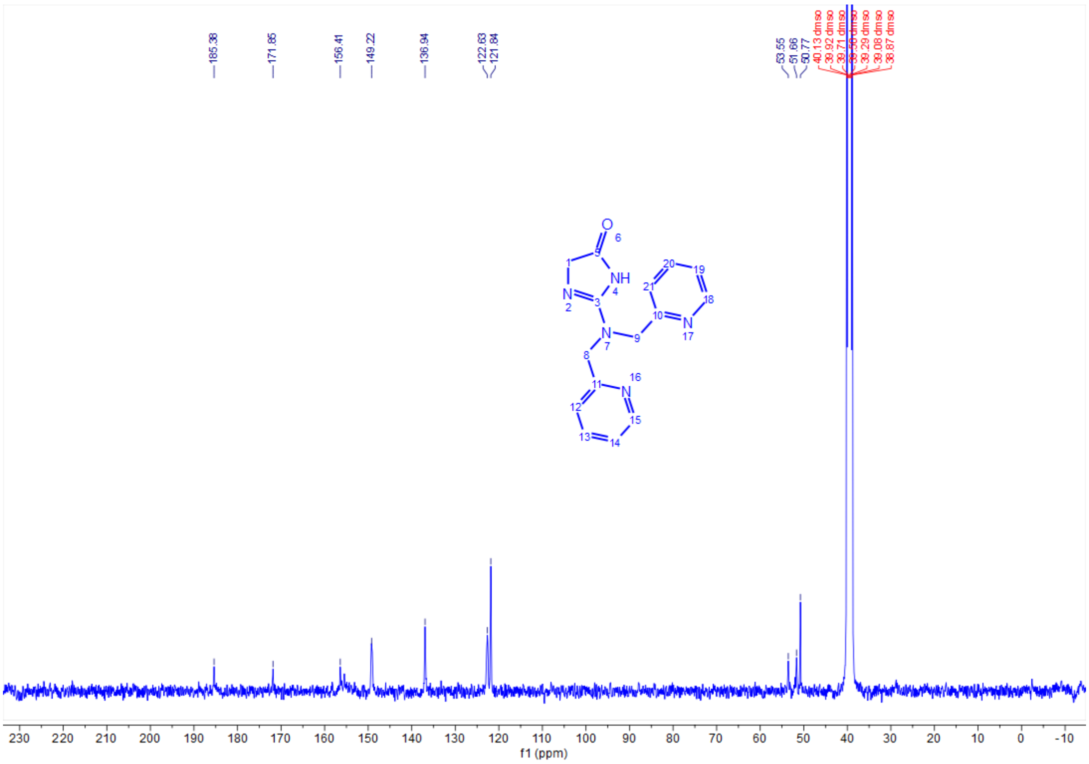

Fig S86. ^{13}C NMR spectrum of **S3** recorded at 101 MHz in DMSO-*d6*

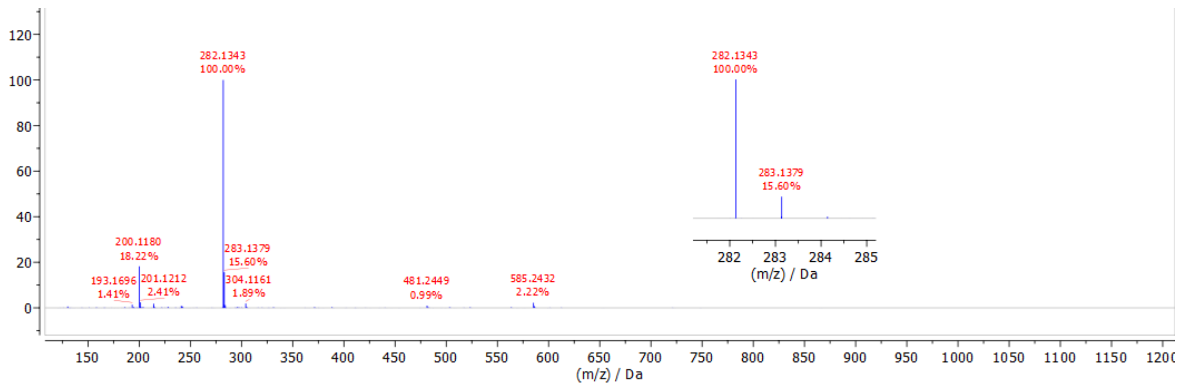

Fig S87. HRMS (ESI-Q-Orbitrap) spectrum of **S3**. *m/z*: $[M + H]^+$ Calcd for $C_{15}H_{16}ON_5$ 282.1349; Found 282.1343.

S2.18 NMR and HRMS spectra of S4

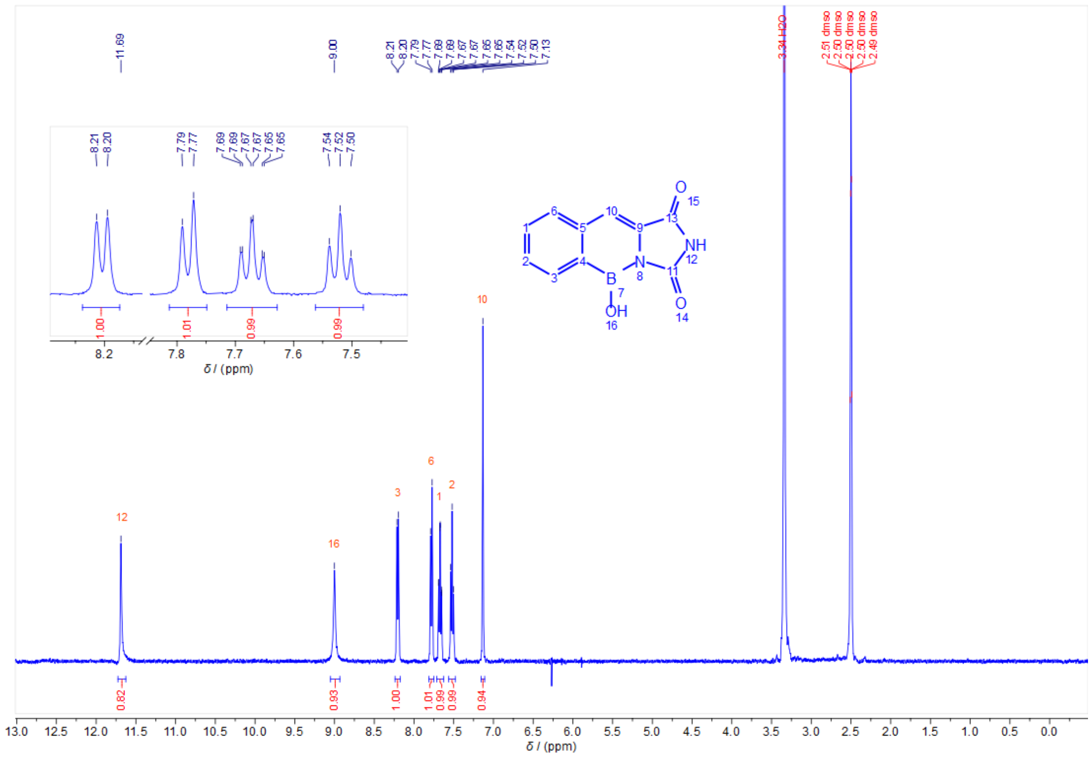

Fig S88. ^1H NMR spectrum of **S4** recorded at 600 MHz in DMSO-*d6*

S3 Coordinates of the computationally optimized structures

000aaa_5_H2O_+2H2O_B3lyp631dp_PCMw.log

Input orientation:

Center Number	Atomic Number	Atomic Type	Coordinates (Angstroms) X	Y	Z
1	8	0	1.277585	2.260990	-0.260597
2	1	0	0.545438	1.897054	0.315230
3	8	0	-0.666926	1.245528	1.260611
4	1	0	-0.493115	0.279363	1.241773
5	8	0	-0.356399	-1.493185	0.877110
6	1	0	0.531269	-1.709205	0.461728
7	8	0	1.993070	-1.998633	-0.209990
8	1	0	2.455034	-1.118670	-0.293673
9	8	0	3.193410	0.384305	-0.427974
10	1	0	2.487543	1.084439	-0.365225
11	8	0	-2.514337	-1.446756	-0.800662
12	1	0	-0.438422	-2.069491	1.648389
13	1	0	-3.227904	-1.917715	-0.349647
14	1	0	0.885259	2.322437	-1.141703
15	1	0	3.733837	0.511835	0.363030
16	1	0	-1.488668	1.333074	0.721618
17	1	0	-1.729341	-1.564824	-0.213230
18	1	0	1.853379	-2.290879	-1.120645
19	8	0	-2.882618	1.227288	-0.394464
20	1	0	-2.824678	0.258345	-0.591987
21	1	0	-2.578525	1.652859	-1.207395

000aaa_5_H2O_+3H2O_b3lyp631dp_PCMw.log

Input orientation:

Center Number	Atomic Number	Atomic Type	Coordinates (Angstroms) X	Y	Z
1	8	0	1.238101	2.403833	-0.574667
2	1	0	0.698273	2.023188	0.178143
3	8	0	-0.187399	1.355053	1.411031
4	1	0	0.006578	0.393879	1.365850
5	8	0	0.364264	-1.371953	1.159799
6	1	0	1.254789	-1.467054	0.704148
7	8	0	2.700933	-1.592592	-0.039248
8	1	0	2.985110	-0.679328	-0.322831
9	8	0	3.426988	0.867370	-0.800776
10	1	0	2.621193	1.448681	-0.722645
11	8	0	-1.638078	-2.563629	-0.262326
12	1	0	0.468971	-1.791636	2.024603
13	1	0	-1.203995	-2.978060	-1.019843
14	1	0	0.707276	2.232540	-1.363917
15	1	0	4.039441	1.209300	-0.135906
16	1	0	-1.126730	1.435183	1.115245
17	1	0	-0.899997	-2.163729	0.259543
18	1	0	2.574498	-2.078739	-0.864967
19	8	0	-2.756295	1.598518	0.472473
20	1	0	-2.894021	0.827287	-0.136843
21	1	0	-2.708266	2.361493	-0.118833
22	8	0	-3.119866	-0.501549	-1.203633
23	1	0	-4.033004	-0.790719	-1.074782
24	1	0	-2.572962	-1.259728	-0.869807

000aaa_5_H2O_+3H2O_-H+_b3lyp631dp_PCMw.log

Input orientation:

Center Number	Atomic Number	Atomic Type	Coordinates (Angstroms) X	Y	Z
1	8	0	-0.438545	-1.692601	-0.820328
2	1	0	-0.331507	-1.620319	0.158344
3	8	0	0.276610	-1.202078	1.822542
4	1	0	0.158380	-0.224497	1.735137
5	8	0	-0.064217	1.518858	1.437450
6	1	0	-1.040534	1.549244	1.338253
7	8	0	-2.818349	1.439455	0.847465
8	1	0	-2.593588	1.022283	-0.021824
9	8	0	-1.867056	0.371138	-1.447363
10	1	0	-1.361054	-0.483743	-1.209987
11	8	0	0.652453	1.675661	-1.214091
12	1	0	-0.237864	1.394253	-1.497980
13	1	0	0.472258	-1.343200	-1.135398
14	1	0	-2.428454	0.157563	-2.203399
15	1	0	1.221184	-1.340150	1.567718
16	1	0	0.260933	1.616197	0.505030
17	1	0	-3.042919	2.351038	0.617620
18	8	0	2.840128	-1.530550	0.813460
19	1	0	2.515874	-1.160924	-0.096532
20	1	0	2.874165	-2.486142	0.673371
21	8	0	1.839476	-0.622590	-1.339381
22	1	0	2.278868	-0.862872	-2.165010
23	1	0	1.191105	0.817419	-1.309917

000aab_5_H2O_+3H2O_-H+_b3lyp631dp_PCMw.log

Input orientation:

Center Number	Atomic Number	Atomic Type	Coordinates (Angstroms) X	Y	Z
1	8	0	-0.811060	-1.219354	-1.046843
2	1	0	-0.405538	-1.269551	-0.089120
3	8	0	0.210951	-1.148450	1.270881
4	1	0	0.222507	-0.166841	1.332915
5	8	0	0.143849	1.649506	0.996465
6	1	0	-0.831000	1.762888	0.939770
7	8	0	-2.640399	1.903122	0.760033
8	1	0	-2.907843	1.087565	0.264678
9	8	0	-3.310872	-0.351132	-0.624332
10	1	0	-2.416191	-0.732173	-0.832287
11	8	0	0.887904	0.961201	-1.576816
12	1	0	0.308788	0.171218	-1.560504
13	1	0	-0.682113	-2.087164	-1.451020
14	1	0	-3.686537	-0.960795	0.024757
15	1	0	1.230817	-1.420867	1.113693
16	1	0	0.421256	1.509665	0.058299
17	1	0	-2.756186	2.615843	0.117851
18	8	0	2.596416	-1.772118	0.834876
19	1	0	3.000205	-0.754937	-0.059459
20	1	0	3.059345	-1.551816	1.655186
21	8	0	3.273472	0.027526	-0.749107
22	1	0	3.552893	0.755021	-0.177688
23	1	0	1.786326	0.605895	-1.325027

000aab_5_H2O_+3H2O+H+_b3lyp631dp_PCMw.log

Input orientation:

Center Number	Atomic Number	Atomic Type	Coordinates (Angstroms) X	Y	Z
1	8	0	-2.435279	-1.999528	0.190192
2	1	0	-0.911208	-1.582445	0.735212
3	8	0	-0.010092	-1.286663	1.046004
4	1	0	0.013795	-0.262300	1.071777
5	8	0	0.100663	1.269275	1.085657
6	1	0	-0.658132	1.690957	0.554156
7	8	0	-1.838556	2.318073	-0.263833
8	1	0	-2.480863	1.603839	-0.522424
9	8	0	-3.539603	0.351683	-0.953661
10	1	0	-3.199332	-0.478889	-0.563801
11	8	0	2.589400	1.969296	0.062674
12	1	0	0.019655	1.608468	1.988942
13	1	0	2.415938	2.515264	-0.716035
14	1	0	-2.399223	-2.707243	-0.471254
15	1	0	-4.384296	0.503509	-0.508381
16	1	0	0.763105	-1.668878	0.389441
17	1	0	1.699739	1.765099	0.428587
18	1	0	-1.499578	2.666564	-1.099476
19	8	0	1.772210	-2.168580	-0.399530
20	1	0	2.504589	-1.481240	-0.534967
21	1	0	1.441015	-2.382097	-1.283164
22	8	0	3.621834	-0.374033	-0.746946
23	1	0	4.355324	-0.550577	-0.142401
24	1	0	3.257562	0.506709	-0.459369
25	1	0	-2.988220	-2.347076	0.906607

000bab_5_H2O_b3lyp631dp_PCMw_f.log

Input orientation:

Center Number	Atomic Number	Atomic Type	Coordinates (Angstroms) X	Y	Z
1	8	0	-1.804755	-1.406612	0.284608
2	1	0	-1.877878	-0.436550	0.406422
3	8	0	-1.735614	1.393529	0.457831
4	1	0	-0.017063	1.576699	-0.353632
5	8	0	0.850527	1.468930	-0.787732
6	1	0	1.418822	1.097731	-0.080643
7	8	0	2.203973	-0.302222	0.949235
8	1	0	1.715257	-0.880761	0.314248
9	8	0	0.636805	-1.326932	-1.056953
10	1	0	-0.227443	-1.461744	-0.605493
11	1	0	-2.483679	-1.616109	-0.370942
12	1	0	0.629116	-0.367521	-1.260971
13	1	0	-1.799304	1.767535	1.348250
14	1	0	1.675556	-0.343440	1.757754
15	1	0	-2.430721	1.836046	-0.049937

000cab_5_H2O_-H+_b3lyp631dp_PCMw.log

Input orientation:

Center Number	Atomic Number	Atomic Type	Coordinates (Angstroms) X	Y	Z
1	8	0	-1.253010	1.625930	-0.581779
2	1	0	-1.642457	0.293798	-0.374360
3	8	0	-1.891402	-0.743706	-0.177600
4	1	0	-0.537730	-1.213139	0.763742
5	8	0	0.306120	-1.391641	1.263220
6	1	0	0.981857	-1.327136	0.561312
7	8	0	2.072406	-0.416853	-0.810864
8	1	0	1.723781	0.373689	-0.306942
9	8	0	0.883792	1.388718	0.769256
10	1	0	0.010864	1.549128	0.211643
11	1	0	-1.847421	2.111858	0.007182
12	1	0	0.652027	0.582918	1.269445
13	1	0	-1.745758	-1.186492	-1.024164
14	1	0	1.468635	-0.486652	-1.562508

01aaa_FL_ClassIII_NHPh_alap_B3LYP631dp_PCMw.log

Input orientation:

Center Number	Atomic Number	Atomic Type	Coordinates (Angstroms) X	Y	Z
1	6	0	-5.610187	-0.397307	0.056091
2	6	0	-4.742326	0.683616	-0.025657
3	6	0	-3.346577	0.480832	-0.026787
4	6	0	-2.443796	1.610657	-0.112908
5	1	0	-2.835448	2.619914	-0.178632
6	6	0	-1.104080	1.426796	-0.112773
7	6	0	-0.013216	2.424112	-0.201963
8	7	0	-0.510285	0.165930	-0.030251
9	6	0	0.880160	0.295407	-0.052827
10	7	0	1.656358	-0.726522	-0.012794
11	8	0	-0.072055	3.637292	-0.299930
12	5	0	-1.289425	-1.063398	0.052305
13	7	0	1.150950	1.654497	-0.151570
14	6	0	-2.824346	-0.845604	0.056119
15	6	0	-3.730522	-1.916977	0.137189
16	6	0	-5.106060	-1.703927	0.138093
17	1	0	-3.342824	-2.929889	0.200348
18	1	0	-5.789427	-2.545280	0.201863
19	1	0	-5.133875	1.695032	-0.089471
20	1	0	-6.683054	-0.229509	0.056305
21	8	0	-0.674371	-2.272818	0.114250
22	1	0	0.297978	-2.140118	0.085780
23	6	0	3.061690	-0.601750	0.025501
24	6	0	3.821015	-1.417080	-0.829756
25	6	0	3.727352	0.240221	0.934343
26	6	0	5.212946	-1.362983	-0.802554
27	1	0	3.303629	-2.080923	-1.515217
28	6	0	5.122420	0.282778	0.959674
29	1	0	3.154593	0.834689	1.639824
30	6	0	5.871369	-0.511238	0.089502
31	1	0	5.785745	-1.992174	-1.477440
32	1	0	5.622248	0.935246	1.669513
33	1	0	6.956012	-0.475272	0.112439
34	1	0	2.082278	2.041102	-0.216130

01bba_FL_ClassIII_NHPh_alap_B3LYP631dp_PCMw.log

Input orientation:

Center Number	Atomic Number	Atomic Type	Coordinates (Angstroms) X	Y	Z
1	6	0	5.300618	-0.876584	-0.000092
2	6	0	4.525410	0.275663	-0.000089
3	6	0	3.118527	0.191463	-0.000020
4	6	0	2.310790	1.394300	-0.000031
5	1	0	2.785002	2.370020	-0.000088
6	6	0	0.962862	1.326332	0.000024
7	6	0	-0.074442	2.403114	0.000029
8	7	0	0.259009	0.121084	0.000092
9	6	0	-1.103787	0.487059	0.000078
10	7	0	-2.021152	-0.493738	0.000120
11	8	0	0.136552	3.610455	-0.000171
12	5	0	0.938140	-1.159035	0.000138
13	7	0	-1.325709	1.787588	0.000005
14	6	0	2.485399	-1.088739	0.000056
15	6	0	3.301707	-2.234188	0.000041
16	6	0	4.689901	-2.138743	-0.000031
17	1	0	2.849184	-3.223117	0.000079
18	1	0	5.298385	-3.037554	-0.000040
19	1	0	6.383733	-0.799235	-0.000147
20	8	0	0.139039	-2.273053	0.000227
21	1	0	0.600087	-3.120010	0.000312
22	6	0	-3.431844	-0.407871	0.000052
23	6	0	-4.130305	-1.626288	-0.000124
24	6	0	-4.140867	0.802266	0.000170
25	6	0	-5.522153	-1.635155	-0.000179
26	1	0	-3.579884	-2.563042	-0.000225
27	6	0	-5.536390	0.774689	0.000108
28	1	0	-3.599533	1.737898	0.000290
29	6	0	-6.235112	-0.433501	-0.000062
30	1	0	-6.048148	-2.584841	-0.000316
31	1	0	-6.079303	1.715228	0.000199
32	1	0	-7.320431	-0.440139	-0.000106
33	1	0	-1.631202	-1.433565	0.000173
34	1	0	4.999440	1.253159	-0.000143

01caa_FL_ClassIII_NHPh_alap_ZW_B3LYP631dp_PCMw.log

Input orientation:

Center Number	Atomic Number	Atomic Type	Coordinates (Angstroms) X	Y	Z
1	6	0	-5.549467	-0.558008	0.050457
2	6	0	-4.731450	0.560125	-0.063909
3	6	0	-3.327642	0.427131	-0.045590
4	6	0	-2.501560	1.616840	-0.172374
5	1	0	-2.969502	2.588814	-0.288324
6	6	0	-1.151149	1.533234	-0.153021
7	6	0	-0.105157	2.569478	-0.288674
8	7	0	-0.500217	0.310664	-0.004198
9	6	0	0.837026	0.501481	-0.038187
10	7	0	1.637449	-0.542325	0.059613
11	8	0	-0.183929	3.771801	-0.446018
12	5	0	-1.165380	-1.047844	0.112235
13	7	0	1.104864	1.832258	-0.197756
14	6	0	-2.734516	-0.867213	0.090623
15	6	0	-3.591316	-1.971287	0.201614
16	6	0	-4.979194	-1.830070	0.183703
17	1	0	-3.152861	-2.960482	0.303958
18	1	0	-5.618316	-2.703810	0.272449
19	1	0	-5.171546	1.548256	-0.168731
20	1	0	-6.629144	-0.443255	0.035613
21	8	0	-0.399374	-2.100225	0.199617
22	1	0	1.000893	-1.433403	0.119342
23	6	0	3.053015	-0.550523	0.091653
24	6	0	3.717996	-1.593736	-0.565954
25	6	0	3.784805	0.417244	0.793494
26	6	0	5.109755	-1.653424	-0.537574
27	1	0	3.141289	-2.341550	-1.100728
28	6	0	5.178806	0.354636	0.802430
29	1	0	3.274651	1.189777	1.359652
30	6	0	5.845554	-0.676249	0.138204
31	1	0	5.619599	-2.462831	-1.050545
32	1	0	5.740854	1.107387	1.346099
33	1	0	6.929644	-0.723194	0.153736
34	1	0	2.022700	2.241092	-0.306189

01daa_FL_ClassIII_NHPh_alap_ZW2_B3LYP631dp_PCMw.log

Input orientation:

Center Number	Atomic Number	Atomic Type	Coordinates (Angstroms) X	Y	Z
1	6	0	-5.600294	-0.518163	-0.036433
2	6	0	-4.769181	0.593884	-0.050483
3	6	0	-3.366815	0.439889	-0.017130
4	6	0	-2.506780	1.604819	-0.031681
5	1	0	-2.939568	2.598295	-0.067951
6	6	0	-1.158397	1.458713	-0.000649
7	6	0	-0.049331	2.404824	0.005929
8	7	0	-0.525461	0.221714	0.044416
9	6	0	0.865818	0.451433	0.068108
10	7	0	1.667918	-0.557790	0.122220
11	8	0	-0.251763	3.712396	-0.021416
12	5	0	-1.256203	-1.033108	0.069198
13	7	0	1.121059	1.826745	0.047065
14	6	0	-2.799758	-0.871277	0.031679
15	6	0	-3.670262	-1.974105	0.044970
16	6	0	-5.052329	-1.808782	0.011417
17	1	0	-3.249606	-2.975175	0.082098
18	1	0	-5.706769	-2.675148	0.022147
19	1	0	-5.194410	1.593061	-0.087310
20	1	0	-6.677993	-0.387467	-0.062447
21	8	0	-0.589811	-2.216359	0.123506
22	6	0	3.069191	-0.482075	0.110552
23	6	0	3.750542	-1.602655	0.623561
24	6	0	3.827181	0.582061	-0.419305
25	6	0	5.142175	-1.649944	0.637822
26	1	0	3.164433	-2.428302	1.015612
27	6	0	5.219983	0.521102	-0.410863
28	1	0	3.321925	1.445131	-0.832844
29	6	0	5.886507	-0.586683	0.119879
30	1	0	5.645627	-2.521050	1.046922
31	1	0	5.789316	1.347636	-0.826827
32	1	0	6.971600	-0.623677	0.122640
33	1	0	0.374550	-2.020463	0.143959
34	1	0	0.609257	4.164944	-0.006838

01dbb_FL_ClassIII_NHPh_alap-H+_B3LYP631dp_PCMw.log

Input orientation:

Center Number	Atomic Number	Atomic Type	Coordinates (Angstroms) X	Y	Z
1	6	0	-5.566307	-0.607719	0.000285
2	6	0	-4.761183	0.523008	0.000228
3	6	0	-3.353240	0.407063	0.000016
4	6	0	-2.514097	1.589330	-0.000049
5	1	0	-2.962932	2.577277	0.000035
6	6	0	-1.164171	1.474213	-0.000264
7	6	0	-0.071896	2.508181	-0.000498
8	7	0	-0.515207	0.256017	-0.000387
9	6	0	0.888394	0.526989	-0.000361
10	7	0	1.671079	-0.526404	-0.000283
11	8	0	-0.258776	3.731095	0.000335
12	5	0	-1.209322	-1.013849	-0.000386
13	7	0	1.137808	1.857925	-0.000164
14	6	0	-2.757849	-0.893418	-0.000151
15	6	0	-3.604523	-2.017377	-0.000090
16	6	0	-4.989691	-1.888003	0.000126
17	1	0	-3.158269	-3.008773	-0.000213
18	1	0	-5.624114	-2.769621	0.000170
19	1	0	-5.211894	1.512354	0.000350
20	1	0	-6.647555	-0.500737	0.000452
21	8	0	-0.503841	-2.181231	-0.000550
22	6	0	3.067604	-0.480700	-0.000069
23	6	0	3.723881	-1.732043	-0.000018
24	6	0	3.873470	0.681529	0.000093
25	6	0	5.112466	-1.825533	0.000184
26	1	0	3.113348	-2.630826	-0.000143
27	6	0	5.265009	0.576972	0.000295
28	1	0	3.390011	1.649574	0.000056
29	6	0	5.898016	-0.668468	0.000343
30	1	0	5.583612	-2.804959	0.000218
31	1	0	5.862179	1.485683	0.000417
32	1	0	6.981858	-0.737149	0.000501
33	1	0	0.453413	-1.913882	-0.000739

01faa_FL_ClassIII_NHPh_alap_HOH_B3LYP631dp_PCMw.log

Input orientation:

Center Number	Atomic Number	Atomic Type	Coordinates (Angstroms) X	Y	Z
1	6	0	-5.532124	-0.340857	0.021013
2	6	0	-4.650220	0.731371	-0.003482
3	6	0	-3.257121	0.510869	0.001939
4	6	0	-2.340311	1.631891	-0.024287
5	1	0	-2.719643	2.647647	-0.050029
6	6	0	-1.002579	1.430662	-0.016676
7	6	0	0.106492	2.405400	-0.048127
8	7	0	-0.424476	0.160185	0.017570
9	6	0	0.969348	0.275243	0.016487
10	7	0	1.729498	-0.759848	0.019771
11	8	0	0.067885	3.632379	-0.091247
12	5	0	-1.219250	-1.061144	0.036545
13	7	0	1.257173	1.632777	-0.019826
14	6	0	-2.751764	-0.824434	0.032262
15	6	0	-3.671960	-1.886472	0.055977
16	6	0	-5.044757	-1.655995	0.050916
17	1	0	-3.297868	-2.906161	0.078864
18	1	0	-5.739079	-2.490561	0.069893
19	1	0	-5.028378	1.749537	-0.026983
20	1	0	-6.602799	-0.159708	0.016728
21	8	0	-0.619253	-2.279540	0.052128
22	1	0	0.354959	-2.155530	0.040768
23	6	0	3.135594	-0.669292	0.079924
24	6	0	3.883356	-1.506164	-0.765217
25	6	0	3.813330	0.153354	0.997462
26	6	0	5.275701	-1.492127	-0.720232
27	1	0	3.355949	-2.155330	-1.457129
28	6	0	5.208306	0.154910	1.041727
29	1	0	3.249709	0.766691	1.693618
30	6	0	5.946174	-0.660197	0.181422
31	1	0	5.839008	-2.137552	-1.387759
32	1	0	5.717207	0.792545	1.758605
33	1	0	7.031003	-0.655538	0.219437
34	1	0	2.178839	2.079412	-0.056057
35	8	0	2.924728	3.864755	-0.114348
36	1	0	3.218321	4.027292	-1.021505
37	1	0	1.993191	4.158759	-0.110297

01fba_FL_ClassIII_NHPh_HOH_alap_B3LYP631dp_PCMw.log

Input orientation:

Center Number	Atomic Number	Atomic Type	Coordinates (Angstroms) X	Y	Z
1	6	0	-5.611268	-0.565236	-0.040722
2	6	0	-4.782340	0.548363	-0.003300
3	6	0	-3.380769	0.398688	-0.006653
4	6	0	-2.523650	1.566775	0.032623
5	1	0	-2.957185	2.560861	0.064737
6	6	0	-1.179942	1.441742	0.030036
7	6	0	-0.096718	2.471926	0.065137
8	7	0	-0.530647	0.208430	-0.009681
9	6	0	0.843366	0.512798	0.001947
10	7	0	1.713171	-0.510562	-0.030617
11	8	0	-0.254567	3.687788	0.104860
12	5	0	-1.256480	-1.055386	-0.052001
13	7	0	1.125774	1.802928	0.044631
14	6	0	-2.805451	-0.908686	-0.048891
15	6	0	-3.674815	-2.015138	-0.086115
16	6	0	-5.057858	-1.852327	-0.082298
17	1	0	-3.261587	-3.019097	-0.118549
18	1	0	-5.708183	-2.721443	-0.111495
19	1	0	-5.209655	1.546807	0.028960
20	1	0	-6.689714	-0.437156	-0.037712
21	8	0	-0.497833	-2.183778	-0.088298
22	1	0	1.266763	-1.427101	-0.061315
23	6	0	3.124698	-0.494588	-0.028460
24	6	0	3.763545	-1.744995	-0.069237
25	6	0	3.893236	0.678398	0.011422
26	6	0	5.153325	-1.822026	-0.070132
27	1	0	3.167590	-2.652992	-0.100125
28	6	0	5.285715	0.582701	0.009901
29	1	0	3.397867	1.638740	0.042699
30	6	0	5.924645	-0.657577	-0.030530
31	1	0	5.632241	-2.795845	-0.101913
32	1	0	5.874010	1.495085	0.040910
33	1	0	7.008353	-0.717473	-0.031224
34	1	0	-0.940899	-3.067341	-0.117588
35	8	0	-1.544758	-4.658069	-0.170563
36	1	0	-1.212432	-5.119623	-0.953472
37	1	0	-1.212791	-5.170375	0.580281

02aaa_FL_ClassIII_5F_NHPh_alap_B3LYP631dp_PCMw.log

Input orientation:

Center Number	Atomic Number	Atomic Type	Coordinates (Angstroms) X	Y	Z
1	6	0	-5.323899	-0.753684	0.067392
2	6	0	-4.488793	0.345418	-0.000669
3	6	0	-3.086750	0.239419	-0.007472
4	6	0	-2.252616	1.417720	-0.081166
5	1	0	-2.696394	2.403654	-0.134650
6	6	0	-0.906221	1.294239	-0.084209
7	6	0	0.136677	2.343594	-0.163346
8	7	0	-0.254258	0.062484	-0.017082
9	6	0	1.129777	0.258108	-0.039471
10	7	0	1.951954	-0.726872	-0.011233
11	8	0	0.018853	3.553067	-0.247395
12	5	0	-0.969483	-1.205497	0.051125
13	7	0	1.335459	1.629297	-0.123236
14	6	0	-2.515312	-1.066746	0.059099
15	6	0	-3.367995	-2.181039	0.127839
16	6	0	-4.752466	-2.031912	0.132493
17	1	0	-2.933743	-3.174707	0.178283
18	1	0	-5.398743	-2.902079	0.186266
19	1	0	-6.398519	-0.607965	0.068986
20	8	0	-0.298018	-2.383472	0.097977
21	1	0	0.667185	-2.204201	0.070676
22	6	0	3.350590	-0.539766	0.025665
23	6	0	4.142856	-1.316241	-0.835679
24	6	0	3.979983	0.326312	0.937537
25	6	0	5.531022	-1.200300	-0.811509
26	1	0	3.653607	-1.998849	-1.523329
27	6	0	5.371851	0.430432	0.960005
28	1	0	3.383248	0.890714	1.647799
29	6	0	6.153021	-0.324896	0.083775
30	1	0	6.129516	-1.799820	-1.491045
31	1	0	5.844037	1.100515	1.672313
32	1	0	7.235036	-0.240884	0.104557
33	1	0	2.247431	2.060188	-0.184280
34	9	0	-5.038962	1.581272	-0.063908

02baa_FL_ClassIII_5F_NHPh_alap_B3LYP631dp_PCMw.log

Input orientation:

Center Number	Atomic Number	Atomic Type	Coordinates (Angstroms) X	Y	Z
1	6	0	5.309459	-0.871505	-0.000230
2	6	0	4.511390	0.256682	-0.000236
3	6	0	3.107446	0.199994	-0.000028
4	6	0	2.311999	1.406573	-0.000043
5	1	0	2.788733	2.378749	-0.000174
6	6	0	0.964480	1.333137	0.000197
7	6	0	-0.074190	2.408566	0.000419
8	7	0	0.263504	0.126886	0.000383
9	6	0	-1.101621	0.491447	0.000323
10	7	0	-2.017246	-0.490014	0.000354
11	8	0	0.136635	3.615653	-0.000650
12	5	0	0.940695	-1.153960	0.000431
13	7	0	-1.324176	1.791623	0.000072
14	6	0	2.489942	-1.086848	0.000216
15	6	0	3.307260	-2.230797	0.000211
16	6	0	4.695534	-2.130613	-0.000005
17	1	0	2.858485	-3.220341	0.000372
18	1	0	5.309928	-3.024780	-0.000002
19	1	0	6.388393	-0.762897	-0.000401
20	8	0	0.143467	-2.267739	0.000546
21	1	0	0.602523	-3.115770	0.000667
22	6	0	-3.428254	-0.406795	0.000160
23	6	0	-4.123728	-1.626726	-0.000282
24	6	0	-4.139479	0.801918	0.000436
25	6	0	-5.515487	-1.638264	-0.000446
26	1	0	-3.571289	-2.562290	-0.000509
27	6	0	-5.534804	0.771551	0.000256
28	1	0	-3.599803	1.738410	0.000759
29	6	0	-6.230773	-0.438158	-0.000179
30	1	0	-6.039684	-2.588906	-0.000790
31	1	0	-6.079724	1.710870	0.000470
32	1	0	-7.316046	-0.447214	-0.000309
33	1	0	-1.626155	-1.429361	0.000485
34	9	0	5.102981	1.474464	-0.000455

03aaa_FL_ClassIII_4F_NHPh_alap_B3LYP631dp631dp_PCMw.log

Input orientation:

Center Number	Atomic Number	Atomic Type	Coordinates (Angstroms) X	Y	Z
1	6	0	-5.231277	-0.371870	0.045029
2	6	0	-4.387118	0.720379	-0.029694
3	6	0	-2.994499	0.504799	-0.028625
4	6	0	-2.086887	1.631725	-0.107104
5	1	0	-2.472658	2.643141	-0.168433
6	6	0	-0.748720	1.438355	-0.105069
7	6	0	0.347674	2.433265	-0.187387
8	7	0	-0.160626	0.176699	-0.028056
9	6	0	1.231097	0.300379	-0.047375
10	7	0	2.001869	-0.724890	-0.010759
11	8	0	0.291185	3.646444	-0.279389
12	5	0	-0.945682	-1.050206	0.046736
13	7	0	1.507693	1.659125	-0.138820
14	6	0	-2.477534	-0.824323	0.048370
15	6	0	-3.388515	-1.893164	0.121871
16	6	0	-4.763146	-1.685066	0.121655
17	1	0	-3.006873	-2.908042	0.180484
18	1	0	-5.470115	-2.505256	0.178510
19	8	0	-0.337513	-2.262876	0.103925
20	1	0	0.635563	-2.136333	0.078320
21	6	0	3.407990	-0.607656	0.030559
22	6	0	4.164198	-1.422536	-0.827699
23	6	0	4.075891	0.226250	0.944983
24	6	0	5.556316	-1.375368	-0.798010
25	1	0	3.644568	-2.080234	-1.517338
26	6	0	5.471105	0.261555	0.972880
27	1	0	3.504860	0.819666	1.652731
28	6	0	6.217379	-0.531616	0.099666
29	1	0	6.127140	-2.003831	-1.475207
30	1	0	5.973048	0.907642	1.687015
31	1	0	7.302140	-0.501262	0.124592
32	1	0	2.440837	2.042117	-0.199826
33	9	0	-6.566120	-0.163123	0.043921
34	1	0	-4.801984	1.721028	-0.087931

03baa_FL_ClassIII_4F_NHPh_alap_B3LYP631dp_PCMw.log

Input orientation:

Center Number	Atomic Number	Atomic Type	Coordinates (Angstroms) X	Y	Z
1	6	0	5.230022	-0.460510	-0.000870
2	6	0	4.417553	0.657931	-0.000316
3	6	0	3.020229	0.485060	0.000106
4	6	0	2.145301	1.639509	0.000239
5	1	0	2.561176	2.641125	0.000069
6	6	0	0.804137	1.490616	0.000231
7	6	0	-0.293732	2.507561	-0.000206
8	7	0	0.171036	0.248698	0.000490
9	6	0	-1.211276	0.536051	0.000478
10	7	0	-2.070761	-0.494829	0.000704
11	8	0	-0.149402	3.724256	0.000424
12	5	0	0.923373	-0.991119	0.000895
13	7	0	-1.506748	1.822078	0.000302
14	6	0	2.461860	-0.830323	0.000125
15	6	0	3.344147	-1.926569	-0.000685
16	6	0	4.723853	-1.761069	-0.001173
17	1	0	2.952311	-2.940171	-0.001140
18	1	0	5.404084	-2.605067	-0.001803
19	8	0	0.188129	-2.148030	0.002010
20	1	0	0.692340	-2.969942	0.003513
21	6	0	-3.484285	-0.490038	0.000205
22	6	0	-4.110832	-1.746687	-0.000027
23	6	0	-4.261871	0.677160	-0.000110
24	6	0	-5.499793	-1.835896	-0.000597
25	1	0	-3.507252	-2.650034	0.000191
26	6	0	-5.653349	0.569003	-0.000694
27	1	0	-3.775597	1.642490	0.000075
28	6	0	-6.280891	-0.677568	-0.000946
29	1	0	-5.970102	-2.814316	-0.000792
30	1	0	-6.249772	1.476490	-0.000957
31	1	0	-7.363979	-0.747045	-0.001412
32	1	0	-1.627504	-1.410886	0.001086
33	9	0	6.569333	-0.291285	-0.001253
34	1	0	4.860673	1.648041	-0.000282

03caa_FL_ClassIII_4F_NHPh_alap_-H+_B3LYP631dp631dp_PCMw.log

Input orientation:

Center Number	Atomic Number	Atomic Type	Coordinates (Angstroms) X	Y	Z
1	6	0	-5.209399	-0.506354	-0.082908
2	6	0	-4.420301	0.614911	0.083607
3	6	0	-3.015321	0.473512	0.078535
4	6	0	-2.161736	1.631605	0.250896
5	1	0	-2.595768	2.616618	0.385179
6	6	0	-0.813417	1.494707	0.245885
7	6	0	0.292674	2.504741	0.399837
8	7	0	-0.180672	0.281451	0.081541
9	6	0	1.227122	0.529793	0.131265
10	7	0	1.994851	-0.524232	-0.009288
11	8	0	0.118866	3.717371	0.568936
12	5	0	-0.891499	-0.966475	-0.102073
13	7	0	1.493200	1.844199	0.319308
14	6	0	-2.436491	-0.823344	-0.098263
15	6	0	-3.297047	-1.924466	-0.262699
16	6	0	-4.680673	-1.787268	-0.257995
17	1	0	-2.865465	-2.912474	-0.397494
18	1	0	-5.347150	-2.633411	-0.384404
19	8	0	-0.202478	-2.132355	-0.259257
20	1	0	0.758366	-1.882733	-0.213884
21	6	0	3.392194	-0.500285	0.008757
22	6	0	4.030468	-1.749170	-0.160975
23	6	0	4.213621	0.638266	0.178254
24	6	0	5.417612	-1.862832	-0.162479
25	1	0	3.407447	-2.629627	-0.291900
26	6	0	5.603460	0.513665	0.175252
27	1	0	3.743811	1.604070	0.309786
28	6	0	6.218887	-0.729134	0.006106
29	1	0	5.875130	-2.839615	-0.295684
30	1	0	6.213209	1.404179	0.307486
31	1	0	7.301613	-0.813541	0.005533
32	9	0	-6.557028	-0.361534	-0.076742
33	1	0	-4.885878	1.586373	0.215710

04aaa_FL_ClassIII_3Cl_NHPh_alap_B3LYP631dp_PCMw.log

Input orientation:

Center Number	Atomic Number	Atomic Type	Coordinates (Angstroms) X	Y	Z
1	6	0	-4.975446	0.610671	-0.012146
2	6	0	-3.959947	1.554266	-0.057405
3	6	0	-2.605607	1.160480	-0.051975
4	6	0	-1.552995	2.153152	-0.101263
5	1	0	-1.797570	3.208574	-0.145189
6	6	0	-0.253289	1.779710	-0.093560
7	6	0	0.969889	2.614400	-0.147101
8	7	0	0.154626	0.446746	-0.037160
9	6	0	1.551101	0.377669	-0.043691
10	7	0	2.171334	-0.745402	-0.023512
11	8	0	1.082690	3.825226	-0.215303
12	5	0	-0.787398	-0.662286	0.006587
13	7	0	2.012212	1.686453	-0.104540
14	6	0	-2.277591	-0.226776	0.001003
15	6	0	-3.317023	-1.169706	0.045646
16	6	0	-4.640200	-0.748371	0.038966
17	1	0	-3.083519	-2.228286	0.085410
18	8	0	-0.356423	-1.947794	0.044139
19	1	0	0.625451	-1.957883	0.028803
20	6	0	3.579744	-0.826898	0.027345
21	6	0	4.219991	-1.727612	-0.839331
22	6	0	4.351517	-0.107632	0.957216
23	6	0	5.604734	-1.876503	-0.802351
24	1	0	3.617887	-2.296134	-1.541071
25	6	0	5.737634	-0.268963	0.992444
26	1	0	3.864642	0.549843	1.671268
27	6	0	6.371280	-1.146578	0.111068
28	1	0	6.086435	-2.569204	-1.486094
29	1	0	6.320287	0.290040	1.718640
30	1	0	7.449375	-1.269088	0.141756
31	1	0	2.989737	1.938190	-0.152035
32	1	0	-4.212169	2.609714	-0.097581
33	17	0	-5.933647	-1.945278	0.095748
34	1	0	-6.015582	0.916276	-0.016383

04baa_FL_ClassIII_3Cl_NHPh_alap_B3LYP631dp_PCMw.log

Input orientation:

Center Number	Atomic Number	Atomic Type	Coordinates (Angstroms) X	Y	Z
1	6	0	4.996145	0.552680	-0.000032
2	6	0	4.002872	1.520448	-0.000036
3	6	0	2.639832	1.160989	-0.000029
4	6	0	1.611262	2.179364	-0.000033
5	1	0	1.882890	3.229371	-0.000043
6	6	0	0.304191	1.843067	-0.000023
7	6	0	-0.927074	2.693013	-0.000017
8	7	0	-0.145662	0.522674	-0.000009
9	6	0	-1.556307	0.611011	0.000009
10	7	0	-2.259631	-0.532026	0.000034
11	8	0	-0.958230	3.917699	-0.000034
12	5	0	0.770476	-0.598412	-0.000009
13	7	0	-2.030782	1.841495	0.000003
14	6	0	2.275407	-0.218584	-0.000016
15	6	0	3.295443	-1.185304	-0.000013
16	6	0	4.629035	-0.798372	-0.000020
17	1	0	3.058851	-2.244605	-0.000005
18	8	0	0.210855	-1.847841	-0.000015
19	1	0	0.824633	-2.591443	0.000038
20	6	0	-3.659613	-0.729092	0.000045
21	6	0	-4.101301	-2.062267	-0.000103
22	6	0	-4.594835	0.315970	0.000215
23	6	0	-5.463615	-2.347584	-0.000084
24	1	0	-3.375940	-2.871323	-0.000232
25	6	0	-5.956984	0.011405	0.000228
26	1	0	-4.250617	1.340629	0.000323
27	6	0	-6.401181	-1.311663	0.000081
28	1	0	-5.790359	-3.382845	-0.000200
29	1	0	-6.676139	0.825115	0.000359
30	1	0	-7.463465	-1.534139	0.000095
31	1	0	-1.690599	-1.375359	0.000020
32	1	0	4.279461	2.570387	-0.000045
33	17	0	5.889879	-2.028777	-0.000016
34	1	0	6.043243	0.833488	-0.000037

04caa_FL_ClassIII_3Cl_NHPh_alap_-H+_B3LYP631dp_PCMw.log

Input orientation:

Center Number	Atomic Number	Atomic Type	Coordinates (Angstroms) X	Y	Z
1	6	0	-4.975453	0.497262	-0.135848
2	6	0	-4.007705	1.471337	0.057513
3	6	0	-2.632937	1.145824	0.059581
4	6	0	-1.629337	2.169985	0.262369
5	1	0	-1.926121	3.202087	0.415367
6	6	0	-0.312761	1.848012	0.262720
7	6	0	0.922991	2.688080	0.445368
8	7	0	0.144932	0.560721	0.075323
9	6	0	1.574089	0.607464	0.139006
10	7	0	2.185608	-0.541671	-0.020276
11	8	0	0.920558	3.909221	0.640080
12	5	0	-0.726604	-0.572154	-0.139575
13	7	0	2.019860	1.866820	0.357514
14	6	0	-2.239597	-0.213255	-0.141747
15	6	0	-3.233734	-1.188120	-0.336270
16	6	0	-4.574009	-0.830850	-0.331913
17	1	0	-2.947780	-2.223668	-0.490209
18	8	0	-0.209645	-1.819377	-0.318198
19	1	0	0.777097	-1.708480	-0.261743
20	6	0	3.572065	-0.717033	0.006386
21	6	0	4.028390	-2.039943	-0.186952
22	6	0	4.544423	0.289962	0.205705
23	6	0	5.385447	-2.348853	-0.182706
24	1	0	3.288299	-2.820394	-0.340877
25	6	0	5.902613	-0.030153	0.208213
26	1	0	4.214781	1.309583	0.355593
27	6	0	6.337359	-1.343797	0.015491
28	1	0	5.701375	-3.377577	-0.334596
29	1	0	6.631094	0.761960	0.363566
30	1	0	7.397200	-1.580730	0.019506
31	1	0	-4.313127	2.502784	0.210033
32	17	0	-5.811272	-2.071468	-0.578366
33	1	0	-6.028882	0.754910	-0.136120

05aaa_FL_ClassIII_23MDOI_NHPh_alap_B3LYP631dp_PCMw.log

Input orientation:

Center Number	Atomic Number	Atomic Type	Coordinates (Angstroms) X	Y	Z
1	6	0	-4.687371	0.250279	0.053733
2	6	0	-3.772144	1.272338	-0.016024
3	6	0	-2.396387	0.914960	-0.014636
4	6	0	-1.395690	1.955969	-0.090956
5	1	0	-1.696364	2.996390	-0.149230
6	6	0	-0.075248	1.657154	-0.091853
7	6	0	1.098119	2.551748	-0.173193
8	7	0	0.400977	0.347610	-0.018563
9	6	0	1.797310	0.350094	-0.040409
10	7	0	2.477225	-0.739132	-0.009543
11	8	0	1.151598	3.766615	-0.262325
12	5	0	-0.487704	-0.804594	0.053734
13	7	0	2.189774	1.679445	-0.127757
14	6	0	-1.994401	-0.453939	0.057781
15	6	0	-2.976409	-1.471370	0.128909
16	6	0	-4.300086	-1.095009	0.124809
17	1	0	-2.690162	-2.515746	0.184739
18	8	0	0.014020	-2.067583	0.107038
19	1	0	0.994201	-2.023610	0.079820
20	6	0	3.887731	-0.744854	0.028078
21	6	0	4.567198	-1.626918	-0.828373
22	6	0	4.630472	0.030631	0.936586
23	6	0	5.958068	-1.703422	-0.802601
24	1	0	3.989220	-2.239016	-1.513605
25	6	0	6.023437	-0.057580	0.960503
26	1	0	4.116835	0.675817	1.643078
27	6	0	6.694170	-0.917668	0.089327
28	1	0	6.468803	-2.383056	-1.478423
29	1	0	6.582804	0.544779	1.670238
30	1	0	7.777441	-0.983369	0.111341
31	1	0	3.151877	1.980999	-0.192342
32	1	0	-4.084026	2.309370	-0.070464
33	8	0	-6.051018	0.338229	0.072587
34	8	0	-5.411931	-1.889759	0.190848
35	6	0	-6.547220	-1.011683	0.110804
36	1	0	-7.106969	-1.220279	-0.806719
37	1	0	-7.174232	-1.143841	0.996901

05aaa_FL_ClassIII_23MDOl_NHPh_alap_HOH_B3LYP631dp_PCMw.log

Input orientation:

Center Number	Atomic Number	Atomic Type	Coordinates (Angstroms) X	Y	Z
1	6	0	-4.707935	0.212326	0.013438
2	6	0	-3.801608	1.244472	0.003372
3	6	0	-2.422618	0.899178	0.006445
4	6	0	-1.431159	1.950947	-0.007095
5	1	0	-1.740642	2.990261	-0.020748
6	6	0	-0.107675	1.663246	-0.003134
7	6	0	1.062233	2.558909	-0.024043
8	7	0	0.380515	0.356063	0.015607
9	6	0	1.778760	0.374069	0.013938
10	7	0	2.466003	-0.711299	0.002447
11	8	0	1.110053	3.786864	-0.053073
12	5	0	-0.498193	-0.805194	0.021066
13	7	0	2.159011	1.708568	-0.005406
14	6	0	-2.008489	-0.468143	0.020269
15	6	0	-2.981593	-1.496101	0.030520
16	6	0	-4.308726	-1.131383	0.026664
17	1	0	-2.686397	-2.539449	0.041031
18	8	0	0.014499	-2.064764	0.022416
19	1	0	0.994684	-2.009275	0.010983
20	6	0	3.874993	-0.716770	0.060672
21	6	0	4.564016	-1.589502	-0.798148
22	6	0	4.607950	0.044870	0.988475
23	6	0	5.954226	-1.669757	-0.755999
24	1	0	3.993389	-2.191561	-1.498353
25	6	0	5.999927	-0.047868	1.029711
26	1	0	4.087615	0.684559	1.694541
27	6	0	6.680338	-0.897988	0.156024
28	1	0	6.472021	-2.341772	-1.434092
29	1	0	6.551369	0.543691	1.754615
30	1	0	7.763087	-0.966793	0.191600
31	1	0	3.108786	2.090631	-0.038899
32	1	0	-4.122322	2.280247	-0.006703
33	8	0	-6.072415	0.287085	0.017199
34	8	0	-5.413271	-1.938171	0.039393
35	6	0	-6.556521	-1.067574	-0.002620
36	1	0	-7.114666	-1.241630	-0.928071
37	1	0	-7.181899	-1.243524	0.877164
38	8	0	3.968746	3.833482	-0.085409
39	1	0	4.264225	3.985387	-0.993788
40	1	0	3.054721	4.178839	-0.072415

05baa_FL_ClassIII_23MDOl_NHPh_alap_B3LYP631dp_PCMw.log

Input orientation:

Center Number	Atomic Number	Atomic Type	Coordinates (Angstroms) X	Y	Z
1	6	0	-4.697921	0.195124	0.037486
2	6	0	-3.805643	1.239978	0.026439
3	6	0	-2.422886	0.916658	0.020735
4	6	0	-1.446563	1.984468	0.009087
5	1	0	-1.773887	3.018722	0.004806
6	6	0	-0.121315	1.723132	0.003329
7	6	0	1.061650	2.633972	-0.008513
8	7	0	0.394959	0.427926	0.008105
9	6	0	1.796763	0.586850	-0.000503
10	7	0	2.557348	-0.520617	0.001807
11	8	0	1.033981	3.859798	-0.015518
12	5	0	-0.468086	-0.736347	0.019693
13	7	0	2.209407	1.839685	-0.010121
14	6	0	-1.985215	-0.443412	0.026315
15	6	0	-2.947353	-1.485245	0.037728
16	6	0	-4.279006	-1.141882	0.043033
17	1	0	-2.660518	-2.532005	0.042286
18	8	0	0.160052	-1.957266	0.023438
19	6	0	3.964309	-0.648580	-0.005000
20	6	0	4.471100	-1.958572	0.000993
21	6	0	4.847844	0.440665	-0.017221
22	6	0	5.845662	-2.177198	-0.005152
23	1	0	3.785486	-2.801696	0.010471
24	6	0	6.223260	0.202883	-0.023218
25	1	0	4.453584	1.447203	-0.021760
26	6	0	6.731733	-1.096868	-0.017308
27	1	0	6.222580	-3.195291	-0.000399
28	1	0	6.901816	1.050744	-0.032672
29	1	0	7.803603	-1.267279	-0.022092
30	1	0	-4.140722	2.271063	0.022225
31	8	0	-6.061832	0.249941	0.044686
32	8	0	-5.370680	-1.966120	0.053895
33	6	0	-6.527225	-1.112173	0.055107
34	1	0	-7.121518	-1.300096	-0.844031
35	1	0	-7.111836	-1.289566	0.962686
36	1	0	2.029917	-1.390608	0.009889
37	1	0	-0.418201	-2.728437	0.031345

05baa_FL_ClassIII_23MDOl_NHPh_alap_HOH_B3LYP631dp_PCMw.log

Input orientation:

Center Number	Atomic Number	Atomic Type	Coordinates (Angstroms) X	Y	Z
1	6	0	4.729253	0.261724	-0.000645
2	6	0	3.827124	1.298238	0.000636
3	6	0	2.447196	0.961928	0.000536
4	6	0	1.464913	2.024856	0.001835
5	1	0	1.786998	3.060884	0.002956
6	6	0	0.141354	1.755309	0.001684
7	6	0	-1.048047	2.657775	0.002858
8	7	0	-0.364406	0.457275	0.000303
9	6	0	-1.763971	0.604923	0.000716
10	7	0	-2.511865	-0.511715	-0.000439
11	8	0	-1.029310	3.884844	0.004319
12	5	0	0.500241	-0.716152	-0.000962
13	7	0	-2.189660	1.855478	0.002168
14	6	0	2.020459	-0.402369	-0.000829
15	6	0	2.992412	-1.435880	-0.002093
16	6	0	4.320716	-1.077097	-0.001978
17	1	0	2.704181	-2.481610	-0.003056
18	8	0	-0.127853	-1.925018	-0.001965
19	6	0	-3.915546	-0.657242	-0.000431
20	6	0	-4.407800	-1.973121	-0.002187
21	6	0	-4.812842	0.421247	0.001219
22	6	0	-5.779775	-2.207823	-0.002302
23	1	0	-3.712312	-2.808136	-0.003469
24	6	0	-6.185387	0.167505	0.001081
25	1	0	-4.429774	1.432262	0.002558
26	6	0	-6.678803	-1.138134	-0.000665
27	1	0	-6.144666	-3.230381	-0.003683
28	1	0	-6.873981	1.007379	0.002370
29	1	0	-7.748635	-1.321141	-0.000757
30	1	0	4.152357	2.332653	0.001677
31	8	0	6.094163	0.329038	-0.000918
32	8	0	5.421833	-1.893145	-0.003148
33	6	0	6.569872	-1.029484	-0.001873
34	1	0	7.161574	-1.207726	0.901313
35	1	0	7.162393	-1.206161	-0.904808
36	1	0	-1.962928	-1.371235	-0.001490
37	1	0	0.412012	-2.752380	-0.001924
38	8	0	1.212647	-4.260273	0.000173
39	1	0	0.956681	-4.782401	0.773533
40	1	0	0.944531	-4.792919	-0.761827

05caa_FL_ClassIII_23MDOl_NHPh_alap_-H+_B3LYP631dp_PCMw.log

Input orientation:

Center Number	Atomic Number	Atomic Type	Coordinates (Angstroms) X	Y	Z
1	6	0	-4.686670	0.183847	-0.011462
2	6	0	-3.811774	1.231867	0.140297
3	6	0	-2.420266	0.933804	0.118541
4	6	0	-1.457797	2.004734	0.279335
5	1	0	-1.798987	3.026015	0.413313
6	6	0	-0.126997	1.747705	0.264379
7	6	0	1.069130	2.645793	0.404968
8	7	0	0.385007	0.477456	0.099253
9	6	0	1.809432	0.589689	0.136898
10	7	0	2.473057	-0.534006	-0.005449
11	8	0	1.018135	3.870998	0.570745
12	5	0	-0.444239	-0.695427	-0.072894
13	7	0	2.201987	1.872793	0.317526
14	6	0	-1.967690	-0.411386	-0.057930
15	6	0	-2.913495	-1.456838	-0.213383
16	6	0	-4.249832	-1.135577	-0.185249
17	1	0	-2.585598	-2.481741	-0.351609
18	8	0	0.129070	-1.924638	-0.230953
19	1	0	1.109411	-1.767330	-0.194462
20	6	0	3.866002	-0.641235	0.002369
21	6	0	4.384131	-1.944964	-0.166928
22	6	0	4.792237	0.415781	0.161544
23	6	0	5.754486	-2.187896	-0.177758
24	1	0	3.680549	-2.763773	-0.289968
25	6	0	6.164293	0.161622	0.149217
26	1	0	4.415160	1.421565	0.292480
27	6	0	6.659768	-1.133745	-0.019293
28	1	0	6.117553	-3.203727	-0.310317
29	1	0	6.855635	0.991687	0.273640
30	1	0	7.729824	-1.319170	-0.027121
31	1	0	-4.165592	2.248916	0.271616
32	8	0	-6.057378	0.222440	-0.044219
33	8	0	-5.338695	-1.963023	-0.335827
34	6	0	-6.492602	-1.147067	-0.089078
35	1	0	-7.210429	-1.273079	-0.903131
36	1	0	-6.937255	-1.419539	0.876126

101aaa_FL_ClassIII_Ph_alap_B3LYP631dp_PCMw.log

Input orientation:

Center Number	Atomic Number	Atomic Type	Coordinates (Angstroms) X	Y	Z
1	6	0	5.028570	-0.831177	0.041478
2	6	0	4.307988	0.355314	0.113545
3	6	0	2.902427	0.337796	0.040910
4	6	0	2.141567	1.566032	0.109651
5	1	0	2.650775	2.522508	0.170961
6	6	0	0.791778	1.558220	0.092529
7	6	0	-0.155522	2.701763	0.082969
8	7	0	0.005300	0.397983	0.016664
9	6	0	-1.325778	0.858140	0.006099
10	8	0	0.121831	3.893119	0.125150
11	5	0	0.650636	-0.897080	-0.205779
12	7	0	-1.452446	2.162868	0.021884
13	6	0	2.207286	-0.895351	-0.115198
14	6	0	2.966665	-2.074886	-0.184228
15	6	0	4.357960	-2.051853	-0.105918
16	1	0	2.472693	-3.037604	-0.294085
17	1	0	4.921030	-2.978240	-0.159960
18	1	0	4.824689	1.303856	0.228417
19	1	0	6.112449	-0.811016	0.100098
20	8	0	-0.121239	-1.978584	-0.494716
21	1	0	0.384322	-2.783614	-0.660057
22	6	0	-2.510828	-0.014457	0.027191
23	6	0	-3.647361	0.394563	-0.692367
24	6	0	-2.575870	-1.171494	0.819793
25	6	0	-4.816464	-0.359900	-0.646284
26	1	0	-3.596760	1.299375	-1.287700
27	6	0	-3.755755	-1.910009	0.879652
28	1	0	-1.715786	-1.482365	1.399032
29	6	0	-4.873572	-1.512790	0.141744
30	1	0	-5.683549	-0.046403	-1.219005
31	1	0	-3.802684	-2.795835	1.505139
32	1	0	-5.787821	-2.096876	0.184687

101baa_FL_ClassIII_Ph_alap_ZW_B3LYP631dp_PCMw.log

Input orientation:

Center Number	Atomic Number	Atomic Type	Coordinates (Angstroms) X	Y	Z
1	6	0	5.096477	-0.761164	-0.059904
2	6	0	4.335504	0.399744	0.017420
3	6	0	2.928172	0.321261	0.016183
4	6	0	2.134544	1.521766	0.082524
5	1	0	2.620788	2.492875	0.110108
6	6	0	0.779409	1.508192	0.095687
7	6	0	-0.108654	2.675210	0.095582
8	7	0	-0.015488	0.339813	0.063636
9	6	0	-1.300057	0.720873	0.030901
10	8	0	0.125084	3.870619	0.140601
11	5	0	0.680607	-1.097644	-0.054173
12	7	0	-1.383262	2.082860	0.028978
13	6	0	2.263958	-0.932197	-0.062422
14	6	0	3.061788	-2.080191	-0.140312
15	6	0	4.457048	-2.004369	-0.140453
16	1	0	2.570255	-3.046782	-0.201686
17	1	0	5.050206	-2.912260	-0.203702
18	1	0	4.818848	1.370969	0.079411
19	1	0	6.180497	-0.703132	-0.059080
20	8	0	0.013756	-2.181822	-0.108153
21	6	0	-2.519841	-0.085729	0.005815
22	6	0	-3.653124	0.432915	-0.654321
23	6	0	-2.612589	-1.317004	0.678822
24	6	0	-4.851815	-0.272081	-0.647092
25	1	0	-3.589884	1.366720	-1.203421
26	6	0	-3.823516	-2.004180	0.695300
27	1	0	-1.733054	-1.729987	1.151126
28	6	0	-4.941156	-1.488997	0.034407
29	1	0	-5.712928	0.126436	-1.173058
30	1	0	-3.893462	-2.948960	1.224731
31	1	0	-5.878961	-2.035764	0.045910
32	1	0	-2.246620	2.603781	0.106516

101caa_FL_ClassIII_Ph_alap-H+_B3LYP631dp_PCMw.log

Input orientation:

Center Number	Atomic Number	Atomic Type	Coordinates (Angstroms) X	Y	Z
1	6	0	5.132121	-0.713753	0.114149
2	6	0	4.358042	0.435579	-0.007180
3	6	0	2.951858	0.343720	-0.010205
4	6	0	2.121781	1.518948	-0.134635
5	1	0	2.571269	2.503754	-0.228937
6	6	0	0.771363	1.451580	-0.139699
7	6	0	-0.164563	2.595606	-0.262036
8	7	0	-0.009852	0.287984	-0.030492
9	6	0	-1.325093	0.752305	-0.088351
10	8	0	0.133402	3.787876	-0.378372
11	5	0	0.711391	-1.091230	0.113520
12	7	0	-1.443922	2.072379	-0.221104
13	6	0	2.302310	-0.911172	0.109320
14	6	0	3.114160	-2.048443	0.229784
15	6	0	4.507661	-1.962463	0.233276
16	1	0	2.626017	-3.014917	0.321967
17	1	0	5.110806	-2.861616	0.328176
18	1	0	4.832539	1.409569	-0.100205
19	1	0	6.216152	-0.642542	0.116441
20	8	0	0.138660	-2.239392	0.223189
21	6	0	-2.568418	-0.048909	-0.019330
22	6	0	-3.789063	0.658822	-0.100020
23	6	0	-2.609046	-1.447100	0.119139
24	6	0	-5.006416	-0.009132	-0.044090
25	1	0	-3.757824	1.735858	-0.206704
26	6	0	-3.838167	-2.108358	0.174281
27	1	0	-1.664721	-1.991897	0.181429
28	6	0	-5.037565	-1.401085	0.093826
29	1	0	-5.932671	0.554394	-0.107945
30	1	0	-3.855137	-3.189697	0.281511
31	1	0	-5.988528	-1.924625	0.137700

101daa_FL_ClassIII_Ph_alap+H+_B3LYP631dp_PCMw.log

Input orientation:

Center Number	Atomic Number	Atomic Type	Coordinates (Angstroms) X	Y	Z
1	6	0	5.028486	-0.807873	0.033772
2	6	0	4.303222	0.377743	0.104468
3	6	0	2.899562	0.349179	0.034646
4	6	0	2.141907	1.574356	0.105449
5	1	0	2.654652	2.528848	0.167290
6	6	0	0.789537	1.584112	0.093174
7	6	0	-0.108358	2.741012	0.104707
8	7	0	-0.008969	0.415141	0.019531
9	6	0	-1.314856	0.792298	0.011874
10	8	0	0.106314	3.931479	0.168962
11	5	0	0.657162	-0.902435	-0.205377
12	7	0	-1.393675	2.134030	0.027790
13	6	0	2.207869	-0.886422	-0.117272
14	6	0	2.967720	-2.061744	-0.185822
15	6	0	4.361786	-2.028598	-0.109738
16	1	0	2.479272	-3.026340	-0.293439
17	1	0	4.927769	-2.952731	-0.163754
18	1	0	4.815267	1.328463	0.216050
19	1	0	6.111782	-0.783709	0.089915
20	8	0	-0.133149	-1.954931	-0.485999
21	6	0	-2.508505	-0.040749	0.021973
22	6	0	-3.630200	0.383083	-0.719271
23	6	0	-2.590209	-1.193624	0.824853
24	6	0	-4.806619	-0.356450	-0.676622
25	1	0	-3.567302	1.261398	-1.353211
26	6	0	-3.780290	-1.909955	0.877429
27	1	0	-1.742440	-1.506262	1.419874
28	6	0	-4.884055	-1.499457	0.123951
29	1	0	-5.660913	-0.041476	-1.265726
30	1	0	-3.848203	-2.788461	1.509837
31	1	0	-5.806586	-2.069640	0.163232
32	1	0	0.333753	-2.781864	-0.662090
33	1	0	-2.261815	2.649417	0.115304

102aaa_FL_ClassIII_Ph_alap_+H+_B3LYP631dp_PCMw.log

Input orientation:

Center Number	Atomic Number	Atomic Type	Coordinates (Angstroms) X	Y	Z
1	6	0	5.042235	-0.832878	0.037419
2	6	0	4.317316	0.353297	0.101938
3	6	0	2.913638	0.324726	0.032290
4	6	0	2.156269	1.550418	0.096851
5	1	0	2.669195	2.505138	0.153595
6	6	0	0.803888	1.560485	0.084952
7	6	0	-0.093712	2.717650	0.090403
8	7	0	0.005054	0.391336	0.017621
9	6	0	-1.300794	0.768765	0.008339
10	8	0	0.121135	3.908405	0.148355
11	5	0	0.670842	-0.927290	-0.201409
12	7	0	-1.379247	2.110628	0.016876
13	6	0	2.221590	-0.911436	-0.113256
14	6	0	2.981087	-2.087315	-0.175608
15	6	0	4.375167	-2.054144	-0.099693
16	1	0	2.492388	-3.052346	-0.278112
17	1	0	4.940889	-2.978709	-0.148862
18	1	0	4.829591	1.304459	0.208591
19	1	0	6.125538	-0.808770	0.093430
20	8	0	-0.119559	-1.981106	-0.476988
21	1	0	0.347462	-2.808691	-0.649675
22	1	0	-2.247123	2.626823	0.102190
23	6	0	-2.494529	-0.063902	0.023625
24	6	0	-3.616887	0.356500	-0.718606
25	6	0	-2.575742	-1.212807	0.832272
26	6	0	-4.793411	-0.382519	-0.671155
27	1	0	-3.554408	1.231654	-1.356947
28	6	0	-3.765897	-1.928640	0.889490
29	1	0	-1.727526	-1.522623	1.428129
30	6	0	-4.870300	-1.521599	0.135079
31	1	0	-5.648234	-0.070226	-1.260917
32	1	0	-3.833372	-2.804019	1.526264
33	1	0	-5.792918	-2.091376	0.178051

103aaa_FL_ClassIII_Ph_alap_-H+_B3LYP631dp_PCMw.log

Input orientation:

Center Number	Atomic Number	Atomic Type	Coordinates (Angstroms) X	Y	Z
1	6	0	5.149645	-0.731180	0.120157
2	6	0	4.374776	0.416529	-0.011116
3	6	0	2.968670	0.323548	-0.014597
4	6	0	2.137795	1.497095	-0.149211
5	1	0	2.586625	2.481443	-0.251131
6	6	0	0.787436	1.428652	-0.154797
7	6	0	-0.149321	2.570880	-0.287135
8	7	0	0.007060	0.265353	-0.036740
9	6	0	-1.308492	0.728105	-0.099383
10	8	0	0.147774	3.762415	-0.412898
11	5	0	0.729189	-1.112120	0.118770
12	7	0	-1.428282	2.046969	-0.242940
13	6	0	2.319995	-0.930845	0.114504
14	6	0	3.132622	-2.066468	0.244842
15	6	0	4.526055	-1.979374	0.248835
16	1	0	2.645148	-3.032549	0.344395
17	1	0	5.129811	-2.877260	0.351516
18	1	0	4.848602	1.390104	-0.111604
19	1	0	6.233617	-0.659096	0.122797
20	8	0	0.157234	-2.259797	0.237083
21	6	0	-2.551204	-0.073550	-0.024799
22	6	0	-3.772367	0.632398	-0.112875
23	6	0	-2.590772	-1.470526	0.125721
24	6	0	-4.989206	-0.036103	-0.052404
25	1	0	-3.741950	1.708498	-0.228851
26	6	0	-3.819377	-2.132342	0.185368
27	1	0	-1.646044	-2.013962	0.193546
28	6	0	-5.019298	-1.426828	0.097569
29	1	0	-5.915875	0.526046	-0.122108
30	1	0	-3.835535	-3.212725	0.301953
31	1	0	-5.969851	-1.950802	0.145008

104aaa_FL_ClassIII_3Cl_Ph_alap_B3LYP631dp_PCMw.log

Input orientation:

Center Number	Atomic Number	Atomic Type	Coordinates (Angstroms) X	Y	Z
1	6	0	4.486068	0.602055	0.107731
2	6	0	3.503529	1.581708	0.152051
3	6	0	2.141153	1.238894	0.063929
4	6	0	1.115587	2.256475	0.109542
5	1	0	1.386964	3.305700	0.162593
6	6	0	-0.194041	1.931021	0.082952
7	6	0	-1.383744	2.820814	0.053046
8	7	0	-0.685659	0.618917	0.015851
9	6	0	-2.089673	0.753855	-0.006741
10	8	0	-1.392333	4.043623	0.081606
11	5	0	0.241984	-0.491806	-0.192755
12	7	0	-2.517756	1.992097	-0.008205
13	6	0	1.756609	-0.123391	-0.082597
14	6	0	2.762744	-1.100498	-0.124398
15	6	0	4.101770	-0.735700	-0.028218
16	1	0	2.518044	-2.153344	-0.224239
17	8	0	-0.252710	-1.721919	-0.486264
18	1	0	0.420424	-2.392361	-0.653538
19	6	0	-3.034557	-0.373431	0.022297
20	6	0	-4.231889	-0.253990	-0.705010
21	6	0	-2.827317	-1.503271	0.829786
22	6	0	-5.188832	-1.263607	-0.651534
23	1	0	-4.394247	0.629247	-1.312572
24	6	0	-3.798741	-2.499563	0.896820
25	1	0	-1.921480	-1.594022	1.415760
26	6	0	-4.974960	-2.387492	0.151300
27	1	0	-6.102478	-1.171791	-1.230229
28	1	0	-3.637697	-3.363533	1.533746
29	1	0	-5.724886	-3.171131	0.199860
30	1	0	3.789734	2.623524	0.258741
31	17	0	5.343022	-1.982893	-0.079491
32	1	0	5.535299	0.864971	0.178323

104baa_FL_ClassIII_3Cl_Ph_alap_B3LYP631dp_PCMw.log

Input orientation:

Center Number	Atomic Number	Atomic Type	Coordinates (Angstroms) X	Y	Z
1	6	0	4.535704	0.642890	0.010616
2	6	0	3.528111	1.598033	0.053656
3	6	0	2.174812	1.206976	0.051101
4	6	0	1.126409	2.194189	0.082437
5	1	0	1.374676	3.251551	0.087983
6	6	0	-0.188217	1.866716	0.087450
7	6	0	-1.319691	2.800010	0.051228
8	7	0	-0.695777	0.547062	0.078734
9	6	0	-2.035055	0.624520	0.023866
10	8	0	-1.364174	4.017276	0.068132
11	5	0	0.308606	-0.690238	0.018092
12	7	0	-2.423917	1.931240	-0.010947
13	6	0	1.814062	-0.164672	0.006038
14	6	0	2.840813	-1.112999	-0.037157
15	6	0	4.174269	-0.707008	-0.035754
16	1	0	2.584724	-2.166417	-0.071145
17	8	0	-0.074685	-1.904637	0.008195
18	6	0	-3.041803	-0.435282	0.001935
19	6	0	-4.264536	-0.182286	-0.655896
20	6	0	-2.856524	-1.658271	0.671873
21	6	0	-5.275512	-1.136399	-0.647648
22	1	0	-4.414332	0.741386	-1.205212
23	6	0	-3.884011	-2.597949	0.688741
24	1	0	-1.904527	-1.866931	1.138642
25	6	0	-5.089975	-2.343849	0.031768
26	1	0	-6.204707	-0.939041	-1.171626
27	1	0	-3.739911	-3.535695	1.215648
28	1	0	-5.882369	-3.085805	0.043798
29	1	0	3.787696	2.651993	0.089693
30	17	0	5.445892	-1.925886	-0.094347
31	1	0	5.579879	0.933256	0.011864
32	1	0	-3.384221	2.243502	0.041127

104caa_FL_ClassIII_3Cl_Ph_alap_-H+_B3LYP631dp_PCMw.log

Input orientation:

Center Number	Atomic Number	Atomic Type	Coordinates (Angstroms) X	Y	Z
1	6	0	4.552617	0.694493	0.125328
2	6	0	3.534758	1.635564	0.021118
3	6	0	2.185633	1.231332	0.011339
4	6	0	1.109264	2.187322	-0.095804
5	1	0	1.319165	3.250373	-0.171830
6	6	0	-0.188508	1.808952	-0.106182
7	6	0	-1.362325	2.710669	-0.212027
8	7	0	-0.682660	0.496656	-0.019576
9	6	0	-2.071382	0.648708	-0.073990
10	8	0	-1.343139	3.940362	-0.306742
11	5	0	0.329602	-0.681872	0.103294
12	7	0	-2.488081	1.907959	-0.184320
13	6	0	1.838867	-0.139098	0.107255
14	6	0	2.876750	-1.074075	0.211620
15	6	0	4.203963	-0.655951	0.219641
16	1	0	2.624700	-2.126619	0.285740
17	8	0	0.046170	-1.933819	0.192123
18	6	0	-3.096845	-0.417150	-0.022973
19	6	0	-4.447402	-0.006696	-0.095790
20	6	0	-2.815281	-1.789575	0.091300
21	6	0	-5.478701	-0.937114	-0.055578
22	1	0	-4.664340	1.050532	-0.183867
23	6	0	-3.859633	-2.716050	0.130798
24	1	0	-1.772311	-2.105919	0.147655
25	6	0	-5.189233	-2.301277	0.058298
26	1	0	-6.509627	-0.600408	-0.112934
27	1	0	-3.627869	-3.774048	0.219343
28	1	0	-5.994546	-3.029804	0.089841
29	1	0	3.785071	2.690474	-0.053221
30	17	0	5.494352	-1.862230	0.352781
31	1	0	5.593869	0.996697	0.133624

104daa_FL_ClassIII_3Cl_Ph_alap_+H+_B3LYP631dp_PCMw.log

Input orientation:

Center Number	Atomic Number	Atomic Type	Coordinates (Angstroms) X	Y	Z
1	6	0	4.478821	0.610572	0.105356
2	6	0	3.494938	1.590785	0.141011
3	6	0	2.136105	1.241530	0.052176
4	6	0	1.116116	2.259558	0.095077
5	1	0	1.393762	3.307289	0.146491
6	6	0	-0.200746	1.953793	0.072945
7	6	0	-1.344656	2.869428	0.061377
8	7	0	-0.704248	0.630559	0.011768
9	6	0	-2.063448	0.692291	-0.006040
10	8	0	-1.413501	4.077344	0.108124
11	5	0	0.246416	-0.498187	-0.200668
12	7	0	-2.452473	1.978066	-0.012106
13	6	0	1.753037	-0.121731	-0.087266
14	6	0	2.755762	-1.097212	-0.120763
15	6	0	4.097599	-0.727698	-0.022766
16	1	0	2.513226	-2.150508	-0.214416
17	8	0	-0.274304	-1.701743	-0.495122
18	1	0	0.366531	-2.400650	-0.677727
19	6	0	-3.025408	-0.398383	0.019478
20	6	0	-4.214755	-0.266762	-0.725836
21	6	0	-2.830274	-1.523291	0.842702
22	6	0	-5.180196	-1.265186	-0.667100
23	1	0	-4.362707	0.589850	-1.375198
24	6	0	-3.814775	-2.501906	0.911029
25	1	0	-1.933942	-1.612554	1.442513
26	6	0	-4.983320	-2.379664	0.153141
27	1	0	-6.084235	-1.173934	-1.259068
28	1	0	-3.672318	-3.359956	1.558895
29	1	0	-5.743108	-3.152844	0.204650
30	1	0	3.778478	2.633337	0.241714
31	17	0	5.332780	-1.969936	-0.063134
32	1	0	5.527085	0.875766	0.176697
33	1	0	-3.417422	2.278587	0.062911

105aaa_FL_ClassIII_23MDO_Ph_alap_B3LYP631dp_PCMw.log

Input orientation:

Center Number	Atomic Number	Atomic Type	Coordinates (Angstroms) X	Y	Z
1	6	0	4.202289	0.197820	0.006430
2	6	0	3.337836	1.263680	0.085969
3	6	0	1.949894	0.977260	0.017334
4	6	0	0.994107	2.056310	0.093231
5	1	0	1.336830	3.084216	0.155322
6	6	0	-0.337582	1.826860	0.085382
7	6	0	-1.459884	2.794649	0.086702
8	7	0	-0.916563	0.552099	0.009909
9	6	0	-2.306507	0.781012	0.010502
10	8	0	-1.387839	4.016468	0.130923
11	5	0	-0.062008	-0.614401	-0.219393
12	7	0	-2.649888	2.045740	0.033411
13	6	0	1.470935	-0.355868	-0.136944
14	6	0	2.402819	-1.418727	-0.215397
15	6	0	3.744919	-1.117283	-0.140128
16	1	0	2.093540	-2.453263	-0.323911
17	8	0	-0.647973	-1.809123	-0.506922
18	1	0	-0.023171	-2.521784	-0.685002
19	6	0	-3.329578	-0.277385	0.036519
20	6	0	-4.525610	-0.059023	-0.669655
21	6	0	-3.194438	-1.433394	0.821655
22	6	0	-5.553295	-0.996806	-0.617956
23	1	0	-4.631934	0.844929	-1.258936
24	6	0	-4.234973	-2.357717	0.887020
25	1	0	-2.288585	-1.601035	1.390183
26	6	0	-5.410728	-2.147501	0.162352
27	1	0	-6.466123	-0.827982	-1.180490
28	1	0	-4.127944	-3.242532	1.506581
29	1	0	-6.215074	-2.875305	0.209622
30	1	0	3.697510	2.280076	0.199346
31	8	0	5.566356	0.209059	0.050283
32	8	0	4.809340	-1.971992	-0.191264
33	6	0	5.990656	-1.162428	-0.053831
34	1	0	6.525272	-1.448382	0.856959
35	1	0	6.620112	-1.282449	-0.940050

105baa_FL_ClassIII_23MDO_Ph_alap_B3LYP631dp_PCMw.log

Input orientation:

Center Number	Atomic Number	Atomic Type	Coordinates (Angstroms) X	Y	Z
1	6	0	4.249096	0.218348	-0.054077
2	6	0	3.365236	1.266557	0.013345
3	6	0	1.980499	0.937987	0.019630
4	6	0	1.005394	1.987224	0.077470
5	1	0	1.327518	3.024702	0.096482
6	6	0	-0.335028	1.761318	0.093747
7	6	0	-1.391465	2.770714	0.085401
8	7	0	-0.933135	0.481836	0.071068
9	6	0	-2.263916	0.652169	0.034549
10	8	0	-1.353425	3.990428	0.119164
11	5	0	-0.016173	-0.818851	-0.017200
12	7	0	-2.558146	1.983400	0.027095
13	6	0	1.522755	-0.407096	-0.040952
14	6	0	2.469277	-1.445878	-0.110270
15	6	0	3.810730	-1.110241	-0.115695
16	1	0	2.141915	-2.478288	-0.156735
17	8	0	-0.488867	-2.003002	-0.036757
18	6	0	-3.344008	-0.334457	0.006891
19	6	0	-4.550109	0.012215	-0.637939
20	6	0	-3.241335	-1.576094	0.659477
21	6	0	-5.626749	-0.867779	-0.632882
22	1	0	-4.637298	0.951481	-1.174469
23	6	0	-4.332673	-2.441191	0.672846
24	1	0	-2.301804	-1.857931	1.113130
25	6	0	-5.522839	-2.094024	0.029726
26	1	0	-6.543413	-0.598014	-1.146692
27	1	0	-4.251081	-3.393876	1.186374
28	1	0	-6.365787	-2.778090	0.039079
29	1	0	3.702385	2.296078	0.060426
30	8	0	5.617959	0.254531	-0.076863
31	8	0	4.886104	-1.945678	-0.179037
32	6	0	6.059511	-1.110970	-0.141502
33	1	0	6.645385	-1.350865	0.751035
34	1	0	6.642922	-1.265907	-1.053752
35	1	0	-3.492420	2.363735	0.093629

105caa_FL_ClassIII_23MDO_Ph_alap_-H+_B3LYP631dp_PCMw.log

Input orientation:

Center Number	Atomic Number	Atomic Type	Coordinates (Angstroms) X	Y	Z
1	6	0	4.265940	0.227285	-0.018888
2	6	0	3.373484	1.271715	-0.073362
3	6	0	1.992280	0.935723	-0.065162
4	6	0	0.989256	1.969283	-0.114329
5	1	0	1.281012	3.014811	-0.164054
6	6	0	-0.337254	1.699201	-0.101966
7	6	0	-1.437379	2.687885	-0.152420
8	7	0	-0.926425	0.424932	-0.039867
9	6	0	-2.298848	0.680990	-0.051970
10	8	0	-1.331421	3.917217	-0.213919
11	5	0	-0.000279	-0.828468	0.008200
12	7	0	-2.621296	1.971297	-0.117062
13	6	0	1.544504	-0.408492	-0.006997
14	6	0	2.502527	-1.443763	0.043289
15	6	0	3.839689	-1.102874	0.038329
16	1	0	2.174139	-2.476513	0.084799
17	8	0	-0.383347	-2.057769	0.056306
18	6	0	-3.401361	-0.306327	0.003175
19	6	0	-4.718426	0.203276	-0.049832
20	6	0	-3.222267	-1.696441	0.110218
21	6	0	-5.816122	-0.647499	0.001240
22	1	0	-4.856868	1.274045	-0.130640
23	6	0	-4.332568	-2.542469	0.162000
24	1	0	-2.204098	-2.089379	0.143045
25	6	0	-5.628534	-2.029839	0.108255
26	1	0	-6.819521	-0.234054	-0.042121
27	1	0	-4.179195	-3.615218	0.244713
28	1	0	-6.485677	-2.696191	0.148523
29	1	0	3.705472	2.303595	-0.120787
30	8	0	5.639928	0.273106	-0.035750
31	8	0	4.934454	-1.934171	0.060423
32	6	0	6.080167	-1.076817	0.180519
33	1	0	6.501327	-1.168057	1.189719
34	1	0	6.817949	-1.345543	-0.579350

105daa_FL_ClassIII_23MDO_Ph_alap_+H+_B3LYP631dp_PCMw.log

Input orientation:

Center Number	Atomic Number	Atomic Type	Coordinates (Angstroms) X	Y	Z
1	6	0	4.195955	0.176879	0.026544
2	6	0	3.335307	1.245676	0.115489
3	6	0	1.948035	0.961287	0.044435
4	6	0	1.004763	2.041534	0.135539
5	1	0	1.358883	3.064433	0.214328
6	6	0	-0.335875	1.840746	0.126929
7	6	0	-1.404326	2.835723	0.160759
8	7	0	-0.933356	0.561023	0.033206
9	6	0	-2.283115	0.720008	0.032582
10	8	0	-1.388257	4.045387	0.245212
11	5	0	-0.064064	-0.627183	-0.212326
12	7	0	-2.577666	2.029675	0.075997
13	6	0	1.463175	-0.369388	-0.122842
14	6	0	2.384707	-1.433925	-0.210462
15	6	0	3.731255	-1.135516	-0.131668
16	1	0	2.072445	-2.465906	-0.327707
17	8	0	-0.681200	-1.786691	-0.512058
18	6	0	-3.324953	-0.297941	0.026651
19	6	0	-4.493183	-0.061919	-0.725132
20	6	0	-3.222962	-1.451299	0.825944
21	6	0	-5.532695	-0.985103	-0.694869
22	1	0	-4.569507	0.816607	-1.357443
23	6	0	-4.279325	-2.354137	0.865615
24	1	0	-2.339718	-1.622250	1.427464
25	6	0	-5.428890	-2.127840	0.102789
26	1	0	-6.421653	-0.812577	-1.291689
27	1	0	-4.207394	-3.234540	1.495058
28	1	0	-6.245646	-2.841876	0.132319
29	1	0	3.697349	2.259654	0.237930
30	8	0	5.555261	0.180102	0.071410
31	8	0	4.782814	-1.991778	-0.188825
32	6	0	5.975020	-1.191505	-0.055262
33	1	0	6.515100	-1.493298	0.845843
34	1	0	6.589454	-1.304405	-0.951983
35	1	0	-0.095900	-2.527596	-0.712686
36	1	0	-3.517096	2.397013	0.171591

106aaa_FL_ClassIII_4OMe_Ph_alap_B3LYP631dp_PCMw.log

Input orientation:

Center Number	Atomic Number	Atomic Type	Coordinates (Angstroms) X	Y	Z
1	6	0	4.381611	-0.274623	0.013339
2	6	0	3.537545	0.839153	0.091070
3	6	0	2.148850	0.681655	0.025027
4	6	0	1.274200	1.833404	0.098323
5	1	0	1.689107	2.834436	0.158057
6	6	0	-0.068148	1.695860	0.086105
7	6	0	-1.118728	2.746625	0.081222
8	7	0	-0.743535	0.467406	0.013156
9	6	0	-2.109700	0.801304	0.007296
10	8	0	-0.952131	3.958677	0.123466
11	5	0	0.023865	-0.762749	-0.211980
12	7	0	-2.358831	2.089481	0.024737
13	6	0	1.566981	-0.612038	-0.130553
14	6	0	2.444984	-1.703357	-0.205521
15	6	0	3.828903	-1.558579	-0.134452
16	1	0	2.054870	-2.712457	-0.316400
17	1	0	4.462594	-2.434374	-0.194314
18	1	0	3.979715	1.823990	0.204372
19	8	0	-0.650060	-1.911226	-0.494512
20	1	0	-0.075026	-2.666961	-0.664114
21	6	0	-3.210025	-0.176447	0.030684
22	6	0	-4.382514	0.129856	-0.682379
23	6	0	-3.166202	-1.337731	0.818497
24	6	0	-5.477798	-0.728209	-0.635113
25	1	0	-4.417572	1.037876	-1.273902
26	6	0	-4.273652	-2.181003	0.879793
27	1	0	-2.278459	-1.571749	1.392015
28	6	0	-5.426260	-1.884653	0.148058
29	1	0	-6.372238	-0.492641	-1.203132
30	1	0	-4.236877	-3.069993	1.501500
31	1	0	-6.283258	-2.549868	0.192069
32	8	0	5.711441	-0.014871	0.089744
33	6	0	6.628777	-1.108531	0.013680
34	1	0	7.622170	-0.667582	0.093455
35	1	0	6.476570	-1.814148	0.837437
36	1	0	6.540524	-1.636713	-0.941791

106baa_FL_ClassIII_4OMe_Ph_alap_B3LYP631dp_PCMw.log

Input orientation:

Center Number	Atomic Number	Atomic Type	Coordinates (Angstroms) X	Y	Z
1	6	0	4.433259	-0.253646	-0.029885
2	6	0	3.561593	0.837789	0.039276
3	6	0	2.174140	0.630674	0.041978
4	6	0	1.279115	1.759927	0.099482
5	1	0	1.677717	2.770277	0.119898
6	6	0	-0.068988	1.627189	0.111027
7	6	0	-1.052467	2.716112	0.099963
8	7	0	-0.762629	0.395719	0.084557
9	6	0	-2.074265	0.668002	0.040744
10	8	0	-0.919228	3.926978	0.138275
11	5	0	0.060146	-0.981582	-0.004355
12	7	0	-2.271337	2.018977	0.031500
13	6	0	1.616600	-0.675650	-0.022603
14	6	0	2.520509	-1.739886	-0.091629
15	6	0	3.905920	-1.553223	-0.097534
16	1	0	2.125321	-2.750328	-0.142422
17	1	0	4.561331	-2.414087	-0.154353
18	1	0	3.976130	1.839965	0.090268
19	8	0	-0.513484	-2.119834	-0.026966
20	6	0	-3.224936	-0.234667	0.007217
21	6	0	-4.394752	0.195001	-0.654313
22	6	0	-3.220106	-1.475040	0.670056
23	6	0	-5.532532	-0.604369	-0.655802
24	1	0	-4.406536	1.133420	-1.199159
25	6	0	-4.371910	-2.257698	0.677409
26	1	0	-2.308696	-1.820247	1.136900
27	6	0	-5.525823	-1.829304	0.017344
28	1	0	-6.421282	-0.273143	-1.182496
29	1	0	-4.366447	-3.209416	1.199012
30	1	0	-6.416693	-2.449699	0.021939
31	8	0	5.761831	0.044736	-0.027060
32	6	0	6.699085	-1.029836	-0.097428
33	1	0	7.685352	-0.565852	-0.083206
34	1	0	6.601991	-1.702562	0.762017
35	1	0	6.581235	-1.603848	-1.023303
36	1	0	-3.175294	2.466590	0.100315

106caa_FL_ClassIII_4OMe_Ph_alap_-H+_B3LYP631dp_PCMw.log

Input orientation:

Center Number	Atomic Number	Atomic Type	Coordinates (Angstroms) X	Y	Z
1	6	0	4.450659	-0.222996	0.022438
2	6	0	3.569869	0.859753	-0.054759
3	6	0	2.183847	0.642516	-0.047580
4	6	0	1.255659	1.747444	-0.126557
5	1	0	1.617283	2.769902	-0.193255
6	6	0	-0.083632	1.563836	-0.121390
7	6	0	-1.113342	2.629662	-0.198615
8	7	0	-0.763824	0.336671	-0.042717
9	6	0	-2.113058	0.691402	-0.074191
10	8	0	-0.915102	3.845296	-0.279053
11	5	0	0.075549	-0.983287	0.050390
12	7	0	-2.344021	2.000860	-0.163310
13	6	0	1.640159	-0.666254	0.037233
14	6	0	2.555093	-1.722501	0.112857
15	6	0	3.939344	-1.527816	0.107126
16	1	0	2.163254	-2.734112	0.178586
17	1	0	4.603728	-2.381882	0.167843
18	1	0	3.976782	1.864972	-0.119918
19	8	0	-0.401939	-2.177315	0.128539
20	6	0	-3.283786	-0.213369	-0.020280
21	6	0	-4.560850	0.389726	-0.069775
22	6	0	-3.203367	-1.613255	0.075820
23	6	0	-5.716191	-0.381349	-0.024742
24	1	0	-4.622158	1.468198	-0.143698
25	6	0	-4.370909	-2.378655	0.120400
26	1	0	-2.214530	-2.075811	0.113637
27	6	0	-5.627000	-1.774385	0.070898
28	1	0	-6.687827	0.102347	-0.064121
29	1	0	-4.294615	-3.460233	0.194683
30	1	0	-6.529351	-2.378445	0.106152
31	8	0	5.781967	0.088603	0.008643
32	6	0	6.725066	-0.977216	0.085650
33	1	0	7.709616	-0.509015	0.059880
34	1	0	6.618158	-1.543169	1.018368
35	1	0	6.628452	-1.662880	-0.764192

106daa_FL_ClassIII_4OMe_Ph_alap_+H+_B3LYP631dp_PCMw.log

Input orientation:

Center Number	Atomic Number	Atomic Type	Coordinates (Angstroms) X	Y	Z
1	6	0	4.380051	-0.269269	-0.003449
2	6	0	3.530081	0.842200	0.100106
3	6	0	2.144732	0.675493	0.039382
4	6	0	1.276223	1.824840	0.140986
5	1	0	1.696893	2.821907	0.221411
6	6	0	-0.070193	1.706513	0.132202
7	6	0	-1.071761	2.775821	0.170747
8	7	0	-0.757912	0.470532	0.035803
9	6	0	-2.090998	0.725906	0.037267
10	8	0	-0.966401	3.979372	0.260595
11	5	0	0.030646	-0.778098	-0.214373
12	7	0	-2.294278	2.055208	0.083733
13	6	0	1.564459	-0.616138	-0.136418
14	6	0	2.443669	-1.702182	-0.237285
15	6	0	3.828199	-1.550282	-0.171515
16	1	0	2.057412	-2.709736	-0.364781
17	1	0	4.463821	-2.422735	-0.252198
18	1	0	3.968143	1.826538	0.227741
19	8	0	-0.668592	-1.893495	-0.504682
20	6	0	-3.204891	-0.212354	0.027677
21	6	0	-4.357627	0.119511	-0.712520
22	6	0	-3.183679	-1.381729	0.809989
23	6	0	-5.461561	-0.725411	-0.688870
24	1	0	-4.373473	1.009837	-1.332413
25	6	0	-4.303336	-2.205035	0.843892
26	1	0	-2.312081	-1.626625	1.402255
27	6	0	-5.437321	-1.884334	0.091793
28	1	0	-6.338592	-0.479716	-1.277573
29	1	0	-4.292706	-3.097408	1.460295
30	1	0	-6.303917	-2.537161	0.116196
31	8	0	5.702424	-0.004744	0.068554
32	6	0	6.630492	-1.090627	-0.036022
33	1	0	7.619135	-0.640602	0.045412
34	1	0	6.488307	-1.811196	0.775733
35	1	0	6.537367	-1.597899	-1.001676
36	1	0	-0.131815	-2.672427	-0.698251
37	1	0	-3.205578	2.487130	0.180309

S4 References

(1) Jancsó, A.; Kovács, E.; Cseri, L.; Rózsa, B. J.; Galbács, G.; Csizmadia, I. G.; Mucsi, Z. Synthesis and Spectroscopic Characterization of Novel GFP Chromophore Analogues Based on Aminoimidazolone Derivatives. *Spectrochim. Acta - Part A Mol. Biomol. Spectrosc.* **2019**, *218*, 161–170. https://doi.org/10.1016/j.saa.2019.03.111.

(2) Wu, L.; Burgess, K. Syntheses of Highly Fluorescent GFP-Chromophore Analogues. *J. Am. Chem. Soc.* **2008**, *130* (12), 4089–4096. https://doi.org/10.1021/ja710388h.

(3) Tahtouh, T.; Durieu, E.; Villiers, B.; Bruyère, C.; Nguyen, T. L.; Fant, X.; Ahn, K. H.; Khurana, L.; Deau, E.; Lindberg, M. F.; Sévère, E.; Miege, F.; Roche, D.; Limanton, E.; L'Helgoual'ch, J. M.; Burgy, G.; Guiheneuf, S.; Herault, Y.; Kendall, D. A.; Carreaux, F.; Bazureau, J. P.; Meijer, L. Structure–Activity Relationship in the Leucettine Family of Kinase Inhibitors. *J. Med. Chem.* **2022**, *65* (2), 1396–1417. https://doi.org/10.1021/acs.jmedchem.1c01141.

(4) Gwynne, E. A.; Holt, J. C.; Dwan, J. R.; Appoh, F. E.; Vogels, C. M.; Decken, A.; Westcott, S. A. Reaction of Hydantoin with Boronic Acids. *Helv. Chim. Acta* **2010**, *93* (6), 1093–1100. https://doi.org/10.1002/hlca.201000022.

(5) Andrade-Lopez, N.; Cartas-Rosado, R.; García-Baéz, E.; Contreras, R.; Tlahuext, H. Boron Heterocycles Derived from 2- Guanidinobenzimidazole. *Heteroat. Chem.* **1998**, *9* (4), 399–409. https://doi.org/10.1002/(SICI)1098-1071(1998)9:4<399::AID-HC8>3.0.CO;2-3.

(6) Kukolich, S. G.; Sun, M.; Daly, A. M.; Luo, W.; Zakharov, L. N.; Liu, S. Y. Identification and Characterization of 1,2-BN Cyclohexene Using Microwave Spectroscopy. *Chem. Phys. Lett.* **2015**, *639*, 88–92. https://doi.org/10.1016/j.cplett.2015.09.009.

(7) Lakowicz, J.; Masters, B. Principles of Fluorescence Spectroscopy, Third Edition; 2008. https://doi.org/10.1007/978-0-387-46312-4.

(8) Makarov, N. S.; Drobizhev, M.; Rebane, A. Two-Photon Absorption Standards in the 550-1600 Nm Excitation Wavelength Range. *Opt. Express* **2008**, *16* (6), 4029. https://doi.org/10.1364/oe.16.004029.

(9) Xu, C.; Webb, W. W.; de Reguardati, S.; Pahapill, J.; Mikhailov, A.; Stepanenko, Y.; Rebane, A.; Makarov, N. S.; Drobizhev, M.; Rebane, A. High-Accuracy Reference Standards for Two-Photon Absorption in the 680–1050 Nm Wavelength Range. *Opt. Express* **2016**, *24* (8), 9053. https://doi.org/10.1364/oe.24.009053.

(10) Melnikov, A. S.; Serdobintsev, P. Y.; Vedyaykin, A. D.; Khodorkovskii, M. A. Two-Photon Absorption Cross Section for Coumarins 102, 153 and 307. *J. Phys. Conf. Ser.* **2017**, *917* (6). https://doi.org/10.1088/1742-6596/917/6/062029.

(11) Frisch, M. J.; Trucks, G. W.; Schlegel, H. B.; Scuseria, G. E.; Robb, M. A.; Cheeseman, J. R.; Scalmani, G.; Barone, V.; Mennucci, B.; Petersson, G. A.; Nakatsuji, H.; Caricato, M.; Li, X.; Hratchian, H. P.; Izmaylov, A. F.; Bloino, J.; Zheng, G.; Sonnenberg, J. L.; Hada, M.; Ehara, M.; Toyota, K.; Fukuda, R.; Hasegawa, J.; Ishida, M.; Nakajima, T.; Honda, Y.; Kitao, O.; Nakai, H.; Vreven, T.; Montgomery Jr., J. A.; Peralta, J. E.; Ogliaro, F.; Bearpark, M.; Heyd, J. J.; Brothers, E.; Kudin, K. N.; Staroverov, V. N.; Kobayashi, R.; Normand, J.; Raghavachari, K.; Rendell, A.; Burant, J. C.; Iyengar, S. S.; Tomasi, J.; Cossi, M.; Rega, N.; Millam, J. M.; Klene, M.; Knox, J. E.; Cross, J. B.;

Bakken, V.; Adamo, C.; Jaramillo, J.; Gomperts, R.; Stratmann, R. E.; Yazyev, O.; Austin, A. J.; Cammi, R.; Pomelli, C.; Ochterski, J. W.; Martin, R. L.; Morokuma, K.; Zakrzewski, V. G.; Voth, G. A.; Salvador, P.; Dannenberg, J. J.; Dapprich, S.; Daniels, A. D.; Farkas, Ö.; Foresman, J. B.; Ortiz, J. V; Cioslowski, J.; Fox, D. J. Gaussian 16, Revision C.01. *Gaussian Inc Wallingford CT*. 2016.

(12) Tomasi, J.; Mennucci, B.; Cammi, R. Quantum Mechanical Continuum Solvation Models. *Chem. Rev.* **2005**, *105* (8), 2999–3094. https://doi.org/10.1021/cr9904009.

(13) Becke, A. D. Density-Functional Thermochemistry. III. The Role of Exact Exchange. *J. Chem. Phys.* **1993**, *98* (7), 5648–5652. https://doi.org/10.1063/1.464913.

(14) Mucsi, Z.; Viskolcz, B.; Csizmadia, I. G. A Quantitative Scale for the Degree of Aromaticity and Antiaromaticity: A Comparison of Theoretical and Experimental Enthalpies of Hydrogenation. *J. Phys. Chem. A* **2007**, *111* (6), 1123–1132. https://doi.org/10.1021/jp0657686.

(15) Mucsi, Z.; Chass, G. A.; Viskolcz, B.; Csizmadia, I. G. Quantitative Scale for the Extent of Conjugation of Carbonyl Groups: “Carbonylicity” Percentage as a Chemical Driving Force. *J. Phys. Chem. A* **2008**, *112* (38), 9153–9165. https://doi.org/10.1021/jp8048586.

(16) Mucsi, Z.; Chass, G. A.; Viskolcz, B.; Csizmadia, I. G. A Quantitative Scale for the Extent of Conjugation of Substituted Olefines. *J. Phys. Chem. A* **2009**, *113* (27), 7953–7962. https://doi.org/10.1021/jp805325a.

(17) Kovács, E.; Rózsa, B.; Csomos, A.; Csizmadia, I. G.; Mucsi, Z. Amide Activation in Ground and Excited States. *Molecules* **2018**. *23* (11), 2859. https://doi.org/10.3390/molecules23112859.
